# Precisely predicting the ^1^H and ^13^C NMR chemical shifts in new types of nerve agents and building spectra database

**DOI:** 10.1038/s41598-022-24647-y

**Published:** 2022-11-24

**Authors:** Keunhong Jeong, Tae In Ryu, Seung-Ryul Hwang, Yoonjae Cho, Kyoung Chan Lim, Ung Hwi Yoon, Jin-Young Lee, Young Wook Yoon, Hey Jin Jeong

**Affiliations:** 1 Department of Physics and Chemistry, Korea Military Academy, Seoul, 01805 South Korea; 2 Accident Coordination and Training Division, National Institute of Chemical Safety (NICS), 90 Gajeongbuk-Ro, Yuseong-Gu, Daejeon, 34114 South Korea; 3 Agency for Defense Development (ADD), Yuseong-Gu, P.O. Box 35, Daejeon, 34186 South Korea

**Keywords:** Chemical safety, Computational chemistry

## Abstract

Following the recent terrorist attacks using Novichok agents and the subsequent decomposition operations, understanding the chemical structures of nerve agents has become important. To mitigate the ever-evolving threat of new variants, the Organization for the Prohibition of Chemical Weapons has updated the list of Schedule 1 substances defined by the Chemical Weapons Convention. However, owing to the several possible structures for each listed substance, obtaining an exhaustive dataset is almost impossible. Therefore, we propose a nuclear magnetic resonance-based prediction method for ^1^H and ^13^C NMR chemical shifts of Novichok agents based on conformational and density functional study calculations. Four organophosphorus compounds and five G- and V-type nerve agents were used to evaluate the accuracy of the proposed procedure. Moreover, ^1^H and ^13^C NMR prediction results for an additional 83 Novichok candidates were compiled as a database to aid future research and identification. Further, this is the first study to successfully predict the NMR chemical shifts of Novichok agents, with an exceptional agreement between predicted and experimental data. The conclusions enable the prediction of all possible structures of Novichok agents and can serve as a firm foundation for preparation against future terrorist attacks using new variants of nerve agents.

## Introduction

Following the assassination of a former Russian spy in Salisbury, United Kingdom in 2018 using Novichok, a deadly nerve agent, a significant amount of time was spent to characterize the exact structure of the Novichok compound to aid the comprehensive identification of the contaminated area and facilitate subsequent decontamination^1–6^. Therefore, the characterization of the exact structures of such chemicals is now of paramount importance to ensure the same lack of preparedness is not repeated. In addition, as exposure to the nerve agent cannot be detected based on color or smell, the development of an appropriate detection method and a framework for the short-term storage of such data is also essential. Nuclear magnetic resonance (NMR) spectroscopy is one of the most powerful tools for the detection and characterization of unknown materials as it is widely used for structural elucidation as well as qualitative and quantitative analysis of hazardous materials^7–9^. In spite of its dependence on mixture analysis, NMR spectroscopy exhibits several advantages over other detection methods: it is non-destructive, no standards are necessary for quantification, and it expresses chemical shifts and coupling constants as functions of the nucleus and its environment^8,9^. However, the practical application of NMR-based analytical and structural methods for the detection and analysis of nerve agents is severely limited by the risk of exposure to highly toxic Novichok agents^10^. Recently, the Chemical Weapons Convention (CWC) list of Schedule 1 substances has been updated with Novichok derivatives (the list is not named Novichok). However, the structures of these substances have not been clearly defined; updated with alkyl groups of limited length (≤ 10 carbons, cycloalkyl group), the list could refer to more than ten thousand derivatives^11^. Naturally, the synthesis of all these variants and their NMR characterization would require extensive time and effort. Moreover, Alex Navalny’s poisoning, which involved new types of nerve agents, demonstrated the insufficiency of even the updated CWC Schedule 1 list in coping with future chemical warfare. Thus, reliable theoretical studies to create a comprehensive database are essential to reduce experimental effort and to prepare against the use of new variants of nerve agents.

Density functional theory (DFT) calculations have been used to predict the NMR chemical shifts of organic compounds with high accuracy^14,15^. Further, notable recent advances, particularly ones accounting for the effects of conformational isomers, have enhanced the accuracy of NMR chemical shift predictions^12,13^. Therefore, the creation of an NMR spectral database of Novichok candidates and its utilization to detect and identify new variants of nerve agents appears feasible. In this study, we report a reliable method to predict ^1^H and ^13^C NMR spectra by applying both conformational study and DFT calculations to Novichok candidates (A-230 : methyl-(1-(diethylamino)ethylidene)phosphonamidofluoridate, A-232 : methoxy-(1-(diethylamino)ethylidene)phosphoramidofluoridate, and A-234 : ethyl *N*-[(1E)-1-(diethylamino)ethylidene]-phosphoramidofluoridate). To this end, we evaluated an optimal NMR spectra calculation method for organophosphorus (OP) compounds, including G- (German) and V-(Venomous) series nerve agents, by comparing the experimental and calculated values. To the best of our knowledge, although simple DFT calculations related to NMR prediction for single Novichok candidates have been reported previously^14^, no study has predicted the ^1^H and ^13^C NMR chemical shifts of Novichok candidates based on optimized structures or performed NMR spectral calculations to obtain the big data of both NMR chemical shifts based on the chloroform solvent system. Moreover, the actual NMR chemical shifts from Novichok agents were compared with the predicted NMR chemical shifts.

## Methods

### Chemicals and materials

Methyldiphenylphosphine oxide, phenylphosphonic dichloride, triphenylphosphine oxide, and triethylphosphine oxide were purchased from Sigma-Aldrich and were used without further purification. Chloroform-d (CDCl3, 99.8 atom % D, Eurisotop), for use in NMR spectroscopy as the solvent, was also used as received.

### Instruments

Spectra from all samples were obtained using a Bruker Avance III NMR spectrometer operating at a ^1^H resonance frequency of 300 MHz and a ^13^C resonance frequency of 75 MHz. The chemical shift was referenced to the residual CH peak of CDCl_3_ (δ = 7.26, proton; δ = 77.06, ^13^C).

### Computational methods

During the first step of the calculation, for comparison with experimental data within a threshold of 4.0 kcal/mol relative energy, the conformational isomers were extracted by generating isomers for each target material structure; the search for this conformation method was systematically performed using Discovery Studio 2021. Each isomer was separated from the other conformational isomers for further DFT calculations, which were performed using the Gaussian 16 software package^15^. The obtained conformers were accounted for in the subsequent optimization of NMR chemical shift predictions, which were carried out for the case of two different solvent systems (chloroform and DMSO), using the M06-2X/6-311 + G(2d,p) and MPW1PW91/6-311 + G(2d,p) functionals/basis set combinations for the chloroform solvent system and B3LYP/6-31 + G(d,p) and PBE0/6-311 + G(2d,p) functional/basis set combinations for the DMSO solvent system^16^. The different levels of theory for predicting the NMR chemical shift in each solvent system were compared, and the scaling factors for each level of theory are presented in the Chemical shift repository by Tantillo^17^. Among them, the most accurate albeit expensive methods and basis sets were selected from the recommendation series for the ^1^H and ^13^C NMR chemical shift prediction of each structure^18^. That accounts for why different methods and basis sets were used for the different solvent systems. Further, chloroform is normally used for detecting/identifying nerve agents, which was used as the solvent system for comparing the NMR chemical shifts of G- and V-series nerve agents and building the big data of the Novichok candidates. Whereas, dimethylsulfoxide was used as an NMR solvent for obtaining the experimental NMR chemical shifts of Novichok agents due to the hazardous conditions required for preparing the sample. The SMD implicit solvent model was used, and an ultrafine integration grid was applied. All the frequency calculations were also performed for all conformers, and no imaginary frequency was produced, which indicates the local minima of each structure. The final ^1^H and ^13^C NMR chemical shift data were further tuned by taking into consideration the influence of each conformer on the total Boltzmann distribution when accounting for the relative energies. The calculated ^1^H and ^13^C chemical shifts were calibrated using an existing standard approach (scaling factors) on the chemical shift repository^16,18^.

The credibility of the proposed method was tested in cases with progressively increasing complexities. The method was first tested for validity in the case of the OP compounds, followed by testing for validity in the case of actual nerve agents, and finally, after establishing the validity of the method for both, it was used in the case of novel Novichok agents. This was followed by the creation of a database with prediction data for the NMR spectra of 83 chosen Novichok candidates.

## Results and discussion

### Experimental and computational results for four OP compounds

**Figure 1:**
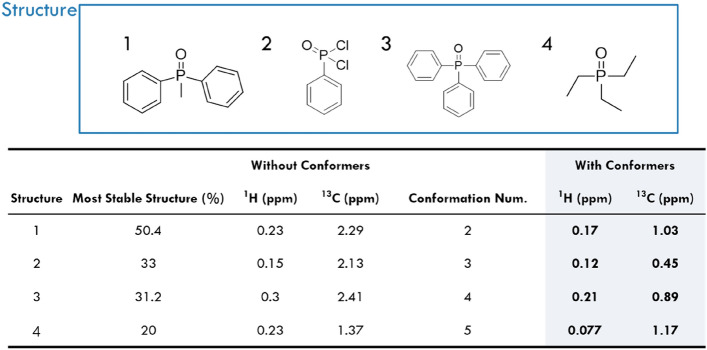
Experimental and calculated OP structures and the average MAE between experimental and calculated data, both with and without conformer considerations. Chloroform was used for experimental sampling; therefore, the M06-2X/6-311 + G(2d,p) and MPW1PW91/6-311 + G(2d,p) functionals/basis set combinations were used for the DFT study.

Prior to DFT calculations for real nerve agents, the reliability of the method was experimentally verified. For this, four low-toxicity and available OP compounds (methyldiphenylphosphine oxide, phenylphosphonic dichloride, triphenylphosphine oxide, and triethylphosphine oxide) were selected based on the main structure of the organophosphorus backbone. Then, their ^1^H and ^13^C NMR spectra in the chloroform solvent were obtained. The empirical values of the chemical shifts were extracted from the spectra and compared with the calculated values (see Supporting Information for further details). The average mean absolute error (MAE) was calculated and is shown in Fig. 1. The complexity of the studied OP structures is comparable to those of nerve agents (this is discussed in greater detail in the following section), with less than five conformational isomers, which were considered for the Boltzmann distribution (Fig. 1). Notably, even though only the conformational isomer with the most stable structure from the Boltzmann distribution was considered for NMR chemical shift prediction (without conformers), it significantly enhanced the accuracy of each predicted nuclear spin after considering the possible conformers from each OP structure in the experimental sample (Fig. 1). Overall, the predicted ^1^H NMR chemical shift MAE was less than 0.21 ppm and the ^13^C NMR chemical shift MAE was less than 1.2 ppm. These metrics confirm the high prediction accuracy achieved using DFT calculations supplemented with conformational isomer considerations.

### Experimental and computational results for nerve agents

**Figure 2:**
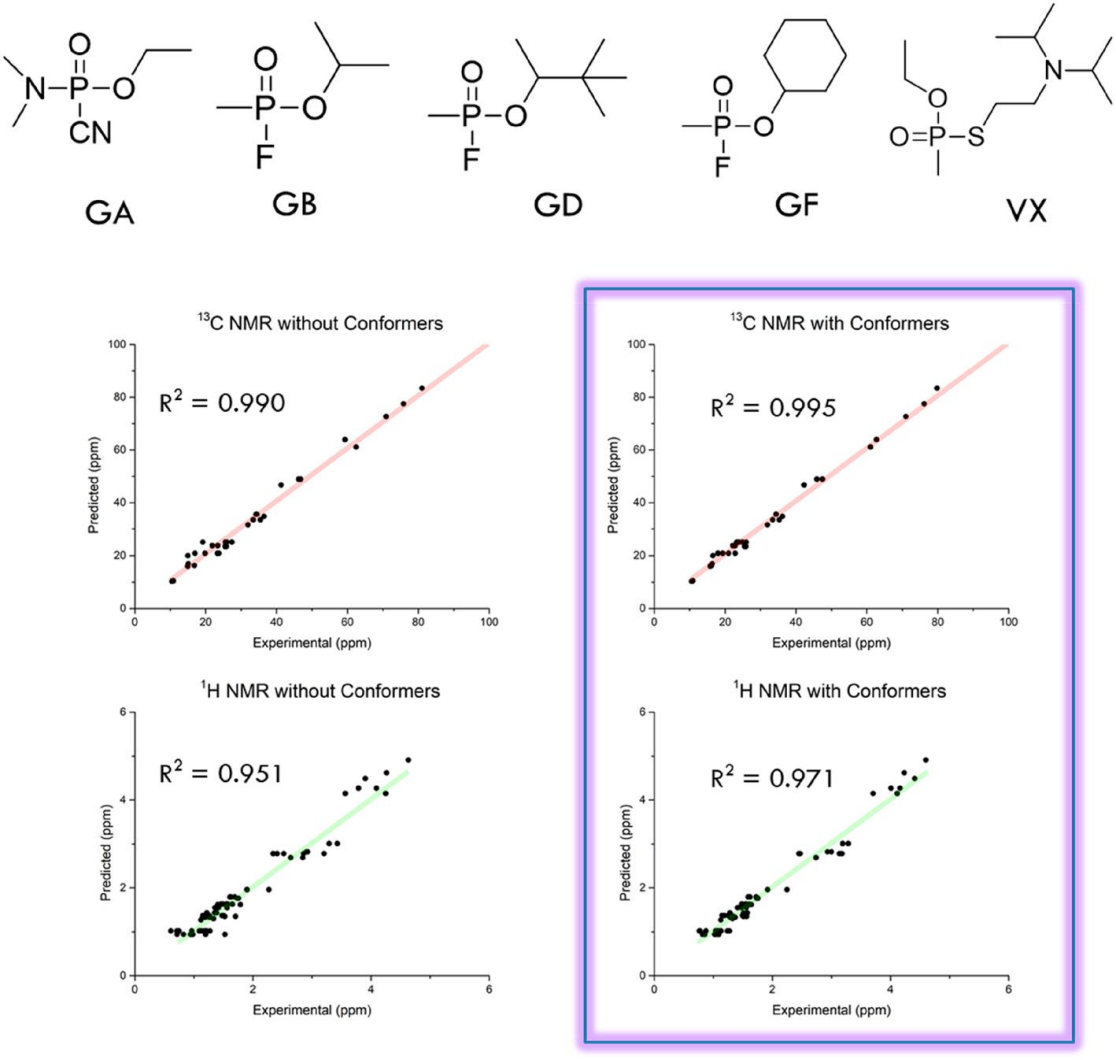
Calculated structures of nerve agents and the ^1^H and ^13^C NMR chemical shift correlation between experimental and predicted data. Chloroform was used for experimental sampling; therefore, the M06-2X/6-311 + G(2d,p) and MPW1PW91/6-311 + G(2d,p) functionals/basis set combinations were used for the DFT study.

After successfully validating the computational method for the case of the four OP compounds, in this step, the predicted and experimental data corresponding to the five selected nerve agents were now compared to validate the method for the case of actual nerve agents. Experimental ^1^H and ^13^C NMR spectra of the five nerve agents (GA (tabun, ethyl dimethylphosphoramidocyanidate), GB (sarin, propan-2-yl methylphosphonofluoridate), GD (soman, 3,3-Dimethylbutan-2-yl methylphosphonofluoridate), GF (cyclosarin, cyclohexyl methylphosphonofluoridate), and VX (venomous agent X, 2-(Diisopropylamino)ethyl]-*O*-ethyl methylphosphonothioate) in CDCl_3_, previously obtained by the Organization for the Prohibition of Chemical Weapons (OPCW) and previous reports^19^, were used instead of conducting new experiments to mitigate the risk of exposure during analysis—the reliability of the proposed method was confirmed by comparing the data with the calculated ^1^H and ^13^C NMR chemical shifts. The results are shown in Tables 1 and Fig. 2. The number of conformational isomers was higher for more complex nerve agents, with GA exhibiting a maximum of 15 isomers. All contributions from the Boltzmann distribution were considered during NMR chemical shift prediction (for further details, please consult the Supporting Information section).

**Table 1:** The number of conformational isomers of each nerve agent and the average MAE of ^1^H and ^13^C NMR between experimental and predicted data (all conformations are considered with Boltzmann factor).

	Conformation Num	MAE (ppm)	
		^1^H	^13^C
GA (Tabun)	15	0.25	0.95
GB (Sarin)	7	0.125	1.17
GD (Soman)	14	0.087	1.25
GF (Cyclosarin)	5	0.11	1.28
VX	13	0.143	1.76

To compare the Boltzmann distribution factors of the nerve agents, all predicted and experimental ^1^H and ^13^C data were plotted in a linear correlation (Fig. 2). It is worth noting that consideration of conformational isomers was observed to noticeably enhance the accuracy of predicted values, and ^13^C NMR prediction was more accurate than ^1^H NMR prediction (for further details, please consult the Supporting Information section).

### Predicting ^1^H and ^13^C NMR spectra of Novichok candidates

**Figure 3:**
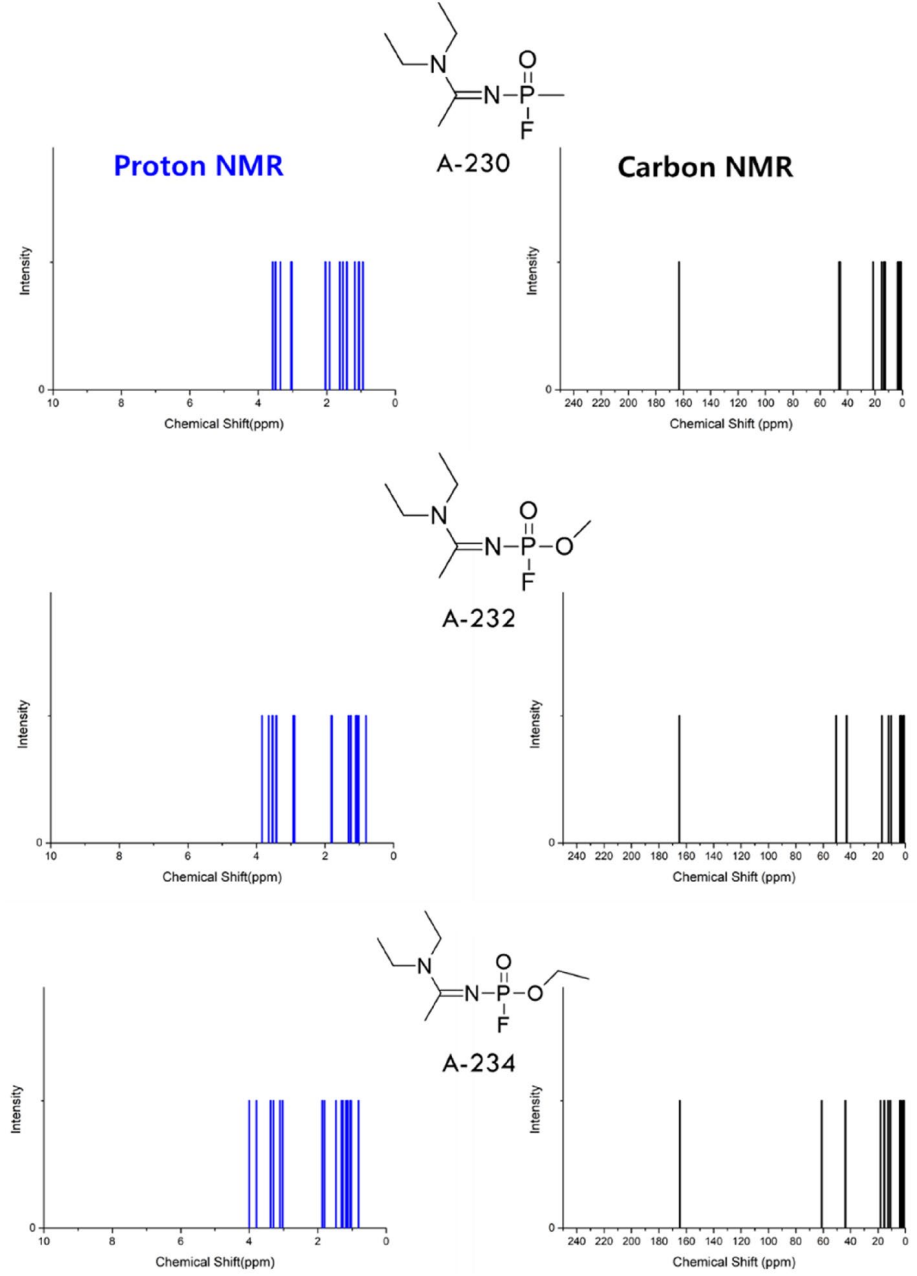
The structure of each Novichok candidate and its predicted ^1^H and ^13^C NMR spectra. Chloroform was estimated for experimental sampling; therefore, the M06-2X/6-311 + G(2d,p) and MPW1PW91/6-311 + G(2d,p) functionals/basis set combinations were used for the DFT study.

As the accuracy of the proposed method for the prediction of the ^1^H and ^13^C NMR spectra was clearly established in the case of both OP compounds and nerve agents, as the next step, the NMR spectra of novel Novichok candidates were predicted. The chemical structures of the Novichok candidates (A-230, A-232, and A-234) were obtained from previous studies and are depicted in Fig. 3^2,20–23^. Their conformational isomers—10, 9, and 24 structures, respectively, were accounted for. All calculations in CDCl_3_ were performed separately and averaged using the Boltzmann distribution, and each chemical shift was predicted as demonstrated in our previous study on the chemical shift prediction of the organophosphorus materials and nerve agents. All the predicted chemical shifts (^1^H and ^13^C) are presented in the Supporting Information.

As apparent from the predicted spectrum in Fig. 3, the ^13^C NMR chemical shift was distinct for each structure, and its prediction was exceptionally accurate (deviation of less than 1–2 ppm) in CDCl_3_. Its chemical shift window was significantly larger than that of proton NMR, establishing its suitability for the detection or characterization of Novichok candidates.

### Generating big data on the possible Novichok candidates

**Figure 4:**
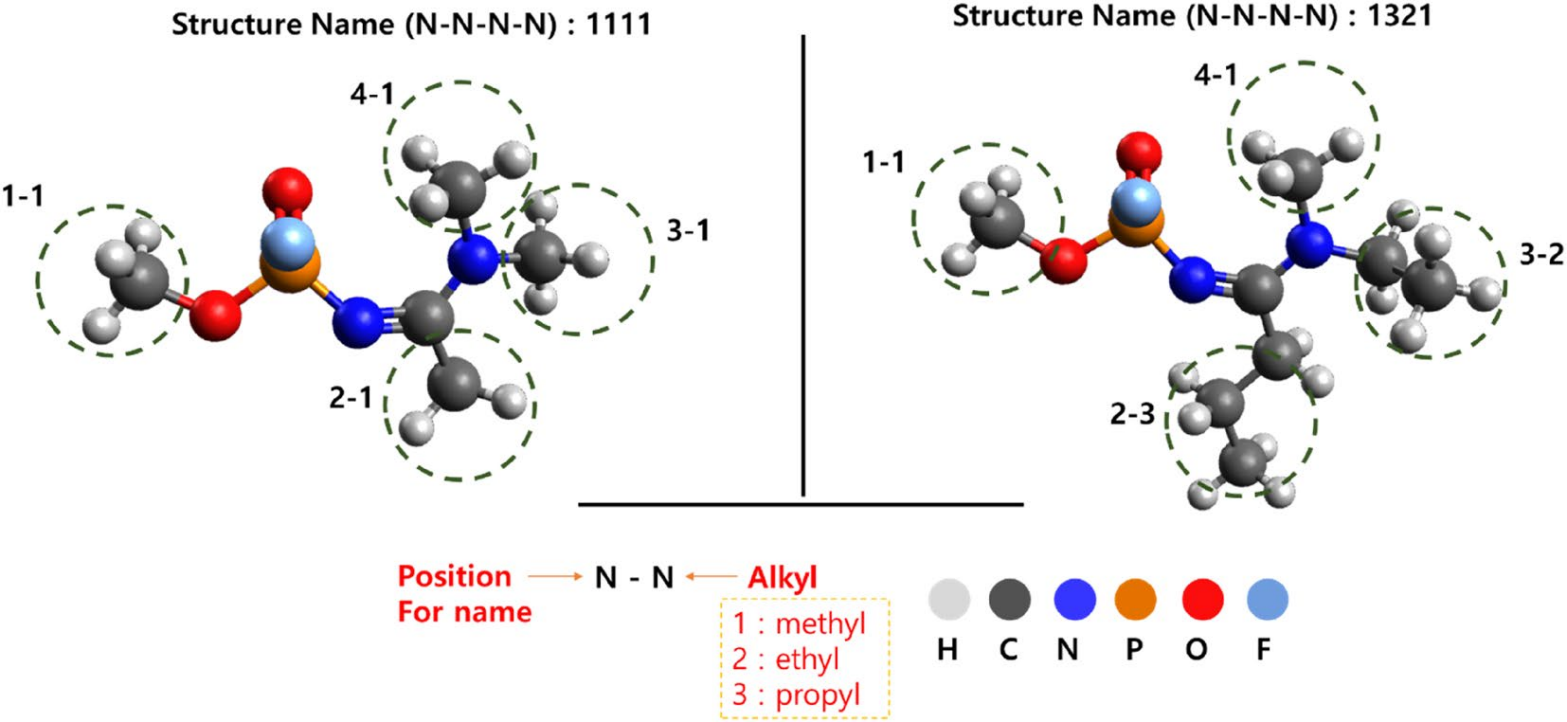
Method for name assignment of A-series Novichok candidates when the functional group is substituted with three alkyl groups (methyl, ethyl, and propyl). Chloroform was estimated for experimental sampling; therefore, the M06-2X/6-311 + G(2d,p) and MPW1PW91/6-311 + G(2d,p) functionals/basis set combinations were used for the DFT study.

In this stage, to build a database, the spectra of about 83 extra substances were calculated to obtain the big data of NMR spectra for additional Novichok candidates. Among the 83 substances, A-242 and A-262 were included, which were already categorized as Novichok candidates, as well as 81 additional structures in which each functional group was substituted with three types of alkyl branches (methyl, ethyl, and propyl) utilizing the A-series candidates of Novichok as the backbone structure. The functional group was changed along 1–1, 2–1, 3–1, and 4–1 because of the suggested structure, which is denoted as 1A13 and 1A14 in the CWC list update (Supplementary Fig. S15)^24,25^. The naming strategy for the structures substituted with the alkyl groups is shown in Fig. 4. For instance, 1111C indicated methyl–methyl–methyl–methyl groups on each series of the functional groups on the scaffold, and the 13C NMR is predicted (1st molecular structure on SI_Novichok_NMR_Figure); 3122H indicates propyl–methyl–ethyl–ethyl groups on each series of the functional groups on the scaffold, and the 1H NMR is predicted (142nd molecular structure on SI_Novichok_NMR_Figure).

All ^1^H and ^13^C predicted NMR data from the structures, as shown in Fig. 4, are given in the Supporting Information. These results provide a database for future Novichok candidate-related research as well as identification.

### Experimental and computational results for A-232 and A-234 in DMSO

**Figure 5:**
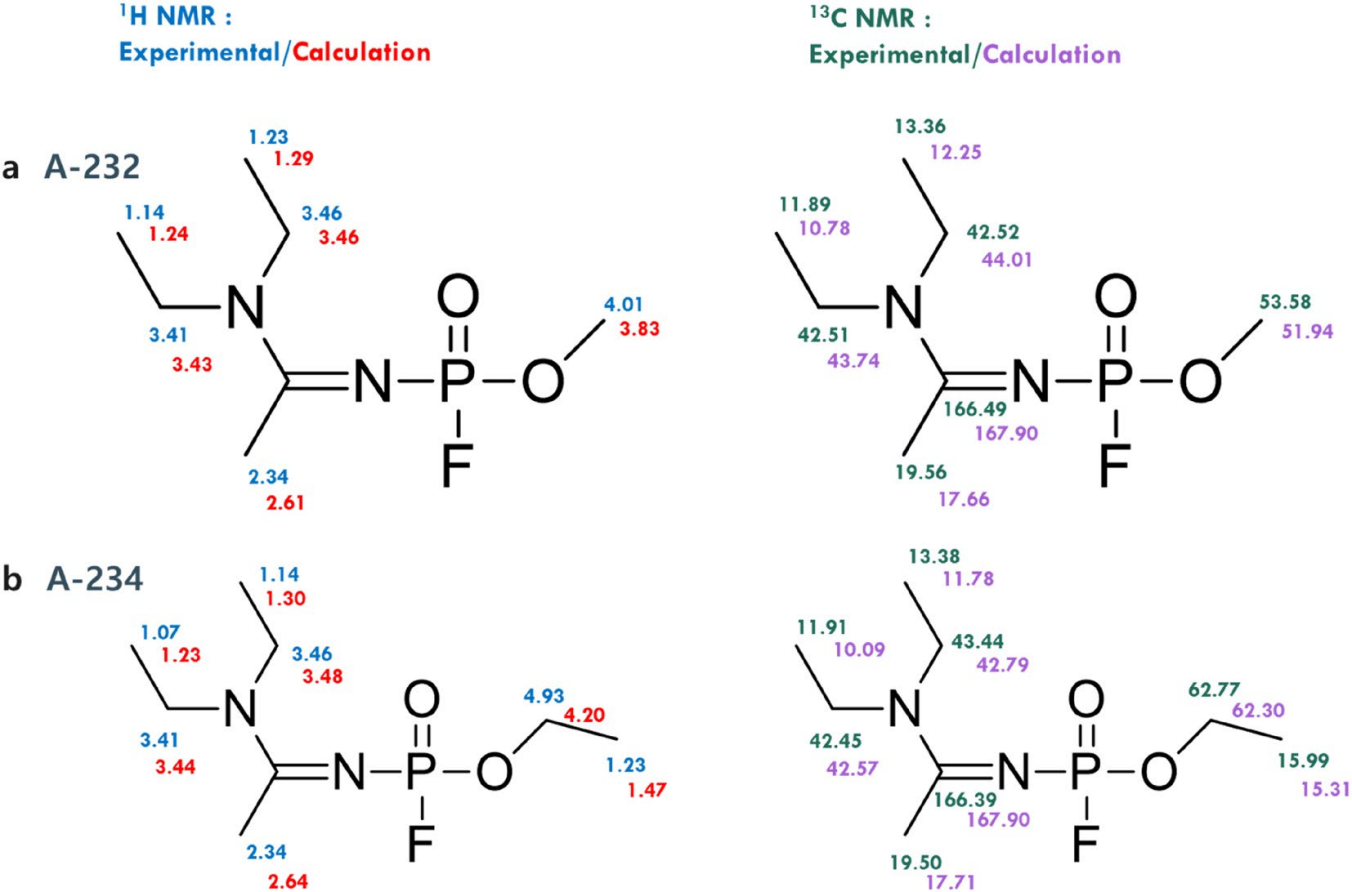
Comparison between experimental and predicted data for ^1^H and ^13^C NMR chemical shift of A-232 (**a**) and A-234 (**b**), which was used in the UK, in DMSO. DMSO was used for experimental sampling; therefore, the B3LYP/6-31 + G(d,p) and PBE0/6-311 + G(2d,p) functionals/basis set combinations were used for the DFT study.

A-232 (CAS number 2387496-04-8) and A-234 (CAS number 2387496-06-0) were microsynthesized by the Agency for Defense Development (ADD) in South Korea^6^. A-234 is the Novichok agent that was used in the UK. Post-synthesis characterization was conducted based on ^1^H and ^13^C NMR with considerable care. However, unlike the NMR experiment data and procedure of the other nerve agents, owing to its safety concerns and experimental setup DMSO-d was used as the NMR solvent instead of CDCl_3_, thereby requiring a different computational method (see the computational method for DMSO and Supporting Information for the experimentally obtained spectrum)^26^. The calculation method detailed in previous sections, supplemented by conformational considerations (20 and 24 different isomers, respectively), was used to compare the experimental and predicted data. Different scaling factors with different SMD intrinsic solvent models were applied, and the comparisons are shown in Fig. 5.

As shown in Fig. 5, DMSO-based predictions also exhibited exceptional accuracy (average MAE of less than 0.3 ppm for ^1^H and 2 ppm for ^13^C). This establishes the effectiveness of DFT calculations, supplemented with conformation isomer considerations, in the accurate prediction of NMR chemical shifts.

## Conclusions

In this study, we successfully predicted the ^1^H and ^13^C NMR chemical shifts of Novichok candidates for the first time. The accuracy of the proposed method was evaluated by comparing the DFT-based values with experimental data of the chemical shifts of four OP compounds and data corresponding to G/V-series nerve agents obtained from the OPCW database. The accuracy of the method was observed to be more than 95% and its MAE was less than 0.2 ppm for ^1^H and 2 ppm for ^13^C. Moreover, this prediction performance was further improved once conformational isomers were considered. This indicates that, even though additional computation is necessary to consider conformational isomers, investigation of the Boltzmann distribution is essential to improve prediction accuracy, and hence the increased computational load is a worthwhile trade-off for the increase in accuracy. This is especially true for Novichok candidates, which share very similar structures. Further, the ^13^C NMR chemical shift prediction was observed to be significantly more accurate in comparison to the ^1^H NMR prediction, indicating its higher suitability for application in future studies. The sequential DFT calculations were performed in CDCl_3_, which is the most commonly used NMR solvent. Further, predictive ^1^H and ^13^C NMR spectra of additional 83 Novichok candidates were calculated and presented as a database for future applications. The additional experimental ^1^H and ^13^C NMR spectra of the A-232 and A-234 (which were used in the UK in 2018) utilized DMSO-d instead of chloroform. To verify the effectiveness of the proposed method, an additional DMSO-based DFT calculation was carried out to successfully predict the ^1^H and ^13^C NMR chemical shifts of A-232 and A-234. Consideration of conformational isomers was observed to enable the successful identification of Novichok agents. We expect that this series of studies will enable the creation of voluminous ^1^H and ^13^C NMR spectral databases via the accurate prediction of various new types of nerve agents. Further, the proposed prediction method is expected to enable the swift identification of new types of nerve agents in the future.

## Supplementary Information

Supplementary Information 1.

Supplementary Information 2.

Supplementary Information 3.

## Data Availability

The MOST dataset generated during this study is included in this published article and supplementary information file. Any other datasets generated during and/or analyzed during the current study are available from the corresponding author (K. J.) upon reasonable request.
