# Supplementary material for: Precisely predicting the 1H and 13C NMR chemical shifts in new types of nerve agents and building spectra database
Source: Sci Rep. 2022 Nov 24;12:20288. doi: 10.1038/s41598-022-24647-y (PMC9700684; doi:10.1038/s41598-022-24647-y)
Supplement: Supplementary file 2 — Supplementary Information 2. [file 41598_2022_24647_MOESM2_ESM.docx]

Supporting Information 1

Precisely predicting the ^1^H and ^13^C NMR Chemical Shifts in New Types of Nerve Agents and building spectra database

*Keunhong Jeong^a*^**, Tae In Ryu^b^, Seung-Ryul Hwang^b^, Yoonjae Cho^b^, Kyoung Chan Lim^c^, Ung Hwi Yoon^a^, Jin-Young Lee^c^, Young Wook Yoon^b^, Hey Jin Jeong^a^*

Experimental and calculation results for four OP compounds

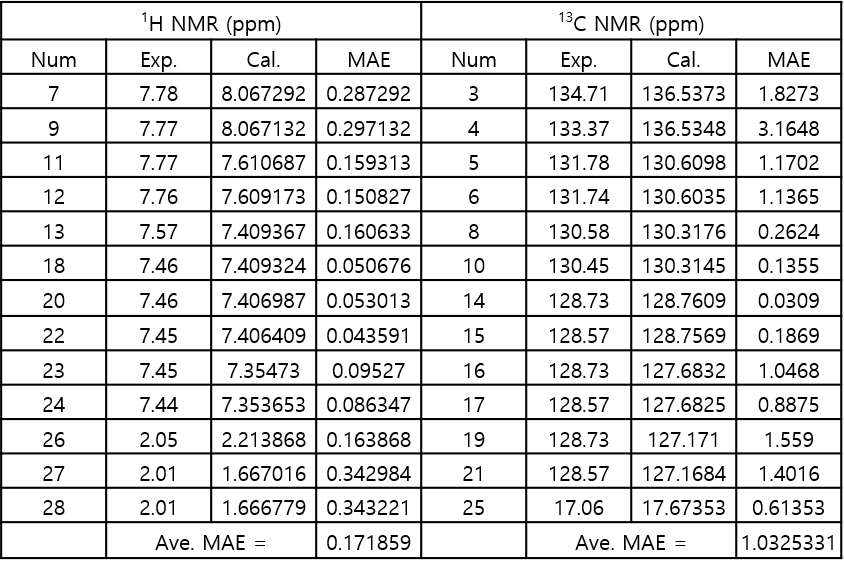


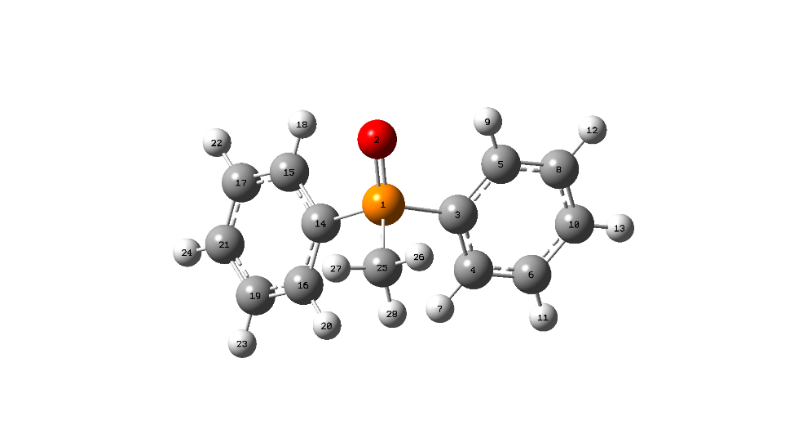


Figure S1. Methyldiphenylphosphine oxide structure (upper figure), its chemical shift with each atomic index (lower table and figure)


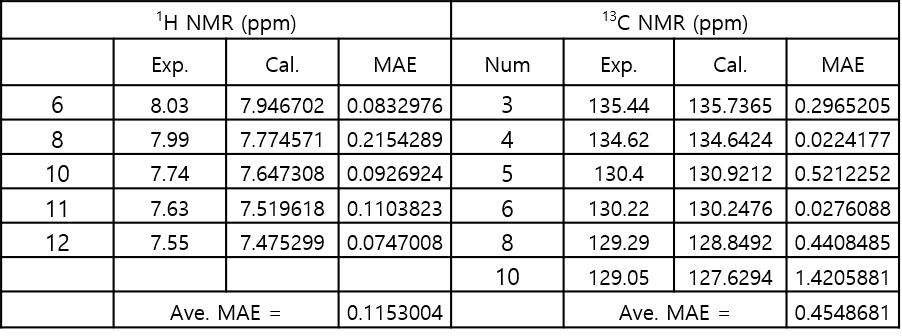


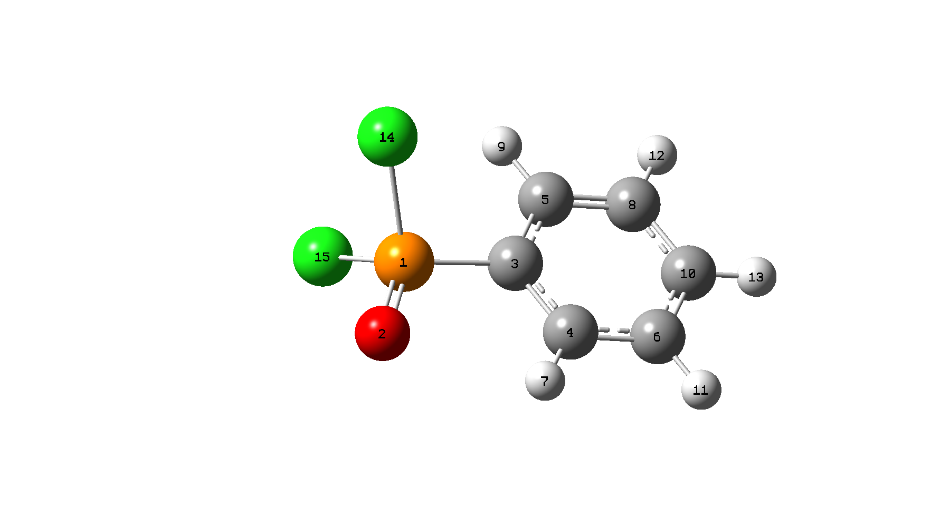


Figure S2. Dichlorophenylphosphine oxidestructure (upper figure), its chemical shift with each atomic index (lower table and figure)

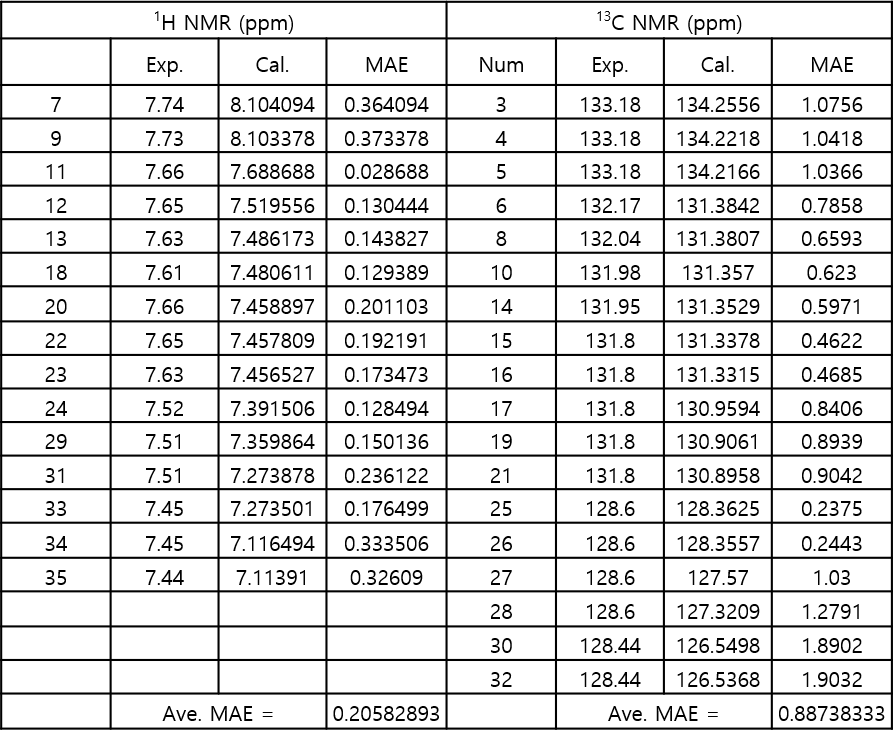


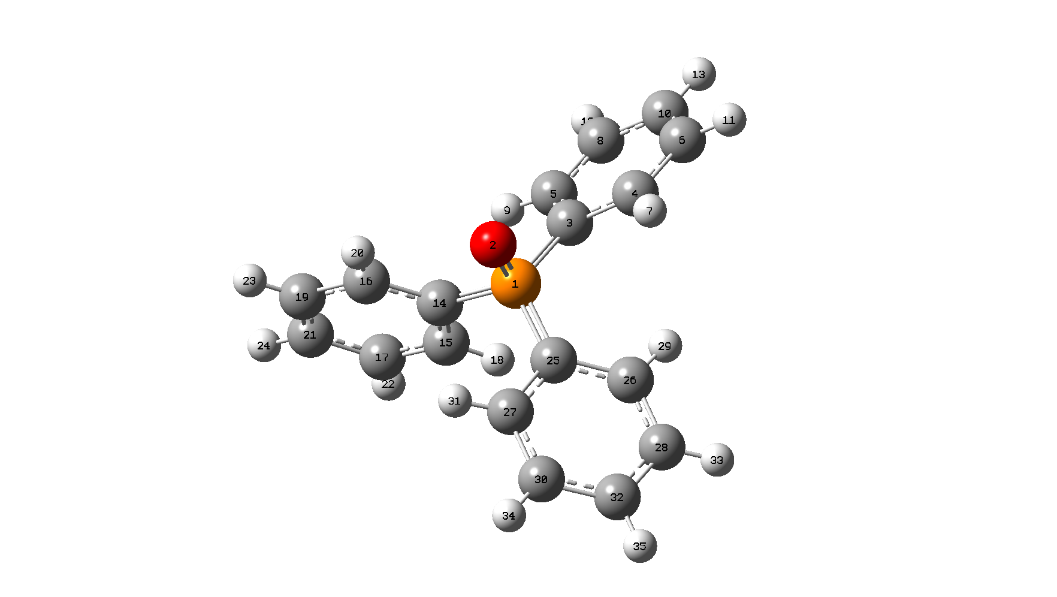


Figure S3. Triphenylphosphine oxidestructure (upper figure), its chemical shift with each atomic index (lower table and figure)

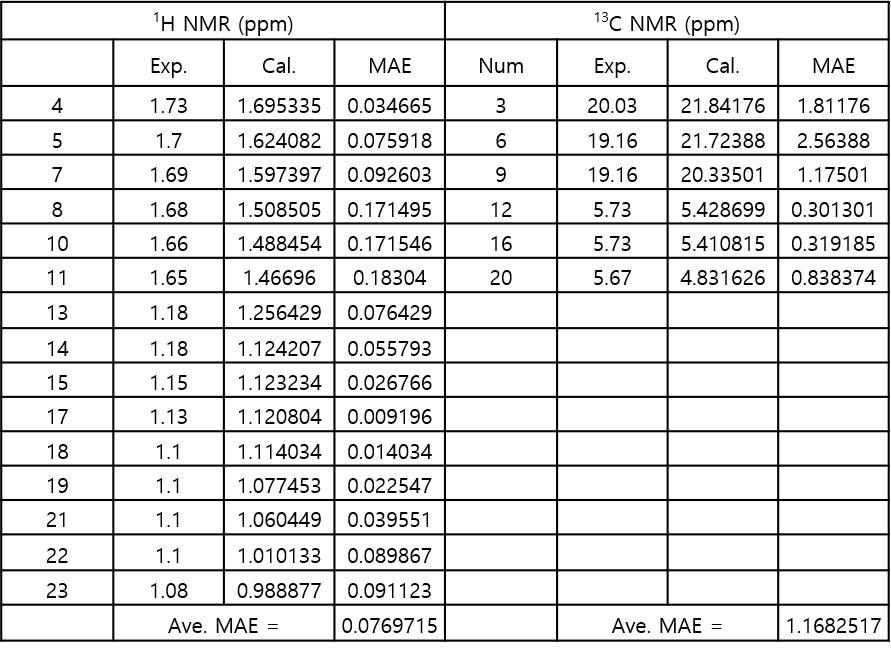


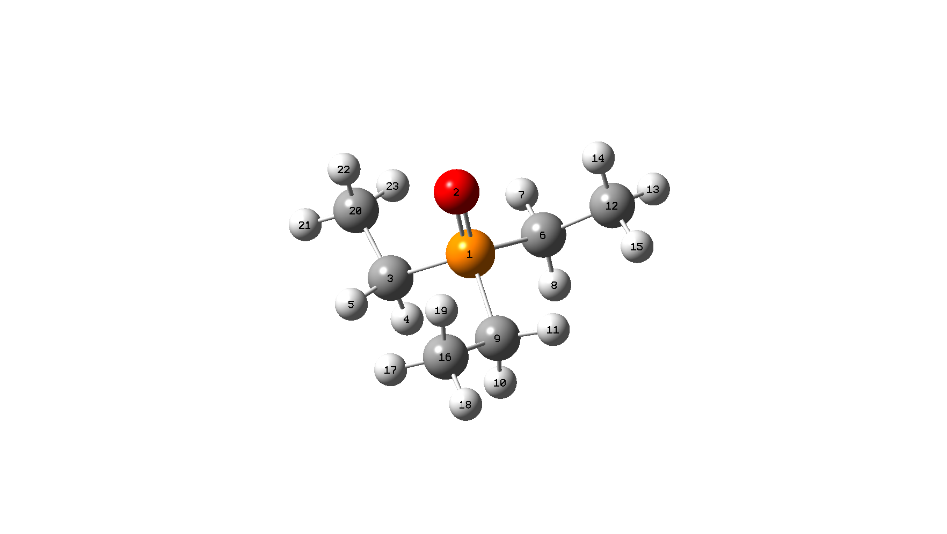


Figure S4. Triethylphosphine oxidestructure (upper figure), its chemical shift with each atomic index (lower table and figure)

Experimental and calculation results for five nerve agent compounds

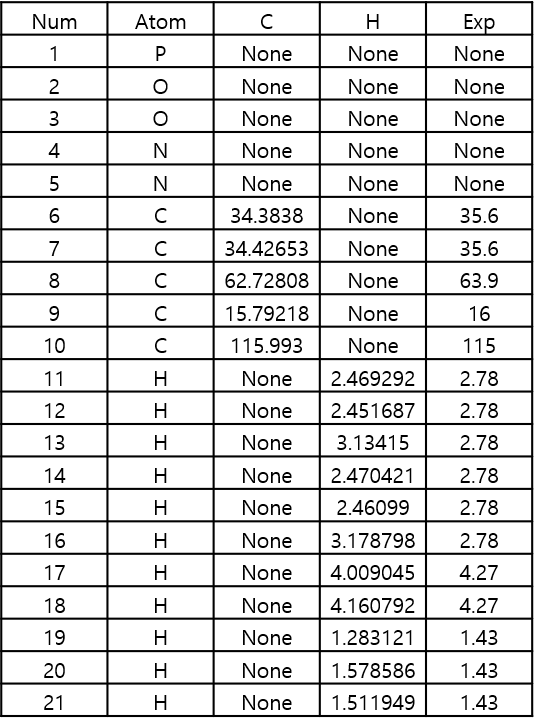


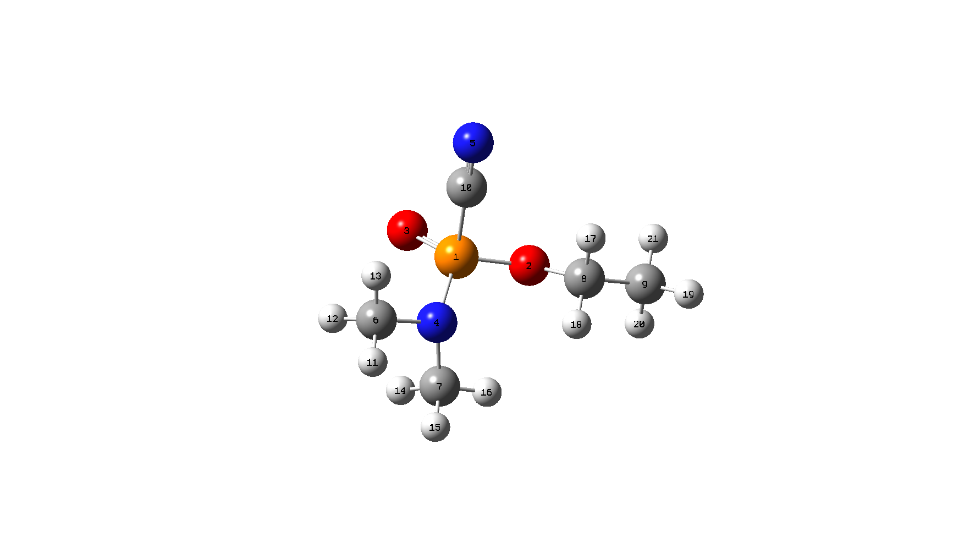


Figure S5. Tabun structure (upper figure), its chemical shift with each atomic index (lower table and figure)

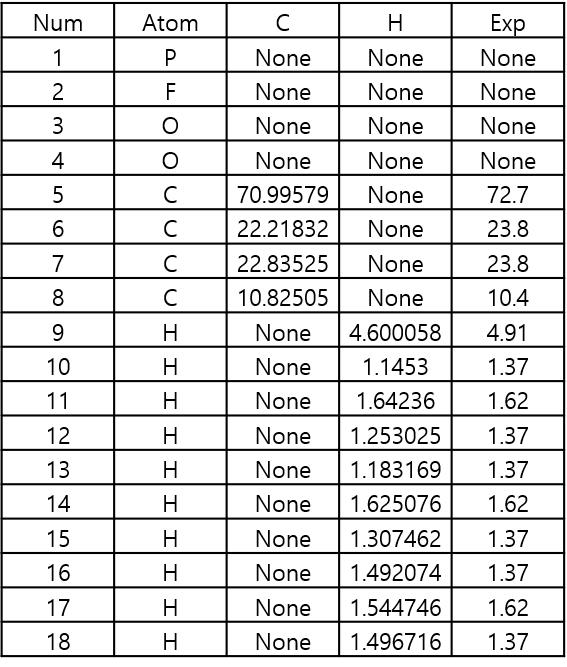


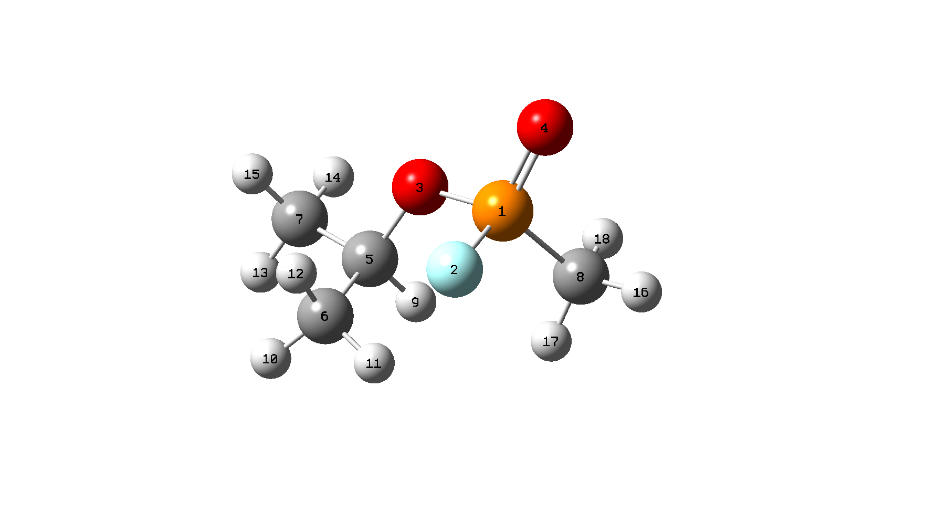


Figure S6. Sarin structure (upper figure), its chemical shift with each atomic index (lower table and figure)


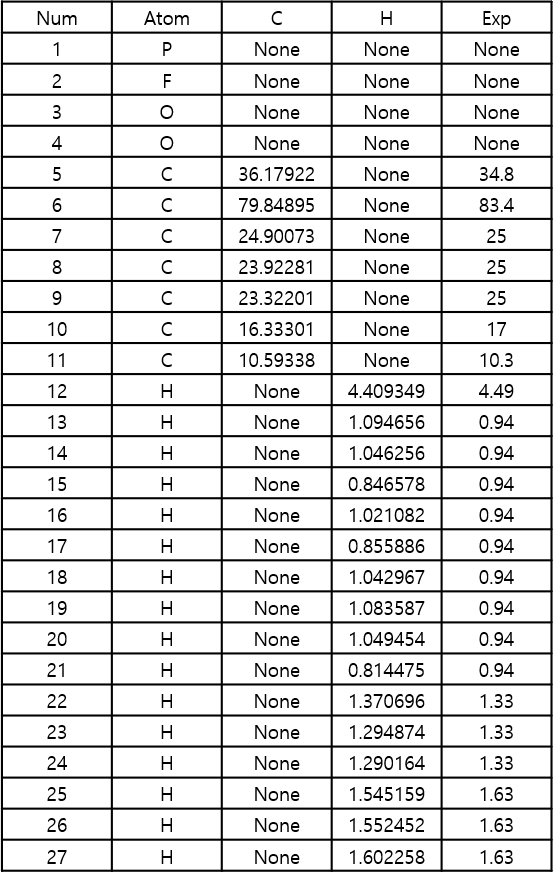

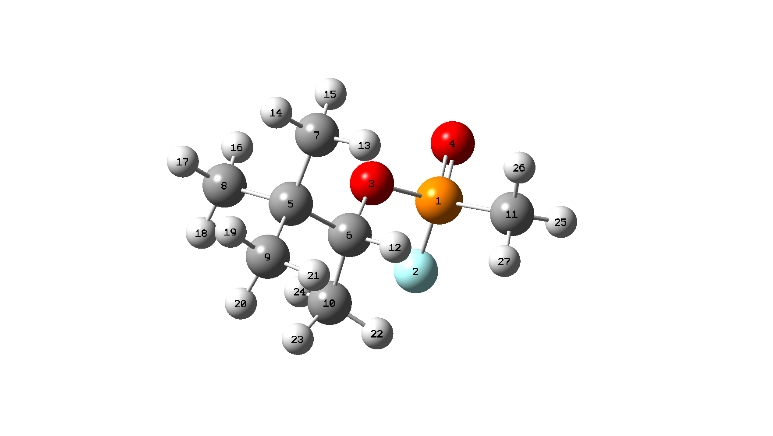


Figure S7. Soman structure (upper figure), its chemical shift with each atomic index (lower table and figure)

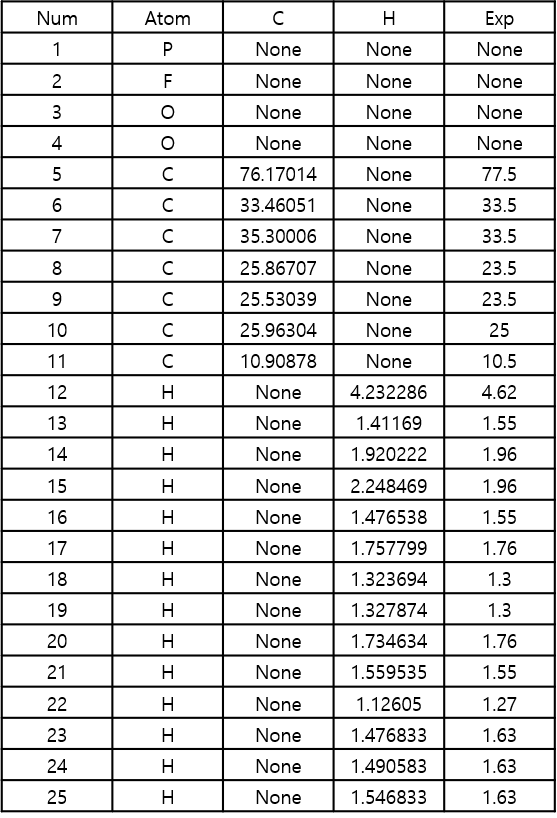


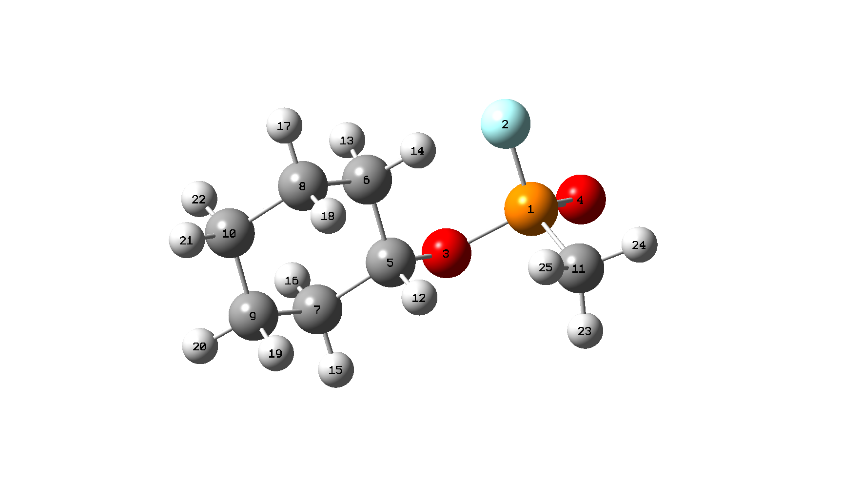


Figure S8. Cyclosarin structure (upper figure), its chemical shift with each atomic index (lower table and figure)


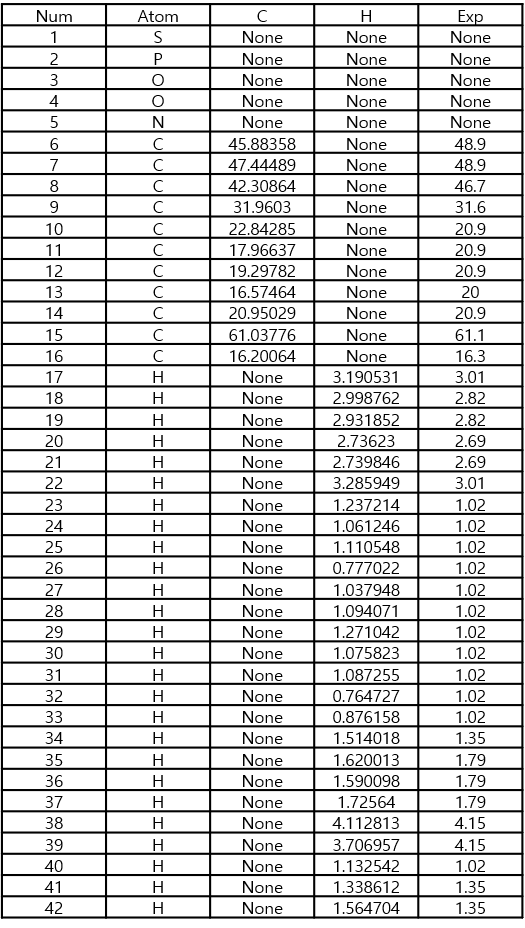

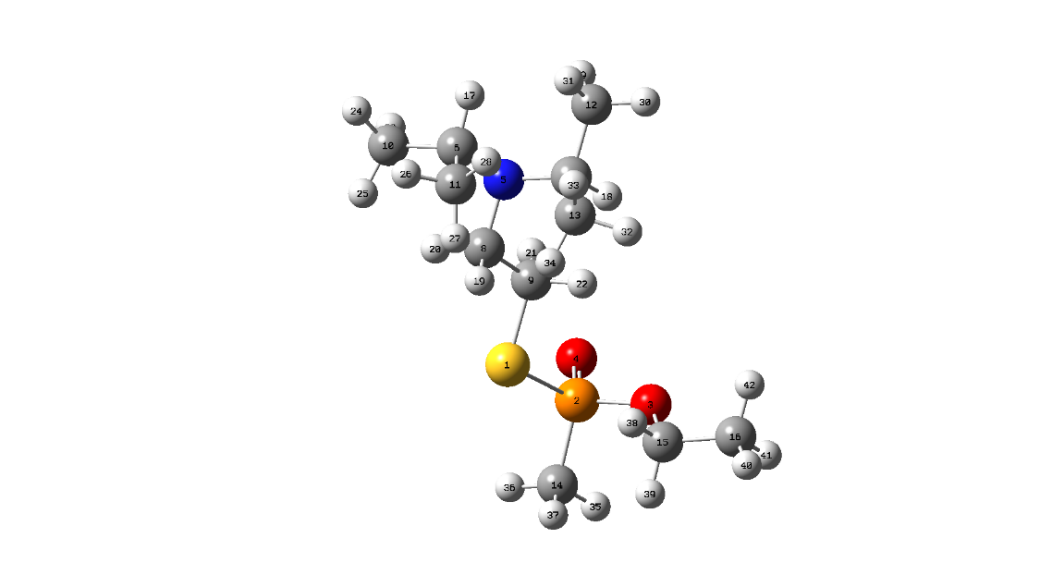
Figure S9. VX structure (upper figure), its chemical shift with each atomic index (lower table and figure)


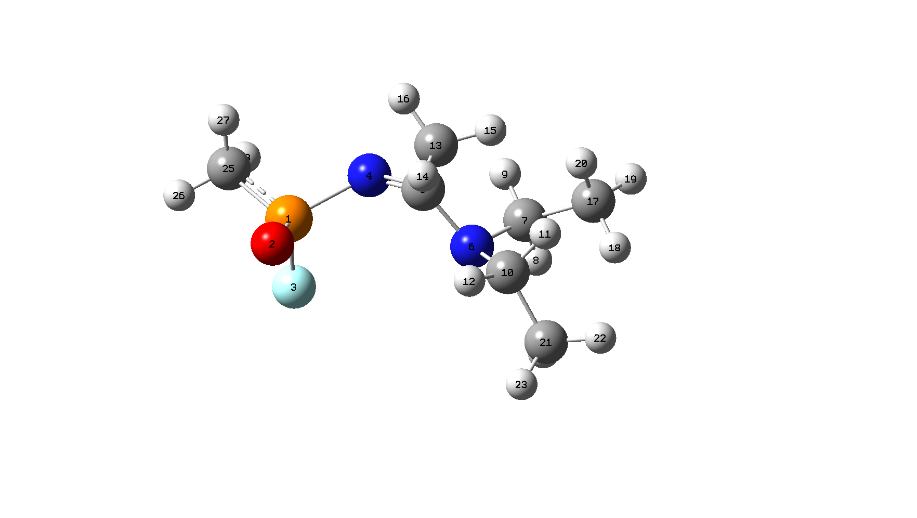

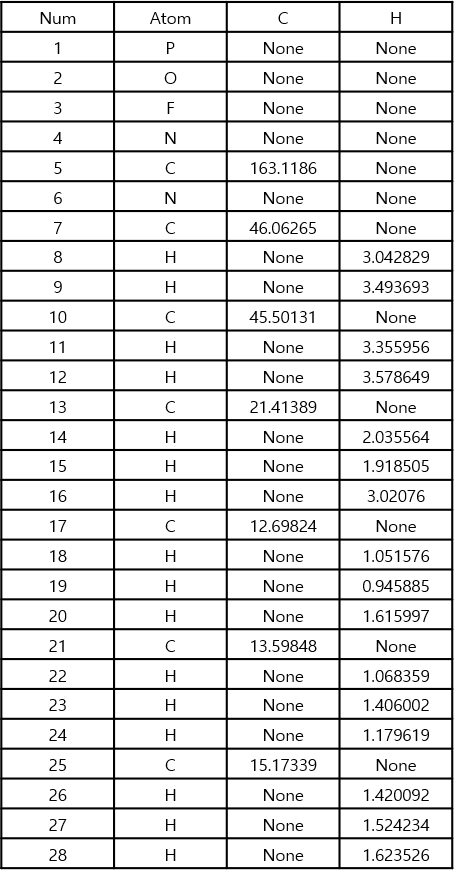
Experimental and calculation results for three Novichok candidates

Figure S10. A-230 structure (upper figure), its chemical shift with each atomic index (lower table and figure)


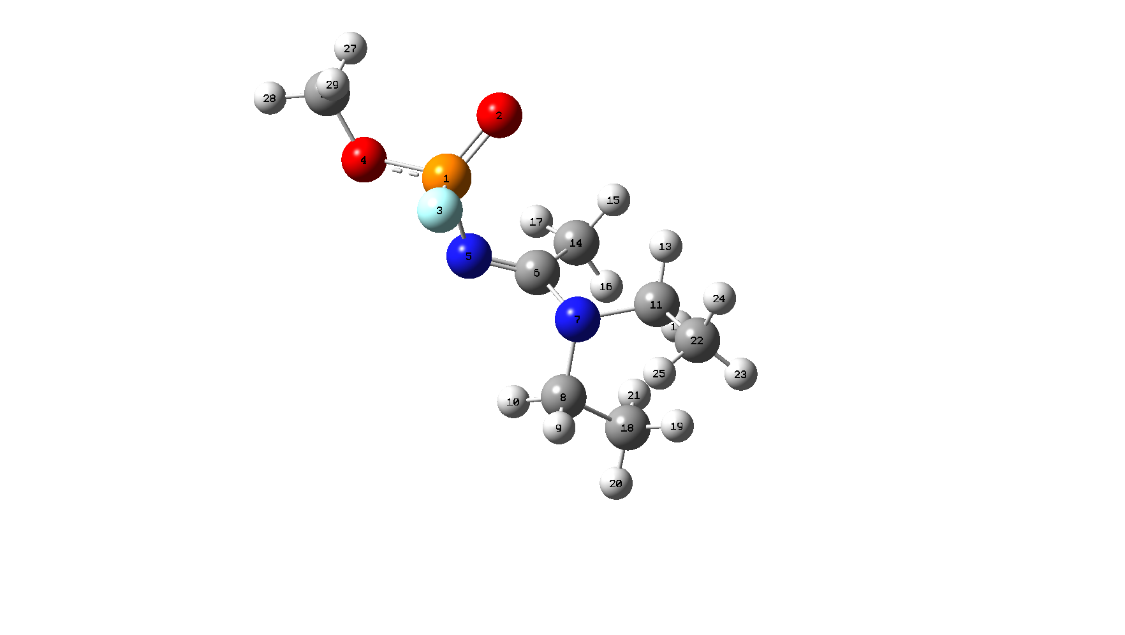

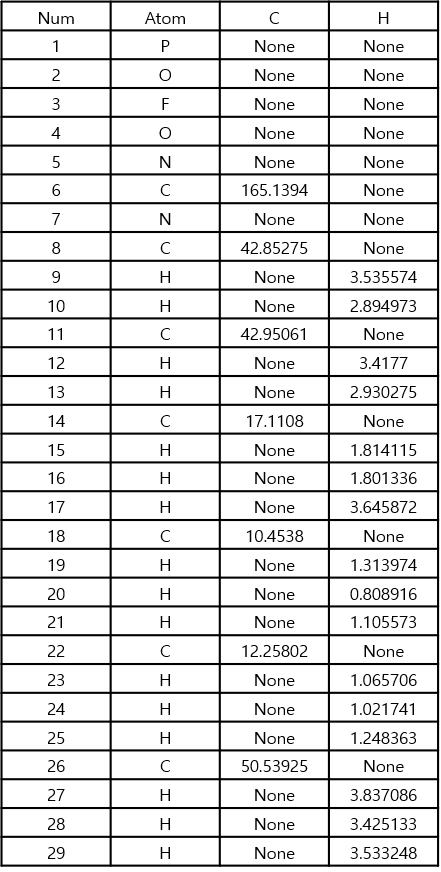
Figure S11. A-232 structure (upper figure), its chemical shift with each atomic index (lower table and figure)


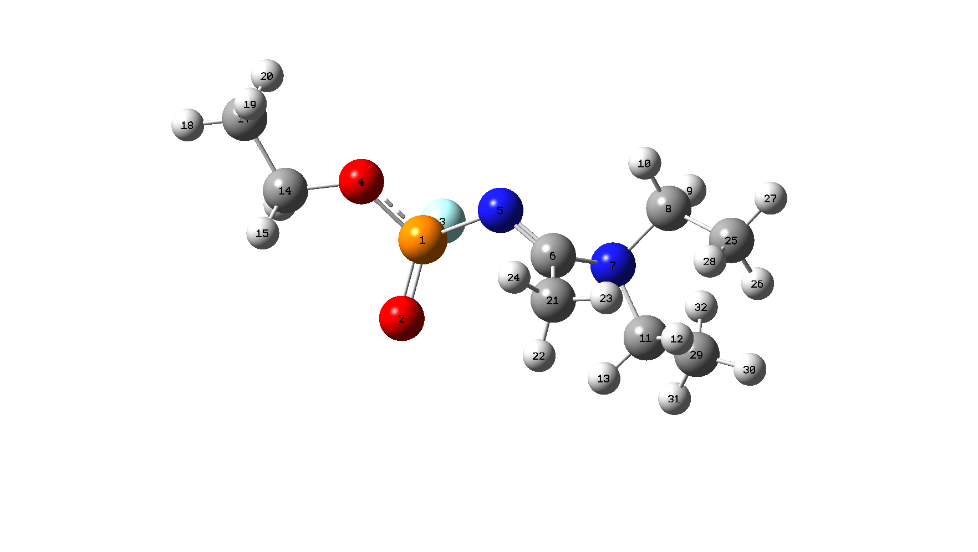

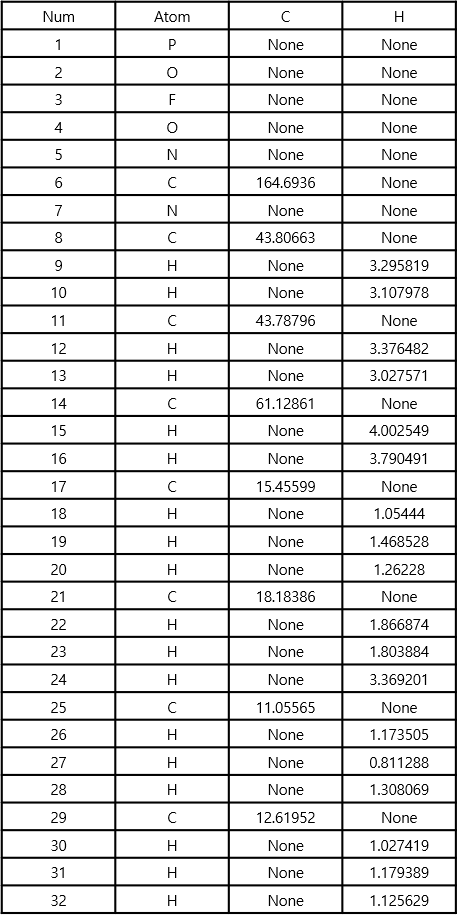
Figure S12. A-234 structure (upper figure), its chemical shift with each atomic index (lower table and figure

^1^H and ^13^C NMR spectra of A-234

Figure S13. ^13^C NMR spectrum of A-234 structure in DMSO solvent

Figure S14. ^1^H NMR spectrum of A-234 structure in DMSO solvent


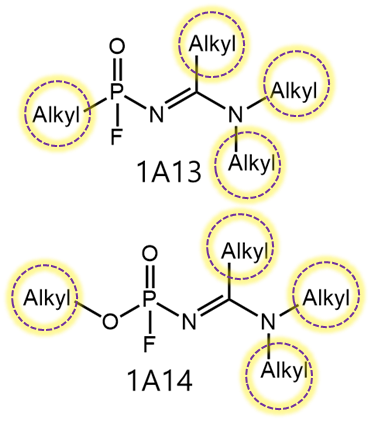


Figure S15. CWC updated list with alkyl < 10 carbons including cycloalkane


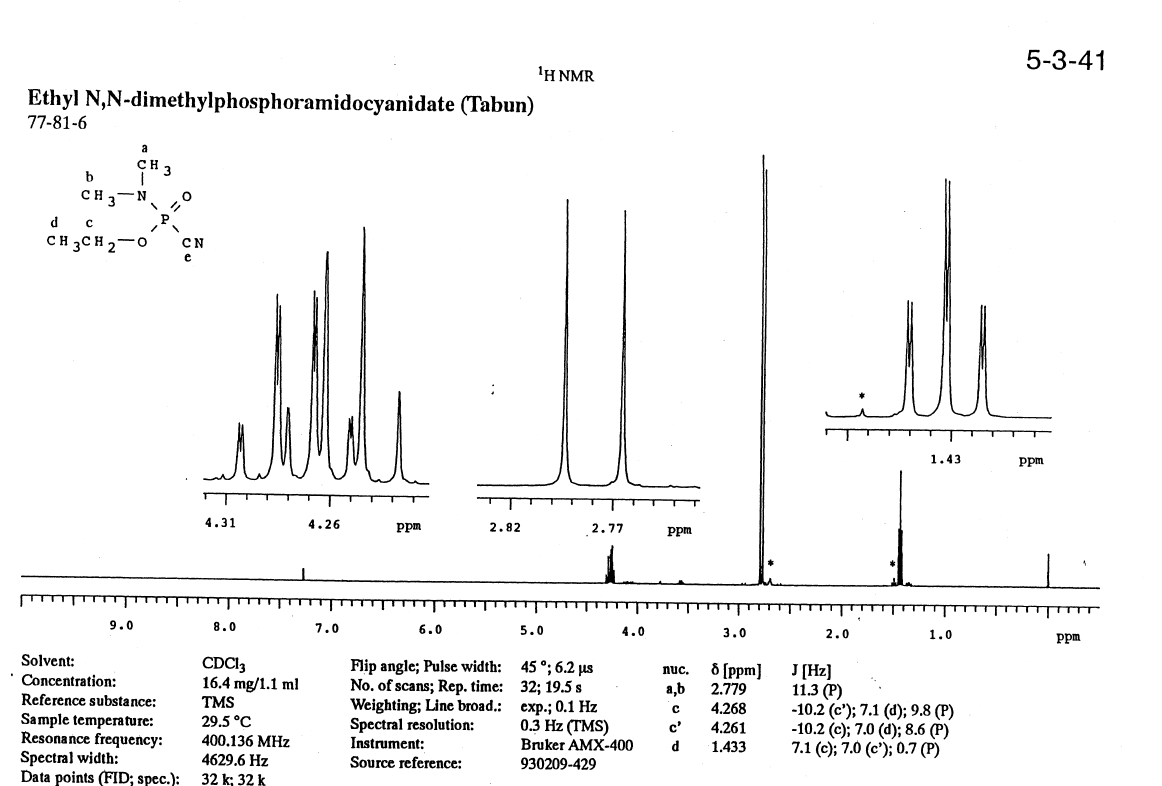


Figure S16. ^1^H NMR spectrum of Tabun.


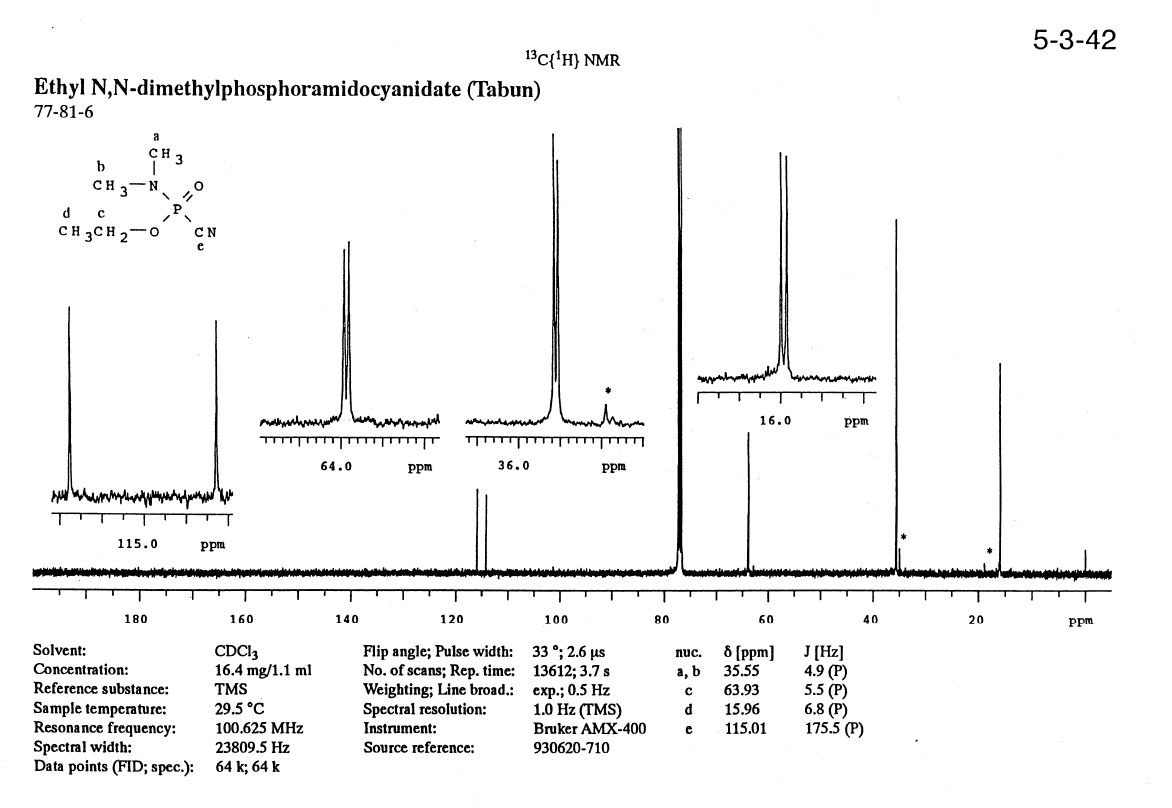


Figure S17. ^13^C NMR spectrum of Tabun.


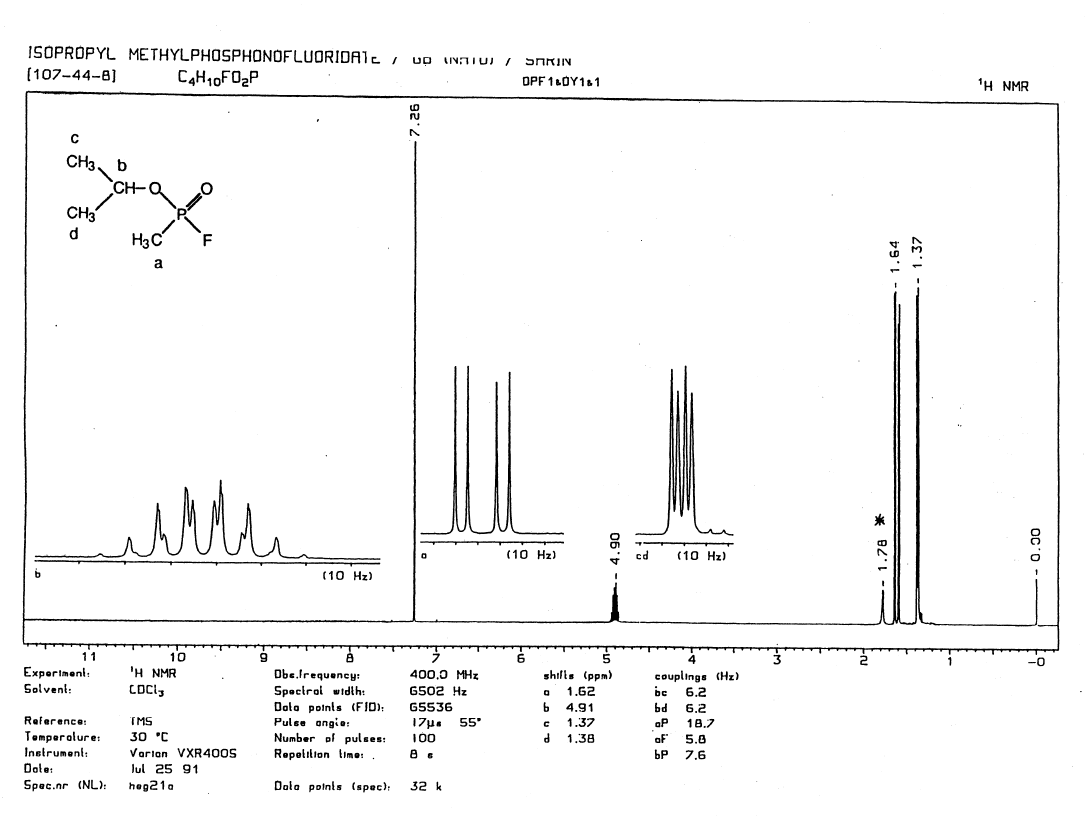


Figure S18. ^1^H NMR spectrum of Sarin.


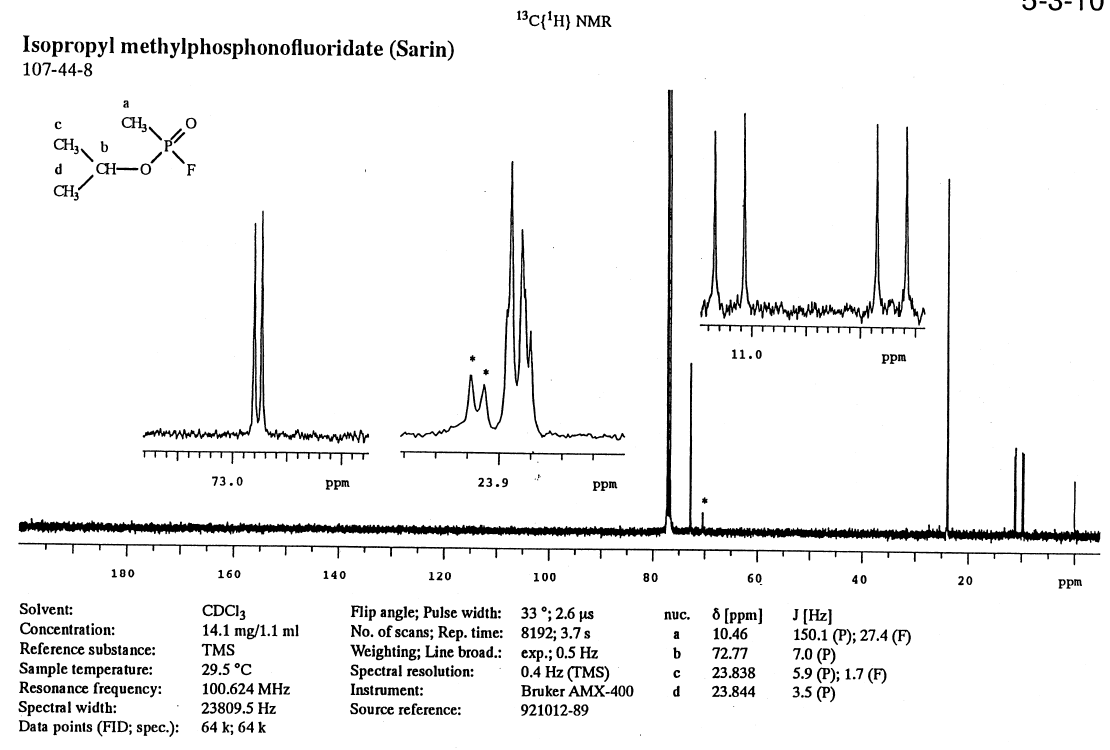


Figure S19. ^13^C NMR spectrum of Sarin.


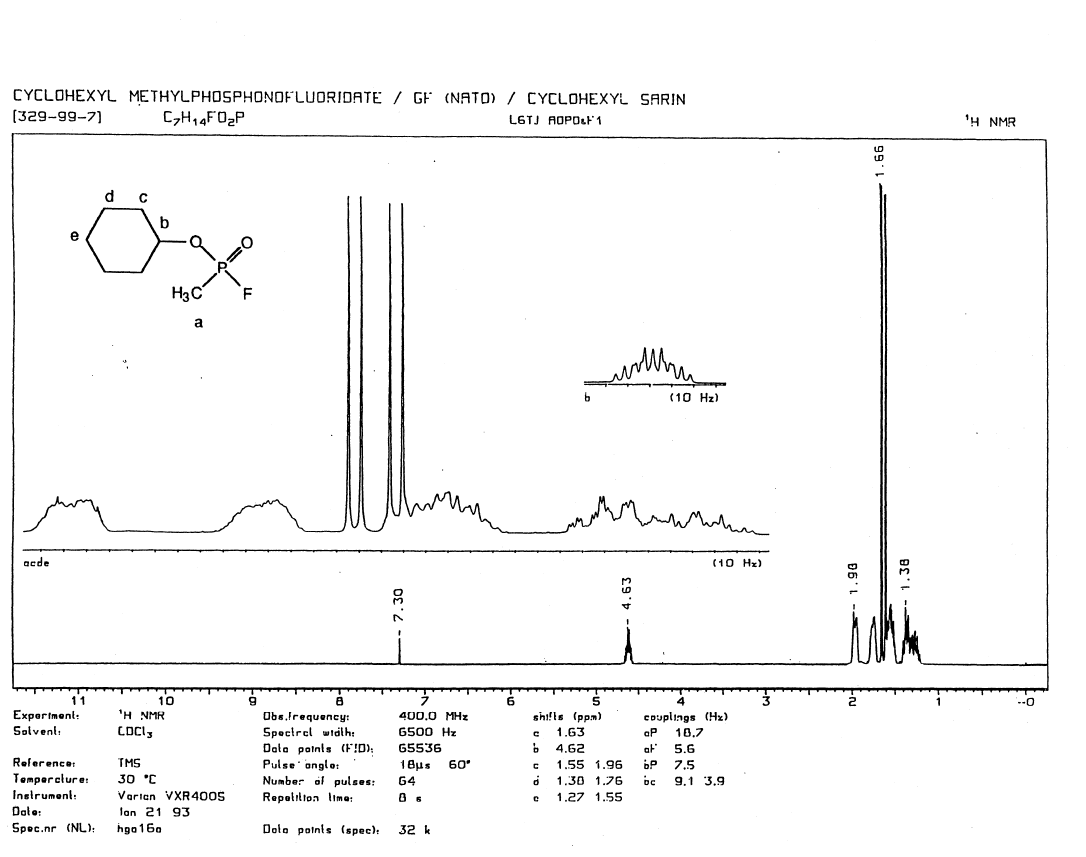


Figure S20. ^1^H NMR spectrum of Cyclosarin.


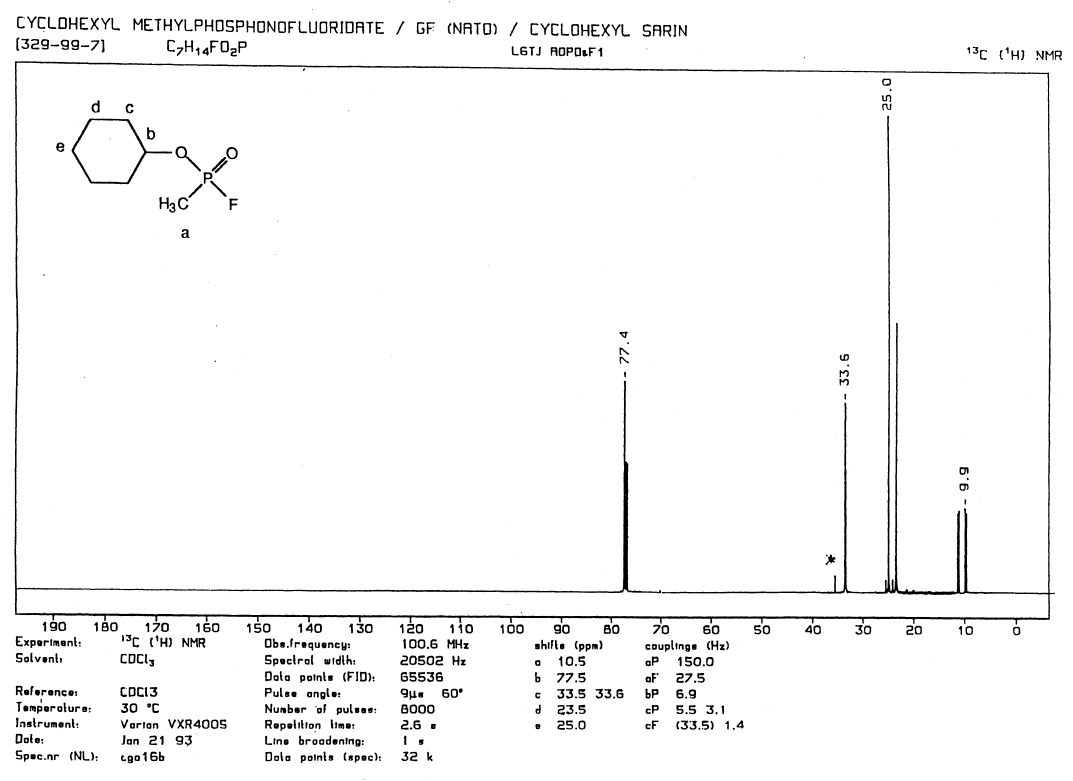


Figure S21. ^13^C NMR spectrum of Cyclosarin.


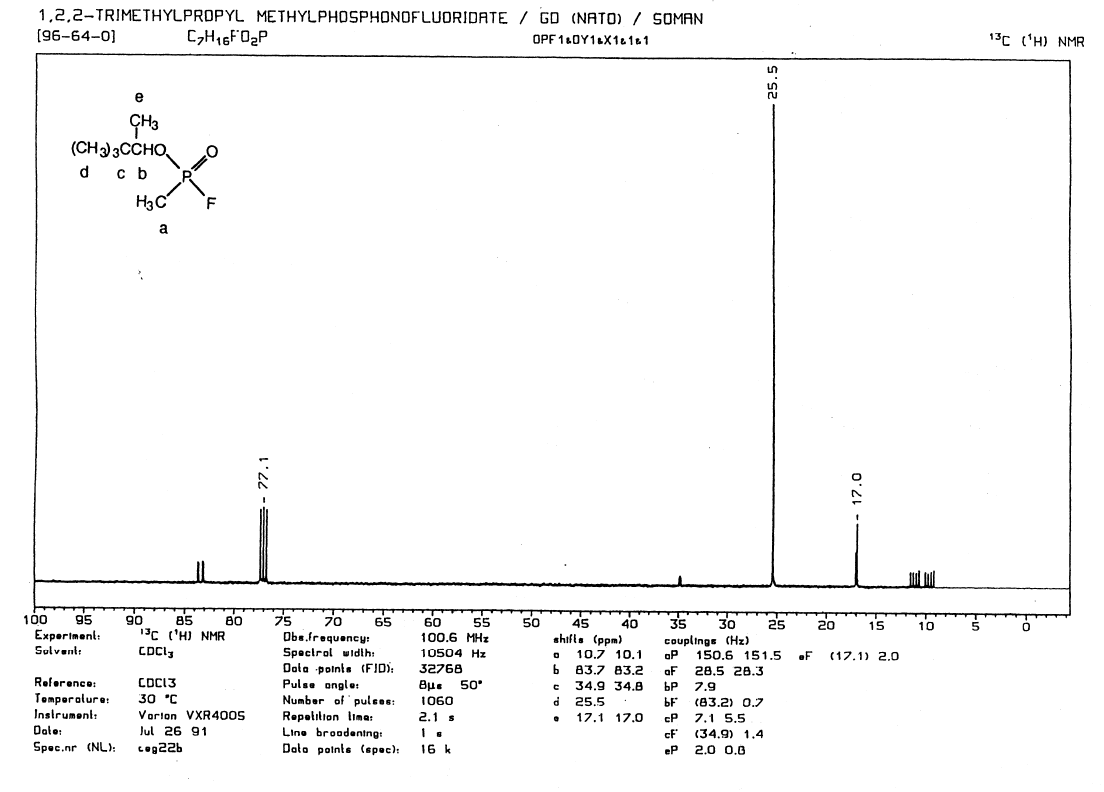


Figure S22. ^13^C NMR spectrum of Soman.


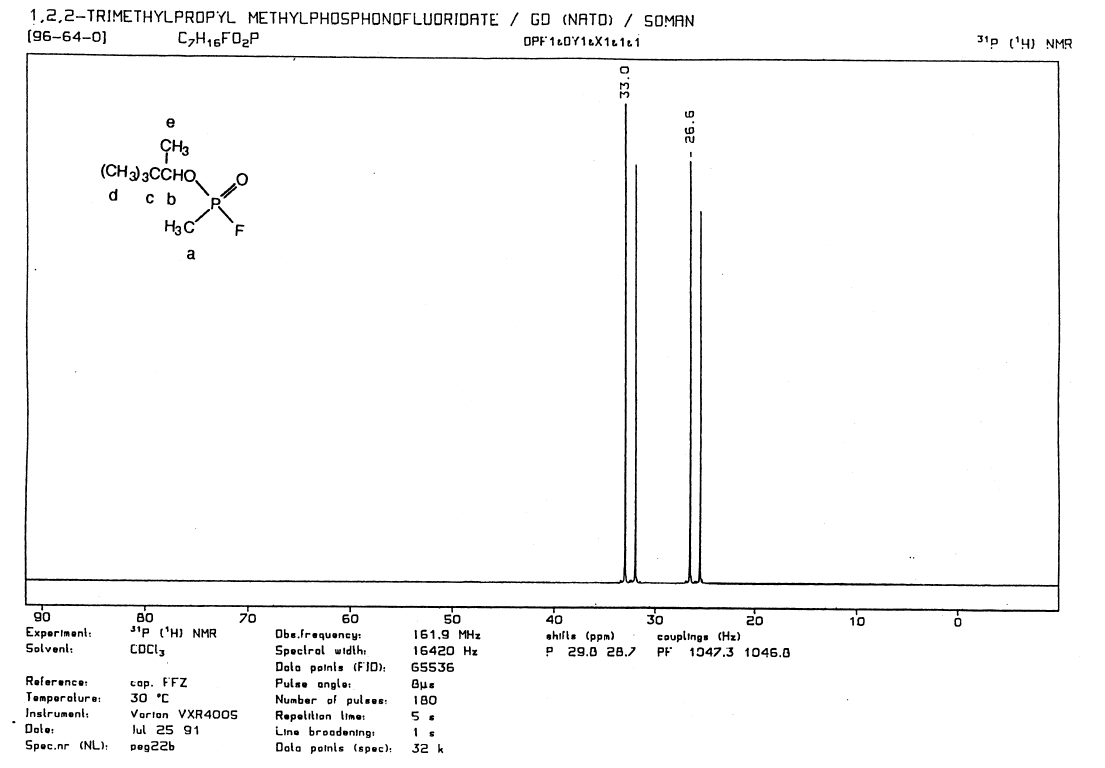


Figure S23. ^1^H NMR spectrum of Soman.


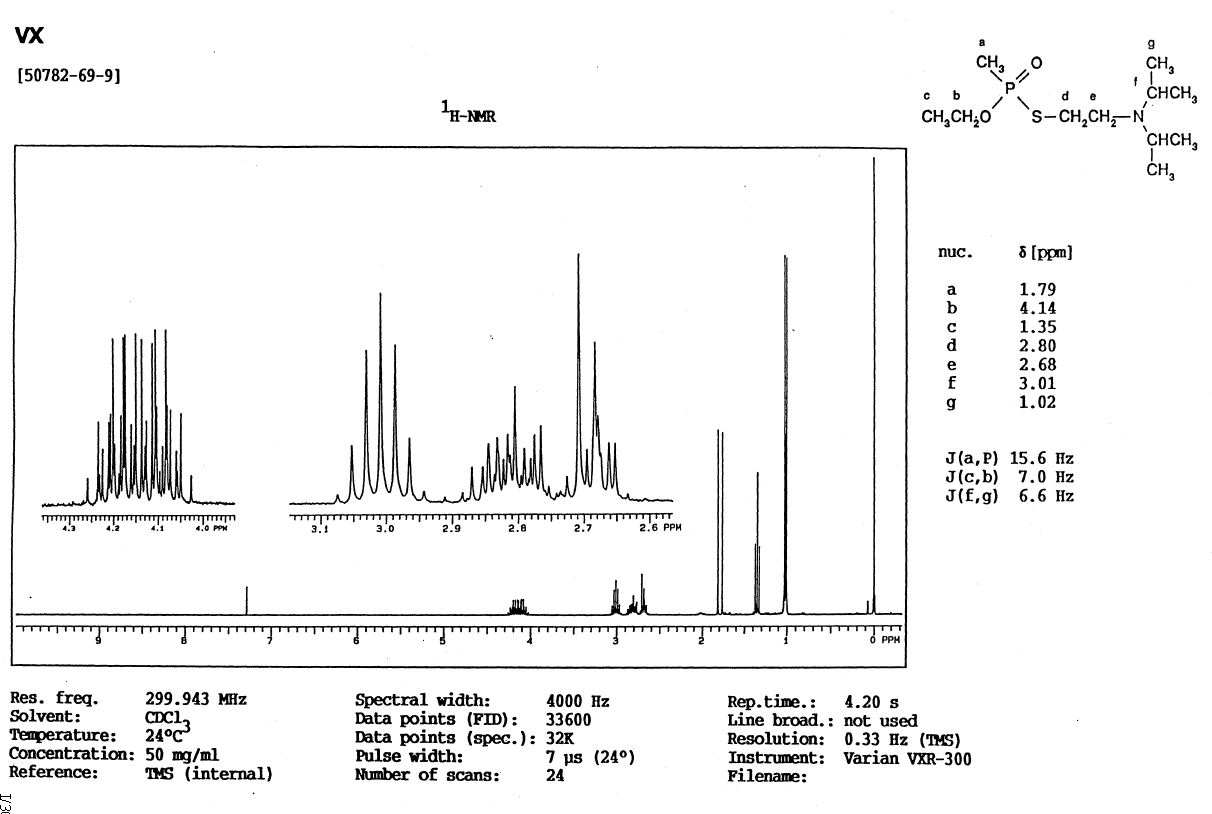


Figure S24. ^1^H NMR spectrum of VX.


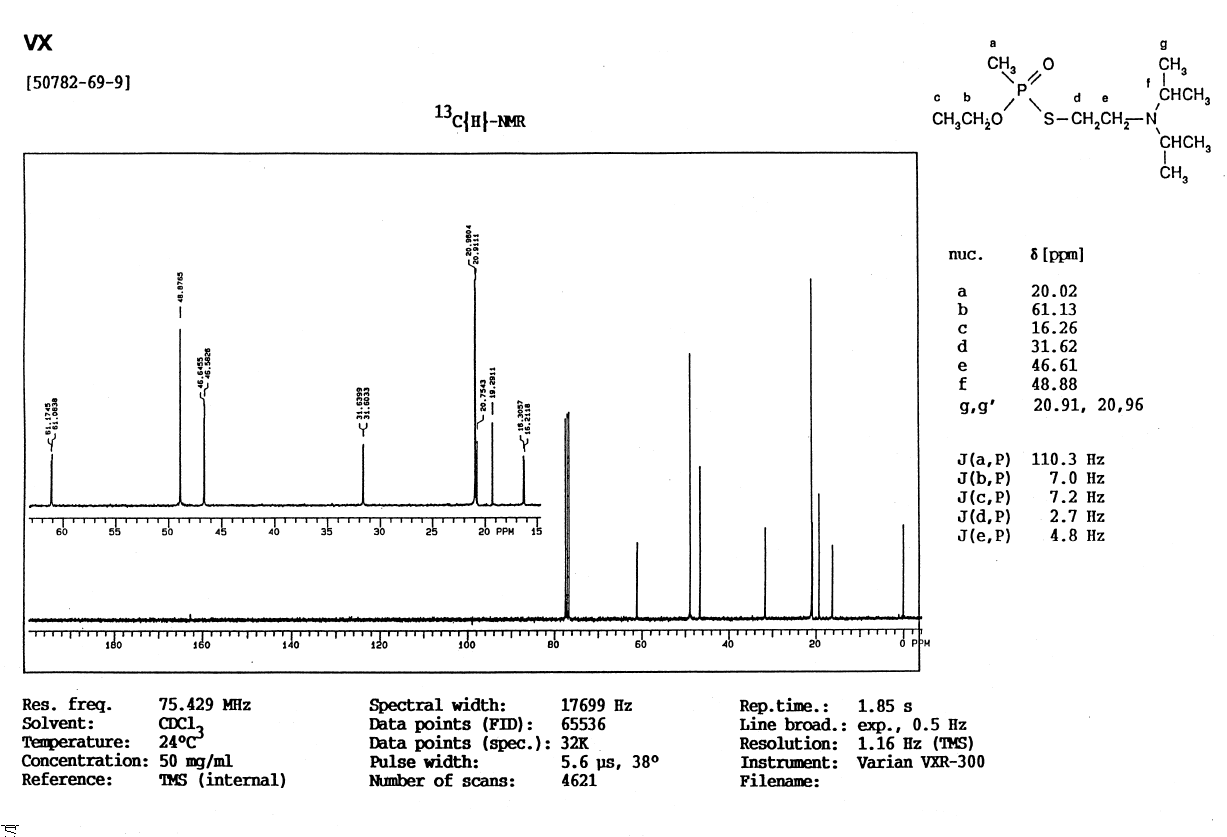


Figure S25. ^13^C NMR spectrum of VX.

Table S1. Scaling factor for prediction on chemical shift of ^1^H and ^13^C

|  | Chloroform | DMSO |
| --- | --- | --- |
| ^1^H | slope: −1.0951, intercept: 31.9773 | slope: −1.0602 intercept: 31.6739 |
| ^13^C | slope: −1.0379 intercept: 187.2065 | slope: −1.0499 intercept: 187.0919 |

Table S2. XYZ coordinates of conformers of nerve agents and Novichok agents

| Tabun−1 |
| --- |
| P 0.14949900 0.20352200 0.24019200  O −1.01684400 −0.01405800 −0.80043800  O −0.24217300 0.26004800 1.64817700  N 1.29263300 −0.84329500 −0.30300700  N 1.29502100 2.81506100 −0.52160700  C 2.60128200 −0.74374600 0.34473200  C 0.89799600 −2.21797500 −0.61202400  C −2.34797300 0.44211800 −0.44755500  C −3.10491400 −0.65030800 0.27067000  C 0.85898500 1.79219600 −0.24152900  H 3.34904800 −1.17810900 −0.31932600  H 2.61844200 −1.27102800 1.30294100  H 2.86349000 0.30102500 0.51228100  H 0.83383200 −2.83276700 0.29124100  H 1.64978000 −2.64675600 −1.27571600  H −0.06076000 −2.22340100 −1.12413800  H −2.26892500 1.34050300 0.16673700  H −2.80912200 0.70531900 −1.39730200  H −4.12432300 −0.31882100 0.47348000  H −3.15025900 −1.54910900 −0.34532600  H −2.61739000 −0.88368300 1.21686000 |
| Tabun −2 |
| P 0.29451400 0.21393200 0.63167300  O −0.94091000 −0.76177800 0.69277500  O 0.82901200 0.58751800 1.93383200  N 1.28414900 −0.41949400 −0.53391000  N −0.80721600 2.54321100 −0.82026600  C 2.41182500 0.42473700 −0.93523800  C 1.66054500 −1.82638600 −0.37007800  C −1.72871400 −1.07629000 −0.47983600  C −3.12059800 −0.51460800 −0.30864900  C −0.36266500 1.64869500 −0.25691100  H 2.79970100 0.05457000 −1.88471400  H 3.21136000 0.41174900 −0.18865500  H 2.08061200 1.45278000 −1.08182100  H 2.45934600 −1.94790800 0.36800700  H 2.00780100 −2.20459300 −1.33234100  H 0.79869400 −2.41217300 −0.05508500  H −1.23741200 −0.67808500 −1.36983500  H −1.73803400 −2.16410000 −0.54885200  H −3.73865600 −0.79863100 −1.16142300  H −3.57669300 −0.90481600 0.60064600  H −3.09013600 0.57339500 −0.25037300 |
| Tabun −3 |
| P −0.24327400 −0.27783700 0.49512200  O 1.16878900 −0.39099500 −0.20218900  O −0.31557500 −0.31301500 1.95196700  N −0.97175400 1.02403200 −0.19017400  N −1.57805000 −2.56183700 −0.80583200  C −0.99937000 1.13903200 −1.64536800  C −2.14117700 1.59055200 0.47993300  C 2.20026200 0.53684600 0.21763800  C 3.49734400 0.08846500 −0.40901600  C −0.99848400 −1.72097300 −0.28385600  H −1.07553300 2.19355900 −1.91427600  H −1.85444300 0.60311700 −2.07111700  H −0.08260800 0.73510900 −2.07138500  H −3.06563400 1.12010800 0.12939600  H −2.18714000 2.65846700 0.26152700  H −2.04728200 1.44964700 1.55378300  H 1.91402900 1.53705500 −0.11448700  H 2.25009200 0.52555900 1.30730800  H 4.29856700 0.76980400 −0.12113800  H 3.75394300 −0.91542600 −0.07276600  H 3.41659500 0.08373500 −1.49583600 |
| Tabun −4 |
| P 0.24041700 0.11074600 0.50540200  O −1.05775200 −0.56731500 −0.07977900  O 0.30935400 0.38725300 1.93573200  N 1.43945500 −0.79464600 −0.13217600  N 0.30441600 2.62038600 −1.07361100  C 1.45714600 −1.06955000 −1.56628800  C 2.76352300 −0.68132600 0.47878700  C −2.33945600 0.02797100 0.23950300  C −3.41190500 −0.85740300 −0.34497800  C 0.25050700 1.66895600 −0.43412800  H 2.01974500 −1.98810000 −1.73688100  H 1.93320900 −0.25401300 −2.12113200  H 0.44326000 −1.21146300 −1.93284100  H 3.35803100 0.10031400 −0.00514600  H 3.28007700 −1.63560100 0.36837400  H 2.65907700 −0.45477300 1.53719500  H −2.41875800 0.10446800 1.32444000  H −2.37080500 1.03143100 −0.19244900  H −4.39343700 −0.43695800 −0.12455400  H −3.29978900 −0.93070600 −1.42645000  H −3.35567400 −1.85736500 0.08392700 |
| Tabun −5 |
| P −0.06582700 −0.24460000 0.35289400  O 1.04427000 −0.32063800 −0.76518400  O 0.34164300 −0.21393200 1.75449700  N −1.05602300 0.98882300 −0.08687500  N −1.61446300 −2.64603000 −0.37031100  C −1.57357600 1.03374800 −1.45149300  C −1.96964100 1.52950400 0.91860100  C 2.13713000 0.63296900 −0.70013900  C 3.26499000 0.08265600 0.14196300  C −0.94779300 −1.75460800 −0.09407200  H −1.79304200 2.07020900 −1.71149300  H −2.49097300 0.44328700 −1.54812100  H −0.83068500 0.64926700 −2.14780300  H −2.92765500 0.99944800 0.90951400  H −2.15048900 2.58240600 0.69725300  H −1.51741000 1.44395400 1.90340100  H 2.43594900 0.78291100 −1.73603200  H 1.76156500 1.58041000 −0.30716900  H 4.09804000 0.78723300 0.14286100  H 2.92963900 −0.07227900 1.16687300  H 3.61191300 −0.86646200 −0.26605700 |
| Tabun −6 |
| P 0.24031600 0.11128700 0.50523000  O −1.05795600 −0.56709300 −0.07941100  O 0.30881900 0.38997700 1.93513600  N 1.43917000 −0.79581500 −0.13047600  N 0.30608000 2.61870600 −1.07726900  C 2.76356400 −0.67932000 0.47927500  C 1.45645900 −1.07383400 −1.56400900  C −2.33948800 0.02895200 0.23939100  C −3.41224600 −0.85680900 −0.34393700  C 0.25159800 1.66814900 −0.43653600  H 3.35641000 0.10262800 −0.00623000  H 3.28159500 −1.63288800 0.36976000  H 2.65958500 −0.45149000 1.53745800  H 2.01968800 −1.99233100 −1.73278500  H 1.93172800 −0.25919700 −2.12088000  H 0.44254300 −1.21728500 −1.92985600  H −2.41857700 0.10673400 1.32424700  H −2.37054100 1.03189800 −0.19375800  H −4.39363100 −0.43581000 −0.12391700  H −3.30025800 −0.93143400 −1.42533200  H −3.35626600 −1.85627200 0.08616000 |
| Tabun −7 |
| P 0.10914000 −0.17844800 0.38817200  O −0.87392800 −0.79873300 −0.67474300  O −0.14584800 −0.44263100 1.80041500  N 1.58816200 −0.57337400 −0.19268900  N −0.04461500 2.71932000 −0.20961600  C 1.86910400 −0.35963800 −1.61164900  C 2.71715400 −0.26025000 0.68622500  C −2.24989300 −1.05987600 −0.29299000  C −3.04716400 0.21784100 −0.15654700  C −0.04135400 1.59961700 0.04240700  H 2.73359600 −0.96505200 −1.88532000  H 2.09353900 0.69252500 −1.82080200  H 1.01929500 −0.67468200 −2.21217700  H 3.03881300 0.78063100 0.56942600  H 3.54810400 −0.91659900 0.42634100  H 2.43522700 −0.43969700 1.72137100  H −2.63100500 −1.68905800 −1.09423200  H −2.23996600 −1.62829000 0.63677600  H −4.09526800 −0.03028000 0.01616700  H −2.69828800 0.81178400 0.68936400  H −2.97484700 0.81856000 −1.06340400 |
| Tabun −8 |
| P 0.24922600 0.18930000 0.37434700  O −1.07554400 −0.36286200 −0.28464400  O 0.22746800 0.38829000 1.82184400  N 1.40016400 −0.72577200 −0.35463100  N 0.60226500 2.81507700 −0.93110300  C 2.78134900 −0.35915200 −0.03948500  C 1.19350000 −2.16501600 −0.51115400  C −2.33765700 0.11700100 0.24219700  C −3.43747500 −0.68424900 −0.40869000  C 0.47759200 1.78760100 −0.43727900  H 3.42677600 −0.72207800 −0.83984700  H 3.10617300 −0.78945000 0.91207000  H 2.88322900 0.72523000 0.01245200  H 1.43956600 −2.71046200 0.40514700  H 1.84344800 −2.51621800 −1.31359100  H 0.16162300 −2.36526800 −0.78665400  H −2.32555200 −0.00062300 1.32565200  H −2.42182900 1.17970500 0.00232100  H −4.40556300 −0.33932700 −0.04439400  H −3.40960500 −0.56460400 −1.49136700  H −3.33289500 −1.74206900 −0.16798900 |
| Tabun −9 |
| P 0.36859600 0.23858300 0.61274700  O −1.17483500 −0.08938100 0.61618500  O 0.95149400 0.36588400 1.94151500  N 1.01956100 −0.80483200 −0.49594400  N 0.49670000 2.77793800 −0.89367700  C 2.40216800 −0.53644000 −0.89895000  C 0.77860200 −2.22710000 −0.23035500  C −1.94755500 −0.12366000 −0.60327100  C −3.38251000 −0.39739700 −0.22370300  C 0.45823500 1.79163100 −0.31014500  H 2.61213700 −1.10365900 −1.80619100  H 3.11380200 −0.82660300 −0.12020400  H 2.53152000 0.52196700 −1.12332500  H 1.45381200 −2.61371200 0.53904300  H 0.93858900 −2.78325300 −1.15490900  H −0.24865700 −2.38091700 0.09798700  H −1.84470200 0.83887100 −1.10941300  H −1.54213400 −0.90172400 −1.25252200  H −3.99984100 −0.42958400 −1.12192500  H −3.46585600 −1.35342600 0.29285900  H −3.75836000 0.38732200 0.43169300 |
| Tabun −10 |
| P 0.07934200 −0.09347100 0.34282900  O −0.95245500 −0.27537300 −0.83458200  O −0.28594500 −0.53292200 1.68551500  N 1.45496200 −0.70702400 −0.28526600  N 0.49707900 2.84127800 0.27961100  C 1.90658200 −0.27482700 −1.60510500  C 2.53410500 −1.02789000 0.64834500  C −2.30787200 0.20517000 −0.64537800  C −3.15763500 −0.85302500 0.01961500  C 0.29324500 1.71278100 0.32378500  H 2.53580000 −1.05740900 −2.03057100  H 2.48717500 0.65172600 −1.54195600  H 1.05309600 −0.12134200 −2.26112600  H 3.20445800 −0.17370300 0.78836000  H 3.10757800 −1.86249900 0.24310200  H 2.11273200 −1.31926500 1.60750200  H −2.28331400 1.12984200 −0.06259600  H −2.65945700 0.44437100 −1.64690000  H −4.18256100 −0.49095500 0.11249400  H −3.16284500 −1.76420700 −0.57828600  H −2.77043000 −1.08116100 1.01209800 |
| Tabun −11 |
| P −0.29762900 −0.42548100 0.42512800  O 0.94142000 −0.97345700 −0.38297600  O −0.38362800 −0.70628000 1.85453100  N −0.42477400 1.16724000 0.04191800  N −2.46472600 −1.65344100 −1.15146400  C −0.38104000 1.56326200 −1.36296400  C −1.30076700 1.99689200 0.86891700  C 2.26878000 −0.78175500 0.17031700  C 2.92802100 0.43085300 −0.44738500  C −1.58644400 −1.24792400 −0.53525700  H −0.01923700 2.59071900 −1.42867400  H −1.37348800 1.50769900 −1.82304200  H 0.30122700 0.91834100 −1.91363300  H −2.33018700 1.98244900 0.49564200  H −0.93443100 3.02419200 0.84435100  H −1.28122800 1.63518100 1.89389600  H 2.19094400 −0.70030200 1.25559300  H 2.80542700 −1.69724000 −0.07015200  H 3.94765100 0.52428200 −0.07088200  H 2.96950600 0.33054900 −1.53244800  H 2.38110400 1.33968000 −0.19495700 |
| Tabun −12 |
| P 0.15300600 −0.11805400 0.38677700  O −0.68144100 −0.67268300 −0.83985100  O −0.06352700 −0.78157600 1.66988100  N 1.69328100 0.00327900 −0.13070700  N −0.72281200 2.70533500 0.40987800  C 2.70329000 −0.90333300 0.40545500  C 1.98583400 0.58258500 −1.43475800  C −1.99545900 −1.24289300 −0.61525000  C −3.01807700 −0.19186500 −0.24338100  C −0.37922500 1.61115500 0.40746700  H 3.63822800 −0.35527200 0.53291100  H 2.87584300 −1.74574800 −0.27075500  H 2.37640300 −1.27991700 1.37216400  H 2.01325400 −0.18431000 −2.21397100  H 2.95368700 1.08418800 −1.39013000  H 1.23090600 1.32330400 −1.69880300  H −2.24170500 −1.72405800 −1.55944900  H −1.90324400 −2.00195400 0.16159100  H −4.00649700 −0.65192100 −0.20658800  H −2.80926900 0.23396500 0.73895800  H −3.03440000 0.61241400 −0.97921700 |
| Tabun −13 |
| P 0.07926000 −0.09436700 0.34265900  O −0.95245500 −0.27496500 −0.83495700  O −0.28579700 −0.53564600 1.68481900  N 1.45507900 −0.70678600 −0.28631200  N 0.49683300 2.84056600 0.28307700  C 2.53410100 −1.02874200 0.64712900  C 1.90707400 −0.27123000 −1.60500300  C −2.30778800 0.20575600 −0.64595700  C −3.15792300 −0.85206900 0.01918000  C 0.29299300 1.71201500 0.32587700  H 3.20323300 −0.17405900 0.78998400  H 3.10892900 −1.86142400 0.23986600  H 2.11249700 −1.32336800 1.60518300  H 2.53689800 −1.05250100 −2.03195300  H 2.48706700 0.65552400 −1.53944300  H 1.05375600 −0.11677100 −2.26102300  H −2.28312400 1.13064600 −0.06351300  H −2.65924500 0.44469200 −1.64758800  H −4.18276700 −0.48968300 0.11171800  H −3.16324600 −1.76339100 −0.57850700  H −2.77100000 −1.08010400 1.01179000 |
| Tabun −14 |
| P −0.36070900 0.28232600 −0.64480500  O 1.15213500 −0.16588100 −0.71981600  O −0.95337300 0.50453000 −1.95621100  N −1.20399400 −0.71559900 0.34428800  N −0.07798500 2.70519500 1.03284600  C −2.40508100 −1.37717100 −0.16011100  C −1.06634900 −0.72682600 1.79374800  C 1.92884800 −0.54172300 0.42880500  C 3.33163300 −0.83883100 −0.04426700  C −0.19009800 1.78957000 0.35086700  H −3.29681000 −0.94256600 0.29966100  H −2.36506100 −2.44105300 0.08258900  H −2.46626400 −1.25518800 −1.23829600  H −0.91281000 −1.74853100 2.14920900  H −1.96988300 −0.32360300 2.25997500  H −0.22518000 −0.11649600 2.11717900  H 1.92474000 0.28430200 1.14654700  H 1.46986000 −1.41991400 0.88848800  H 3.95282000 −1.13168800 0.80269400  H 3.32186600 −1.65036400 −0.77104700  H 3.76738400 0.04373500 −0.51089800 |
| Tabun −15 |
| P 0.12540000 0.02636100 0.30434100  O −0.81238200 −0.86520000 −0.60002400  O −0.13026600 −0.03327600 1.74298200  N 1.60935200 −0.27869200 −0.33102500  N −0.36521200 2.78046400 −0.64109700  C 2.69927000 0.56019300 0.16889600  C 2.00514200 −1.66610400 −0.57269600  C −2.12262600 −1.22841100 −0.09399100  C −3.07319000 −0.05349600 −0.13787400  C −0.16359800 1.70766800 −0.28981900  H 3.50686200 0.55030000 −0.56361000  H 3.07793500 0.19907800 1.12945500  H 2.36257700 1.59021400 0.28739300  H 2.35728800 −2.15160000 0.34272700  H 2.81468600 −1.66629400 −1.30359300  H 1.16790400 −2.22537300 −0.98190700  H −2.44671100 −2.03453000 −0.74892600  H −2.00499500 −1.61008700 0.91989900  H −4.07237200 −0.38505500 0.14771600  H −2.76372800 0.72471300 0.56141900  H −3.11823200 0.36951700 −1.14158600 |
| Sarin−1 |
| P −1.02899000 −0.10796900 0.08875200  F −1.35870800 1.09443000 1.05357200  O 0.35433200 0.33596300 −0.52877100  O −1.05575100 −1.38468700 0.79391100  C 1.57954700 0.03763000 0.20546600  C 2.08559500 −1.32352100 −0.22308100  C 2.54214000 1.16417000 −0.09896000  C −2.17883200 0.15281200 −1.24413000  H 1.33965400 0.02672300 1.27205800  H 3.00956900 −1.55896300 0.30738500  H 1.34485500 −2.08984000 0.00223100  H 2.29054800 −1.32144100 −1.29530300  H 3.48151800 1.00196500 0.43102300  H 2.12546700 2.12239000 0.20980800  H 2.74797200 1.19908700 −1.17007800  H −3.19026300 0.04125800 −0.85672600  H −2.04454000 1.14982400 −1.65916200  H −2.00089500 −0.59809700 −2.01156300 |
| Sarin−2 |
| P 0.90106000 0.03314900 0.16326900  F 1.24575600 −1.48884600 −0.06406900  O −0.23858500 0.28137200 −0.89876200  O 0.59342400 0.31181700 1.56237300  C −1.65218400 0.07996200 −0.60116700  C −1.89169100 −1.27384900 0.04141100  C −2.17416600 1.23769400 0.22673800  C 2.31182000 0.83990600 −0.56322700  H −2.10581100 0.09865300 −1.59256400  H −2.96578300 −1.44550900 0.12327000  H −1.45696400 −2.07188500 −0.56050100  H −1.46088100 −1.30659100 1.04389300  H −3.25600300 1.14569600 0.33635100  H −1.95616700 2.18572500 −0.26542700  H −1.71434900 1.23486500 1.21489400  H 3.19748800 0.57160000 0.01052300  H 2.42694700 0.52515100 −1.59858800  H 2.16243800 1.91688500 −0.51568000 |
| Sarin−3 |
| P −1.02001900 0.03140300 0.14383600  F −1.26991300 1.10785100 −0.97954700  O 0.35972500 −0.60004300 −0.29576200  O −1.06682900 0.61238400 1.48107600  C 1.59447700 −0.10494300 0.29836200  C 2.66254300 −1.11282600 −0.06331600  C 1.89016000 1.28871100 −0.21979500  C −2.21283100 −1.21847700 −0.28371600  H 1.43913000 −0.07726000 1.37871100  H 3.61190200 −0.82253500 0.38807100  H 2.39329500 −2.10532600 0.29677500  H 2.78924100 −1.15436000 −1.14638100  H 2.84121400 1.63702000 0.18557500  H 1.11320200 1.99193200 0.08220100  H 1.95564700 1.27790700 −1.30920000  H −3.21092200 −0.79250200 −0.19508800  H −2.04132400 −1.55871500 −1.30328900  H −2.11113700 −2.05137400 0.40929600 |
| Sarin−4 |
| P −1.02001100 0.03126400 0.14396700  F −1.26970400 1.10863200 −0.97862800  O 0.35969300 −0.59999200 −0.29588000  O −1.06687600 0.61124900 1.48161700  C 1.59443300 −0.10486600 0.29826000  C 2.66246800 −1.11284000 −0.06323600  C 1.89019100 1.28869200 −0.22009300  C −2.21290000 −1.21814100 −0.28484300  H 1.43898900 −0.07699300 1.37858900  H 3.61189400 −0.82240100 0.38791100  H 2.39329200 −2.10523600 0.29717900  H 2.78898200 −1.15466200 −1.14631400  H 2.84116900 1.63707000 0.18540300  H 1.11317600 1.99196700 0.08163300  H 1.95586300 1.27774200 −1.30948500  H −3.21118000 −0.79427000 −0.18857400  H −2.04601400 −1.55201600 −1.30727800  H −2.10635100 −2.05497800 0.40266600 |
| Sarin−5 |
| P 0.90111300 −0.03322200 −0.16325500  F 1.24594400 1.48893200 0.06374400  O −0.23862300 −0.28098600 0.89858700  O 0.59374100 −0.31232500 −1.56235700  C −1.65231100 −0.07987600 0.60111600  C −1.89188300 1.27387800 −0.04153100  C −2.17428300 −1.23773800 −0.22657900  C 2.31170700 −0.83977100 0.56363200  H −2.10576000 −0.09852800 1.59259300  H −2.96598600 1.44544800 −0.12352100  H −1.45736400 2.07194500 0.56049500  H −1.46090100 1.30667400 −1.04391700  H −3.25610300 −1.14568700 −0.33626500  H −1.95636200 −2.18568900 0.26576900  H −1.71448700 −1.23512200 −1.21474900  H 3.19747500 −0.57188800 −0.01016200  H 2.42679400 −0.52492300 1.59895200  H 2.16218200 −1.91676300 0.51627100 |
| Sarin−6 |
| P 1.02894400 0.10772800 0.08881400  F 1.35878500 −1.09476400 1.05343900  O −0.35443500 −0.33636200 −0.52875700  O 1.05567400 1.38439000 0.79394900  C −1.57955400 −0.03769200 0.20551300  C −2.08489600 1.32381100 −0.22266900  C −2.54262700 −1.16370400 −0.09937100  C 2.17878700 −0.15230100 −1.24418900  H −1.33970500 −0.02722300 1.27211800  H −3.00876000 1.55951200 0.30788300  H −1.34374800 2.08965100 0.00294600  H −2.28986000 1.32221500 −1.29488400  H −3.48204900 −1.00117500 0.43042500  H −2.12643800 −2.12214900 0.20934800  H −2.74820400 −1.19836200 −1.17055000  H 3.19014400 −0.04054900 −0.85667200  H 2.04477800 −1.14901600 −1.65998900  H 2.00043700 0.59914300 −2.01102100 |
| Sarin−7 |
| P −1.06199400 0.05439200 −0.12782300  F −1.04459800 −1.41122900 0.45421900  O 0.27236600 0.11132700 −0.96163000  O −2.24221400 0.34102500 −0.92565800  C 1.64502400 −0.09839100 −0.55197400  C 2.22763800 1.19722900 −0.01805200  C 1.81765000 −1.27040200 0.39633200  C −0.88981500 1.01873500 1.36885400  H 2.13456400 −0.33988100 −1.49608300  H 3.30761600 1.09126200 0.09465400  H 2.02985200 2.01515100 −0.71042300  H 1.81264200 1.45192700 0.95798700  H 2.88492100 −1.44325300 0.54169800  H 1.36846100 −2.17438200 −0.01169800  H 1.37231200 −1.07508700 1.37318200  H −1.83401900 0.95533500 1.90852600  H −0.08547200 0.64494500 1.99954400  H −0.70377500 2.05732400 1.09932100 |
| Soman−1 |
| P 1.81716900 0.04367700 0.16146300  F 2.39035300 −1.02472200 −0.84479900  O 0.33560700 0.22609500 −0.35014600  O 1.97636900 −0.36885700 1.55155800  C −2.04217700 0.28020400 0.00357700  C −0.74946900 −0.49368600 0.30430000  C −2.34701700 0.29661300 −1.49589300  C −1.87944600 1.71771000 0.50909600  C −3.19783900 −0.38067100 0.76200900  C −0.70847000 −1.93961700 −0.15403900  C 2.63731500 1.52740200 −0.38222300  H −0.56037700 −0.44507700 1.38051200  H −2.56575600 −0.70381400 −1.87332900  H −1.50674200 0.70358600 −2.06079900  H −3.22179500 0.92134900 −1.68846100  H −1.62231600 1.73107700 1.57151300  H −2.81352700 2.26798000 0.38011200  H −1.09674400 2.24179400 −0.03972000  H −2.97333100 −0.46818800 1.82826800  H −3.42432500 −1.37496500 0.37508000  H −4.09927700 0.22634600 0.65872600  H 0.22896300 −2.40023900 0.16076700  H −1.52375400 −2.50961000 0.29032200  H −0.77614700 −2.00434000 −1.24024000  H 3.70934200 1.41025100 −0.23236100  H 2.27710400 2.36376400 0.21408700  H 2.42476900 1.70180000 −1.43547400 |
| Soman−2 |
| P −1.74761300 −0.09128000 0.16702900  F −2.52819000 1.26334900 −0.03156900  O −0.37346700 0.20722800 −0.54741400  O −1.68555200 −0.48491400 1.57045400  C 1.92222700 −0.33802900 −0.00197300  C 0.77647600 0.66110700 0.22518500  C 1.43861000 −1.73181500 0.41387500  C 3.10330000 0.06194100 0.88807100  C 2.35915600 −0.36301400 −1.46800500  C 1.03926800 2.09702000 −0.18406300  C −2.58744000 −1.16731200 −0.97564500  H 0.50241900 0.61704200 1.28292000  H 0.66263800 −2.09581100 −0.26147000  H 1.02739200 −1.72117200 1.42603400  H 2.26860500 −2.44040400 0.38310500  H 3.53270800 1.01926200 0.58925500  H 3.89101300 −0.69022700 0.81430800  H 2.80264200 0.12977300 1.93680400  H 2.82286200 0.57836400 −1.76815300  H 1.50870600 −0.55534400 −2.12479900  H 3.09326500 −1.15687700 −1.62136400  H 0.15927600 2.70399500 0.03008200  H 1.88094800 2.51089800 0.37062100  H 1.24847800 2.16441400 −1.25176900  H −3.62250900 −1.27720200 −0.65629400  H −2.09979000 −2.14050500 −0.96149000  H −2.54818300 −0.74505700 −1.97809400 |
| Soman−3 |
| P −1.81719800 −0.04364400 0.16141700  F −2.39001400 1.02442000 −0.84542600  O −0.33557900 −0.22659100 −0.34984200  O −1.97658700 0.36951200 1.55131200  C 2.04229000 −0.28014400 0.00355800  C 0.74946100 0.49345300 0.30447300  C 1.87993900 −1.71772800 0.50897400  C 3.19788000 0.38092700 0.76194200  C 2.34698900 −0.29633400 −1.49593500  C 0.70806800 1.93937000 −0.15385500  C −2.63758400 −1.52743800 −0.38168500  H 0.56050400 0.44477700 1.38070000  H 1.09722000 −2.24190600 −0.03972300  H 1.62304500 −1.73124200 1.57144700  H 2.81409900 −2.26780600 0.37974200  H 3.42420700 1.37523800 0.37496400  H 4.09940500 −0.22596300 0.65868100  H 2.97337200 0.46846600 1.82819900  H 2.56534500 0.70419900 −1.87331700  H 1.50679400 −0.70354800 −2.06078300  H 3.22196100 −0.92075600 −1.68863400  H −0.22927600 2.39986900 0.16139900  H 1.52350800 2.50948100 0.29007000  H 0.77520600 2.00404500 −1.24009200  H −3.70958900 −1.40998600 −0.23188100  H −2.27758700 −2.36358900 0.21505700  H −2.42505600 −1.70240200 −1.43483900 |
| Soman−4 |
| P 1.81725200 −0.04373000 −0.16140300  F 2.38993500 1.02543300 0.84414900  O 0.33559200 −0.22637300 0.35007200  O 1.97659300 0.36787900 −1.55174500  C −2.04223200 −0.28017100 −0.00359100  C −0.74945400 0.49349000 −0.30434800  C −1.87993800 −1.71773900 −0.50903500  C −3.19780500 0.38099000 −0.76204200  C −2.34706500 −0.29644900 1.49591300  C −0.70825600 1.93939800 0.15403700  C 2.63769100 −1.52703700 0.38300400  H −0.56038900 0.44491400 −1.38056500  H −1.09719800 −2.24199700 0.03955100  H −1.62313000 −1.73131000 −1.57153000  H −2.81410500 −2.26777800 −0.37972300  H −3.42426800 1.37517900 −0.37484200  H −4.09926500 −0.22603500 −0.65906800  H −2.97309900 0.46878900 −1.82823600  H −2.56530700 0.70405800 1.87339100  H −1.50694800 −0.70384200 2.06077100  H −3.22210900 −0.92080600 1.68846100  H 0.22908100 2.40004200 −0.16103400  H −1.52369100 2.50944300 −0.28998300  H −0.77552400 2.00402700 1.24026100  H 3.70979100 −1.40931100 0.23403900  H 2.27856000 −2.36338900 −0.21399600  H 2.42428200 −1.70186500 1.43598200 |
| Soman−5 |
| P 1.74754000 −0.09119600 −0.16710100  F 2.52857400 1.26307900 0.03179000  O 0.37343000 0.20769400 0.54719500  O 1.68542500 −0.48484000 −1.57050300  C −1.92206200 −0.33813800 0.00200800  C −0.77659000 0.66122800 −0.22534800  C −1.43806100 −1.73192300 −0.41338700  C −3.10311300 0.06132500 −0.88827600  C −2.35907800 −0.36271100 1.46802300  C −1.03979500 2.09704900 0.18390500  C 2.58705400 −1.16733000 0.97569600  H −0.50256500 0.61722700 −1.28308800  H −0.66208400 −2.09552300 0.26214800  H −1.02681900 −1.72144400 −1.42554900  H −2.26789500 −2.44070100 −0.38255900  H −3.53263000 1.01873600 −0.58991000  H −3.89076900 −0.69087400 −0.81424300  H −2.80241400 0.12873600 −1.93702800  H −2.82334300 0.57854200 1.76768100  H −1.50854500 −0.55421700 2.12496100  H −3.09274800 −1.15691500 1.62174100  H −0.16015600 2.70440100 −0.03063900  H −1.88188300 2.51056200 −0.37044900  H −1.24859100 2.16436700 1.25168300  H 3.62180900 −1.27839700 0.65574400  H 2.09851600 −2.14007800 0.96289400  H 2.54889500 −0.74402100 1.97775700 |
| Soman−6 |
| P 1.81719100 0.04364200 0.16141800  F 2.39021200 −1.02446800 −0.84525700  O 0.33558400 0.22632500 −0.34999600  O 1.97657900 −0.36932000 1.55136600  C −2.04223300 0.28016500 0.00357100  C −0.74946700 −0.49357400 0.30442500  C −3.19789200 −0.38083400 0.76189600  C −2.34694100 0.29650400 −1.49592100  C −1.87975300 1.71769900 0.50909500  C −0.70825100 −1.93950800 −0.15387000  C 2.63735600 1.52752100 −0.38181200  H −0.56042600 −0.44488100 1.38063900  H −2.97342900 −0.46842300 1.82815800  H −3.42429300 −1.37511600 0.37488100  H −4.09936500 0.22613000 0.65861300  H −1.50677200 0.70381700 −2.06073300  H −3.22194600 0.92090300 −1.68854900  H −2.56525000 −0.70399400 −1.87342500  H −1.09690700 2.24180600 −0.03948700  H −1.62299400 1.73112500 1.57160100  H −2.81382700 2.26791000 0.37978600  H 0.22907400 −2.40010600 0.16129900  H −1.52368900 −2.50953800 0.29016100  H −0.77549100 −2.00423300 −1.24010100  H 3.70937500 1.41036100 −0.23188500  H 2.27709600 2.36363500 0.21482600  H 2.42485300 1.70231400 −1.43499800 |
| Soman−7 |
| P 1.81719700 0.04365300 0.16142500  F 2.39003400 −1.02455700 −0.84522600  O 0.33556800 0.22649000 −0.34991000  O 1.97656700 −0.36926900 1.55138600  C −2.04226000 0.28013900 0.00356900  C −0.74943300 −0.49352600 0.30440900  C −3.19783500 −0.38076500 0.76208500  C −2.34707700 0.29632900 −1.49591900  C −1.87973500 1.71774200 0.50890300  C −0.70818900 −1.93946100 −0.15391300  C 2.63752900 1.52740200 −0.38188800  H −0.56045900 −0.44485900 1.38063800  H −2.97335900 −0.46800700 1.82837500  H −3.42416100 −1.37518400 0.37538900  H −4.09936500 0.22608900 0.65864400  H −1.50676700 0.70317900 −2.06086100  H −3.22181700 0.92108300 −1.68861200  H −2.56586500 −0.70414100 −1.87320900  H −1.09710900 2.24186600 −0.03997700  H −1.62261800 1.73126900 1.57132000  H −2.81390300 2.26784800 0.37985200  H 0.22916300 −2.40005100 0.16117700  H −1.52359000 −2.50949800 0.29017900  H −0.77552000 −2.00415400 −1.24013300  H 3.70962300 1.40967800 −0.23294200  H 2.27816200 2.36346000 0.21536100  H 2.42423200 1.70270800 −1.43483200 |
| Soman−8 |
| P −1.74764900 −0.09136800 0.16705400  F −2.52794200 1.26342700 −0.03116600  O −0.37345700 0.20688500 −0.54739700  O −1.68571600 −0.48534700 1.57039100  C 1.92230200 −0.33794600 −0.00195400  C 0.77641200 0.66098100 0.22519400  C 3.10322300 0.06209200 0.88825100  C 2.35940200 −0.36270000 −1.46795600  C 1.43884300 −1.73185800 0.41366400  C 1.03895700 2.09693000 −0.18409600  C −2.58758400 −1.16688700 −0.97600900  H 0.50235600 0.61686100 1.28293300  H 2.80247400 0.12966600 1.93697500  H 3.53247200 1.01954400 0.58963700  H 3.89107400 −0.68992000 0.81439700  H 1.50900800 −0.55489200 −2.12486500  H 3.09348900 −1.15657800 −1.62135300  H 2.82318900 0.57870300 −1.76790200  H 0.66317100 −2.09594500 −0.26197200  H 1.02729300 −1.72137700 1.42569500  H 2.26900800 −2.44025400 0.38313000  H 0.15888600 2.70376800 0.03010100  H 1.88061300 2.51094500 0.37052100  H 1.24807900 2.16432100 −1.25181900  H −3.62220600 −1.27826900 −0.65574700  H −2.09887400 −2.13957000 −0.96361300  H −2.54976900 −0.74328900 −1.97795000 |
| Soman−9 |
| P 1.74757700 −0.09131100 −0.16702700  F 2.52807600 1.26338400 0.03111100  O 0.37349500 0.20722500 0.54749800  O 1.68550600 −0.48540600 −1.57032700  C −1.92221300 −0.33803100 0.00197700  C −0.77647600 0.66108500 −0.22518500  C −3.10323200 0.06199600 −0.88812300  C −2.35923200 −0.36296700 1.46798100  C −1.43863000 −1.73184900 −0.41379500  C −1.03924100 2.09699700 0.18403200  C 2.58760400 −1.16691700 0.97590600  H −0.50235600 0.61695000 −1.28289900  H −2.80249100 0.12991200 −1.93682600  H −3.53264600 1.01929500 −0.58925000  H −3.89094100 −0.69018200 −0.81447000  H −1.50882500 −0.55529200 2.12483200  H −3.09335700 −1.15682800 1.62130800  H −2.82296800 0.57840300 1.76811500  H −0.66271500 −2.09582100 0.26162600  H −1.02735600 −1.72131700 −1.42593800  H −2.26866600 −2.44039100 −0.38304700  H −0.15920000 2.70392800 −0.03001900  H −1.88084800 2.51091300 −0.37074300  H −1.24858600 2.16440400 1.25171000  H 3.62201100 −1.27870100 0.65507600  H 2.09858900 −2.13944700 0.96403400  H 2.55053400 −0.74306600 1.97777000 |
| Soman−10 |
| P −1.81717300 −0.04366300 0.16144600  F −2.39013500 1.02456000 −0.84517100  O −0.33559200 −0.22639700 −0.34999700  O −1.97647700 0.36919100 1.55143600  C 2.04226400 −0.28016200 0.00355200  C 0.74947000 0.49350700 0.30445000  C 2.34727400 −0.29579900 −1.49588500  C 1.87959400 −1.71791700 0.50837500  C 3.19776400 0.38042800 0.76247200  C 0.70821600 1.93943800 −0.15383900  C −2.63754300 −1.52737200 −0.38191100  H 0.56043500 0.44475800 1.38066000  H 2.56622000 0.70480700 −1.87273500  H 1.50696800 −0.70230400 −2.06108800  H 3.22195300 −0.92058700 −1.68872700  H 1.62207800 −1.73179600 1.57069400  H 2.81382900 −2.26795100 0.37950200  H 1.09719300 −2.24190400 −0.04095800  H 2.97320100 0.46714200 1.82878400  H 3.42406500 1.37504200 0.37625800  H 4.09933500 −0.22632400 0.65879200  H −0.22902400 2.40007100 0.16152300  H 1.52379900 2.50943400 0.28997000  H 0.77522600 2.00409500 −1.24008800  H −3.70957600 −1.40980000 −0.23238600  H −2.27778900 −2.36359300 0.21487100  H −2.42478000 −1.70228100 −1.43502900 |
| Soman−11 |
| P −1.81726400 0.04355000 −0.16136100  F −2.38987100 −1.02379400 0.84634200  O −0.33556400 0.22689200 0.34952800  O −1.97684600 −0.37061700 −1.55092600  C 2.04235200 0.28013800 −0.00355600  C 0.74945700 −0.49334200 −0.30455100  C 2.34648500 0.29712800 1.49602700  C 1.88051900 1.71747000 −0.50987000  C 3.19811700 −0.38162400 −0.76106100  C 0.70794100 −1.93928500 0.15372000  C −2.63764800 1.52769500 0.38080500  H 0.56067700 −0.44472300 −1.38082200  H 2.56376400 −0.70333800 1.87422100  H 1.50647900 0.70552000 2.06028800  H 3.22199400 0.92083900 1.68859600  H 1.62487100 1.73041600 −1.57264500  H 2.81449800 2.26767000 −0.37983200  H 1.09714700 2.24187100 0.03768000  H 2.97384000 −0.47017200 −1.82728300  H 3.42435300 −1.37557200 −0.37309200  H 4.09960800 0.22538000 −0.65816100  H −0.22951000 −2.39964300 −0.16142600  H 1.52320900 −2.50946600 −0.29042700  H 0.77525800 −2.00409700 1.23994100  H −3.70962900 1.41026300 0.23077700  H −2.27744300 2.36350300 −0.21628600  H −2.42538400 1.70318100 1.43392300 |
| Soman−12 |
| P 1.81238900 −0.19888100 0.13603800  F 1.49415900 −1.01623500 −1.17627100  O 0.36753300 0.13940400 0.66855500  O 2.63649800 −0.92004800 1.09079000  C −1.91046900 −0.28169200 −0.02492300  C −0.69159200 0.64561900 −0.18294200  C −3.02683700 0.20619400 −0.95373900  C −2.40507800 −0.29753100 1.42332300  C −1.50454400 −1.69978900 −0.43922800  C −0.91225200 2.09887000 0.19353700  C 2.51258300 1.29209600 −0.55797200  H −0.36352600 0.57730200 −1.22649800  H −2.67497700 0.29775800 −1.98512400  H −3.42935600 1.17008500 −0.63903400  H −3.84883600 −0.51187000 −0.94633200  H −1.60048800 −0.57374800 2.10637000  H −3.20735500 −1.03070900 1.52781100  H −2.80399400 0.67195500 1.72698200  H −0.73315400 −2.10013000 0.21922700  H −1.12163900 −1.71770900 −1.46246300  H −2.37098100 −2.36179600 −0.38840200  H 0.00650700 2.66760400 0.04603900  H −1.69014300 2.54861000 −0.42251400  H −1.19427400 2.18307800 1.24275700  H 3.46580500 1.04205200 −1.02129200  H 2.68257000 2.00323100 0.24906300  H 1.84745000 1.72617800 −1.30382400 |
| Soman−13 |
| P 1.74755600 −0.09126600 −0.16703300  F 2.52830000 1.26327400 0.03130600  O 0.37345800 0.20748700 0.54739100  O 1.68547300 −0.48523400 −1.57036600  C −1.92215300 −0.33805000 0.00197500  C −0.77649900 0.66118600 −0.22524100  C −1.43821700 −1.73189800 −0.41330200  C −3.10306000 0.06144700 −0.88848500  C −2.35941600 −0.36261000 1.46792200  C −1.03944000 2.09708000 0.18398900  C 2.58728400 −1.16713400 0.97586600  H −0.50243500 0.61707100 −1.28297400  H −0.66251600 −2.09562100 0.26249500  H −1.02657500 −1.72149800 −1.42529900  H −2.26817100 −2.44054300 −0.38272700  H −3.53258500 1.01888300 −0.59021300  H −3.89074600 −0.69072700 −0.81451000  H −2.80220100 0.12878600 −1.93719200  H −2.82349200 0.57873200 1.76761500  H −1.50905000 −0.55442000 2.12498200  H −3.09330300 −1.15665400 1.62143000  H −0.15963900 2.70421700 −0.03047000  H −1.88139400 2.51076100 −0.37042300  H −1.24831100 2.16446500 1.25176200  H 3.62201900 −1.27815200 0.65583700  H 2.09880000 −2.13992300 0.96289700  H 2.54912300 −0.74400600 1.97799200 |
| Soman−14 |
| P −1.81718700 0.04364300 −0.16141800  F −2.39001400 −1.02439900 0.84545500  O −0.33556500 0.22656400 0.34982600  O −1.97659400 −0.36952700 −1.55129900  C 2.04228600 0.28014200 −0.00356100  C 0.74946400 −0.49347000 −0.30446900  C 3.19790900 −0.38099500 −0.76182300  C 2.34690800 0.29648500 1.49595500  C 1.87995700 1.71768400 −0.50911500  C 0.70806500 −1.93939100 0.15385300  C −2.63756400 1.52746000 0.38166500  H 0.56050100 −0.44480900 −1.38070000  H 2.97348000 −0.46858100 −1.82809300  H 3.42417800 −1.37529400 −0.37478100  H 4.09944400 0.22587300 −0.65852000  H 1.50670800 0.70380500 2.06071600  H 3.22190700 0.92087500 1.68862900  H 2.56518300 −0.70401700 1.87346700  H 1.09717600 2.24188600 0.03947300  H 1.62316600 1.73109900 −1.57161300  H 2.81409300 2.26779400 −0.37983900  H −0.22927300 −2.39989700 −0.16141300  H 1.52350500 −2.50950600 −0.29007000  H 0.77520000 −2.00407900 1.24008800  H −3.70957500 1.40996300 0.23190200  H −2.27760100 2.36358800 −0.21512600  H −2.42502200 1.70245800 1.43481100 |
| Cyclosarin−1 |
| P −2.04933700 −0.16261500 0.02823500  F −1.67200600 0.56830600 1.37729000  O −0.64293900 −0.70542400 −0.44217900  O −3.08300700 −1.17254700 0.18393200  C 0.63295600 −0.36372600 0.14686000  C 1.63758800 −1.36254400 −0.39770100  C 1.02895300 1.06756200 −0.17964400  C 3.03837200 −1.06426400 0.13706100  C 2.43267800 1.36749300 0.35199400  C 3.45579500 0.36946700 −0.18783200  C −2.46686000 1.20773900 −1.03742600  H 0.54613700 −0.48274400 1.23087800  H 1.63039000 −1.29142800 −1.49019200  H 1.31768700 −2.37203400 −0.13485800  H 0.30791700 1.76358800 0.25484200  H 1.00573200 1.19052400 −1.26824500  H 3.75135300 −1.77681300 −0.28115600  H 3.05077900 −1.20629900 1.22325200  H 2.42016300 1.31511100 1.44607100  H 2.71445300 2.38882500 0.08992700  H 4.44360900 0.58067200 0.22647400  H 3.53396200 0.48476900 −1.27443300  H −2.62400500 0.81834700 −2.04239700  H −3.39192800 1.65927900 −0.68254900  H −1.66746200 1.94608100 −1.05064900 |
| Cyclosarin−2 |
| P −1.94239700 0.06548600 0.15480100  F −2.12318500 1.17388200 −0.95090800  O −0.65330900 −0.70051000 −0.34220700  O −1.87998900 0.63375100 1.49696600  C 0.64425600 −0.33242800 0.19771800  C 1.61444400 −1.42439200 −0.20927600  C 1.07220100 1.03088200 −0.31997400  C 3.02454300 −1.09315900 0.28209900  C 2.48287100 1.36265900 0.17043400  C 3.47797800 0.27397700 −0.22955800  C −3.27360600 −1.05013100 −0.23502000  H 0.54708600 −0.29742700 1.28715300  H 1.60732600 −1.50042600 −1.30152400  H 1.27260400 −2.38189800 0.18839300  H 0.36224000 1.79207000 0.01159000  H 1.04794100 1.00722700 −1.41458600  H 3.71858700 −1.87299700 −0.03649900  H 3.03446400 −1.08929700 1.37750500  H 2.47062200 1.45817400 1.26151700  H 2.79201100 2.33120400 −0.22623000  H 4.47234600 0.51027000 0.15474100  H 3.55719900 0.24133900 −1.32174800  H −3.23136400 −1.89813200 0.44568800  H −4.21818800 −0.52500200 −0.10270600  H −3.17799100 −1.39271600 −1.26373600 |
| Cyclosarin−3 |
| P −1.94246400 0.06546600 0.15492800  F −2.12290900 1.17451600 −0.95003400  O −0.65332200 −0.70046000 −0.34266700  O −1.87995600 0.63285300 1.49743500  C 0.64415500 −0.33235500 0.19756700  C 1.61435200 −1.42441300 −0.20919900  C 1.07224100 1.03089000 −0.32016300  C 3.02440200 −1.09314700 0.28229400  C 2.48288000 1.36268600 0.17025500  C 3.47795000 0.27388500 −0.22952700  C −3.27353800 −1.04995700 −0.23583000  H 0.54681100 −0.29740900 1.28698700  H 1.60736700 −1.50059300 −1.30143700  H 1.27243300 −2.38184500 0.18856800  H 0.36227300 1.79214400 0.01120400  H 1.04803600 1.00708600 −1.41478700  H 3.71846600 −1.87305400 −0.03609900  H 3.03420500 −1.08912300 1.37770500  H 2.47061700 1.45837700 1.26132400  H 2.79208300 2.33114900 −0.22655700  H 4.47231200 0.51015900 0.15480000  H 3.55722800 0.24110000 −1.32170900  H −3.23126600 −1.89853200 0.44415200  H −4.21832800 −0.52522300 −0.10345300  H −3.17753100 −1.39155300 −1.26484800 |
| Cyclosarin−4 |
| P 1.78123200 −0.02339100 −0.21159500  F 2.06995800 1.51701700 −0.03509700  O 0.76942200 −0.31205000 0.96323700  O 1.35310100 −0.33748900 −1.57100100  C −0.67038600 −0.16671900 0.82440200  C −1.05830700 1.16811300 0.20651200  C −1.26073800 −1.34167600 0.06253700  C −2.58332500 1.29060600 0.16352000  C −2.78505100 −1.21558600 0.01654400  C −3.20752100 0.11993500 −0.59592100  C 3.29607800 −0.75040200 0.37745700  H −1.02021200 −0.19467600 1.85986500  H −0.65976900 1.21894600 −0.81202500  H −0.61377900 1.98341700 0.78095800  H −0.95956500 −2.27310400 0.54698200  H −0.85013900 −1.34292000 −0.95055000  H −2.86166700 2.23993700 −0.29724900  H −2.97563700 1.30829500 1.18657800  H −3.18882500 −1.28929500 1.03281700  H −3.20595400 −2.04764300 −0.55061600  H −4.29593500 0.20832600 −0.59731700  H −2.88081200 0.15514000 −1.64048100  H 3.19392700 −1.83381600 0.35997400  H 4.10445300 −0.45251100 −0.28838200  H 3.50112800 −0.41168300 1.39106200 |
| Cyclosarin−5 |
| P −2.04933700 −0.16261500 0.02823500  F −1.67200600 0.56830600 1.37729000  O −0.64293900 −0.70542400 −0.44217900  O −3.08300700 −1.17254700 0.18393200  C 0.63295600 −0.36372600 0.14686000  C 1.63758800 −1.36254400 −0.39770100  C 1.02895300 1.06756200 −0.17964400  C 3.03837200 −1.06426400 0.13706100  C 2.43267800 1.36749300 0.35199400  C 3.45579500 0.36946700 −0.18783200  C −2.46686000 1.20773900 −1.03742600  H 0.54613700 −0.48274400 1.23087800  H 1.63039000 −1.29142800 −1.49019200  H 1.31768700 −2.37203400 −0.13485800  H 0.30791700 1.76358800 0.25484200  H 1.00573200 1.19052400 −1.26824500  H 3.75135300 −1.77681300 −0.28115600  H 3.05077900 −1.20629900 1.22325200  H 2.42016300 1.31511100 1.44607100  H 2.71445300 2.38882500 0.08992700  H 4.44360900 0.58067200 0.22647400  H 3.53396200 0.48476900 −1.27443300  H −2.62400500 0.81834700 −2.04239700  H −3.39192800 1.65927900 −0.68254900  H −1.66746200 1.94608100 −1.05064900 |
| VX−1 |
| S 1.30080100 −1.93344900 −0.35313400  P 2.64673000 −0.50820400 0.40908900  O 2.13820300 0.92839400 −0.08900600  O 4.00592100 −0.87748500 −0.00792800  N −2.21545400 0.29306500 −0.32929900  C −3.25341000 −0.74795000 −0.30877400  C −2.29834600 1.27443700 0.76503700  C −0.88519900 −0.19231400 −0.67016800  C −0.32035500 −1.30462000 0.22410000  C −4.64477900 −0.14146400 −0.13220700  C −3.22945800 −1.55656600 −1.60582900  C −2.22081600 0.70840800 2.19103200  C −1.28641300 2.40291800 0.59481100  C 2.36698700 −0.33560200 2.17494400  C 2.38736300 1.26898800 −1.46927000  C 1.92343500 2.68898100 −1.68673500  H −3.09452700 −1.45183900 0.52511800  H −3.28069200 1.73750300 0.65859600  H −0.89582500 −0.56134900 −1.69745300  H −0.19539800 0.64790600 −0.66377300  H −0.23333700 −0.99873800 1.26385200  H −0.96387400 −2.18550100 0.20591300  H −4.79534400 0.29079100 0.85680000  H −5.39801500 −0.91831000 −0.26604600  H −4.81169600 0.63561800 −0.88200100  H −4.07434900 −2.24545600 −1.62255100  H −3.31110000 −0.88488800 −2.46351800  H −2.32330600 −2.15168000 −1.72011800  H −2.86737900 −0.16056100 2.32464600  H −2.53664100 1.47090200 2.90635800  H −1.20152100 0.42095000 2.45548400  H −1.53265400 3.21317500 1.28279900  H −0.26392800 2.08511100 0.81444700  H −1.31996100 2.79446400 −0.42333000  H 3.12673900 0.34969900 2.55158200  H 2.48303100 −1.30841700 2.64855600  H 1.37995500 0.07501300 2.38021700  H 3.45376200 1.15174800 −1.66487900  H 1.83740200 0.56734600 −2.10289800  H 2.11347000 2.98491700 −2.71901800  H 0.85467600 2.77961900 −1.49145900  H 2.45788300 3.37020200 −1.02458700 |
| VX−2 |
| S 1.32220200 −0.54480300 −1.56810800  P 2.22518300 0.79495100 −0.21998800  O 3.03871200 −0.09559100 0.83422300  O 1.28031300 1.67279900 0.48399800  N −2.37747600 −0.26359600 0.16244700  C −2.86439200 0.80101400 1.04649400  C −3.37045300 −1.12802800 −0.47444700  C −1.25573700 0.08849100 −0.68571600  C −0.14080400 −0.94180700 −0.52572100  C −1.90608400 0.95985600 2.22577700  C −3.09594200 2.16220500 0.37342800  C −4.03251900 −2.05450300 0.54315300  C −4.42678700 −0.39260300 −1.30702500  C 3.46689600 1.61053300 −1.23528100  C 4.07097000 −1.00045300 0.42483500  C 4.36632700 −1.92319700 1.58459000  H −3.82546200 0.45569200 1.43534600  H −2.80002000 −1.76465500 −1.15913900  H −1.53793000 0.16363800 −1.74522800  H −0.84741300 1.05424300 −0.39472200  H 0.16933000 −0.99396300 0.51772100  H −0.45800100 −1.93724000 −0.83726400  H −1.82491500 0.02111800 2.77507500  H −2.25398000 1.73989700 2.90653300  H −0.90960900 1.24174500 1.87315500  H −3.62219700 2.82688400 1.06165600  H −2.14840300 2.64341100 0.12280200  H −3.68954200 2.07600700 −0.53614500  H −3.27435300 −2.59233900 1.11290300  H −4.67562500 −2.77733200 0.03733200  H −4.65673500 −1.49782900 1.24510000  H −5.05594100 −1.10856400 −1.83968200  H −5.07566900 0.20903500 −0.66624100  H −3.96410300 0.26659700 −2.04411300  H 4.11804800 2.18737600 −0.57756100  H 2.95713500 2.28086000 −1.92471500  H 4.05426100 0.88414700 −1.79712200  H 3.73229000 −1.56461400 −0.44814800  H 4.95715700 −0.42155100 0.15050600  H 5.16640400 −2.61429400 1.31679900  H 4.67569100 −1.34888200 2.45742600  H 3.47787700 −2.49834600 1.84384000 |
| VX−3 |
| S 0.71628400 −1.08773500 −0.57011400  P 2.77433900 −0.82087200 −0.23709000  O 2.88884800 0.60994500 0.48083900  O 3.51086500 −0.97804100 −1.49830400  N −2.14042300 −0.16503200 −0.10837400  C −3.49757600 −0.70244400 −0.26574900  C −1.91195000 1.25587100 −0.41265800  C −1.43952600 −0.63212400 1.07133400  C 0.06592200 −0.40038700 1.00186000  C −3.47293700 −2.23036800 −0.34514100  C −4.51497900 −0.24547400 0.78787400  C −2.65771400 1.69855600 −1.66585100  C −2.16846700 2.24258300 0.73638100  C 3.29514500 −1.88781000 1.10958100  C 2.65492500 1.77874300 −0.32619700  C 2.71387500 2.98595500 0.57939400  H −3.84396900 −0.35553600 −1.23911900  H −0.84647500 1.32486900 −0.65955900  H −1.79630700 −0.17373900 2.00789900  H −1.60404600 −1.70553100 1.16322000  H 0.32423700 0.65697800 1.05246200  H 0.53731600 −0.90399200 1.84475400  H −2.64352000 −2.56680800 −0.96875300  H −4.40670600 −2.58604500 −0.78278000  H −3.38426400 −2.69440900 0.63918900  H −5.45993600 −0.77565800 0.65119500  H −4.15688500 −0.46244300 1.79738900  H −4.72099700 0.82231200 0.71888800  H −2.49641000 0.99456300 −2.48326100  H −2.28661000 2.67565900 −1.97729300  H −3.73117800 1.79627700 −1.48896900  H −1.85677200 3.24301100 0.42839600  H −3.22354100 2.29195200 1.00636900  H −1.60231500 1.98725800 1.63378300  H 4.34397600 −1.67407100 1.31606000  H 3.18784700 −2.92140400 0.78618300  H 2.70335300 −1.70952300 2.00520000  H 3.41360300 1.81651600 −1.10906400  H 1.67462100 1.68646700 −0.80400500  H 2.54476700 3.89388400 −0.00067000  H 1.94950600 2.92157200 1.35498700  H 3.69002700 3.05607200 1.05901700 |
| VX−4 |
| S −0.64851900 −0.26030800 −0.55688300  P −2.52189100 −0.95259400 0.10114900  O −3.36389500 0.33945200 0.54959400  O −2.44564600 −1.92107600 1.20164700  N 2.25079400 0.13626700 −0.03158900  C 3.25569900 1.10536400 −0.49121600  C 2.70728100 −1.24890100 0.12292600  C 1.40238200 0.61936100 1.03917900  C 0.06861000 −0.11336100 1.12891400  C 2.62896200 2.05527300 −1.51013900  C 3.95711700 1.89422700 0.62244900  C 2.99752300 −1.87671600 −1.23821200  C 3.88880100 −1.43545700 1.08095900  C −3.29778700 −1.53059200 −1.41779700  C −3.64788700 1.40433100 −0.36375300  C −4.11471300 2.59590000 0.44043800  H 4.02169500 0.52823900 −1.01251600  H 1.85627300 −1.79573700 0.53560500  H 1.87645500 0.56633400 2.03378700  H 1.19231700 1.67310500 0.84672900  H 0.14958900 −1.11649300 1.54052500  H −0.61842400 0.45053600 1.75908600  H 2.20913500 1.48922100 −2.34174400  H 3.37049000 2.75906800 −1.89414900  H 1.82167600 2.63584900 −1.05677400  H 4.77725700 2.47805100 0.20050500  H 3.27252100 2.59573300 1.10420500  H 4.36776600 1.23843500 1.39015200  H 2.14321500 −1.73696200 −1.90105800  H 3.18488500 −2.94603100 −1.12539800  H 3.88032500 −1.43983100 −1.70966900  H 4.08927000 −2.49821600 1.22865100  H 4.79594400 −0.97942000 0.67688300  H 3.68721500 −0.99414100 2.05915800  H −4.34864000 −1.73274100 −1.20749700  H −2.80351400 −2.45102500 −1.72282200  H −3.21324200 −0.78859600 −2.21193500  H −2.74562700 1.64553600 −0.93255600  H −4.42552300 1.07505900 −1.05858800  H −4.36335700 3.42234700 −0.22644700  H −4.99797700 2.33732600 1.02390700  H −3.33020800 2.92074900 1.12356900 |
| VX−5 |
| S −1.03980800 −1.47560100 −0.70784500  P −2.73641400 −0.78327500 0.29662800  O −3.06119800 0.59138900 −0.47113200  O −2.58894800 −0.61385500 1.75171100  N 2.58785100 −0.03205300 0.39572900  C 3.93947800 −0.56429700 0.16844600  C 2.43005200 1.43220600 0.45376400  C 1.55230100 −0.69662000 −0.37975300  C 0.15863200 −0.38940700 0.15911500  C 4.00539100 −2.05346700 0.51330200  C 4.52611300 −0.32607300 −1.22988200  C 3.55342300 2.09730000 1.24082800  C 2.22923500 2.14451200 −0.89263900  C −4.02318400 −1.92739900 −0.22167100  C −3.88027700 1.56217600 0.21036700  C −3.82991700 2.84399900 −0.58673800  H 4.58187000 −0.05940900 0.89017200  H 1.51735400 1.59812300 1.03512100  H 1.58109500 −0.45093100 −1.45235800  H 1.70656900 −1.77260600 −0.29881100  H 0.10005500 −0.59312200 1.22701800  H −0.13833500 0.64064400 −0.03186300  H 3.45671700 −2.25856500 1.43326500  H 5.04658600 −2.34632100 0.65555900  H 3.60247200 −2.68051900 −0.28436800  H 5.47287100 −0.86080300 −1.33271200  H 3.85212400 −0.69433200 −2.00706100  H 4.72161600 0.72942700 −1.41651500  H 3.71868200 1.58817100 2.19130700  H 3.27989200 3.13213200 1.44996200  H 4.49136000 2.11203400 0.68134000  H 2.03191200 3.20332700 −0.71230700  H 3.11000500 2.07395400 −1.53065700  H 1.37855700 1.74502500 −1.44729000  H −4.98196700 −1.54310100 0.12848900  H −3.83871300 −2.89844700 0.23418500  H −4.03539500 −2.01257600 −1.30742300  H −4.90343800 1.18003700 0.27392300  H −3.49830500 1.69485200 1.22274300  H −4.44664800 3.60461900 −0.10633900  H −2.80551500 3.21178200 −0.64573600  H −4.19955300 2.68187900 −1.59911300 |
| VX−6 |
| S 0.65129500 −0.19647700 0.52630000  P 2.59376400 −0.69074000 −0.12306100  O 3.50852100 0.14694600 0.88479900  O 2.79432000 −0.43845400 −1.56116300  N −2.32035200 0.20356000 0.10807100  C −3.29191400 1.28213300 0.32799000  C −2.75252600 −1.18261600 0.34768800  C −1.49876100 0.36403200 −1.07792900  C −0.24164500 −0.49444400 −1.05073800  C −2.58359500 2.63114900 0.47213600  C −4.41387200 1.38512600 −0.71350500  C −3.60622700 −1.30546600 1.60453400  C −3.43775000 −1.88918500 −0.83154000  C 2.95664200 −2.35986200 0.42532700  C 3.66697300 1.56198200 0.64849000  C 4.85233800 1.81810900 −0.25580300  H −3.75063500 1.07797800 1.29536000  H −1.83075200 −1.73694300 0.55635500  H −2.03091500 0.13897500 −2.01581600  H −1.18171900 1.40476600 −1.13517900  H −0.46971800 −1.55678200 −1.13154500  H 0.42555200 −0.21646800 −1.86423100  H −1.68318800 2.52495700 1.07877800  H −3.25305600 3.34283000 0.95752100  H −2.30777200 3.05866300 −0.49380300  H −5.01570400 2.27768200 −0.52902100  H −4.00349500 1.46507600 −1.72319400  H −5.08016900 0.52356800 −0.68093000  H −3.14108400 −0.78623800 2.44365500  H −3.70723100 −2.35916000 1.86716200  H −4.61211100 −0.90625600 1.45563400  H −3.62673400 −2.93162800 −0.56674200  H −4.39429500 −1.43104600 −1.08360500  H −2.81640400 −1.88920100 −1.72837600  H 3.99033500 −2.58921900 0.16664100  H 2.28855400 −3.04709700 −0.09171700  H 2.80944800 −2.43701500 1.50077400  H 2.74395900 1.96106900 0.22155200  H 3.80601800 2.00308600 1.63452600  H 4.98921900 2.89254100 −0.38962100  H 5.76035100 1.40504900 0.18429200  H 4.68611400 1.35692600 −1.22884300 |
| VX−7 |
| S 0.48294200 0.37195700 −0.72717400  P 2.43695800 0.79285000 −0.12641400  O 3.15203200 −0.64880300 −0.18301800  O 2.57506500 1.45171600 1.18492000  N −2.34860500 −0.14079700 −0.01267400  C −3.43506100 −0.89476400 −0.65423100  C −2.72687300 1.11543400 0.64248200  C −1.41395100 −0.95776100 0.73412500  C −0.04368100 −0.30807900 0.89518500  C −2.95601400 −1.45101300 −1.99359500  C −4.05027600 −2.00779300 0.20448600  C −3.12725500 2.16479800 −0.39166900  C −3.79571800 0.97752700 1.73204500  C 3.12116400 1.70621700 −1.51780200  C 4.37855600 −0.81274900 0.55415200  C 4.73722800 −2.27976700 0.52785600  H −4.22594400 −0.17247200 −0.86497000  H −1.81540100 1.48670400 1.11707400  H −1.77371000 −1.22913200 1.74096100  H −1.27605600 −1.89145700 0.18614200  H −0.03009000 0.50336800 1.61922300  H 0.68044200 −1.05860400 1.21119200  H −2.60377600 −0.64075700 −2.63205200  H −3.76045900 −1.98500100 −2.50384200  H −2.12838000 −2.15032800 −1.85184100  H −4.93316600 −2.41239800 −0.29384400  H −3.34970500 −2.83363000 0.34592600  H −4.35207800 −1.64542500 1.18725600  H −2.35061400 2.25237000 −1.15181500  H −3.25946100 3.13500300 0.09052700  H −4.06981000 1.91457300 −0.88301600  H −3.93572800 1.93104300 2.24461200  H −4.75745600 0.68731500 1.30200500  H −3.51424500 0.23364700 2.48013600  H 4.19111000 1.83454400 −1.34983200  H 2.64759200 2.68511200 −1.56632600  H 2.95685300 1.15834400 −2.44468300  H 5.16064500 −0.21055100 0.08190400  H 4.22949200 −0.44634100 1.57015600  H 5.66876000 −2.44466700 1.07080300  H 3.95054100 −2.86948700 0.99842500  H 4.86393100 −2.62586700 −0.49783500 |
| VX−8 |
| S 0.58952800 −1.85426600 0.45237800  P 2.26863400 −0.70678800 −0.05122500  O 2.06372900 0.70966100 0.68131600  O 2.48928100 −0.57128600 −1.50399100  N −1.90031300 0.12281300 0.13901000  C −2.23689200 1.52959000 0.38067500  C −2.95185500 −0.88616600 0.32199100  C −1.05885900 −0.10561200 −1.01789800  C −0.39624700 −1.47700600 −1.04559500  C −0.96822700 2.36212300 0.57652500  C −3.14294600 2.18564300 −0.67024200  C −3.81622000 −0.60020400 1.54470500  C −3.83917800 −1.16237100 −0.90154200  C 3.58726700 −1.48203800 0.89152700  C 2.43151000 1.91356000 −0.01930400  C 3.93440000 2.08420800 −0.08861100  H −2.76204000 1.55276000 1.33558100  H −2.42155500 −1.81893800 0.54546600  H −1.59166200 0.00762100 −1.97698100  H −0.26066100 0.63833100 −1.01965800  H −1.11679000 −2.29077100 −1.11809900  H 0.27826800 −1.53848100 −1.89824900  H −0.24619400 1.81106700 1.18143700  H −1.21991200 3.29702300 1.08011100  H −0.50035200 2.62597100 −0.37497400  H −3.24812400 3.25273800 −0.46186200  H −2.71698200 2.08363400 −1.67144100  H −4.14154200 1.74903500 −0.67614500  H −3.19750200 −0.38558700 2.41705000  H −4.42682100 −1.47679800 1.76442100  H −4.49460500 0.23948300 1.37750700  H −4.51636700 −1.98989300 −0.67975200  H −4.44575500 −0.29638800 −1.16756400  H −3.25492500 −1.44517400 −1.77872300  H 4.47701200 −0.85725800 0.80859300  H 3.78598600 −2.47093800 0.48323400  H 3.29375500 −1.55897000 1.93833700  H 1.99697700 1.88747000 −1.01986100  H 1.96464800 2.71643300 0.54960700  H 4.17709400 3.04938000 −0.53600700  H 4.36967200 2.04799600 0.91116400  H 4.37801400 1.30326700 −0.70791600 |
| VX−9 |
| S 0.73729900 −0.10174000 0.47206700  P 2.60392100 −0.86544300 −0.12837400  O 3.40148600 0.32579700 −0.85031100  O 2.51139100 −2.03918600 −1.00667400  N −2.19623700 0.08586900 0.02788600  C −3.21125300 1.09821000 0.35200900  C −2.61072300 −1.31512100 0.15853800  C −1.41677200 0.37941800 −1.15744500  C −0.05896100 −0.31379700 −1.17136900  C −2.57004000 2.23730400 1.14222900  C −3.99182200 1.64633000 −0.84989900  C −2.81092900 −1.68422300 1.62637300  C −3.83114300 −1.70715400 −0.68140200  C 3.46752200 −1.15505200 1.42272600  C 3.17631900 1.72817700 −0.68132700  C 3.60292100 2.23914600 0.67915700  H −3.93094100 0.60963800 1.01148300  H −1.76271100 −1.90774600 −0.19267000  H −1.93128100 0.11971200 −2.09797600  H −1.24192600 1.45682700 −1.18228600  H −0.11423300 −1.38062300 −1.37411600  H 0.57347300 0.13390500 −1.93830100  H −2.09232300 1.84757300 2.04119400  H −3.31601000 2.98222500 1.42726400  H −1.80518600 2.74232800 0.54692500  H −4.81148600 2.27696800 −0.50069300  H −3.35635200 2.26336400 −1.48891100  H −4.41456000 0.84801200 −1.45984100  H −1.92955600 −1.40380700 2.20361100  H −2.96858400 −2.75975800 1.72422600  H −3.68276400 −1.18722000 2.05690500  H −4.00092700 −2.78352100 −0.61816100  H −4.73274800 −1.20704200 −0.31928700  H −3.69435200 −1.45156900 −1.73410300  H 4.50628000 −1.38280000 1.18174700  H 3.01110700 −2.01164000 1.91553200  H 3.42478800 −0.28226800 2.07217000  H 3.76666400 2.19320000 −1.46982300  H 2.12173500 1.94625600 −0.86138200  H 3.57259600 3.32981700 0.68505500  H 2.93200500 1.88363500 1.46211400  H 4.62161400 1.92061400 0.90500900 |
| VX−10 |
| S 1.67353800 −0.54376100 1.68074700  P 2.37411300 −0.70940400 −0.30087100  O 2.17160900 0.71542100 −1.00343500  O 1.69333300 −1.76091900 −1.06774500  N −2.24727700 −0.16607200 0.54917100  C −3.04617600 1.02089800 0.20865700  C −2.56863400 −1.42819000 −0.15007600  C −0.81834600 0.09906500 0.61616000  C −0.06537500 −1.01228000 1.33730700  C −2.87128400 2.11649400 1.26291400  C −2.81486900 1.60532300 −1.19179400  C −4.07306000 −1.63894700 −0.27814100  C −1.87618800 −1.64833800 −1.50299000  C 4.15229000 −0.88442900 −0.09494000  C 2.64527300 1.93636900 −0.42130400  C 1.60224700 3.00584600 −0.65330500  H −4.08801800 0.70657000 0.27191400  H −2.21013600 −2.21996600 0.51604200  H −0.35742900 0.25047600 −0.37095500  H −0.66464900 1.01610400 1.18524000  H −0.51117000 −1.20721000 2.31304900  H −0.02763900 −1.93700200 0.76481900  H −2.87808600 1.68759900 2.26571700  H −3.69131400 2.83174900 1.18354500  H −1.94418200 2.67751700 1.12686100  H −3.35953600 2.54628000 −1.29738800  H −1.75520500 1.81327000 −1.36084100  H −3.15932400 0.93264800 −1.97622900  H −4.57860900 −1.46352800 0.67285800  H −4.26375300 −2.66870500 −0.58227800  H −4.51313900 −0.98701300 −1.03622800  H −2.15090600 −2.63463800 −1.88337800  H −2.18450800 −0.91086400 −2.24455500  H −0.78737300 −1.62436500 −1.42748900  H 4.60952300 −0.83925900 −1.08381500  H 4.35917100 −1.84899000 0.36423300  H 4.55031700 −0.08593100 0.53215600  H 2.81660300 1.80065900 0.64955900  H 3.59322500 2.18974300 −0.90123600  H 1.95186100 3.96416200 −0.26711500  H 1.39688100 3.10913100 −1.71874400  H 0.67522600 2.73841400 −0.14431000 |
| VX−11 |
| S −0.85739000 −1.31784200 −0.98655400  P −2.33711700 −0.76741900 0.41325700  O −2.44571500 0.83228800 0.37911100  O −2.05177200 −1.25032500 1.77120800  N 1.98895100 −0.05306800 −0.05554200  C 2.09569600 1.37838300 0.24942700  C 3.21892400 −0.71881700 −0.49241400  C 1.19500900 −0.82685900 0.87761000  C 0.39376400 −1.93411100 0.20538200  C 0.83697700 2.09848500 −0.22830900  C 2.37313600 1.72058900 1.72047400  C 3.57938800 −0.30766900 −1.91825400  C 4.42030600 −0.55366300 0.44574300  C −3.87846700 −1.34951100 −0.31317600  C −2.60475800 1.55258900 −0.84970200  C −2.69426300 3.02427300 −0.51715900  H 2.93506800 1.75418500 −0.34012600  H 2.98156900 −1.78602900 −0.52249100  H 1.80820500 −1.29318200 1.66722400  H 0.48492300 −0.17646900 1.38942800  H 1.02431400 −2.59212200 −0.39241100  H −0.12955000 −2.52874000 0.95153800  H 0.68984700 1.92924100 −1.29604100  H 0.90816400 3.17244900 −0.03930100  H −0.04124400 1.71856500 0.30084500  H 2.58290300 2.78773700 1.81734800  H 1.50667300 1.50216900 2.34784100  H 3.22654500 1.17100100 2.11582800  H 2.72602000 −0.46206800 −2.57921800  H 4.42373500 −0.89530000 −2.28377700  H 3.86653300 0.74472500 −1.97111800  H 5.25429800 −1.16634500 0.09806400  H 4.75911300 0.48458000 0.47125600  H 4.17812200 −0.86263600 1.46431200  H −4.70984800 −0.90402300 0.23410600  H −3.90550500 −2.43277200 −0.21148700  H −3.94136600 −1.08823300 −1.36924800  H −1.74736100 1.34403500 −1.49454900  H −3.51589400 1.21643300 −1.35300000  H −2.81552600 3.60394100 −1.43308300  H −3.54564600 3.21621900 0.13552100  H −1.78613700 3.35303600 −0.01294400 |
| VX−12 |
| S 1.48485200 −0.76700000 1.68649000  P 2.44224700 −0.69460000 −0.19422300  O 3.05001700 0.78010200 −0.31197200  O 1.53474300 −1.09718700 −1.28360700  N −2.38159900 −0.21429300 0.46314200  C −3.19825900 1.00509800 0.37861900  C −2.65554700 −1.29204200 −0.50953800  C −0.96150700 0.04730900 0.64240800  C −0.21246400 −1.18364000 1.13716800  C −3.10813000 1.81293300 1.67544100  C −2.91658200 1.91633100 −0.82392700  C −4.15068900 −1.50032600 −0.72224100  C −1.93006600 −1.18800900 −1.85868500  C 3.95639400 −1.63478400 −0.01062700  C 2.14487800 1.87307100 −0.54819400  C 2.96967400 3.11818800 −0.77167100  H −4.23206900 0.66765200 0.30636400  H −2.28997700 −2.20570100 −0.02902300  H −0.46379200 0.40182700 −0.27169400  H −0.84832700 0.82604100 1.39680300  H −0.70504900 −1.60268400 2.01530200  H −0.12306800 −1.95088200 0.37167700  H −3.16046000 1.15190800 2.54127500  H −3.93942700 2.51803100 1.72350900  H −2.18859400 2.39841400 1.73580600  H −3.49560900 2.83847000 −0.73738900  H −1.85961000 2.19139700 −0.86775900  H −3.18558200 1.44189200 −1.76678200  H −4.68203800 −1.54915800 0.22962200  H −4.30775700 −2.44111500 −1.25104000  H −4.58987000 −0.70609900 −1.33034800  H −2.15645200 −2.07744200 −2.45105900  H −2.25772600 −0.31979000 −2.43146700  H −0.84546500 −1.13882700 −1.74731600  H 4.55653100 −1.49636900 −0.90967500  H 3.69915100 −2.68520500 0.10938000  H 4.50678000 −1.28355900 0.86099500  H 1.53029600 1.63464100 −1.41896700  H 1.49473900 1.97638800 0.32512700  H 2.31247500 3.97096000 −0.94577700  H 3.58836900 3.32704700 0.10093800  H 3.61809700 2.99512100 −1.63886900 |
| VX−13 |
| S 1.24822800 −1.50989300 −0.92495000  P 2.83554100 −0.72217800 0.20962400  O 2.41490900 0.79289200 0.52696500  O 4.10097800 −0.91147100 −0.50994800  N −2.53183400 0.05057500 −0.49985000  C −3.25874200 −0.90012400 0.35459700  C −2.52634800 1.43870700 −0.00320500  C −1.28260700 −0.45247500 −1.04689900  C −0.19223100 −0.80997200 −0.02688100  C −4.67028100 −0.39388800 0.65023700  C −3.35591200 −2.27316300 −0.30908600  C −2.03477200 1.64347500 1.43827200  C −1.77610300 2.37655300 −0.94285200  C 2.73342300 −1.41537400 1.86095900  C 2.38558600 1.73335000 −0.56250200  C 2.10412600 3.09936000 0.01676600  H −2.74511000 −1.03755400 1.32029200  H −3.57062700 1.75787700 −0.03065600  H −1.50019100 −1.33212800 −1.65379200  H −0.87565500 0.28089400 −1.74144500  H 0.14583100 0.07703300 0.50368800  H −0.54923600 −1.54347100 0.69678400  H −4.67818500 0.49982700 1.27359200  H −5.23107400 −1.16500800 1.17920700  H −5.18687700 −0.16879200 −0.28580700  H −4.01180700 −2.91439000 0.28013700  H −3.77672700 −2.17622900 −1.31265600  H −2.39193300 −2.77701700 −0.38407700  H −2.53991700 0.98356800 2.14467000  H −2.23838600 2.67150300 1.74616300  H −0.95945700 1.48198500 1.53164700  H −1.98836100 3.40952700 −0.66376400  H −0.69377000 2.23807000 −0.87919000  H −2.08825300 2.22781300 −1.97747200  H 3.48188400 −0.92542500 2.48377600  H 2.94445200 −2.48111700 1.79768700  H 1.74076000 −1.25316000 2.27887700  H 3.34527700 1.69884400 −1.08077500  H 1.60372500 1.42580600 −1.26404900  H 2.04123200 3.83682600 −0.78441100  H 1.15816100 3.09719000 0.56069300  H 2.89837200 3.39438600 0.70210400 |
| A230−1 |
| P 1.91321200 0.00522600 −0.02871200  O 1.98765400 1.24414700 0.75632100  F 1.91491300 0.32485500 −1.59282200  N 0.66506000 −1.00895200 0.16179700  C −0.61912000 −0.95931400 0.30333500  N −1.44010400 0.08957400 0.10192200  C −2.89186700 −0.01452800 0.26800300  H −3.12756700 −0.72686300 1.05451100  H −3.24114500 0.95686400 0.62076500  C −0.98959500 1.41134800 −0.34414400  H −0.05563400 1.31637600 −0.88812000  H −1.73045900 1.76684900 −1.06485400  C −1.27091000 −2.25163300 0.75110900  H −1.61047200 −2.15767900 1.78490100  H −2.12738800 −2.52547900 0.13652800  H −0.52190700 −3.03431800 0.70118700  C −3.60558800 −0.38103500 −1.02871100  H −3.28949800 −1.36027200 −1.38926200  H −4.68492900 −0.40274600 −0.87270300  H −3.39061800 0.34855500 −1.81015800  C −0.85196700 2.39916300 0.80585900  H −0.09496300 2.05318900 1.50605600  H −0.53063000 3.36661900 0.41861300  H −1.80324500 2.53881800 1.32331300  C 3.36657900 −1.03040300 0.08053700  H 4.24258700 −0.43157900 −0.16370900  H 3.45374700 −1.39860000 1.10144300  H 3.27862200 −1.87093200 −0.60496400 |
| A230−2 |
| P 2.12672700 0.11509500 0.07179700  O 2.53260400 1.38728300 0.68569500  F 2.77371300 −0.06151500 −1.36219100  N 0.55988100 −0.21767600 −0.24004400  C −0.46999400 0.56625000 −0.16201100  N −1.68119100 0.04033500 −0.44510700  C −1.78493700 −1.38662200 −0.75952600  H −2.75296100 −1.53464900 −1.24080600  H −1.00521300 −1.63314300 −1.48015500  C −2.91921300 0.81040900 −0.46637500  H −2.68466500 1.86871500 −0.53023300  H −3.44961200 0.55454000 −1.38713300  C −0.41558600 2.02729600 0.21680400  H −0.61701300 2.64406200 −0.66147600  H −1.16459100 2.25880800 0.97425500  H 0.57365300 2.26948100 0.59213000  C −1.65976500 −2.28537500 0.46550900  H −2.43116300 −2.05961200 1.20237800  H −1.76311800 −3.33047000 0.16972300  H −0.68329100 −2.15282100 0.92846900  C −3.81179200 0.53802200 0.73880400  H −4.70941600 1.15586900 0.69034100  H −4.12334000 −0.50678900 0.76756500  H −3.28360300 0.76265500 1.66692200  C 2.75579700 −1.34303700 0.89795600  H 3.83778400 −1.25626000 0.98692700  H 2.31900300 −1.39593600 1.89442600  H 2.49450700 −2.23576700 0.33296600 |
| A230−3 |
| P −2.12663600 −0.11514800 0.07184700  O −2.53235500 −1.38726400 0.68599700  F −2.77394500 0.06127000 −1.36202100  N −0.55984900 0.21761100 −0.24032700  C 0.47008600 −0.56621700 −0.16236000  N 1.68125700 −0.04015800 −0.44542600  C 1.78487500 1.38682900 −0.75971900  H 2.75292100 1.53503700 −1.24089900  H 1.00518000 1.63330800 −1.48038800  C 2.91935900 −0.81011400 −0.46634200  H 2.68492300 −1.86840200 −0.53092800  H 3.45026500 −0.55369700 −1.38664500  C 0.41583300 −2.02730100 0.21629900  H 0.61732100 −2.64389700 −0.66208500  H 1.16493700 −2.25884700 0.97365000  H −0.57334700 −2.26960400 0.59169200  C 1.65945400 2.28542300 0.46542500  H 2.43100100 2.05986900 1.20219600  H 1.76234600 3.33060100 0.16978700  H 0.68306700 2.15238300 0.92843400  C 3.81125700 −0.53829900 0.73949600  H 3.28261900 −0.76354400 1.66721000  H 4.70901300 −1.15596700 0.69116300  H 4.12261500 0.50654300 0.76903200  C −2.75558900 1.34304300 0.89811600  H −3.83753400 1.25616900 0.98748200  H −2.31851000 1.39579800 1.89447800  H −2.49444700 2.23580100 0.33311700 |
| A230−4 |
| P 2.11971700 −0.17265300 −0.05602000  O 2.51869700 −1.57163500 0.15426600  F 2.64327300 0.75223500 1.11909200  N 0.54824800 0.26167200 −0.12729300  C −0.49895200 −0.45005500 0.15585000  N −1.71114100 0.12059000 0.00029800  C −1.80933200 1.49638200 −0.48596400  H −1.04752100 1.64373000 −1.24867800  H −2.78802700 1.60183300 −0.95796300  C −2.96832700 −0.52634400 0.35890400  H −2.79607400 −1.25274700 1.14976200  H −3.61517500 0.24421100 0.78496400  C −0.46804700 −1.87147600 0.66438300  H −0.67592000 −1.87466700 1.73697300  H −1.22075200 −2.48440000 0.17066400  H 0.51894600 −2.29475700 0.50580100  C −1.63869700 2.51070800 0.63710800  H −0.64950900 2.40591300 1.08155300  H −1.74314100 3.52562800 0.25143200  H −2.39139700 2.36308600 1.41403900  C −3.65262700 −1.18134400 −0.83538300  H −3.03182300 −1.97697700 −1.24895100  H −4.61122900 −1.60890100 −0.53875000  H −3.83452100 −0.45162100 −1.62559200  C 2.89275800 0.61800800 −1.46303600  H 3.97228200 0.50181100 −1.37801600  H 2.55062600 0.12647500 −2.37256200  H 2.62803700 1.67302800 −1.49254500 |
| A230−5 |
| P −1.88850000 −0.02733400 −0.20107700  O −1.80669200 0.27608800 −1.63685300  F −2.01171400 1.31529400 0.65269600  N −0.68228100 −0.86250200 0.48987200  C 0.57679600 −0.98849100 0.20155100  N 1.37123400 −0.05516000 −0.35160100  C 2.73723400 −0.34844200 −0.78544900  H 2.84790900 −1.41988300 −0.92399500  H 2.86345200 0.10342700 −1.77177200  C 0.96880800 1.35706400 −0.43897000  H 1.72244000 1.85251800 −1.05197200  H 0.02964500 1.44132500 −0.98264800  C 1.20790600 −2.31539600 0.56219800  H 1.31813000 −2.92890300 −0.33488100  H 2.18715500 −2.20480000 1.02704800  H 0.53216900 −2.82538900 1.24108200  C 3.79943700 0.18115600 0.17026900  H 3.66455200 −0.23405300 1.17043900  H 4.79349100 −0.08965300 −0.18777800  H 3.75382400 1.26815400 0.24468400  C 0.89695900 2.01744000 0.93358300  H 0.53203400 3.03980800 0.83031400  H 0.20776400 1.48397900 1.58575100  H 1.87918300 2.04302100 1.40702900  C −3.38790700 −0.85115300 0.31850800  H −4.24321800 −0.25883900 −0.00298600  H −3.42782800 −1.83165600 −0.15255200  H −3.39230900 −0.96482900 1.40092100 |
| A230−6 |
| P 2.11958600 −0.17281100 −0.05607800  O 2.51868700 −1.57172900 0.15442100  F 2.64214800 0.75201700 1.11954200  N 0.54813200 0.26122400 −0.12847500  C −0.49912700 −0.45015200 0.15540700  N −1.71119600 0.12086100 0.00055200  C −1.80926400 1.49657700 −0.48599300  H −1.04765100 1.64361100 −1.24897400  H −2.78808400 1.60211400 −0.95770200  C −2.96843400 −0.52608800 0.35895400  H −2.79635900 −1.25213600 1.15020200  H −3.61546300 0.24460000 0.78447700  C −0.46835500 −1.87147000 0.66417900  H −0.67529700 −1.87428500 1.73697100  H −1.22174100 −2.48426300 0.17137400  H 0.51834700 −2.29517400 0.50498200  C −1.63798600 2.51098700 0.63692300  H −0.64870100 2.40577600 1.08106900  H −1.74212200 3.52593900 0.25126600  H −2.39050500 2.36362500 1.41407500  C −3.65241500 −1.18147800 −0.83528000  H −3.03162600 −1.97730900 −1.24847900  H −4.61112800 −1.60883500 −0.53872000  H −3.83403300 −0.45195100 −1.62573800  C 2.89369600 0.61820000 −1.46235200  H 3.97313800 0.50194100 −1.37639100  H 2.55233700 0.12690100 −2.37228800  H 2.62903300 1.67323800 −1.49177100 |
| A230−7 |
| P 2.12673500 0.11452900 0.07287800  O 2.53145100 1.38321300 0.69474400  F 2.77201400 −0.05126400 −1.36328700  N 0.55981300 −0.21863300 −0.23821600  C −0.46972400 0.56590300 −0.16139300  N −1.68101100 0.04043900 −0.44483900  C −1.78522400 −1.38655300 −0.75897300  H −2.75298200 −1.53422500 −1.24091200  H −1.00513200 −1.63356200 −1.47903300  C −2.91885200 0.81082600 −0.46664200  H −2.68399500 1.86911100 −0.52951200  H −3.44852900 0.55571600 −1.38803700  C −0.41474000 2.02719200 0.21654000  H −0.61650700 2.64353100 −0.66194700  H −1.16334300 2.25923000 0.97424200  H 0.57460700 2.26916800 0.59168900  C −1.66126000 −2.28507700 0.46634400  H −2.43306600 −2.05884600 1.20263600  H −1.76483600 −3.33019500 0.17072800  H −0.68505100 −2.15278200 0.92993600  C −3.81253700 0.53787500 0.73757500  H −4.70978300 1.15625400 0.68882900  H −4.12473900 −0.50676400 0.76526200  H −3.28502400 0.76149800 1.66631700  C 2.75934400 −1.34806000 0.88829100  H 3.84156600 −1.26121200 0.97417100  H 2.32591200 −1.40680900 1.88590900  H 2.49650000 −2.23765800 0.31911800 |
| A230−8 |
| P −2.11909100 −0.18349100 −0.06304100  O −2.53507000 −1.48491500 0.47865200  F −2.53354900 −0.03981500 −1.58387200  N −0.54738300 0.25033200 −0.12490800  C 0.49946500 −0.43819000 0.20932800  N 1.70991500 0.13283400 0.03976800  C 1.80516700 1.47482200 −0.53366700  H 2.78648500 1.55554000 −1.00476400  H 1.04739900 1.56926400 −1.30875400  C 2.96927600 −0.49696400 0.41981800  H 3.61730100 0.29090900 0.81128500  H 2.80090900 −1.19133600 1.23954800  C 0.47431500 −1.83174400 0.79147300  H 1.18800300 −2.47868900 0.28225700  H 0.74905200 −1.79045000 1.84780000  H −0.52523800 −2.24601000 0.71163700  C 1.62455800 2.55863900 0.52089600  H 2.37085600 2.46271800 1.31196100  H 1.72948200 3.54713400 0.07196100  H 0.63200600 2.48053600 0.96373800  C 3.64927800 −1.20080000 −0.74917000  H 3.82955700 −0.50487700 −1.56954400  H 4.60838300 −1.61658400 −0.43780700  H 3.02665300 −2.01216200 −1.12779400  C −2.97117200 1.22441400 0.64203900  H −4.04470100 1.06514500 0.55010600  H −2.71030900 1.29931100 1.69684100  H −2.68001000 2.13635200 0.12444600 |
| A230−9 |
| P 1.85824900 −0.10199200 0.30402900  O 1.71396900 0.30019600 1.70996900  F 2.09279300 1.17571400 −0.62460700  N 0.65918900 −0.92555400 −0.40425700  C −0.63469900 −0.91214200 −0.42247000  N −1.44897200 0.11092800 −0.10560600  C −2.90804900 0.00484700 −0.16740700  H −3.28361300 0.97247700 −0.50585700  H −3.20066200 −0.71865500 −0.92372900  C −0.95466300 1.44474700 0.25260100  H −1.71274600 1.89687600 0.89472800  H −0.06009700 1.36031800 0.86351500  C −1.29789500 −2.18859300 −0.89574400  H −2.07032800 −2.52507200 −0.20403700  H −1.75858000 −2.04242000 −1.87475100  H −0.53026400 −2.95010000 −0.97882000  C −3.51596200 −0.34987700 1.18414000  H −3.22940200 0.38169200 1.94055800  H −4.60462300 −0.36831800 1.11922600  H −3.17141300 −1.32882400 1.51934500  C −0.72853800 2.30226000 −0.98591000  H −0.34448800 3.28115000 −0.69748200  H 0.00135500 1.83382100 −1.64446900  H −1.65988400 2.44692900 −1.53734200  C 3.34243700 −1.02626300 −0.06811100  H 4.20546100 −0.45509100 0.27042600  H 3.30359900 −1.97453800 0.46508900  H 3.40774700 −1.21085500 −1.13867500 |
| A230−10 |
| P −2.12673500 −0.11503300 0.07190100  O −2.53246700 −1.38678300 0.68678000  F −2.77370100 0.06035800 −1.36224900  N −0.55990700 0.21763800 −0.23998000  C 0.46994400 −0.56632100 −0.16197400  N 1.68118100 −0.04039500 −0.44493500  C 2.91920100 −0.81047000 −0.46629600  H 2.68466600 −1.86879900 −0.52980800  H 3.44939900 −0.55482600 −1.38723200  C 1.78492900 1.38651300 −0.75952100  H 2.75297200 1.53449900 −1.24077000  H 1.00522500 1.63296600 −1.48019800  C 0.41549200 −2.02737100 0.21677100  H 0.61697400 −2.64409500 −0.66151200  H 1.16447500 −2.25889800 0.97424000  H −0.57372500 −2.26951300 0.59215000  C 3.81203800 −0.53774300 0.73861200  H 3.28410400 −0.76224800 1.66690200  H 4.70971800 −1.15550200 0.69004100  H 4.12348800 0.50710600 0.76710100  C 1.65968800 2.28534700 0.46545400  H 1.76306200 3.33043300 0.16965400  H 0.68317000 2.15280900 0.92832900  H 2.43102500 2.05957600 1.20238300  C −2.75594900 1.34371500 0.89693600  H −3.83767500 1.25599100 0.98789300  H −2.31746100 1.39844300 1.89256800  H −2.49631800 2.23585900 0.33026800 |
| A232−1 |
| P −1.56703100 0.00435600 −0.13209100  O −1.57877600 0.31147800 −1.56504100  F −1.68454900 1.34675100 0.70331800  O −2.84645700 −0.76353700 0.39821100  N −0.36709300 −0.83064300 0.52203500  C 0.88196200 −0.98478500 0.21055700  N 1.68058000 −0.07084600 −0.36629800  C 3.03812800 −0.38744100 −0.81119300  H 3.13326300 −1.46210700 −0.93498300  H 3.15856600 0.04853000 −1.80530700  C 1.29209500 1.34423700 −0.47011200  H 2.04015500 1.82131500 −1.10403400  H 0.34456900 1.43069700 −0.99938400  C 1.49304000 −2.31918900 0.57482300  H 1.58327800 −2.94023900 −0.31923900  H 2.47871400 −2.22111900 1.02872700  H 0.81480800 −2.81226800 1.26352900  C 4.11689500 0.14225200 0.12546300  H 3.98646700 −0.25579000 1.13313800  H 5.10322200 −0.14816100 −0.23840800  H 4.08745600 1.23080100 0.18324300  C 1.24916400 2.02814300 0.89229600  H 0.89514500 3.05305000 0.77706100  H 0.56492000 1.51420900 1.56505100  H 2.23890600 2.04997200 1.34970900  C −4.11392600 −0.31266600 −0.08883100  H −4.11247700 −0.29056500 −1.17809700  H −4.85187800 −1.02238500 0.27476300  H −4.33541000 0.68155000 0.30218700 |
| A232−2 |
| P 1.79463200 0.16601500 −0.21518000  O 2.21630500 1.52815100 −0.55489700  F 2.29414400 −0.84544500 −1.31307200  O 2.48307500 −0.42991700 1.08232000  N 0.25263500 −0.26459400 −0.01972100  C −0.81412000 0.44828300 −0.21579800  N −2.00439400 −0.12184300 0.04699800  C −2.05630300 −1.49297500 0.55732100  H −1.22781900 −1.63085500 1.24885600  H −2.98857300 −1.59156600 1.11649400  C −3.28910300 0.52368800 −0.19976500  H −3.18871100 1.24670500 −1.00597500  H −3.97103000 −0.24917600 −0.56194700  C −0.82576100 1.86596300 −0.73452200  H −1.14612100 1.86550900 −1.77888100  H −1.51778700 2.48665300 −0.16720200  H 0.17445500 2.28464900 −0.68353600  C −1.98737900 −2.51935700 −0.56534900  H −1.03978800 −2.42459000 −1.09449200  H −2.06005700 −3.52911700 −0.15973600  H −2.80454900 −2.37777400 −1.27545700  C −3.86202700 1.18392300 1.04896300  H −3.20638200 1.98094500 1.40156200  H −4.84340800 1.61060300 0.83812900  H −3.97182400 0.45769000 1.85540400  C 3.87446500 −0.14796000 1.26539100  H 4.05540200 0.92342500 1.18803600  H 4.13185000 −0.50611900 2.25836300  H 4.46620500 −0.67841500 0.51796000 |
| A232−3 |
| P −1.80049200 −0.30315200 −0.15990500  O −2.23161500 −1.57461100 0.42801000  F −2.43304100 −0.11996500 −1.58769000  O −2.35337500 0.98336000 0.58605300  N −0.25583700 0.08476000 −0.41012300  C 0.80921300 −0.61683800 −0.17277200  N 2.00062100 −0.03579200 −0.41110000  C 2.03866200 1.36478100 −0.84511300  H 3.03801300 1.54144300 −1.24590500  H 1.31972500 1.48845800 −1.65488600  C 3.28351300 −0.71046100 −0.25152700  H 3.12517900 −1.78431300 −0.22085200  H 3.87444100 −0.50889100 −1.14850600  C 0.81022000 −2.03647900 0.34174500  H 1.14659500 −2.71493600 −0.44463900  H 1.48610000 −2.13759100 1.19105800  H −0.19484800 −2.31983000 0.63789700  C 1.73067600 2.35200100 0.27480700  H 2.42993300 2.23891300 1.10401600  H 1.80954400 3.37226200 −0.10347000  H 0.71749400 2.19912500 0.64276000  C 4.04538800 −0.25186200 0.98635400  H 3.45348800 −0.42199100 1.88718700  H 4.98322900 −0.80150400 1.07603700  H 4.28189400 0.81125400 0.92928100  C −3.71147000 0.93764900 1.03658200  H −3.87948200 0.04424500 1.63673500  H −3.86697900 1.83172600 1.63407700  H −4.39034600 0.94308300 0.18260800 |
| A232−4 |
| P −1.60506300 0.24091400 −0.44708900  O −1.38802800 0.76895700 −1.78986700  F −1.75006500 1.39927200 0.62469000  O −3.01176800 −0.45937200 −0.28621900  N −0.54160800 −0.78293400 0.20712900  C 0.72703400 −0.97334700 0.01025100  N 1.63346600 −0.04313800 −0.33459800  C 3.00805400 −0.38843300 −0.69867000  H 3.05179700 −1.43564300 −0.98310600  H 3.25409200 0.18399800 −1.59567100  C 1.34632900 1.39701400 −0.24161200  H 2.18997800 1.90750700 −0.70697100  H 0.47284900 1.64122400 −0.84324300  C 1.22402200 −2.38503200 0.22832800  H 1.36265600 −2.88025400 −0.73536700  H 2.16545600 −2.42206400 0.77531500  H 0.45672700 −2.92251600 0.77621200  C 4.01541000 −0.08903100 0.40464900  H 3.76243300 −0.62787500 1.31929000  H 5.01546900 −0.38909000 0.08940400  H 4.04154000 0.97670100 0.63444300  C 1.19219000 1.85630100 1.20431800  H 0.91442900 2.91051600 1.22698500  H 0.40796700 1.29512400 1.70944800  H 2.12420300 1.72876600 1.75622400  C −3.39878900 −0.99611700 0.97736000  H −3.43042900 −0.20747900 1.73020400  H −4.39287800 −1.41373300 0.84179600  H −2.69988000 −1.77464400 1.28238800 |
| A232−5 |
| P 1.92254500 0.32129700 −0.22150000  O 2.32896800 1.46634300 0.59707100  F 2.48324600 0.37750500 −1.67466700  O 2.58671500 −1.05480300 0.20902900  N 0.35611000 −0.02405100 −0.46165900  C −0.69401000 0.66335700 −0.13164900  N −1.89881800 0.13591800 −0.42861700  C −1.96616900 −1.18989900 −1.05131800  H −2.97062000 −1.29139200 −1.46520200  H −1.25334100 −1.21444200 −1.87542500  C −3.16905800 0.80425000 −0.17045700  H −2.99248200 1.86100300 0.00535800  H −3.76739100 0.73670300 −1.08256600  C −0.66916100 2.00927400 0.55254300  H −1.00763600 2.78236400 −0.14005700  H −1.33271600 2.01421400 1.41761400  H 0.34336900 2.24062900 0.86672900  C −1.67356200 −2.33045300 −0.08335700  H −2.35765700 −2.31476000 0.76577900  H −1.78561200 −3.28638400 −0.59695500  H −0.65125900 −2.25639300 0.28323100  C −3.93415500 0.19463600 0.99839200  H −4.86260300 0.74180400 1.16651000  H −4.18832700 −0.84720700 0.79988100  H −3.33672700 0.23292500 1.91079100  C 2.56534700 −1.36923700 1.60055500  H 1.53806700 −1.52886700 1.93755000  H 3.13228400 −2.28856800 1.71944200  H 3.02336500 −0.56558200 2.17670000 |
| A232−6 |
| P 1.90019600 −0.09357000 0.33811900  O 2.29765700 −1.47689800 0.61200500  F 2.30492000 0.89292000 1.47767700  O 2.70989200 0.59982200 −0.83591200  N 0.34717200 0.29084700 0.06705300  C −0.71294600 −0.42432600 0.29412400  N −1.90748100 0.10989900 −0.02398500  C −1.97183000 1.44788100 −0.61397100  H −1.13562300 1.55889400 −1.30097600  H −2.89735700 1.49998100 −1.19042100  C −3.18714200 −0.53739400 0.24415200  H −3.09066500 −1.20445800 1.09763400  H −3.88571200 0.24712800 0.54406800  C −0.71557700 −1.80474400 0.90528400  H −1.04174200 −1.73683600 1.94580100  H −1.39914300 −2.46899800 0.37858200  H 0.28866300 −2.21600300 0.88423900  C −1.93282800 2.53913900 0.44729400  H −0.99158900 2.48865700 0.99350300  H −2.01344300 3.52234700 −0.01769900  H −2.75810100 2.42734700 1.15329700  C −3.73010200 −1.28600400 −0.96754800  H −3.05735800 −2.09409600 −1.25717100  H −4.70856500 −1.71333500 −0.74486500  H −3.83716700 −0.61515500 −1.82102400  C 2.83682000 −0.13460800 −2.05277200  H 1.86387700 −0.22685200 −2.54065200  H 3.50964200 0.43392000 −2.68897300  H 3.25046300 −1.12353800 −1.85582000 |
| A232−7 |
| P 1.79462600 −0.16515300 0.21560400  O 2.21660600 −1.52664600 0.55746900  F 2.29371000 0.84815500 1.31196500  O 2.48314300 0.42888300 −1.08275900  N 0.25255500 0.26485200 0.01936400  C −0.81405400 −0.44808000 0.21605800  N −2.00445900 0.12170600 −0.04686400  C −2.05666100 1.49252400 −0.55802600  H −1.22840200 1.63008700 −1.24990300  H −2.98911500 1.59063500 −1.11697100  C −3.28900300 −0.52417500 0.19981900  H −3.18861700 −1.24667900 1.00650000  H −3.97132400 0.24865800 0.56131300  C −0.82541100 −1.86542100 0.73569100  H −1.14598600 −1.86437900 1.77999000  H −1.51718000 −2.48665900 0.16866800  H 0.17491400 −2.28389300 0.68522400  C −1.98755700 2.51954800 0.56404600  H −1.03982000 2.42512500 1.09299200  H −2.06040800 3.52909000 0.15792900  H −2.80454200 2.37823000 1.27441900  C −3.86131900 −1.18527500 −1.04872900  H −3.20529100 −1.98222000 −1.40077700  H −4.84259700 −1.61221500 −0.83794900  H −3.97115700 −0.45944900 −1.85553500  C 3.87403900 0.14481500 −1.26638000  H 4.05319700 −0.92693600 −1.19015700  H 4.13179800 0.50364100 −2.25901500  H 4.46688700 0.67343600 −0.51852200 |
| A232−8 |
| P −1.80058300 0.30313800 0.15994900  O −2.23173800 1.57410800 −0.42897200  F −2.43289200 0.12121300 1.58805400  O −2.35362200 −0.98391100 −0.58484700  N −0.25590000 −0.08468700 0.41031000  C 0.80911100 0.61684300 0.17261100  N 2.00058000 0.03589700 0.41098900  C 2.03875400 −1.36464000 0.84505800  H 3.03813600 −1.54118800 1.24583700  H 1.31988000 −1.48837400 1.65488200  C 3.28342700 0.71064700 0.25151000  H 3.12499000 1.78447500 0.22045200  H 3.87416900 0.50947400 1.14871300  C 0.80998400 2.03632900 −0.34230800  H 1.14567500 2.71503300 0.44416200  H 1.48624900 2.13751500 −1.19129100  H −0.19501700 2.31928100 −0.63908500  C 1.73084000 −2.35200900 −0.27474900  H 2.43004400 −2.23893700 −1.10400200  H 1.80986000 −3.37221200 0.10366100  H 0.71761400 −2.19933700 −0.64266300  C 4.04566800 0.25175800 −0.98603600  H 4.98342000 0.80155000 −1.07571500  H 4.28237800 −0.81129100 −0.92854900  H 3.45391800 0.42146800 −1.88705000  C −3.71123800 −0.93811200 −1.03682700  H −3.87866300 −0.04450000 −1.63682500  H −3.86605200 −1.83198900 −1.63480200  H −4.39098000 −0.94390600 −0.18354900 |
| A234−1 |
| P 1.48926500 0.91237300 −0.08144800  O 1.54709900 2.36739300 0.01379400  F 2.10815000 0.37958800 −1.43202800  O 2.41805400 0.19679400 0.97541500  N 0.08637500 0.09492100 −0.04555500  C −1.12205300 0.55742800 −0.12477600  N −2.13889100 −0.32286800 −0.03264600  C −1.85146800 −1.74113000 0.17891200  H −1.01904600 −1.82037600 0.87611800  H −2.73165100 −2.18068500 0.65159100  C −3.54481300 0.03782300 −0.17803800  H −3.63389500 0.92164300 −0.80545100  H −4.03022700 −0.77585400 −0.72214900  C 2.65313500 −1.22009200 0.91347000  H 1.86100700 −1.69862000 0.33395000  H 2.58594400 −1.57536800 1.94161200  C 4.02008600 −1.49297200 0.32504200  H 4.21909500 −2.56603900 0.32887700  H 4.07200100 −1.13055000 −0.70113600  H 4.79027700 −0.99311400 0.91221300  C −1.48058200 2.00916000 −0.32969400  H −1.80717400 2.15835600 −1.36144000  H −2.29237100 2.31136200 0.33042100  H −0.60882000 2.63033000 −0.14897800  C −1.52439000 −2.45938900 −1.12369000  H −0.63272300 −2.02343600 −1.57341200  H −1.34140000 −3.51851300 −0.93743700  H −2.35070500 −2.37678000 −1.83234300  C −4.23575600 0.25670500 1.16293300  H −3.78136200 1.08927400 1.70120500  H −5.29395400 0.47583500 1.01525500  H −4.15590200 −0.63242900 1.78984700 |
| A234−2 |
| P −1.24160200 0.20251100 −0.67306600  O −0.89680600 0.71293700 −1.99624800  F −1.49238600 1.37640800 0.36517800  O −2.64905600 −0.50757200 −0.64049900  N −0.23270600 −0.79616000 0.09900200  C 1.04979200 −0.97597300 0.01859300  N 1.97561500 −0.04382100 −0.26654300  C 3.37842900 −0.38387000 −0.50628600  H 3.45362500 −1.43616900 −0.76412000  H 3.69511700 0.17248100 −1.39122400  C 1.67188000 1.39517100 −0.22585900  H 2.54931600 1.90427400 −0.62569400  H 0.85215000 1.62114000 −0.90503800  C −3.21453900 −0.97283800 0.59559900  H −2.42170400 −1.10479300 1.33426800  H −3.63773400 −1.95244600 0.37495900  C −4.27839700 −0.01015200 1.07707600  H −4.74128200 −0.39427400 1.98772900  H −3.84321800 0.96571100 1.28986000  H −5.05077100 0.10968300 0.31748200  C 1.53847600 −2.37713900 0.31197100  H 1.76432000 −2.89236700 −0.62437200  H 2.42941400 −2.39192400 0.93884600  H 0.73085900 −2.91005300 0.80340500  C 4.28747100 −0.05316900 0.67101700  H 3.96369600 −0.57682600 1.57203100  H 5.31292600 −0.34903400 0.44631400  H 4.28525300 1.01703300 0.88070100  C 1.38966400 1.87925500 1.19236600  H 1.11103200 2.93332300 1.17190500  H 0.56355700 1.32550100 1.63499400  H 2.26882700 1.76264400 1.82724900 |
| A234−3 |
| P 1.43572300 0.72199700 −0.25789400  O 1.75688100 1.87737000 0.58507900  F 1.95566400 0.95538500 −1.72358600  O 2.20365200 −0.60955200 0.13155200  N −0.06806100 0.19255700 −0.50472200  C −1.18390300 0.66552200 −0.04303700  N −2.31381800 −0.00285000 −0.34608200  C −2.22173100 −1.25292800 −1.10748800  H −3.22435500 −1.46691000 −1.48109400  H −1.56852600 −1.08146600 −1.96303000  C −3.64934000 0.43280700 0.04459200  H −3.61816600 1.47977600 0.33096400  H −4.28733100 0.37147800 −0.84070900  C 3.58773800 −0.48581600 0.50873600  H 3.66820300 0.25755000 1.30290100  H 4.15531600 −0.13390800 −0.35630900  C 4.06418200 −1.84637300 0.95803800  H 5.11410800 −1.79463400 1.24884500  H 3.48167900 −2.18850900 1.81347700  H 3.96006400 −2.57302900 0.15240600  C −1.31004100 1.91168800 0.80038300  H −1.79687900 2.70098000 0.22431100  H −1.91272700 1.71641500 1.68758300  H −0.32384700 2.25576500 1.09556900  C −1.69806100 −2.42419300 −0.28431800  H −2.32856600 −2.60796500 0.58632500  H −1.68730900 −3.32714300 −0.89658000  H −0.68175600 −2.22359800 0.05065600  C −4.24179200 −0.40628100 1.17071000  H −5.22813000 −0.02826100 1.44263100  H −4.35185300 −1.44791000 0.86684100  H −3.60017600 −0.37409400 2.05279600 |
| A234−4 |
| P 1.43193000 0.38932000 −0.52398200  O 1.74921200 1.81672900 −0.62862000  F 1.81955200 −0.35066000 −1.85840000  O 2.30686800 −0.40526600 0.53092400  N −0.05503800 −0.16997700 −0.23917600  C −1.16342300 0.49806100 −0.14613600  N −2.28839000 −0.19078700 0.12275800  C −2.22313700 −1.64007000 0.31796200  H −1.30824900 −1.87016000 0.85990700  H −3.07171800 −1.91736400 0.94610600  C −3.61719700 0.40828300 0.17790500  H −3.64814300 1.29375600 −0.45274900  H −4.31071800 −0.31004900 −0.26552900  C 3.70685400 −0.07760400 0.60589600  H 3.80666700 0.99929000 0.74767200  H 4.18059900 −0.34956700 −0.34064100  C 4.29853700 −0.85590900 1.75665000  H 5.36345900 −0.63712600 1.84421700  H 3.80790300 −0.58231400 2.69071900  H 4.17338500 −1.92691300 1.59803100  C −1.29502600 1.99048100 −0.33256800  H −1.75068600 2.19446300 −1.30418600  H −1.92798400 2.42824300 0.43806900  H −0.31276900 2.45195500 −0.31217300  C −2.26021800 −2.39337700 −1.00488600  H −1.39197700 −2.12599400 −1.60618200  H −2.24331400 −3.46945300 −0.82816300  H −3.16594100 −2.15437400 −1.56578600  C −4.04995300 0.74390200 1.60045700  H −3.38374800 1.48502000 2.04351600  H −5.06487600 1.14335300 1.60577500  H −4.03008200 −0.14557600 2.23153300 |
| A234−5 |
| P 1.46545100 0.40045700 −0.21716300  O 1.86780500 1.67403300 0.38894800  F 2.05129700 0.27451800 −1.67012600  O 2.08528200 −0.87534300 0.48979700  N −0.07250600 −0.03296900 −0.44208400  C −1.15555400 0.62407400 −0.16855600  N −2.33198900 0.00629800 −0.39462000  C −2.32991700 −1.38409100 −0.86072100  H −3.33072100 −1.58727300 −1.24515800  H −1.62450500 −1.46356100 −1.68785600  C −3.63380000 0.63130900 −0.19398300  H −3.51194000 1.70883400 −0.13688800  H −4.23490400 0.43331200 −1.08511100  C 3.34747100 −0.73208400 1.16903700  H 3.45538700 −1.65159500 1.74196700  H 3.27849600 0.11269900 1.85531600  C 4.49273500 −0.55052800 0.19520600  H 5.43854700 −0.54399200 0.73929400  H 4.51015100 −1.36416300 −0.53029200  H 4.39773400 0.39558700 −0.33866000  C −1.19503700 2.03197900 0.37637400  H −1.57712600 2.71381600 −0.38578000  H −1.85109000 2.09025400 1.24514600  H −0.19341200 2.34612500 0.65259300  C −1.96331900 −2.38574700 0.22837000  H −2.64789900 −2.31700200 1.07453200  H −2.01439000 −3.39911600 −0.17260100  H −0.94835700 −2.20544600 0.57860100  C −4.35617800 0.11437100 1.04465700  H −5.31092300 0.62786900 1.16519100  H −4.55615000 −0.95469600 0.96388300  H −3.75398300 0.28231300 1.93905600 |
| A234−6 |
| P −1.48419600 0.12045500 −0.50259300  O −1.95557300 −1.11251200 −1.14243700  F −1.78972300 1.35462900 −1.43110000  O −2.26709300 0.52283600 0.81254100  N 0.05445800 0.37059400 −0.08538300  C 1.09022600 −0.36619600 −0.34552700  N 2.28081400 0.03434600 0.13847800  C 2.36356000 1.25268000 0.94573200  H 1.47978300 1.30302300 1.57826000  H 3.23951500 1.15187300 1.58923400  C 3.54242500 −0.64388000 −0.13733200  H 3.48036500 −1.16310300 −1.09095800  H 4.30278400 0.13133500 −0.25754300  C −3.68825000 0.27923600 0.84115200  H −4.09815500 0.37574400 −0.16638000  H −4.10059700 1.07616800 1.45858600  C −3.98236700 −1.08685400 1.41942900  H −5.06124300 −1.23631200 1.48859900  H −3.55841100 −1.85933900 0.77887200  H −3.55444200 −1.17288300 2.41868300  C 1.06857900 −1.63247800 −1.16735600  H 1.49178200 −1.42967400 −2.15382900  H 1.66128900 −2.41654500 −0.69830200  H 0.04477000 −1.96952200 −1.29624800  C 2.46866100 2.50106500 0.08018400  H 1.57463600 2.60014200 −0.53446100  H 2.56062600 3.38912000 0.70655500  H 3.34274300 2.45230900 −0.57228300  C 3.94615100 −1.60483900 0.97491600  H 3.21112800 −2.40324700 1.08291000  H 4.91598600 −2.05393800 0.75726100  H 4.01840900 −1.08320700 1.93016300 |
| A234−7 |
| P 1.43568300 −0.72193500 0.25840000  O 1.75698000 −1.87836700 −0.58306500  F 1.95599000 −0.95315200 1.72418800  O 2.20310600 0.60936300 −0.13307500  N −0.06820800 −0.19253100 0.50495300  C −1.18399500 −0.66552900 0.04318800  N −2.31399600 0.00261400 0.34649600  C −2.22195100 1.25258500 1.10808200  H −3.22459000 1.46650600 1.48168400  H −1.56881600 1.08096600 1.96364300  C −3.64936000 −0.43265700 −0.04515200  H −3.61838200 −1.47981700 −0.33088500  H −4.28805900 −0.37061700 0.83957600  C 3.58738100 0.48546700 −0.50951600  H 3.66824300 −0.25816800 −1.30339300  H 4.15456000 0.13383100 0.35590300  C 4.06409000 1.84585800 −0.95904900  H 5.11412000 1.79395800 −1.24944900  H 3.48192400 2.18776800 −1.81481000  H 3.95971600 2.57277000 −0.15368200  C −1.31014900 −1.91146700 −0.80060600  H −1.79713900 −2.70087100 −0.22482600  H −1.91268400 −1.71582600 −1.68782300  H −0.32394600 −2.25557000 −1.09573300  C −1.69816300 2.42400400 0.28521300  H −2.32865200 2.60818800 −0.58534700  H −1.68725400 3.32675900 0.89776800  H −0.68188200 2.22340200 −0.04983700  C −4.24054000 0.40608900 −1.17218000  H −3.59804500 0.37351800 −2.05361400  H −5.22665100 0.02810600 −1.44498900  H −4.35076100 1.44783200 −0.86877600 |
| A234−8 |
| P −1.46960800 −0.64567200 0.23356800  O −1.95489600 −0.26003000 1.56075400  F −1.86964800 −2.12368700 −0.09925200  O −2.14914500 0.14363200 −0.96637600  N 0.11094200 −0.65563300 −0.11565200  C 0.94322000 0.27035300 0.25141100  N 2.24364600 0.11237200 −0.05508700  C 2.67283800 −1.07452400 −0.79639800  H 1.90808900 −1.30844700 −1.53389700  H 3.58911200 −0.80704200 −1.32624200  C 3.30080900 1.01795700 0.38171200  H 3.00693200 1.50778800 1.30764000  H 4.16878800 0.40082700 0.62474200  C −3.55508300 0.44735600 −0.85904300  H −4.05816900 −0.33800300 −0.29133100  H −3.93090600 0.42895600 −1.88120000  C −3.75689600 1.79982100 −0.21244400  H −4.81919300 2.04891600 −0.19137800  H −3.37977800 1.78120800 0.80985200  H −3.23182200 2.57000700 −0.77907200  C 0.55573700 1.52080400 1.00689600  H 0.75061200 1.37777000 2.07188500  H 1.11373200 2.38826100 0.66027000  H −0.50786600 1.71097900 0.89748700  C 2.90618300 −2.26611600 0.12239900  H 1.97587400 −2.53558000 0.62088300  H 3.25394200 −3.12462700 −0.45327400  H 3.65881400 −2.03608200 0.87915500  C 3.67104700 2.04431100 −0.68250400  H 2.82226400 2.68967500 −0.91197200  H 4.49786000 2.66767200 −0.33981700  H 3.97574100 1.55022600 −1.60600900 |
| A234−9 |
| P 1.52779900 0.64921800 −0.51697500  O 1.81411700 1.97017200 0.04877700  F 1.78213000 0.58091400 −2.05588100  O 2.54482300 −0.48653800 −0.08408900  N 0.05551600 −0.01929800 −0.37096700  C −1.05540700 0.54629400 −0.00789200  N −2.16080200 −0.22200900 0.04324500  C −2.07439900 −1.64323900 −0.29534600  H −1.13424900 −2.03000900 0.09215600  H −2.89169800 −2.14709800 0.22407700  C −3.49211100 0.28500100 0.35718300  H −3.56165000 1.33411900 0.07922200  H −4.19743500 −0.25030700 −0.28269800  C 2.83009500 −0.61252100 1.31673700  H 1.91407900 −0.91199600 1.83519900  H 3.14818100 0.35917300 1.69879700  C 3.91023600 −1.65599200 1.47601800  H 4.15230900 −1.78347800 2.53184900  H 3.57795700 −2.61300900 1.07406200  H 4.81116900 −1.34945400 0.94502800  C −1.21351100 2.00522800 0.34663300  H −1.70373100 2.52545900 −0.47959300  H −1.82706700 2.13070600 1.23760400  H −0.23667600 2.45120300 0.50447200  C −2.16265000 −1.87580200 −1.79763500  H −1.32461800 −1.38951500 −2.29570600  H −2.12720500 −2.94327400 −2.01835200  H −3.09479800 −1.47532300 −2.20105000  C −3.86299500 0.08937000 1.82268400  H −3.18664700 0.64742500 2.47127700  H −4.88158400 0.43283100 2.00745900  H −3.80255000 −0.96390200 2.10002600 |
| A234−10 |
| P −1.73775600 −0.64827300 −0.27842400  O −2.21916300 −1.30858800 0.93903300  F −2.20755700 −1.36454700 −1.58190800  O −2.38740300 0.77820400 −0.53069900  N −0.15765300 −0.45375400 −0.57625600  C 0.86058600 −0.73138000 0.17457500  N 2.08296200 −0.38827400 −0.28224100  C 2.19206000 0.33173500 −1.55451400  H 3.23482700 0.25934400 −1.86771300  H 1.57830900 −0.18548200 −2.29203000  C 3.32812700 −0.67506300 0.41929800  H 3.15058500 −1.43377700 1.17547700  H 4.02122500 −1.11300700 −0.30366000  C −2.89781400 1.50536700 0.59764600  H −3.54905800 0.84896200 1.17538600  H −3.49181200 2.30925500 0.16601500  C −1.77414300 2.05110500 1.45524500  H −2.18230600 2.67096500 2.25503900  H −1.21271300 1.23391600 1.91292400  H −1.09430600 2.65628100 0.85422100  C 0.78265900 −1.40994400 1.52183600  H 1.22642500 −2.40549300 1.46346900  H 1.33019100 −0.83795500 2.27195000  H −0.25565400 −1.51338100 1.82060800  C 1.76407400 1.79205600 −1.46096500  H 2.34882600 2.32754500 −0.71202100  H 1.91056300 2.28110000 −2.42514700  H 0.70895100 1.85617300 −1.20007100  C 3.95263800 0.56352900 1.05150100  H 4.86607300 0.29463200 1.58356800  H 4.21005400 1.30361100 0.29285900  H 3.26042200 1.02516500 1.75772200 |
| A234−11 |
| P 1.46568300 0.40027000 −0.21755100  O 1.86846900 1.67412800 0.38762000  F 2.05109400 0.27322700 −1.67055800  O 2.08536400 −0.87523900 0.49009600  N −0.07248800 −0.03292200 −0.44190200  C −1.15536600 0.62436100 −0.16820900  N −2.33195800 0.00683600 −0.39415600  C −2.33022600 −1.38337900 −0.86071700  H −3.33104700 −1.58616900 −1.24533000  H −1.62474600 −1.46278500 −1.68780100  C −3.63369600 0.63197200 −0.19344400  H −3.51151800 1.70935100 −0.13453800  H −4.23433400 0.43553700 −1.08524500  C 3.34740100 −0.73172900 1.16951000  H 3.45514300 −1.65090400 1.74301700  H 3.27840700 0.11347300 1.85527000  C 4.49284400 −0.55083900 0.19576300  H 5.43855900 −0.54389100 0.74001000  H 4.51040300 −1.36498000 −0.52916400  H 4.39790200 0.39490900 −0.33877000  C −1.19447200 2.03216400 0.37702100  H −1.57739400 2.71419900 −0.38452300  H −1.84968200 2.09005400 1.24646800  H −0.19263200 2.34636800 0.65239100  C −1.96398900 −2.38543700 0.22813500  H −2.64866800 −2.31678100 1.07422700  H −2.01519600 −3.39867900 −0.17313100  H −0.94904400 −2.20541200 0.57856600  C −4.35695600 0.11336400 1.04399100  H −3.75523200 0.27986300 1.93897700  H −5.31164400 0.62691600 1.16470400  H −4.55714400 −0.95553700 0.96154500 |
| A234−12 |
| P 1.49727000 0.43651800 −0.45174000  O 1.96622300 1.60849800 0.29540100  F 1.95638500 0.53623800 −1.95228600  O 2.16284500 −0.93247900 −0.01398200  N −0.05868000 0.03467100 −0.58958100  C −1.10361000 0.65074300 −0.13107800  N −2.30260500 0.06711100 −0.32335600  C −2.36065200 −1.25180400 −0.96215500  H −3.39544100 −1.40423900 −1.27295200  H −1.73698600 −1.22372300 −1.85557000  C −3.57245400 0.65937100 0.08004500  H −3.43384900 1.72078000 0.26265800  H −4.25790000 0.57656800 −0.76720900  C 3.55939700 −0.90869400 0.34555200  H 4.07486100 −0.14029200 −0.23440900  H 3.94618200 −1.88232800 0.04762600  C 3.72313900 −0.67074000 1.83007500  H 4.78029300 −0.71088900 2.09820400  H 3.32798900 0.30968900 2.09359300  H 3.19178900 −1.43708700 2.39535300  C −1.07550600 1.97570900 0.59280100  H −1.51739200 2.75072100 −0.03644600  H −1.64845400 1.92110600 1.51867400  H −0.04890000 2.25068000 0.81340300  C −1.90506100 −2.38491900 −0.04981500  H −2.50561300 −2.42500800 0.85963100  H −2.00667500 −3.33841000 −0.57020400  H −0.85928700 −2.25131900 0.22197300  C −4.17859300 −0.01370900 1.30592300  H −3.49115000 0.03723100 2.15187400  H −5.11090000 0.47961600 1.58368800  H −4.39858100 −1.06337800 1.10791200 |
| A234−13 |
| P 1.49740500 0.43615700 −0.45220500  O 1.96665400 1.60878000 0.29373800  F 1.95636800 0.53431400 −1.95289300  O 2.16267000 −0.93251100 −0.01320300  N −0.05870500 0.03469400 −0.58987800  C −1.10353300 0.65096700 −0.13139200  N −2.30262100 0.06738000 −0.32333100  C −2.36088400 −1.25160400 −0.96203800  H −3.39578000 −1.40398400 −1.27251100  H −1.73749000 −1.22359300 −1.85563800  C −3.57235200 0.65959000 0.08051500  H −3.43364500 1.72092600 0.26344400  H −4.25799500 0.57710700 −0.76662000  C 3.55914700 −0.90874000 0.34666200  H 4.07489300 −0.14089700 −0.23378100  H 3.94581300 −1.88268900 0.04962400  C 3.72248400 −0.66960900 1.83102300  H 4.77956200 −0.70956300 2.09947500  H 3.32727500 0.31103600 2.09363500  H 3.19093700 −1.43548900 2.39674300  C −1.07521200 1.97597800 0.59242900  H −1.51758000 2.75098800 −0.03644800  H −1.64761300 1.92116100 1.51864400  H −0.04852500 2.25112500 0.81239200  C −1.90507000 −2.38467200 −0.04977500  H −2.50542200 −2.42480300 0.85979600  H −2.00673900 −3.33816800 −0.57015100  H −0.85923500 −2.25104500 0.22176600  C −4.17831700 −0.01376300 1.30630600  H −5.11046300 0.47968800 1.58439200  H −4.39856400 −1.06332400 1.10803400  H −3.49069200 0.03679800 2.15213100 |
| A234−14 |
| P −1.49731200 0.43808400 0.44974000  O −1.96576100 1.60705300 −0.30238900  F −1.95501100 0.54511900 1.95027800  O −2.16463100 −0.93225500 0.01895600  N 0.05838500 0.03531600 0.58779800  C 1.10379400 0.65077900 0.12955100  N 2.30252400 0.06711000 0.32327400  C 2.35992500 −1.25098500 0.96387700  H 3.39445200 −1.40309700 1.27569500  H 1.73553500 −1.22163900 1.85673900  C 3.57281700 0.65854900 −0.07999800  H 3.43451000 1.71966700 −0.26449300  H 4.25752200 0.57704300 0.76798300  C −3.56091800 −0.90855300 −0.34144100  H −4.07576800 −0.13665300 0.23440600  H −3.94906200 −1.88021800 −0.03887800  C −3.72363800 −0.67781600 −1.82722100  H −4.78070000 −0.71806400 −2.09568700  H −3.32721900 0.30082400 −2.09544400  H −3.19288300 −1.44758800 −2.38839000  C 1.07645500 1.97500400 −0.59575100  H 1.51856700 2.75056800 0.03262100  H 1.64955900 1.91900800 −1.52146300  H 0.05003800 2.25008400 −0.81705700  C 1.90494800 −2.38523800 0.05264900  H 2.50612900 −2.42647600 −0.85633200  H 2.00617200 −3.33807500 0.57430400  H 0.85937400 −2.25193300 −0.22003900  C 4.17993500 −0.01662800 −1.30423800  H 5.11242800 0.47628600 −1.58210000  H 4.39982400 −1.06594200 −1.10425300  H 3.49316100 0.03283000 −2.15081800 |
| A234−15 |
| P −1.43586800 −0.72208700 −0.25857000  O −1.75759600 −1.87796900 0.58349300  F −1.95594600 −0.95401300 −1.72438100  O −2.20309400 0.60959300 0.13201600  N 0.06813800 −0.19319700 −0.50518100  C 1.18380200 −0.66582400 −0.04276300  N 2.31384400 0.00225200 −0.34602100  C 2.22197700 1.25178400 −1.10834300  H 3.22471700 1.46551900 −1.48178900  H 1.56910200 1.07968200 −1.96401100  C 3.64919200 −0.43294500 0.04573400  H 3.61794100 −1.47966600 0.33302000  H 4.28763200 −0.37239000 −0.83929500  C −3.58710100 0.48623400 0.50950200  H −3.66772500 −0.25754400 1.30326800  H −4.15509200 0.13505500 −0.35557400  C −4.06288500 1.84673100 0.95970600  H −5.11274200 1.79522500 1.25079800  H −3.47999000 2.18816400 1.81516200  H −3.95873300 2.57380700 0.15445600  C 1.30967900 −1.91122500 0.80187400  H 1.79697400 −2.70097700 0.22684000  H 1.91179300 −1.71493600 1.68924000  H 0.32336000 −2.25516900 1.09680800  C 1.69796200 2.42362800 −0.28623700  H 2.32820500 2.60820500 0.58442300  H 1.68727900 3.32607000 −0.89925200  H 0.68157400 2.22322100 0.04860500  C 4.24098300 0.40715000 1.17143300  H 5.22711300 0.02930100 1.44435800  H 4.35131800 1.44848900 0.86668900  H 3.59879800 0.37580000 2.05314100 |
| A234−16 |
| P 1.43196900 −0.38937500 0.52403000  O 1.74937500 −1.81685300 0.62714000  F 1.81915000 0.34927000 1.85937200  O 2.30712200 0.40641900 −0.52976300  N −0.05501700 0.16999600 0.23950800  C −1.16333100 −0.49808500 0.14604700  N −2.28829400 0.19078500 −0.12288300  C −2.22302000 1.64010500 −0.31778300  H −1.30812900 1.87029500 −0.85967900  H −3.07159400 1.91755800 −0.94587200  C −3.61711100 −0.40826100 −0.17802300  H −3.64799500 −1.29385000 0.45246600  H −4.31061400 0.30997100 0.26560200  C 3.70679300 0.07777500 −0.60618600  H 3.80571700 −0.99910700 −0.74864300  H 4.18156700 0.34888400 0.34008200  C 4.29801300 0.85630400 −1.75702900  H 5.36272500 0.63688600 −1.84557500  H 3.80645100 0.58349400 −2.69084000  H 4.17368000 1.92730800 −1.59776500  C −1.29489200 −1.99053600 0.33224300  H −1.74991000 −2.19463000 1.30413500  H −1.92835600 −2.42816900 −0.43805100  H −0.31265200 −2.45201100 0.31107600  C −2.26014100 2.39319800 1.00519000  H −1.39197200 2.12570500 1.60653300  H −2.24319200 3.46929400 0.82859500  H −3.16593300 2.15416700 1.56596800  C −4.04994200 −0.74363200 −1.60061200  H −3.38386900 −1.48482800 −2.04374200  H −5.06494200 −1.14288400 −1.60597200  H −4.02988600 0.14591300 −2.23158800 |
| A234−17 |
| P 1.46047200 0.20473700 −0.28402900  O 1.85553200 1.57680800 −0.62027600  F 1.91891900 −0.78801600 −1.41579800  O 2.20206200 −0.39124600 0.98129100  N −0.07158600 −0.24601000 −0.04730300  C −1.15438100 0.44379300 −0.22883200  N −2.32952500 −0.14315700 0.06829300  C −2.34532400 −1.50766800 0.59753300  H −1.49949200 −1.62133800 1.27239800  H −3.26315100 −1.61573700 1.17847600  C −3.63009900 0.47728300 −0.15693900  H −3.56100300 1.19083400 −0.97484500  H −4.30728900 −0.31145100 −0.49296800  C 3.50699100 0.12147900 1.31101300  H 3.70787100 −0.26467400 2.30894200  H 3.45254200 1.20993100 1.35119100  C 4.55716400 −0.33497500 0.32019800  H 5.54324200 −0.00991000 0.65580000  H 4.55558200 −1.42191700 0.23501400  H 4.37050700 0.09607700 −0.66394900  C −1.20223400 1.85386700 −0.76635600  H −1.54850100 1.83401100 −1.80221500  H −1.88999300 2.47156200 −0.19052800  H −0.20795800 2.28913200 −0.74494700  C −2.28177100 −2.54810200 −0.51256700  H −1.34825700 −2.44237100 −1.06424100  H −2.32564300 −3.55346000 −0.09209600  H −3.11722700 −2.43178900 −1.20580500  C −4.18573900 1.14498500 1.09573200  H −3.53576100 1.95750100 1.42254800  H −5.17882300 1.55235600 0.90206800  H −4.26460800 0.42803800 1.91403900 |
| A234−18 |
| P −1.20471600 0.51643700 −0.52522900  O −0.86844400 1.10246300 −1.81892200  F −1.26712900 1.61414300 0.61659600  O −2.68443700 −0.03012200 −0.46508500  N −0.28671100 −0.65968500 0.09437800  C 0.96300200 −0.96997700 −0.06625000  N 1.97687800 −0.12042400 −0.30537300  C 3.32312000 −0.58518700 −0.64114600  H 3.27012400 −1.60803600 −1.00226400  H 3.66931800 0.01974100 −1.48203000  C 1.83803400 1.33193300 −0.11541500  H 2.75036800 1.78046700 −0.50970000  H 1.02271900 1.71058500 −0.72894300  C −3.19626700 −0.61011800 0.74340800  H −3.12164600 0.12652200 1.54678500  H −2.58028600 −1.47347200 1.00056500  C −4.63318300 −1.00264900 0.49199800  H −5.05812000 −1.45255600 1.39023100  H −5.22597400 −0.12846100 0.22400200  H −4.69117500 −1.72461700 −0.32233200  C 1.30032000 −2.43855000 0.06559200  H 1.43286500 −2.87701800 −0.92605100  H 2.20572100 −2.61399400 0.64558300  H 0.45612400 −2.92787200 0.54067800  C 4.30437400 −0.47546100 0.51940900  H 3.95486800 −1.04812800 1.38011000  H 5.28124200 −0.85766400 0.22071100  H 4.43055400 0.56218000 0.83022000  C 1.66813500 1.70221100 1.35408000  H 1.50360400 2.77631400 1.44523400  H 0.80617800 1.19645100 1.78567800  H 2.55509500 1.43454600 1.92955900 |
| A234−19 |
| P −1.56674500 −0.87831500 −0.26133100  O −1.85590300 −1.82734700 0.81674800  F −1.99169900 −1.40309400 −1.66643800  O −2.46401800 0.43013200 −0.24171600  N −0.05612700 −0.34835600 −0.52771900  C 1.05342100 −0.72059800 0.03196600  N 2.18280300 −0.09045100 −0.35116600  C 2.09671300 1.00410000 −1.32285800  H 3.10668000 1.16696300 −1.70227300  H 1.46991800 0.67427300 −2.15127200  C 3.51286500 −0.42522000 0.14402600  H 3.49061700 −1.40866300 0.60360000  H 4.17636300 −0.50168500 −0.72108600  C −2.57246100 1.12512400 1.00860300  H −1.57595800 1.46165200 1.31338300  H −2.95207000 0.43354800 1.76275000  C −3.50111900 2.29869500 0.80567000  H −3.60891600 2.85484300 1.73767700  H −3.10774100 2.96803400 0.04062700  H −4.48434600 1.95021900 0.49034900  C 1.17810700 −1.80682200 1.07357900  H 1.69502900 −2.66928700 0.64825700  H 1.75306800 −1.45490400 1.93046900  H 0.19001900 −2.11723000 1.39734100  C 1.53647300 2.29439300 −0.73539700  H 2.13151600 2.63428900 0.11295600  H 1.54295100 3.07748200 −1.49495300  H 0.50902700 2.14137700 −0.40951100  C 4.05776500 0.60524300 1.12617300  H 5.04124700 0.29963300 1.48543200  H 4.16070500 1.58215000 0.65226400  H 3.39041300 0.70976200 1.98326800 |
| A234−20 |
| P −1.19732200 0.19845300 −0.19176200  O −1.14336200 0.54986200 −1.61369200  F −1.22222300 1.51988500 0.68456200  O −2.54828600 −0.47546200 0.28318000  N −0.08538500 −0.75318300 0.46150200  C 1.15505100 −0.99634300 0.17445800  N 2.03909100 −0.13087600 −0.35119500  C 3.37775100 −0.54065800 −0.77624300  H 3.39057000 −1.61504700 −0.93370900  H 3.55702900 −0.08456400 −1.75235700  C 1.76779400 1.31361100 −0.41250600  H 2.56691900 1.74998800 −1.01254100  H 0.84343700 1.49423700 −0.95855000  C −3.77281100 0.08653700 −0.22241500  H −3.71949900 0.11650300 −1.31163800  H −3.86968200 1.10925200 0.15062500  C −4.90892500 −0.78212800 0.26236900  H −5.85918200 −0.38645300 −0.09829700  H −4.79095900 −1.80037000 −0.10791800  H −4.93134100 −0.80824700 1.35171500  C 1.64885200 −2.38685500 0.50599100  H 1.71273900 −2.98337500 −0.40685800  H 2.62709000 −2.38338300 0.98589500  H 0.91601200 −2.84647100 1.16108500  C 4.47179500 −0.12901300 0.20119800  H 4.28498700 −0.54660600 1.19193200  H 5.44058000 −0.48625700 −0.15016700  H 4.52785400 0.95601900 0.29461400  C 1.74609700 1.95138100 0.97263700  H 1.47503500 3.00417600 0.88779400  H 1.00843300 1.46905500 1.61156600  H 2.72343100 1.87980300 1.45120800 |
| A234−21 |
| P −1.20932400 0.58089900 −0.20386400  O −1.08650000 0.78241000 −1.64940400  F −1.02645400 1.91502900 0.60358000  O −2.66658700 0.24948100 0.31737900  N −0.25330700 −0.52275100 0.48511900  C 0.93887200 −0.94622900 0.19736000  N 1.93903600 −0.22526800 −0.33858300  C 3.20355500 −0.83057600 −0.75767700  H 3.06009000 −1.89692500 −0.90461200  H 3.44715400 −0.41541600 −1.73805600  C 1.88140000 1.24185600 −0.42210500  H 2.73853100 1.54828700 −1.02246500  H 0.99731000 1.54633000 −0.97961800  C −3.31951800 −0.89686200 −0.24787300  H −2.74810800 −1.78866700 0.02322900  H −3.32452400 −0.79669400 −1.33530200  C −4.72158800 −0.95173600 0.31090100  H −5.24996800 −1.81577500 −0.09395500  H −4.69464100 −1.03472700 1.39729800  H −5.27083600 −0.04915300 0.04393000  C 1.22696700 −2.39100300 0.54032300  H 1.20460900 −2.99869200 −0.36714900  H 2.19551700 −2.52508700 1.02115400  H 0.43739600 −2.73476600 1.20062900  C 4.34584400 −0.57255700 0.21718400  H 4.10121400 −0.95005700 1.21143200  H 5.25308800 −1.06862800 −0.12984300  H 4.55748400 0.49389300 0.30108800  C 1.94728900 1.89848500 0.95275300  H 1.82699200 2.97740600 0.85063000  H 1.14954300 1.53468800 1.59788500  H 2.90478100 1.69797000 1.43472400 |
| A234−22 |
| P −1.32148300 −0.76767300 0.41490400  O −1.45008000 −0.39622700 1.82429200  F −2.31421300 −1.93551000 0.07949900  O −1.81323900 0.35158300 −0.60947800  N 0.04789700 −1.32681500 −0.21042200  C 1.18446400 −0.84980700 −0.59520200  N 1.70277200 0.35780000 −0.29650300  C 2.92774600 0.85902200 −0.91950600  H 3.10474800 0.32034900 −1.84562800  H 2.74307100 1.89874700 −1.19967300  C 1.15136100 1.19076800 0.78086200  H 1.62341400 2.16945700 0.68402100  H 0.08579300 1.34579800 0.62388700  C −3.05850600 1.01316700 −0.31510100  H −3.02147800 1.38812200 0.70923600  H −3.86610700 0.28113400 −0.38836000  C −3.23713300 2.12844400 −1.31753900  H −4.17736100 2.64878300 −1.13110400  H −2.42066400 2.84661200 −1.23775200  H −3.25327900 1.73088700 −2.33221800  C 2.01452900 −1.76564700 −1.46823400  H 1.95117100 −1.44654000 −2.51078200  H 3.06409500 −1.78291900 −1.17582300  H 1.59253900 −2.76228700 −1.39144400  C 4.14909000 0.77622500 −0.01176000  H 4.31935300 −0.24994400 0.31796800  H 5.03504900 1.12209600 −0.54585000  H 4.02482000 1.40101400 0.87307100  C 1.41642300 0.60788500 2.16358200  H 0.93500700 1.22874200 2.91931700  H 0.98755700 −0.38834700 2.24998100  H 2.48645100 0.56289700 2.36946200 |
| A234−23 |
| P 1.48423100 0.12013000 0.50271100  O 1.95561200 −1.11332300 1.14167400  F 1.78963100 1.35365500 1.43215600  O 2.26712200 0.52349700 −0.81198600  N −0.05446000 0.37048600 0.08567400  C −1.09032200 −0.36617800 0.34575000  N −2.28081400 0.03445800 −0.13845400  C −2.36329500 1.25286500 −0.94562900  H −1.47974600 1.30283300 −1.57851000  H −3.23954200 1.15248700 −1.58880800  C −3.54250200 −0.64369500 0.13712900  H −3.48049500 −1.16318800 1.09059900  H −4.30284500 0.13151300 0.25755300  C 3.68812800 0.27950200 −0.84133800  H 4.09854800 0.37557200 0.16603800  H 4.10040300 1.07653600 −1.45868400  C 3.98173200 −1.08644700 −1.42026300  H 5.06055600 −1.23606800 −1.48987200  H 3.55783200 −1.85910100 −0.77988000  H 3.55344600 −1.17200500 −2.41940700  C −1.06886500 −1.63251500 1.16750600  H −1.49240000 −1.42982500 2.15384800  H −1.66137600 −2.41656700 0.69814700  H −0.04506700 −1.96952000 1.29660000  C −2.46771800 2.50130300 −0.08008300  H −1.57356500 2.60020400 0.53438500  H −2.55957900 3.38930800 −0.70654500  H −3.34171300 2.45284100 0.57252400  C −3.94617100 −1.60435900 −0.97541100  H −3.21117500 −2.40278100 −1.08350700  H −4.91606800 −2.05345600 −0.75802800  H −4.01823900 −1.08248000 −1.93053600 |
| A234−24 |
| P 1.48334000 −0.99071500 0.11732400  O 1.53179600 −2.38469800 −0.31134400  F 1.94861100 −0.81774700 1.61466200  O 2.54264800 −0.08715600 −0.62880000  N 0.10683000 −0.12792500 0.10433900  C −1.11254900 −0.56822100 0.06090600  N −2.10845900 0.33866800 0.02070700  C −1.78865000 1.76528400 −0.01009500  H −0.92040700 1.90591600 −0.65159700  H −2.63620300 2.27436100 −0.47253700  C −3.52569100 −0.00587900 0.05501900  H −3.66030600 −0.95230300 0.57368300  H −4.02495200 0.75188100 0.66346900  C 2.70007700 1.29491100 −0.27476700  H 2.87633100 1.36673000 0.80079200  H 1.77407300 1.82221200 −0.51149800  C 3.86950400 1.84025500 −1.06020400  H 4.01770100 2.89451600 −0.82265700  H 4.78013800 1.29446300 −0.81485800  H 3.68522100 1.74468000 −2.13012000  C −1.50393200 −2.02596400 0.07142200  H −1.86518800 −2.29472400 1.06690400  H −2.29872900 −2.22848100 −0.64470600  H −0.63778600 −2.63787400 −0.16180300  C −1.51858300 2.32330800 1.38091700  H −0.65565500 1.82314000 1.81938500  H −1.31398000 3.39342200 1.32703800  H −2.38049300 2.17463500 2.03423000  C −4.15091800 −0.05766100 −1.33412300  H −3.68211200 −0.83291600 −1.94123400  H −5.21830100 −0.27064700 −1.26392900  H −4.02666100 0.89488300 −1.85101900 |
