# Supplementary material for: Precisely predicting the 1H and 13C NMR chemical shifts in new types of nerve agents and building spectra database
Source: Sci Rep. 2022 Nov 24;12:20288. doi: 10.1038/s41598-022-24647-y (PMC9700684; doi:10.1038/s41598-022-24647-y)
Supplement: Supplementary file 3 — Supplementary Information 3. [file 41598_2022_24647_MOESM3_ESM.pdf]

SI2. Each Novichok candidate structure and averaged ^{1}H and ^{13}C NMR chemical shift.

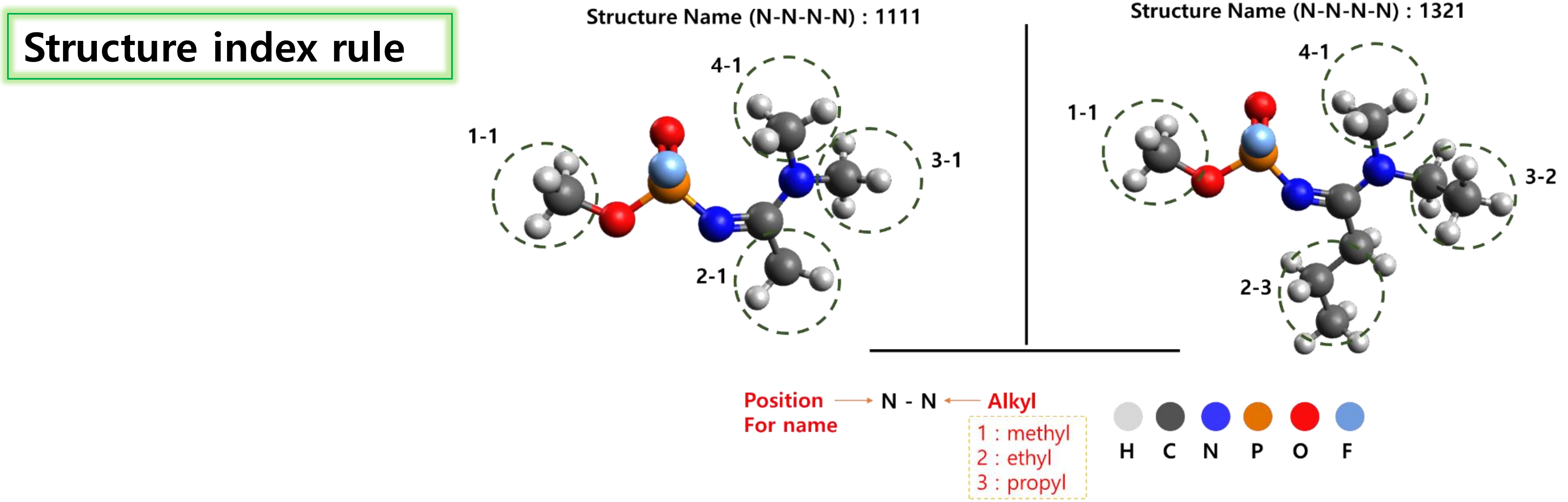

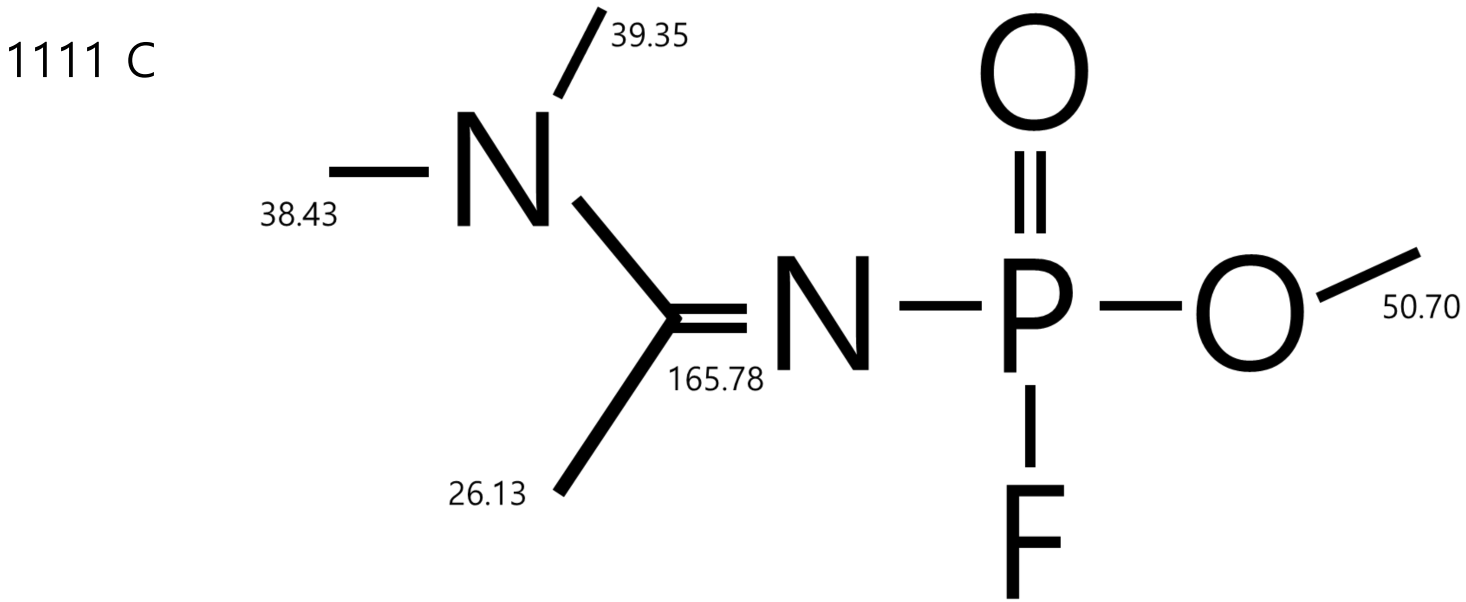

Figure S1. Structural index rule (upper figure) structure 1111 and its ^{13}C chemical shift (lower figure)

1112 C

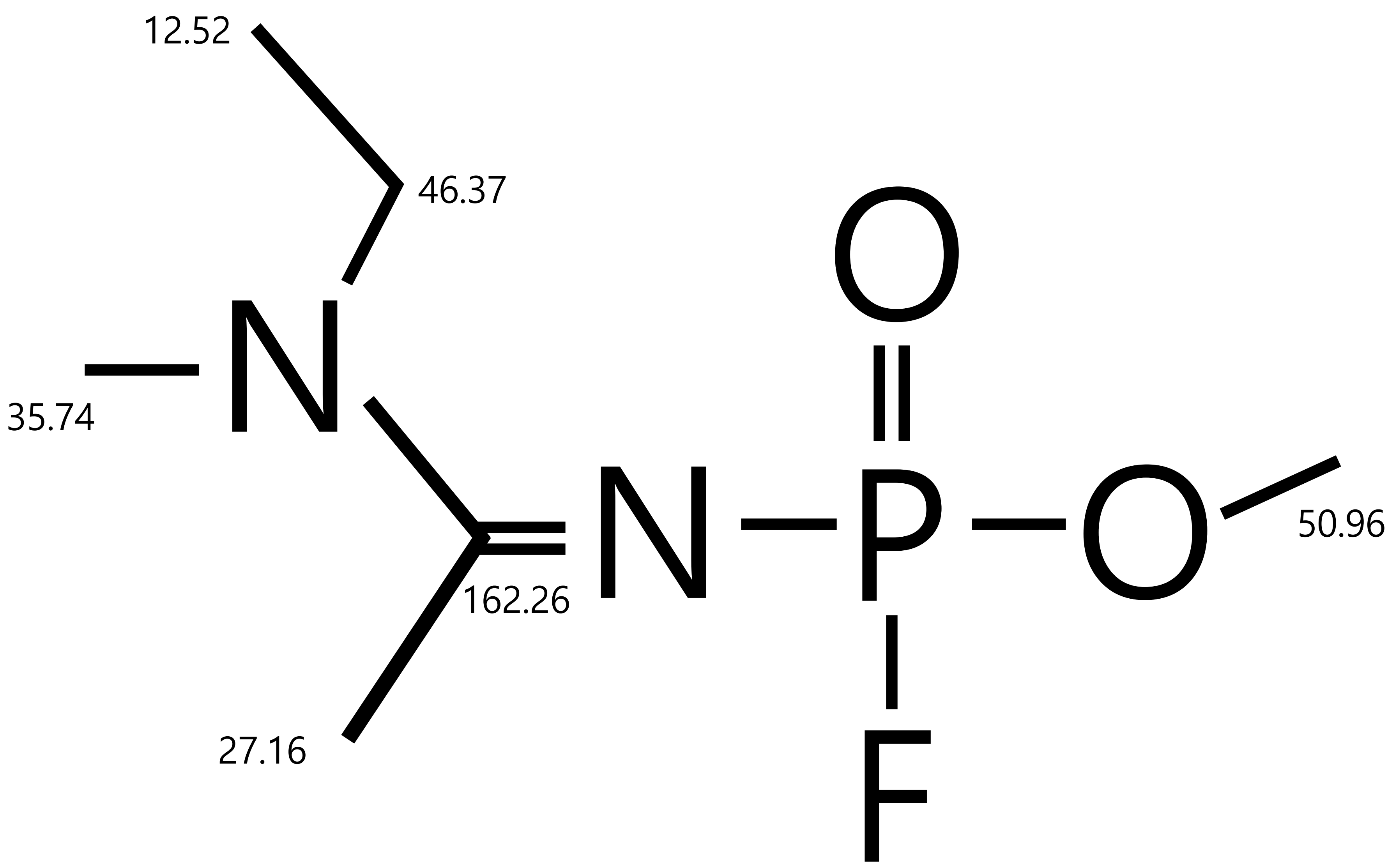

Figure S2. Structure 1112 and its ^{13}C chemical shift

1113 C

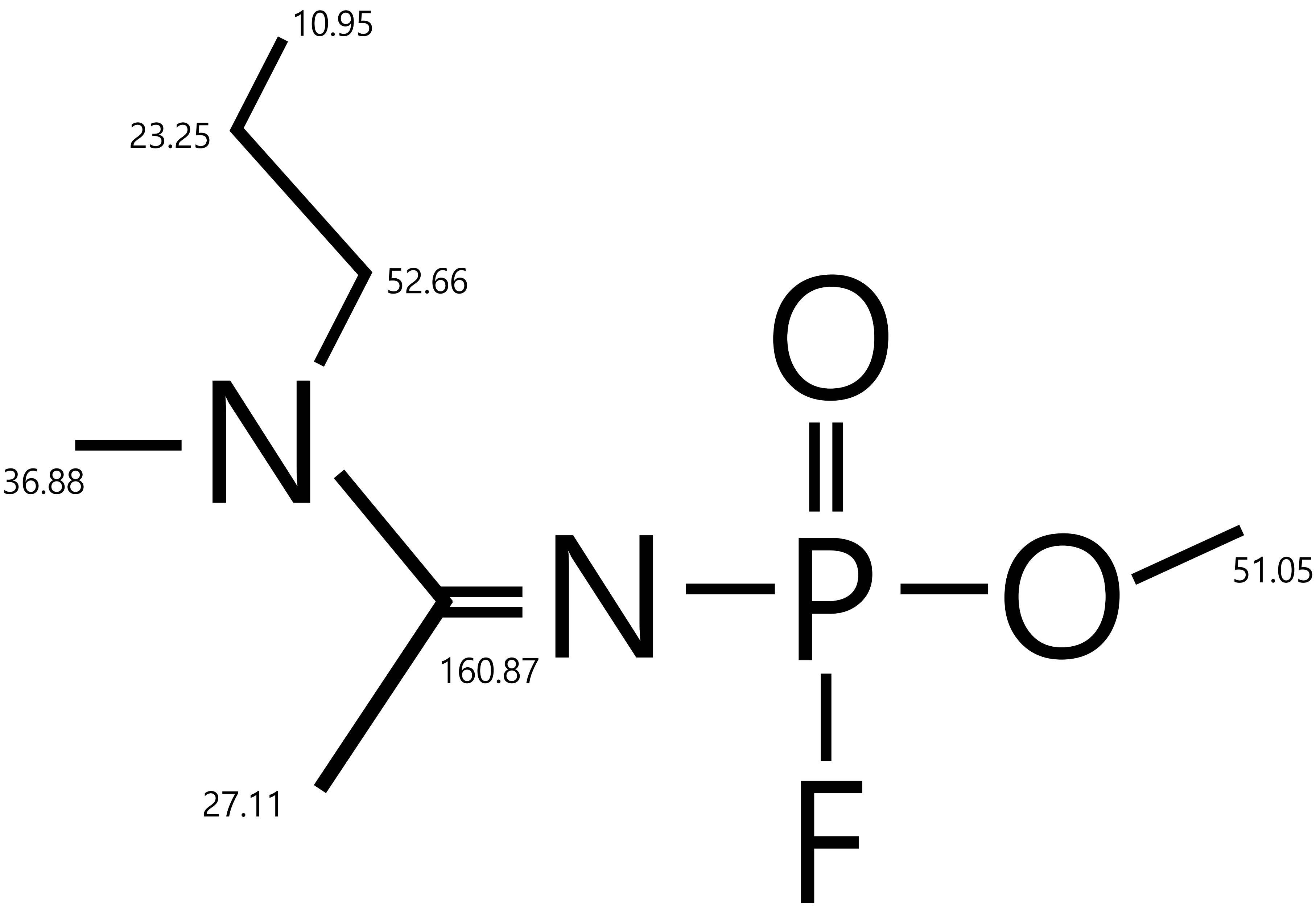

Figure S3. Structure 1113 and its ^{13}C chemical shift

1121 C

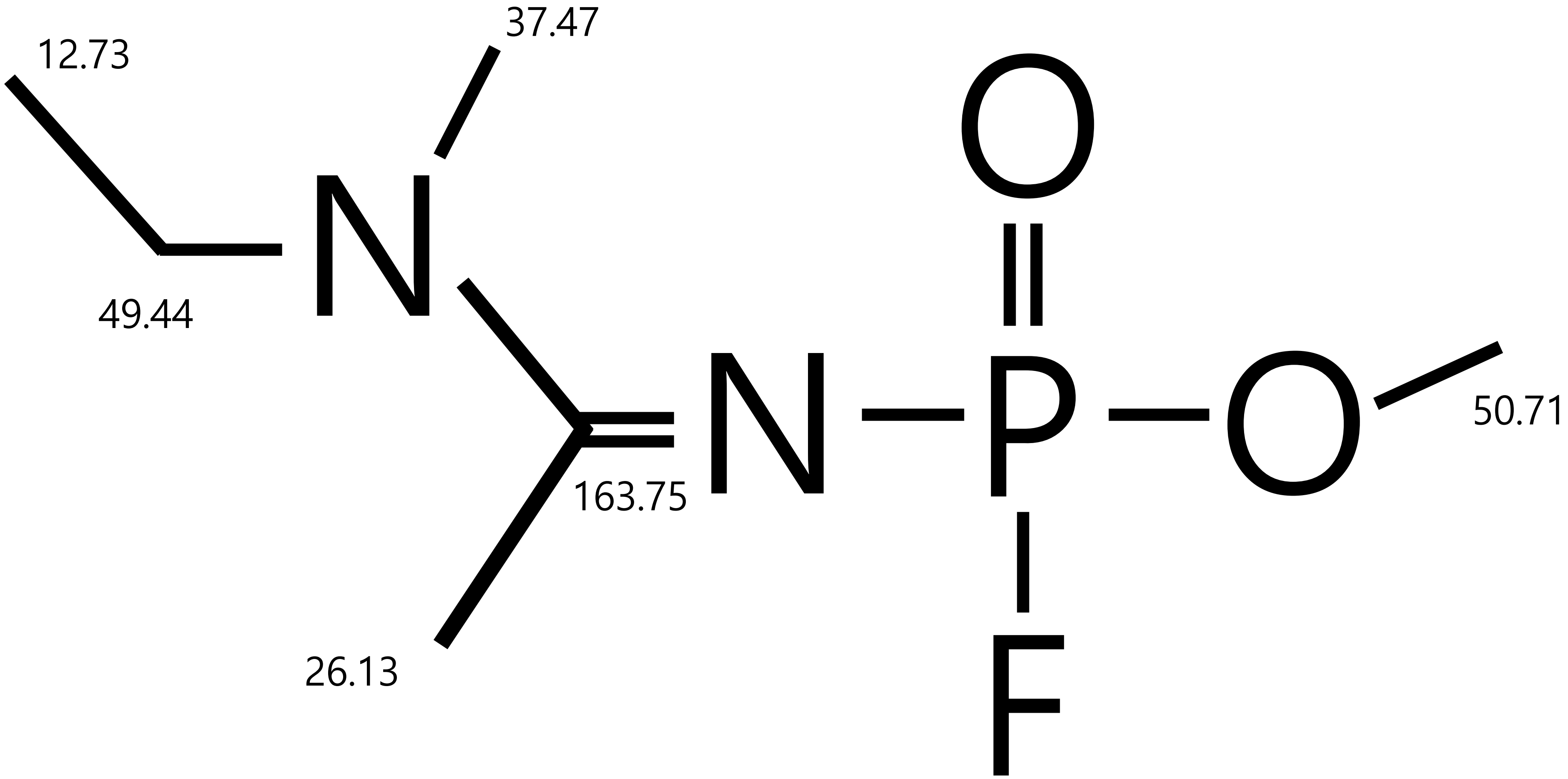

Figure S4. Structure 1121 and its ^{13}C chemical shift

1122 C

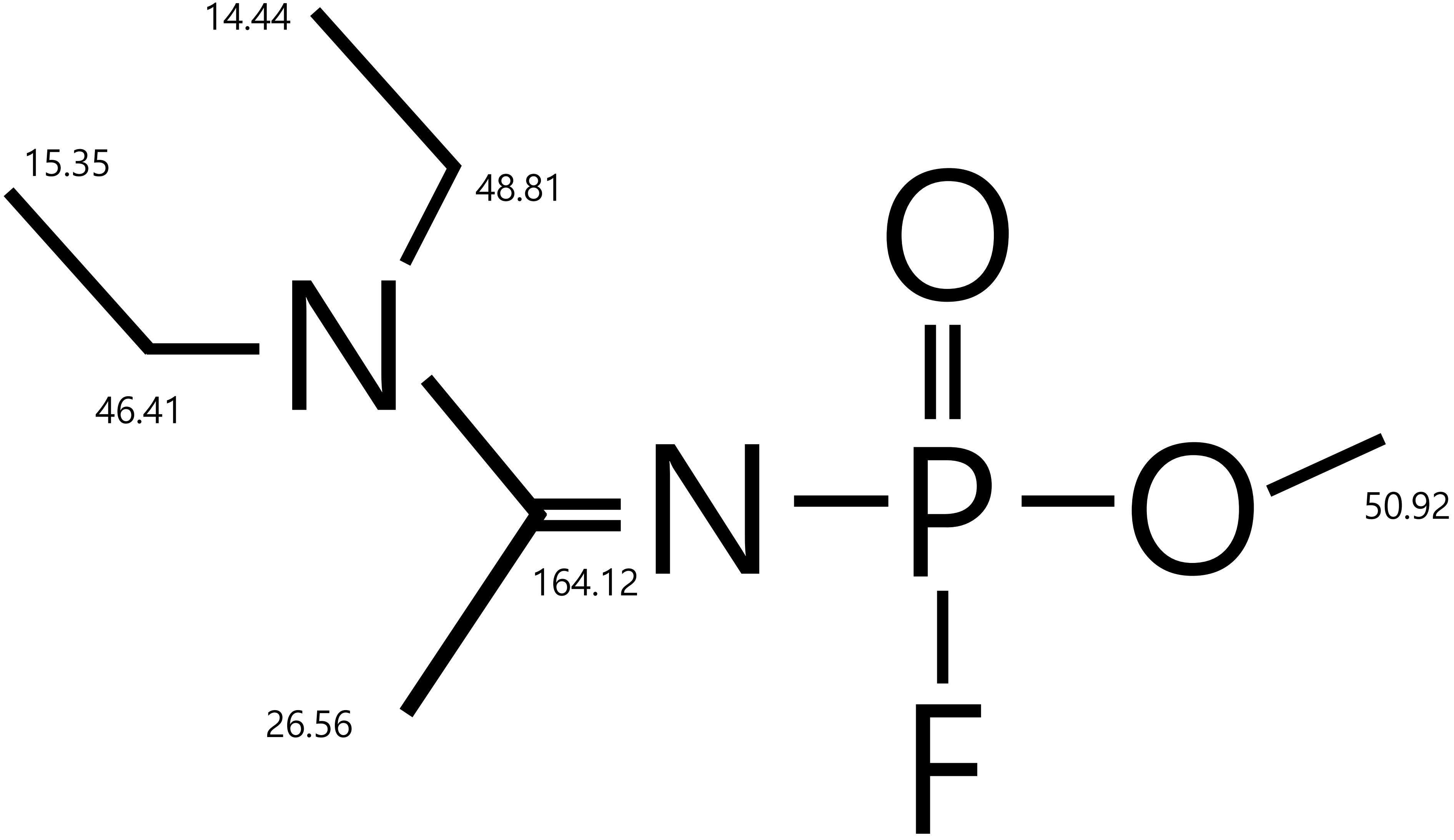

Figure S5. Structure 1122 and its ^{13}C chemical shift

1123 C

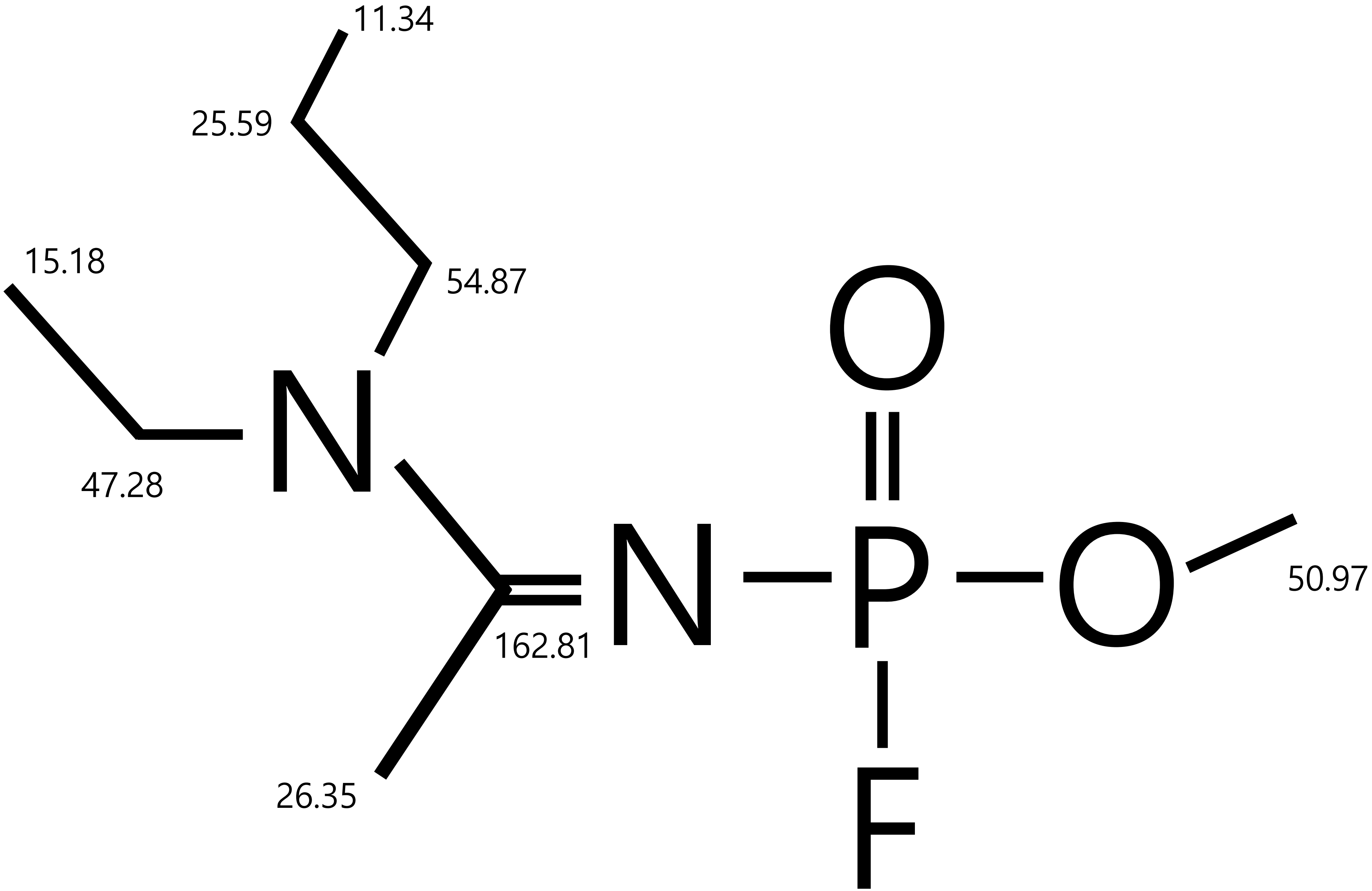

Figure S6. Structure 1123 and its ^{13}C chemical shift

1131 C

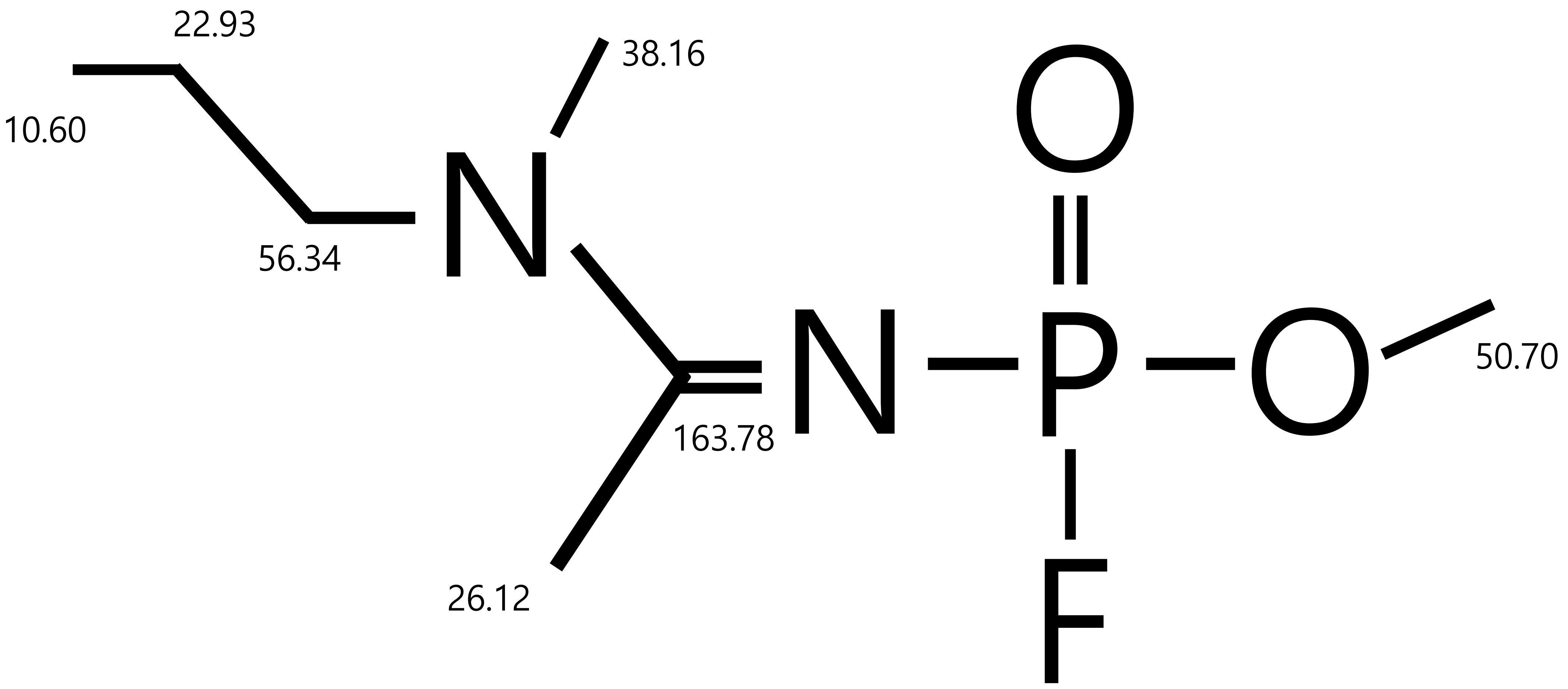

Figure S7. Structure 1131 and its ^{13}C chemical shift

1132 C

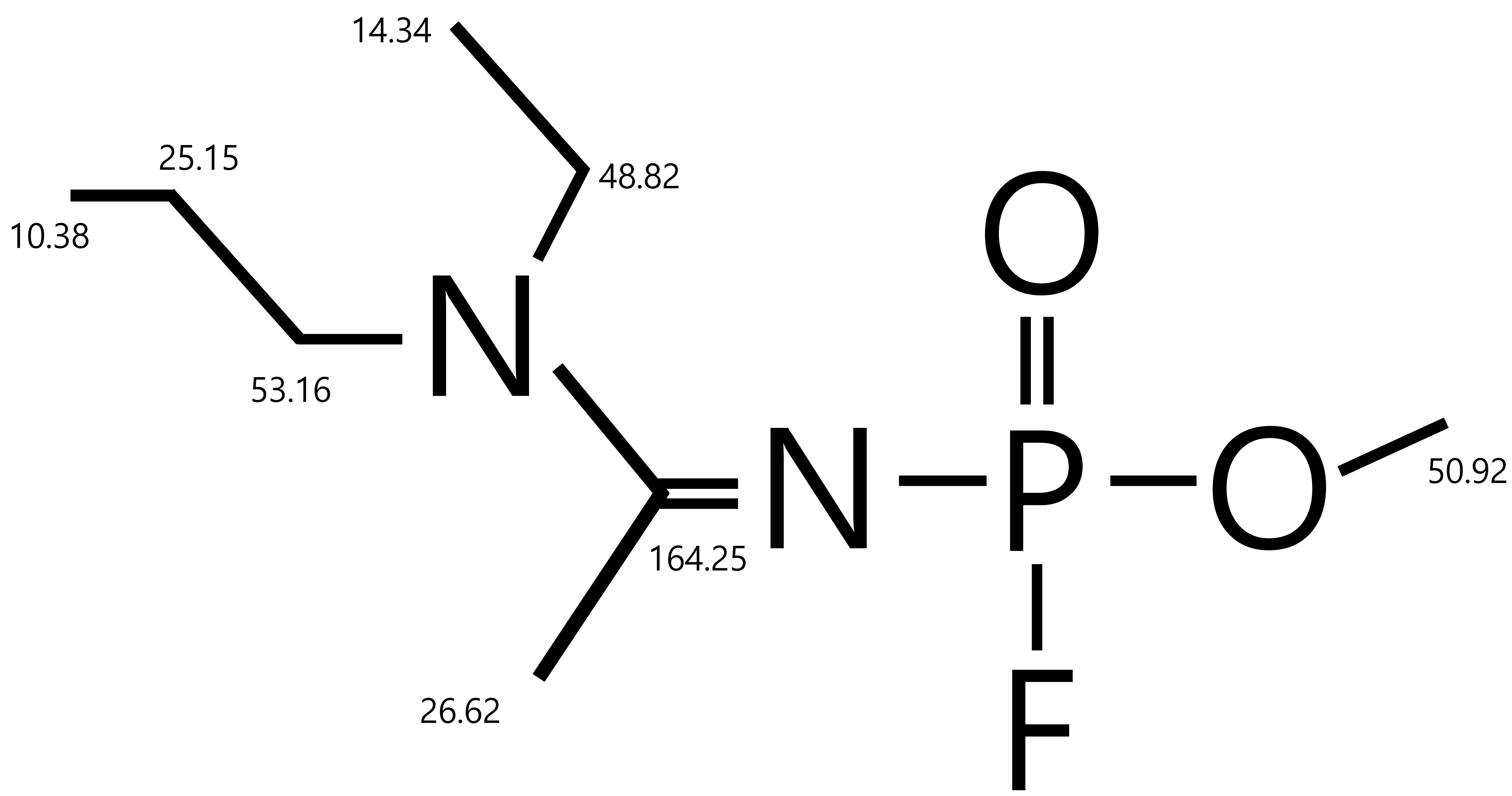

Figure 8. Structure 1132 and its ^{13}C chemical shift

1133 C

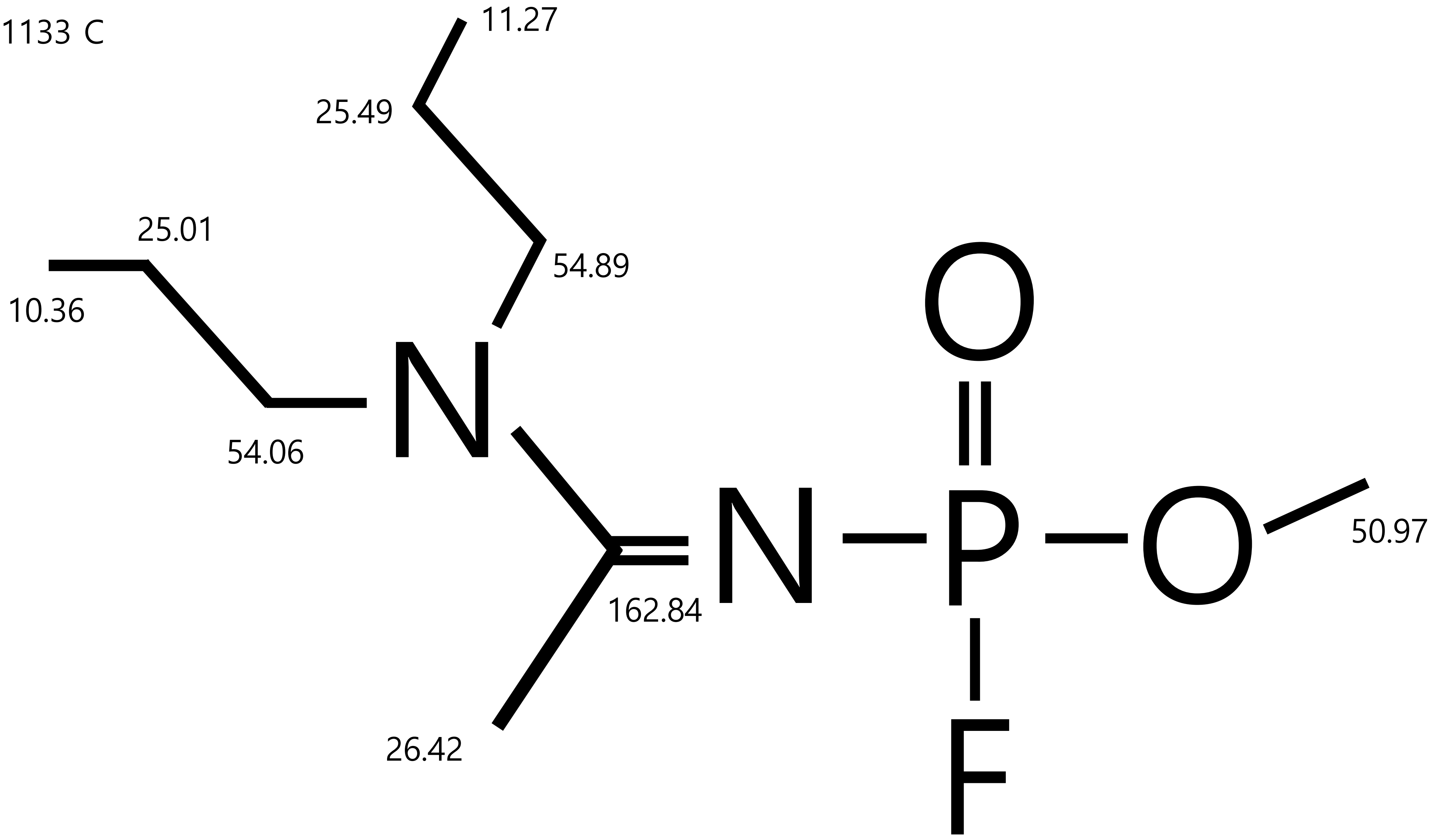

Figure S9. Structure 1133 and its ^{13}C chemical shift

1211 C

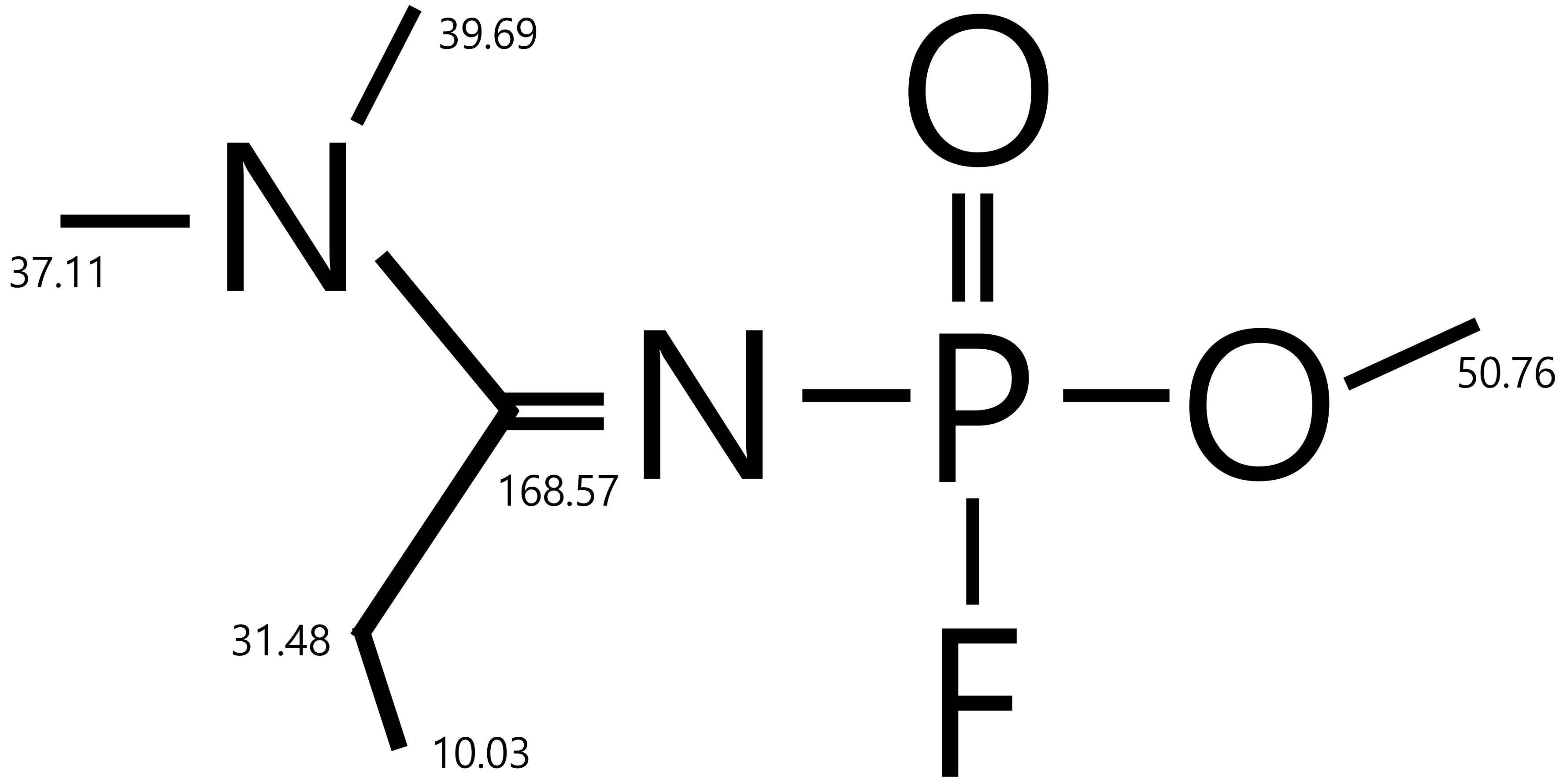

Figure S10. Structure 1211 and its ^{13}C chemical shift

1212 C

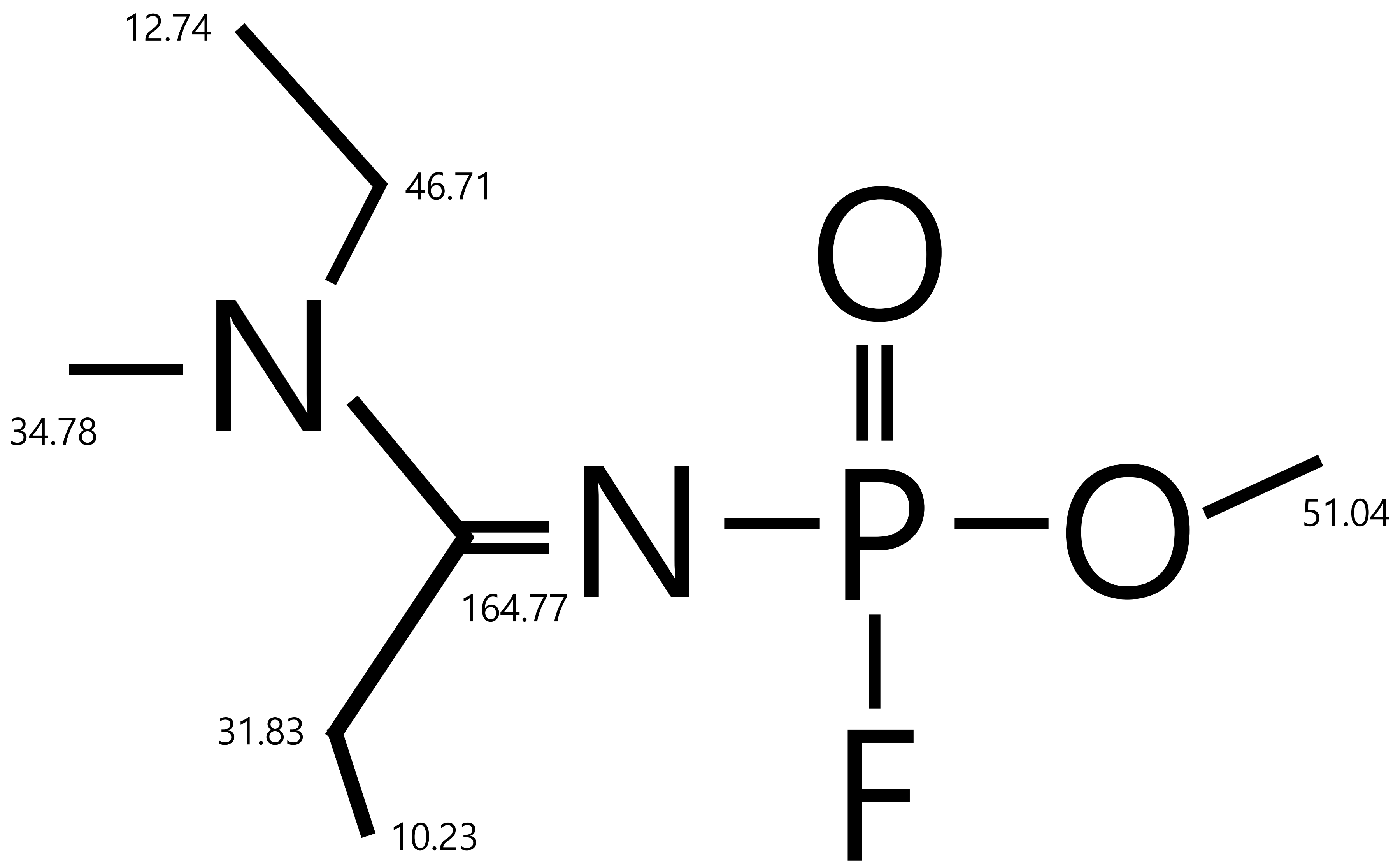

Figure S11. Structure 1212 and its ^{13}C chemical shift

1213 C

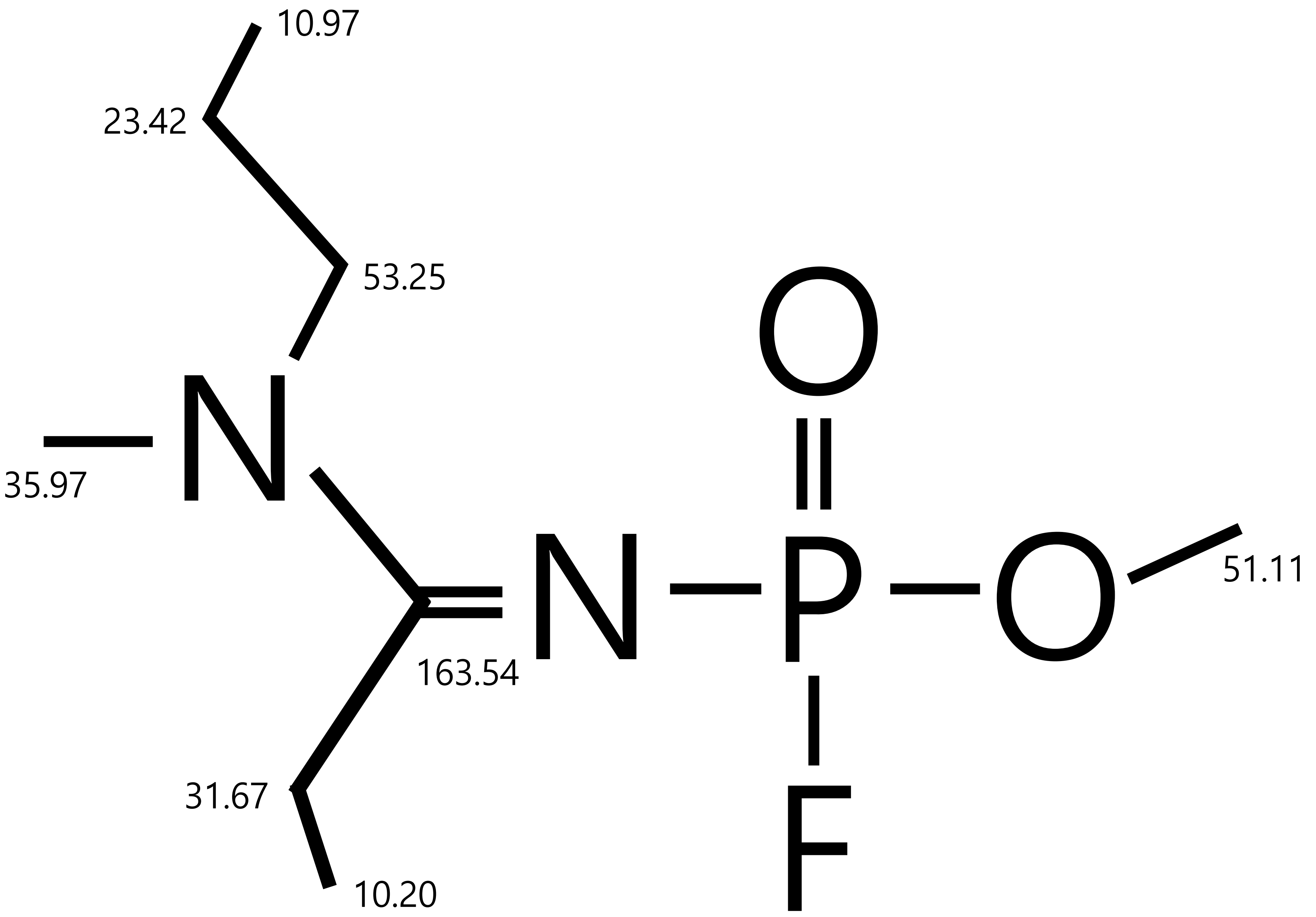

Figure S12. Structure 1213 and its ^{13}C chemical shift

1221 C

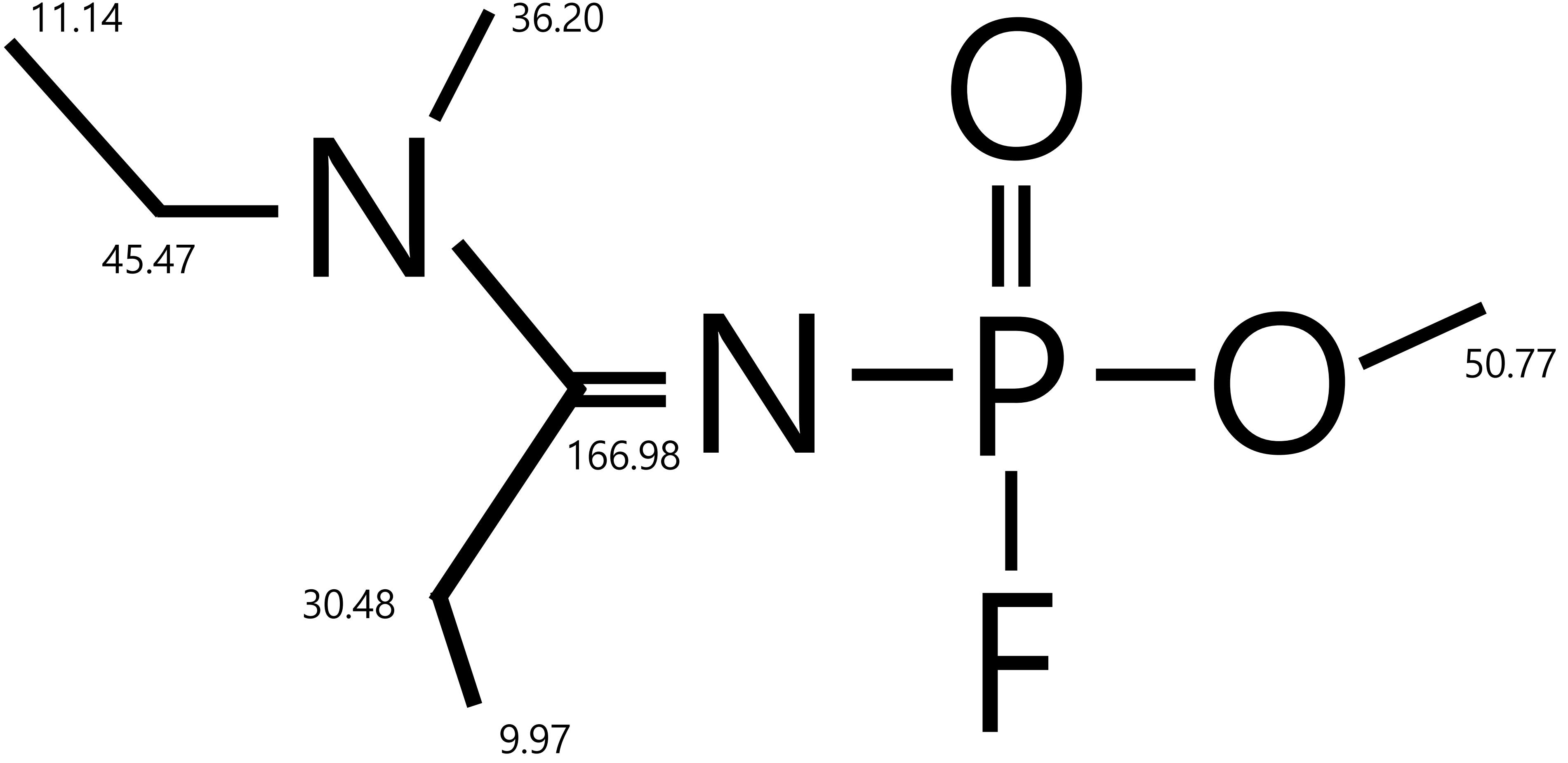

Figure S13. Structure 1221 and its ^{13}C chemical shift

1222 C

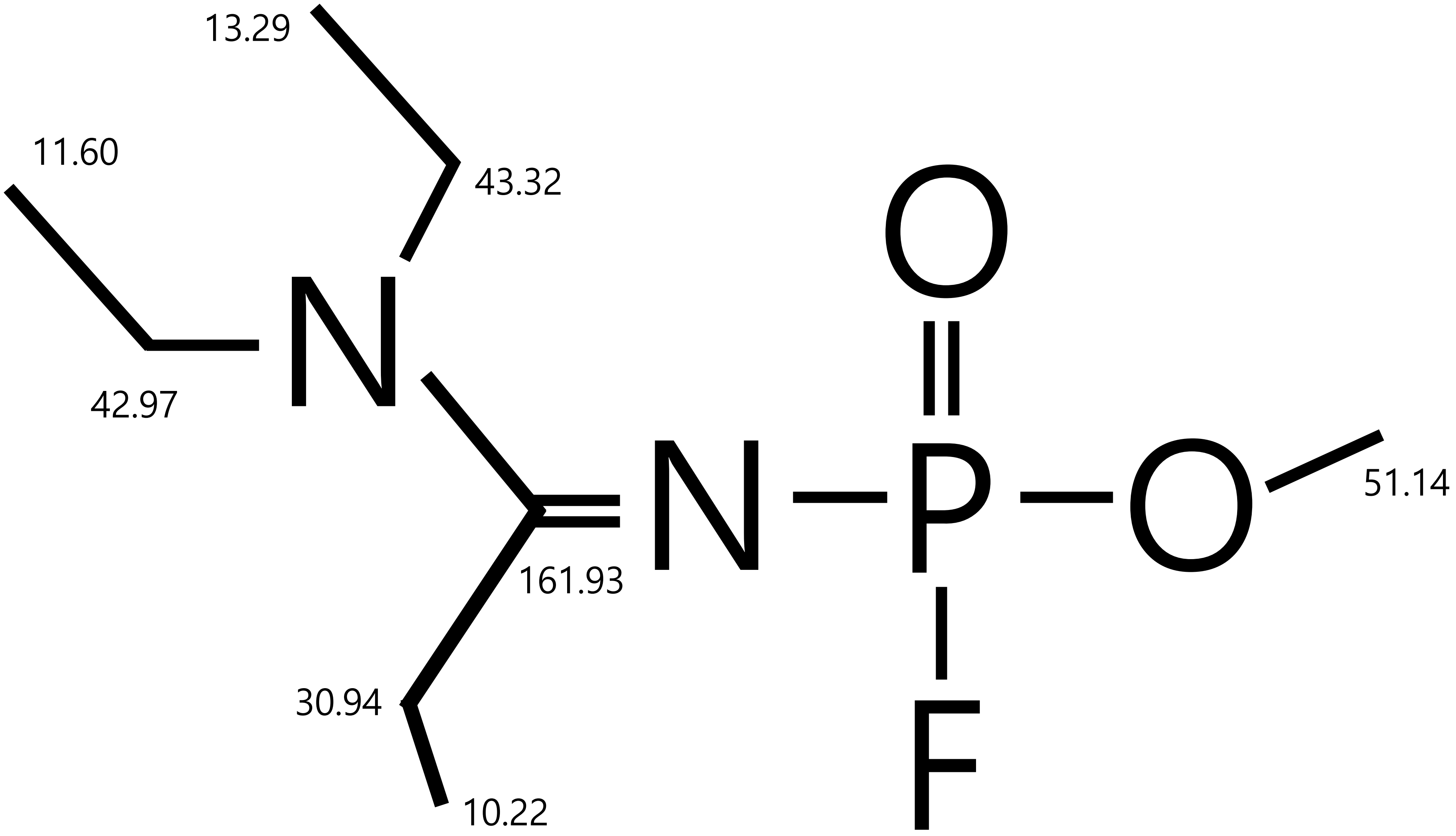

Figure S14. Structure 1222 and its ^{13}C chemical shift

1223 C

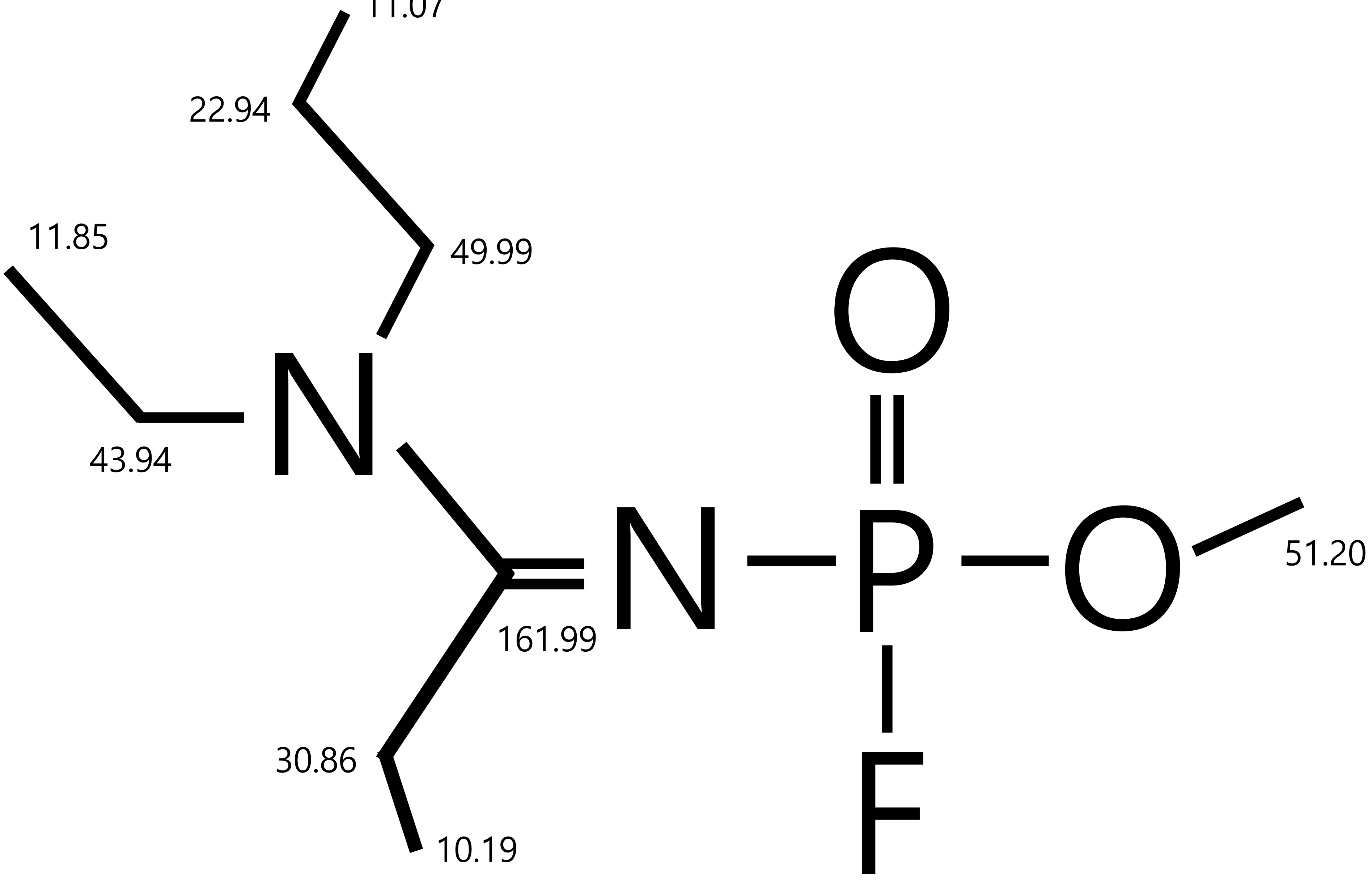

Figure S15. Structure 1223 and its ^{13}C chemical shift

1231 C

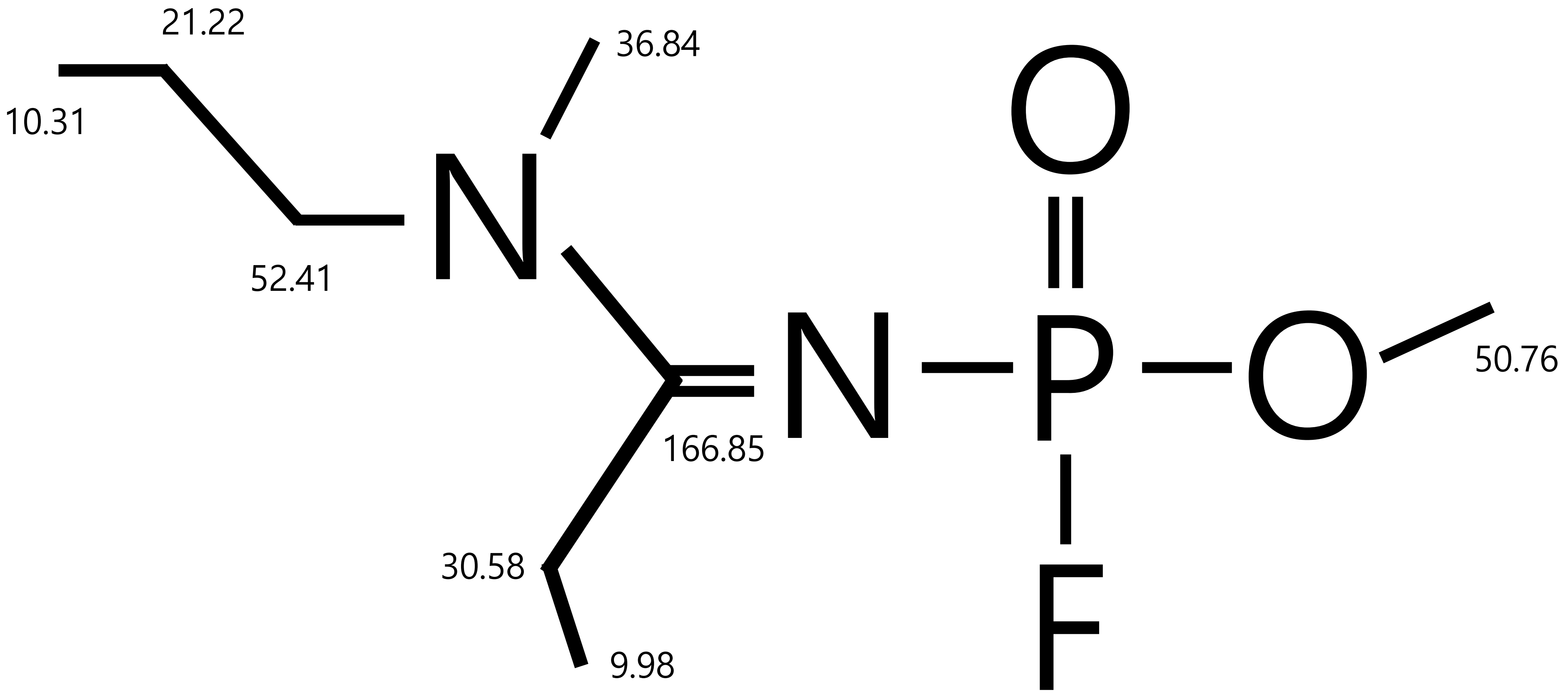

Figure S16. Structure 1231 and its ^{13}C chemical shift

1232 C

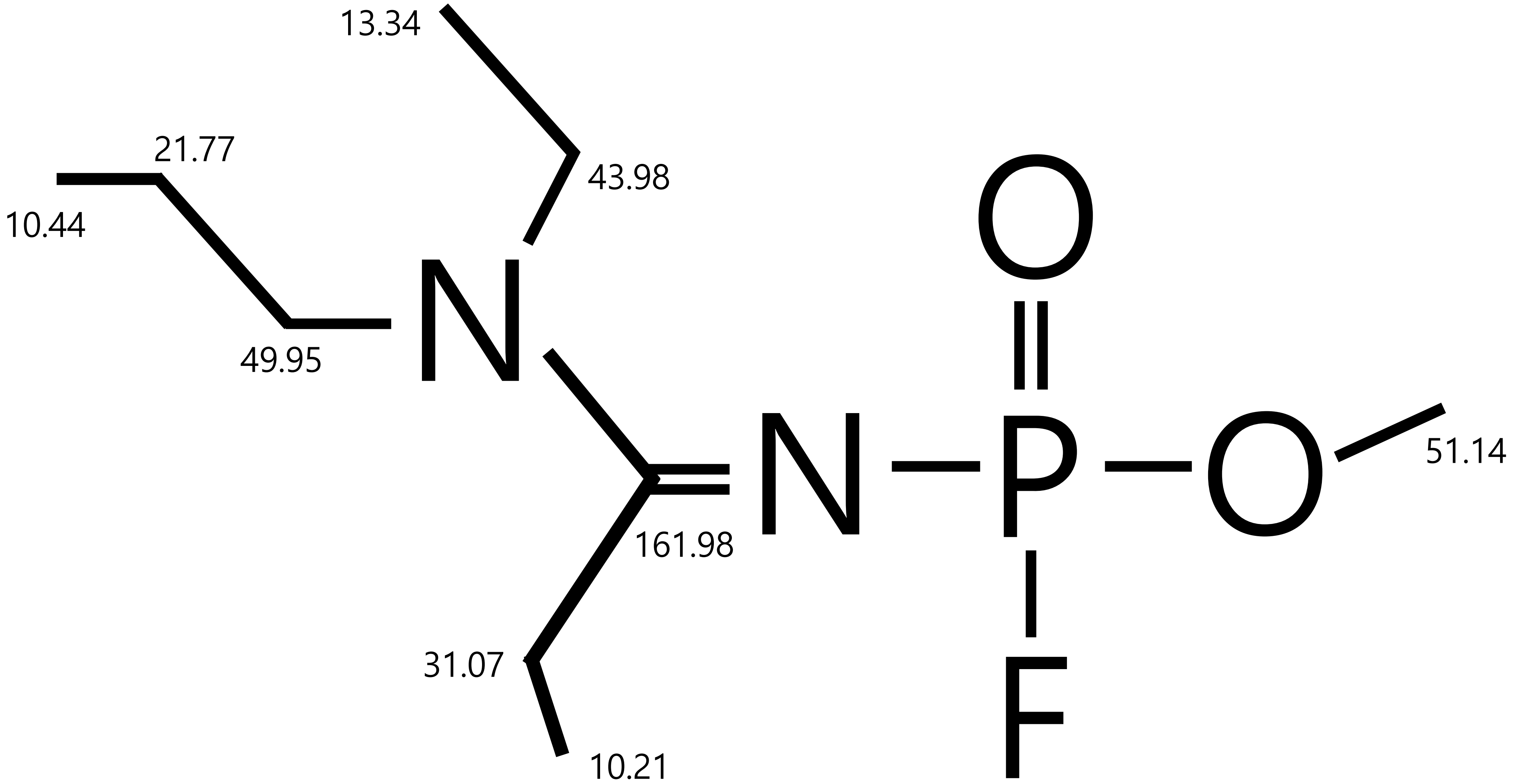

Figure S17. Structure 1232 and its ^{13}C chemical shift

1233 C

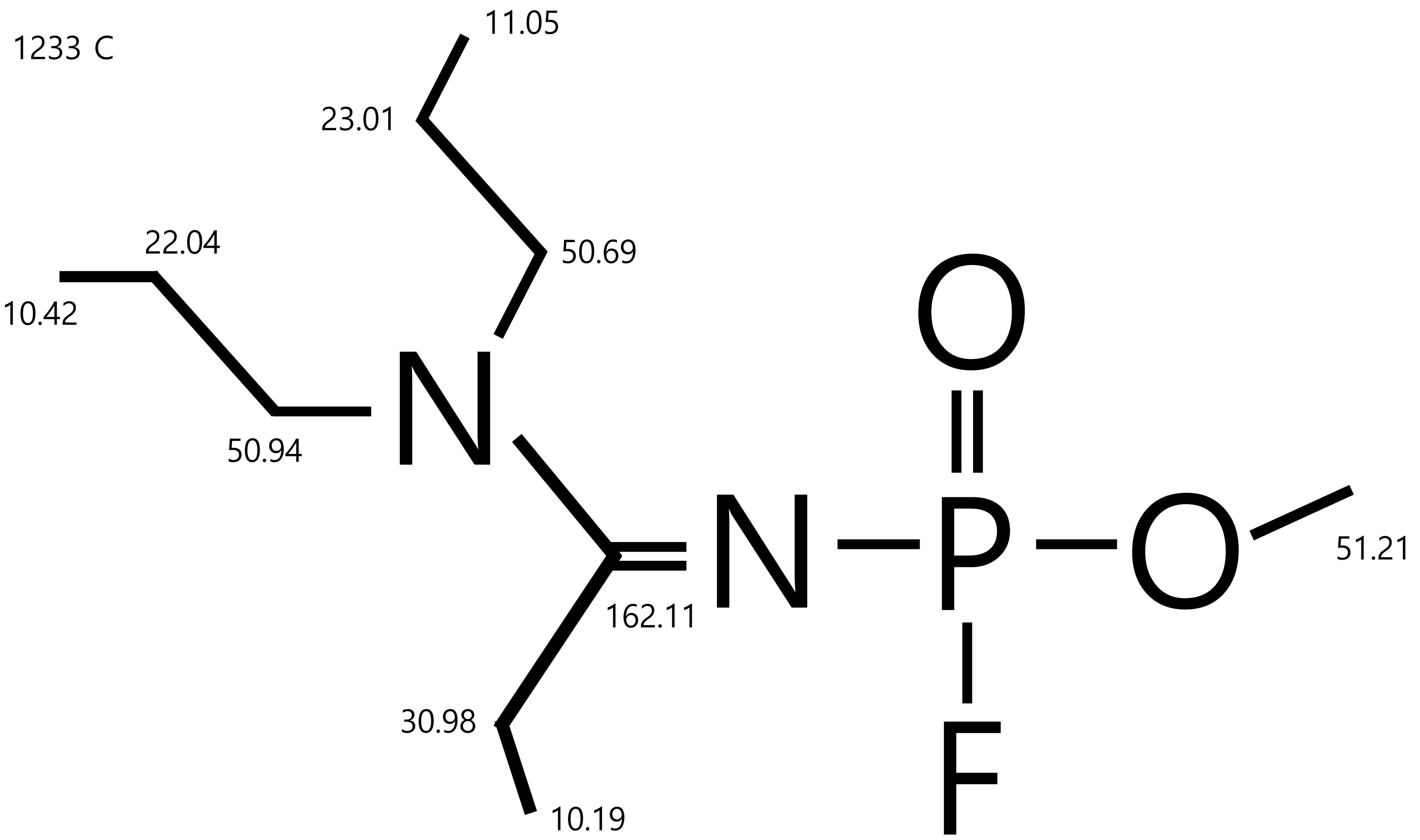

Figure S18. Structure 1233 and its ^{13}C chemical shift

1311 C

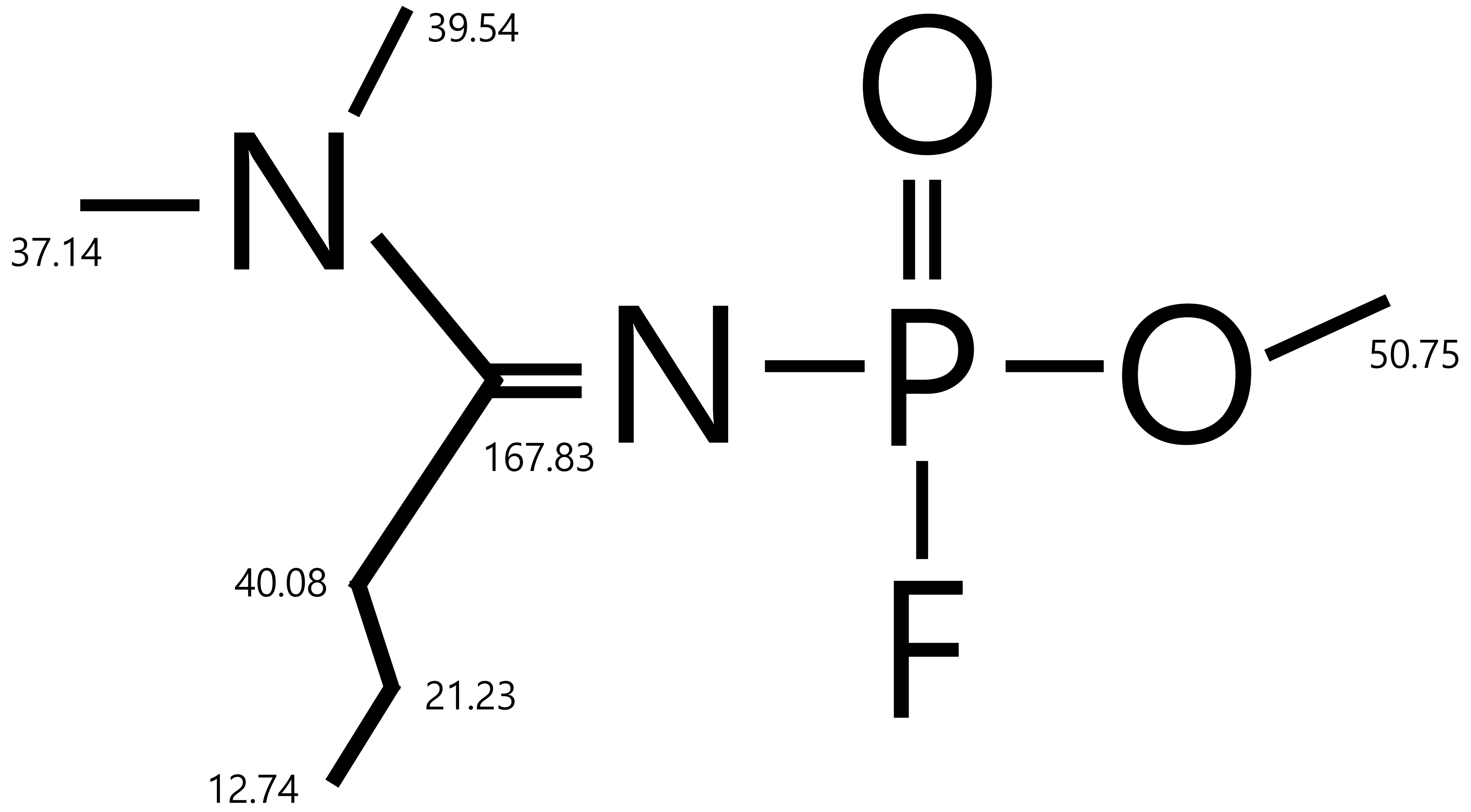

Figure S19. Structure 1311 and its ^{13}C chemical shift

1312 C

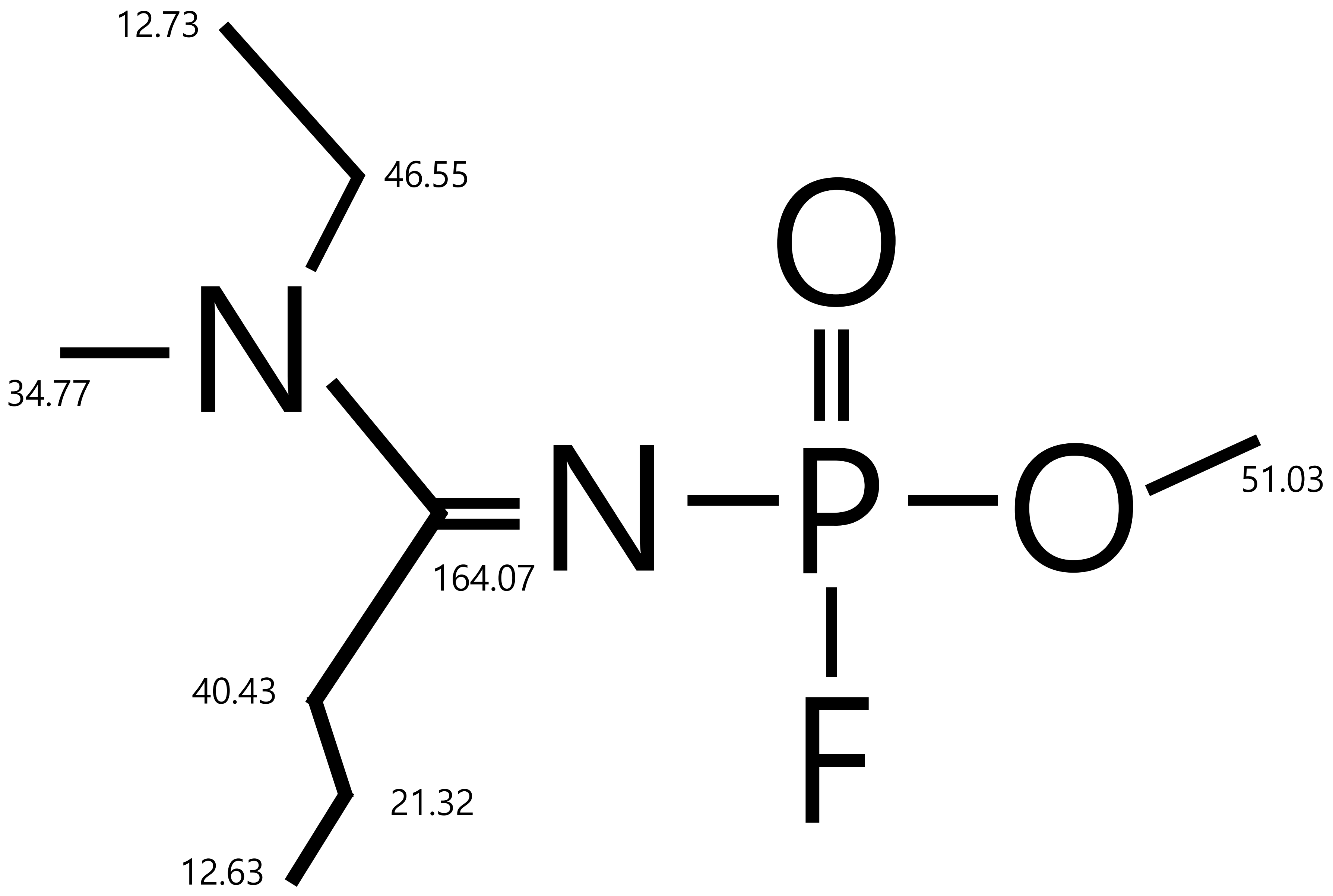

Figure S20. Structure 1312 and its ^{13}C chemical shift

1313 C

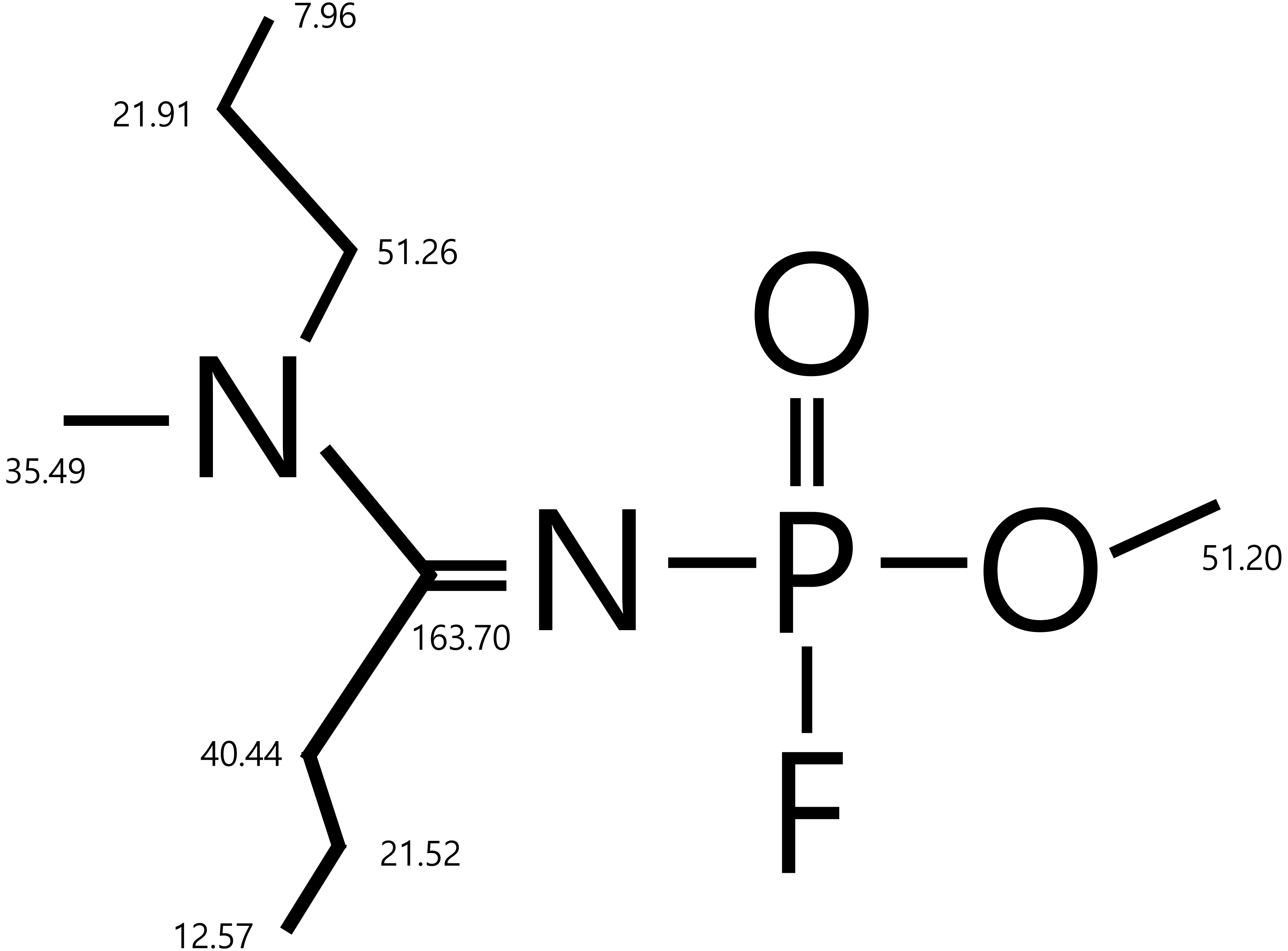

Figure S21. Structure 1313 and its ^{13}C chemical shift

1321 C

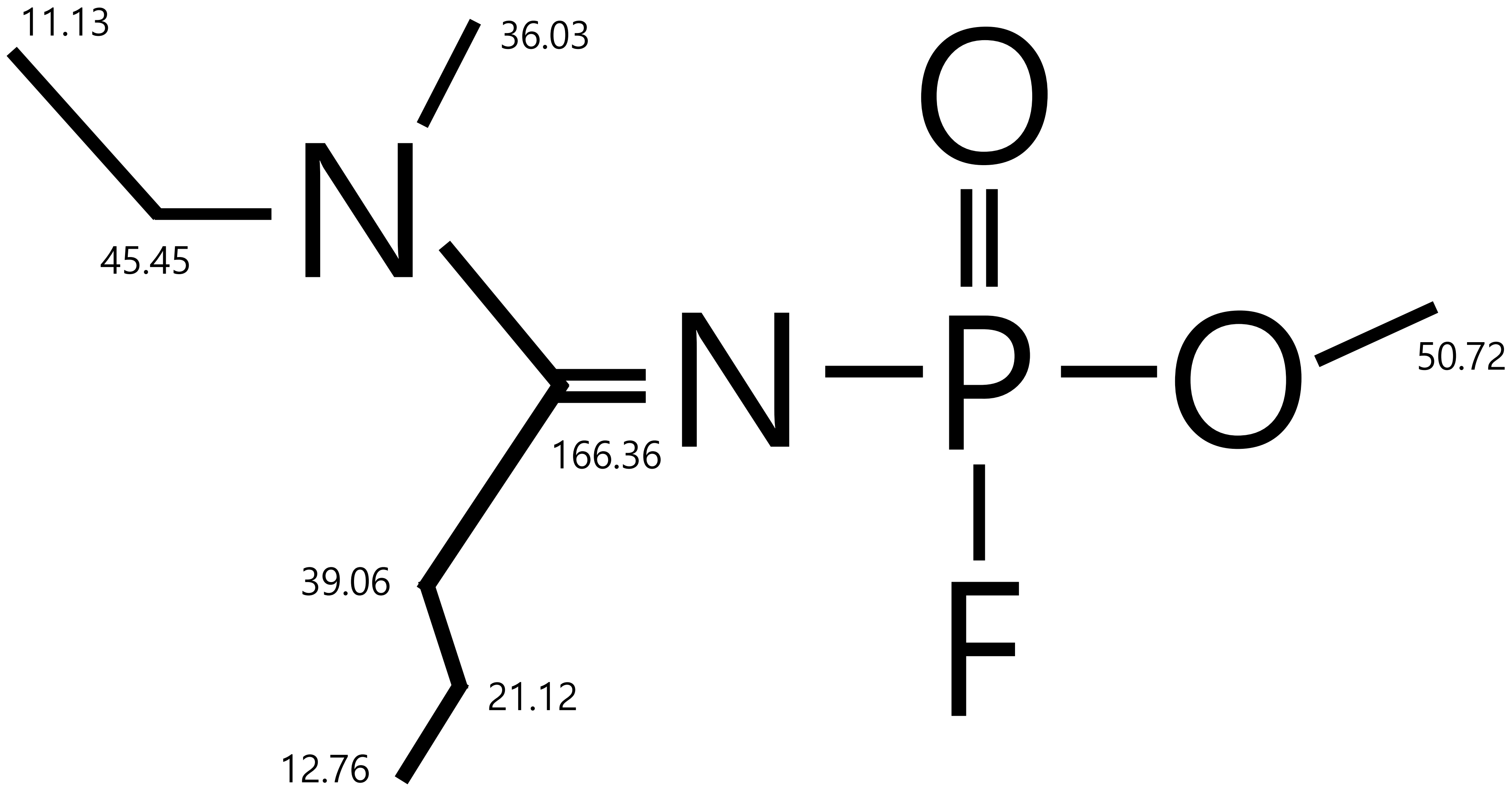

Figure S22. Structure 1321 and its ^{13}C chemical shift

1322 C

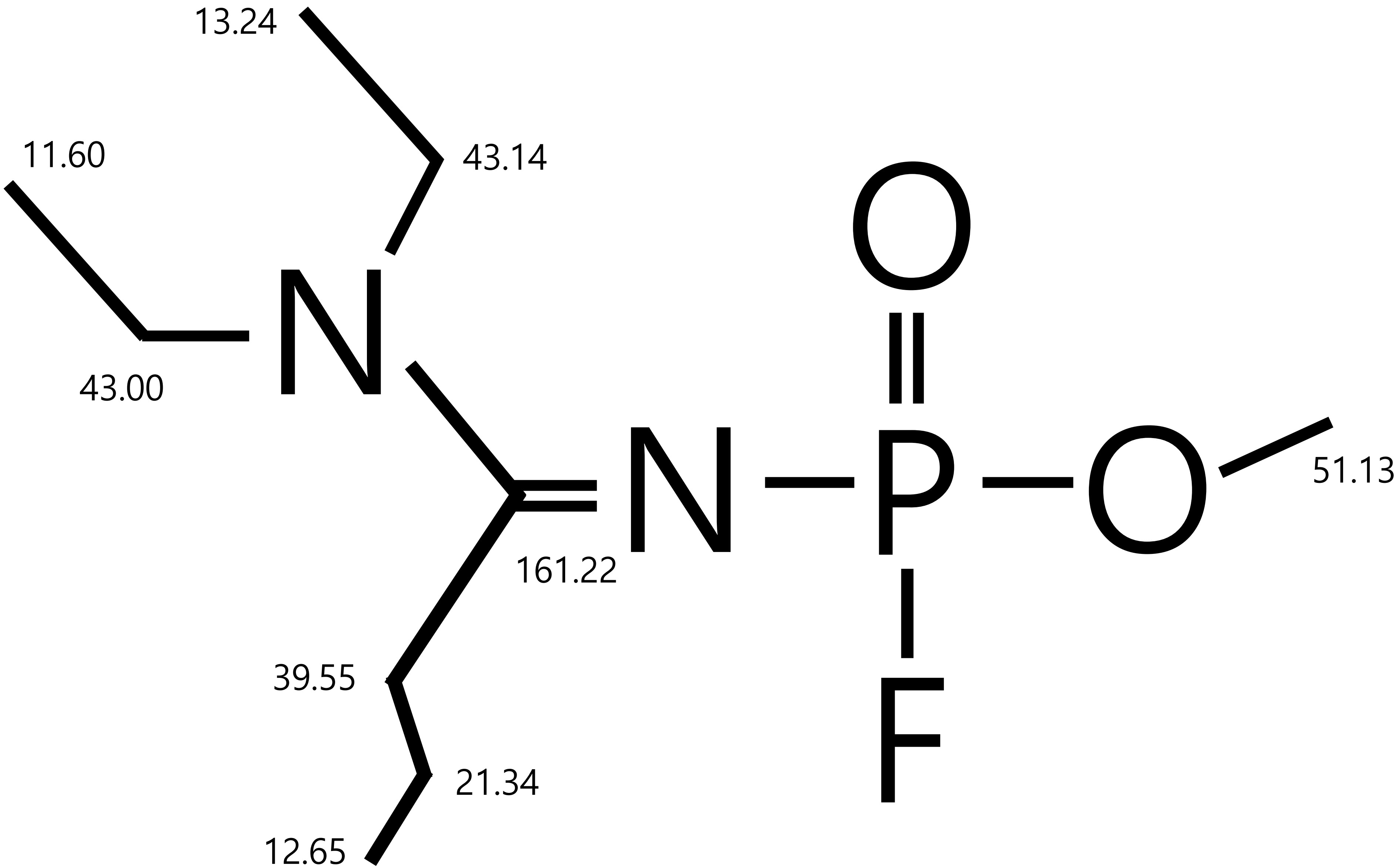

Figure S23. Structure 1322 and its ^{13}C chemical shift

1323 C

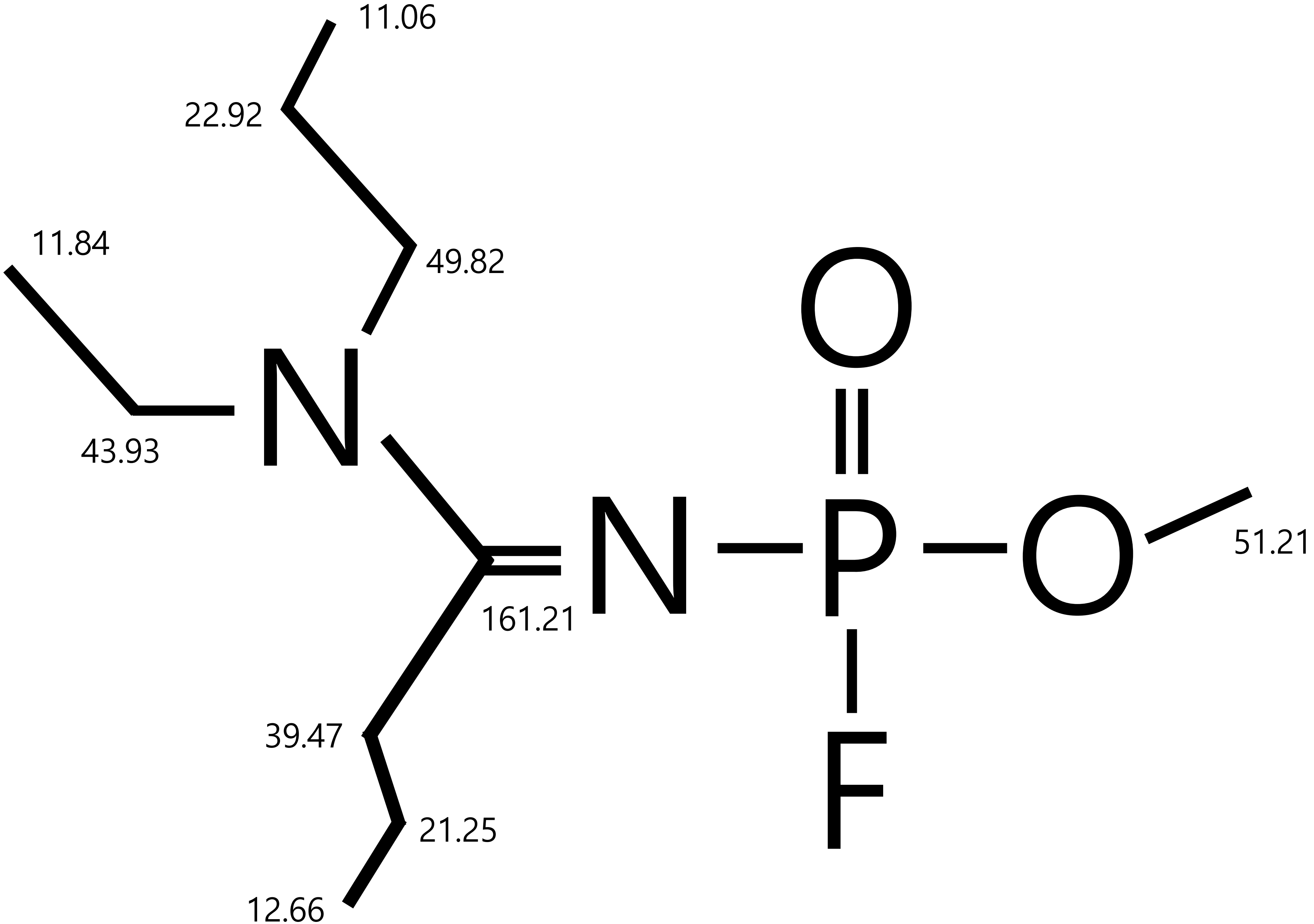

Figure S24. Structure 1323 and its ^{13}C chemical shift

1331 C

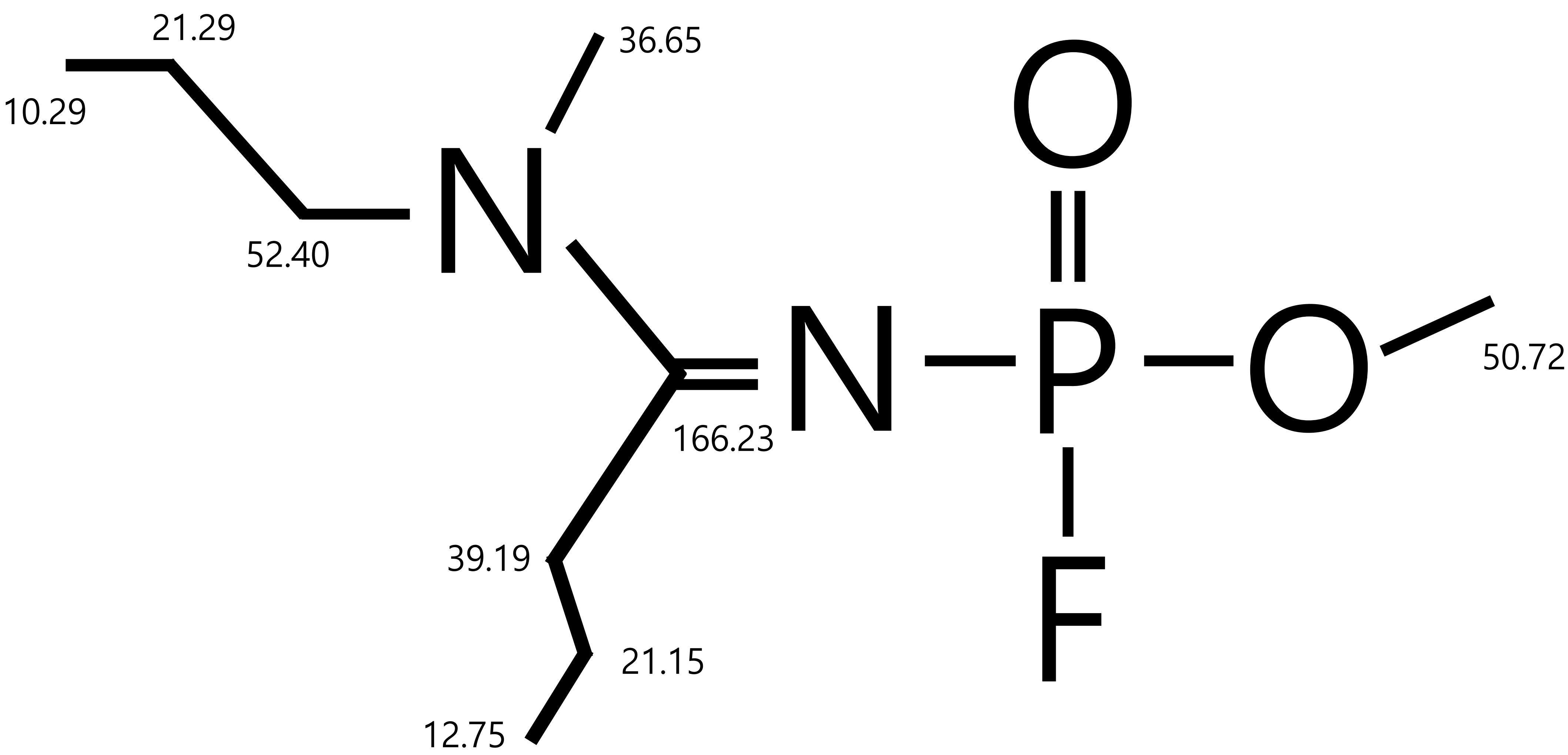

Figure S25. Structure 1331 and its 13C chemical shift

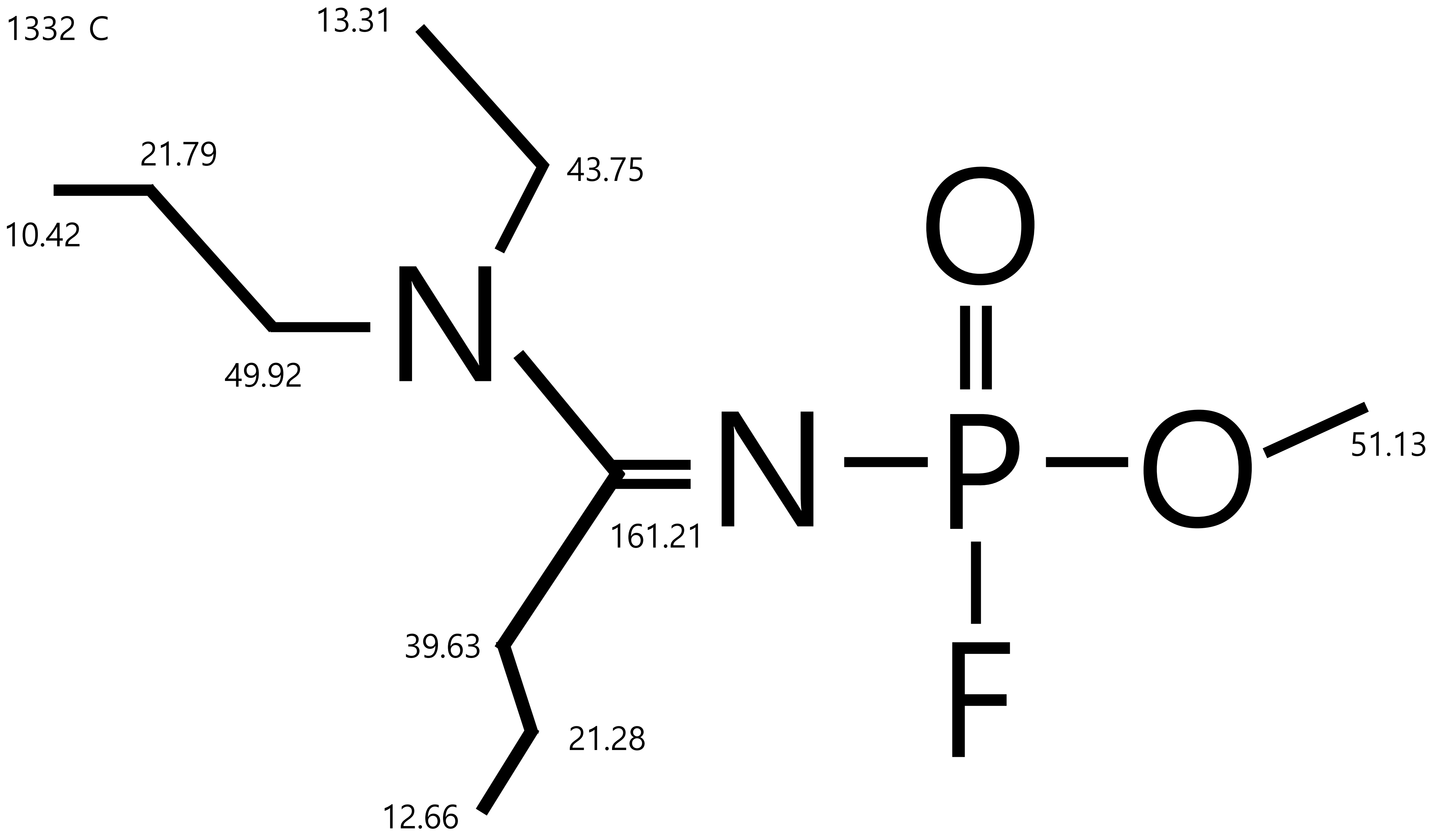

Figure S26. Structure 1332 and its ^{13}C chemical shift

1333 C

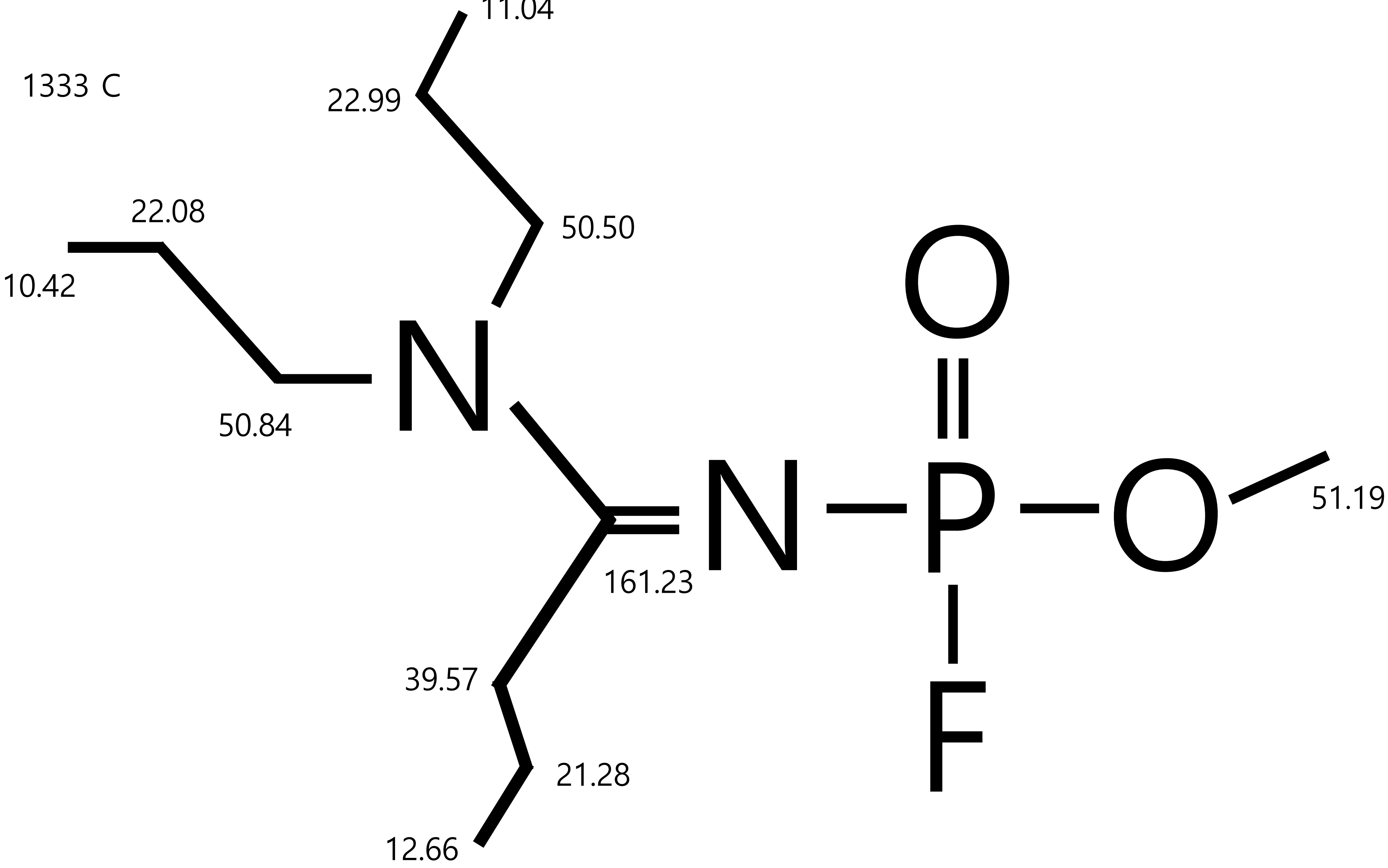

Figure S27. Structure 1333 and its ^{13}C chemical shift

2111 C

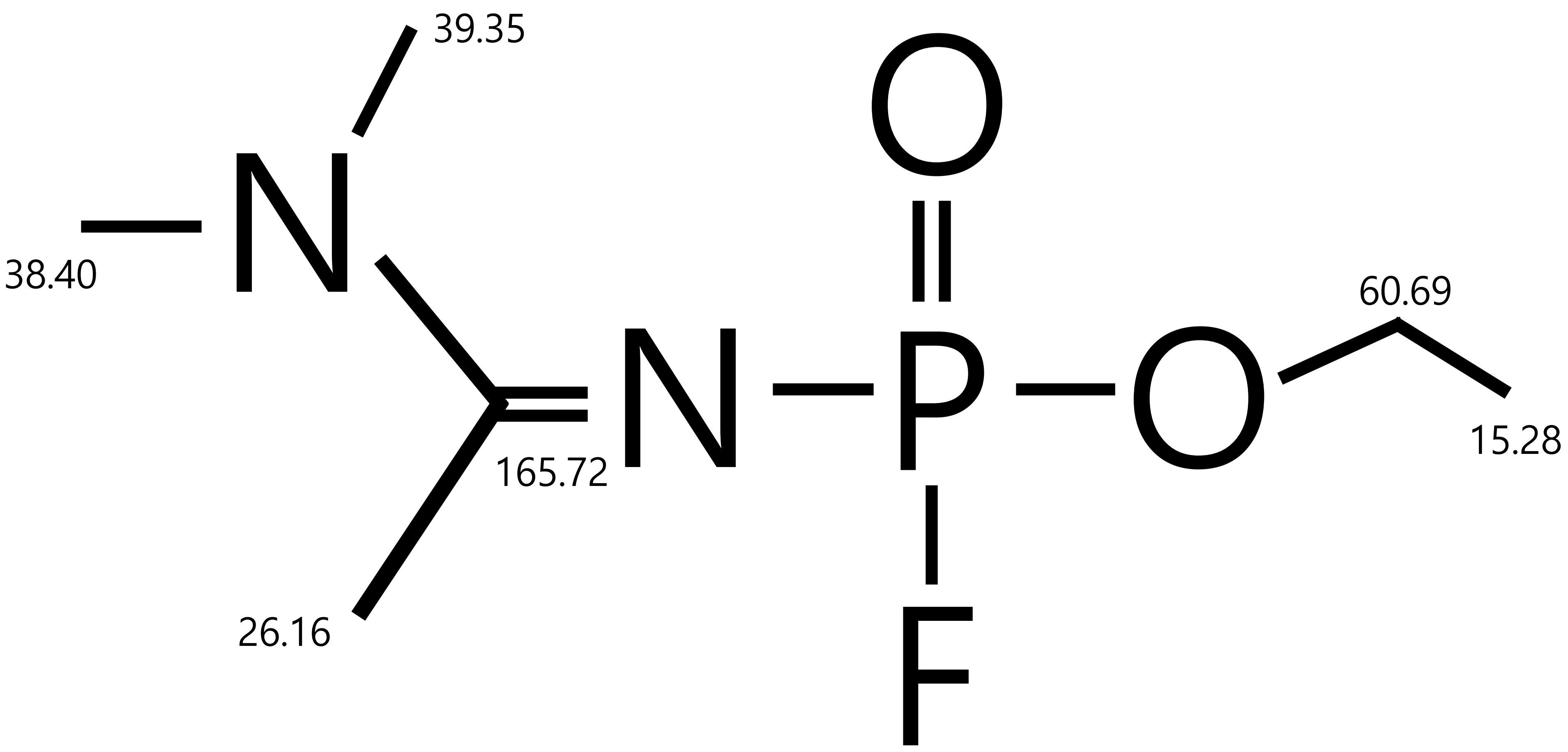

Figure S28. Structure 2111 and its ^{13}C chemical shift

2112 C

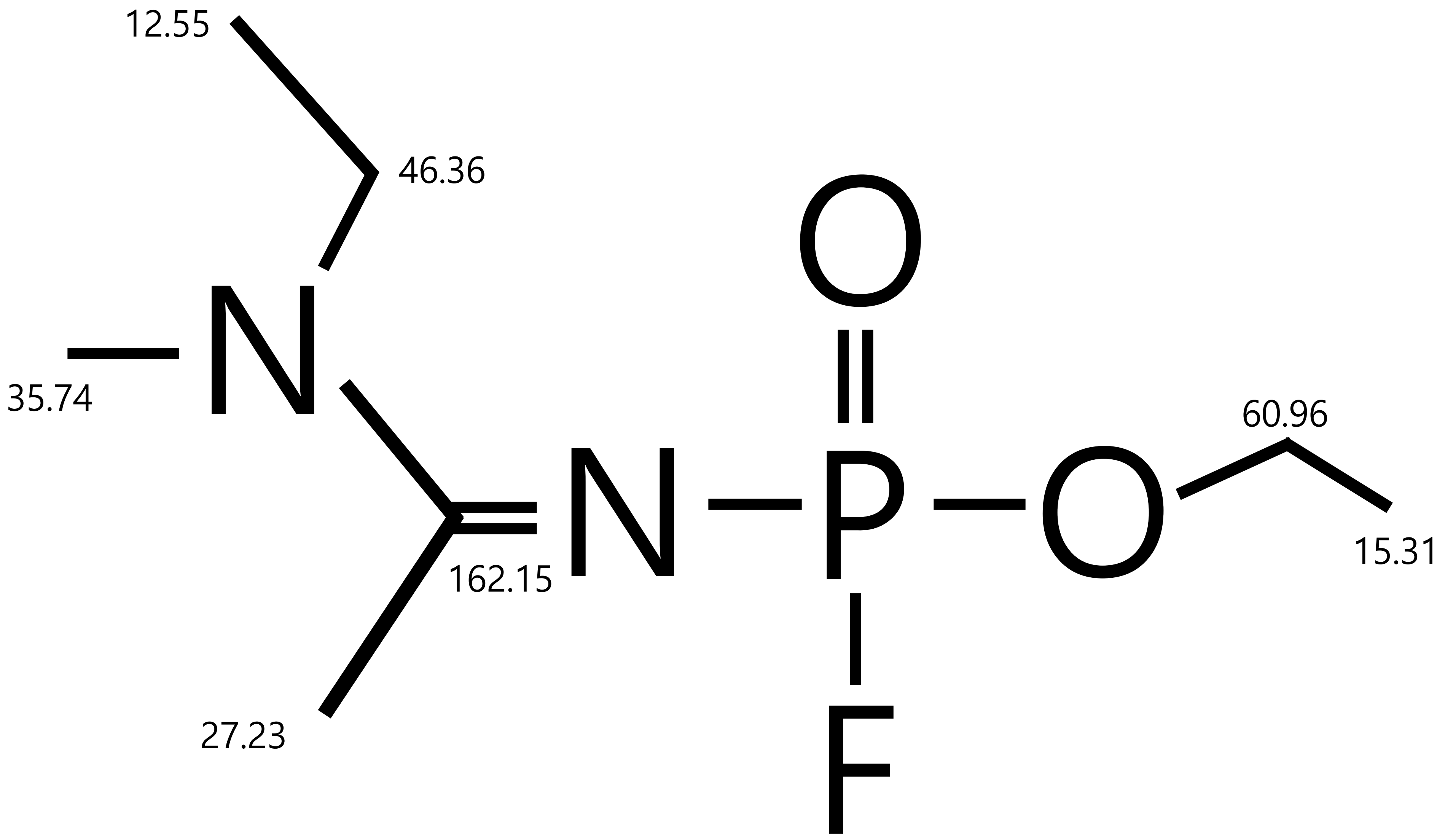

Figure S29. Structure 2112 and its ^{13}C chemical shift

2113 C

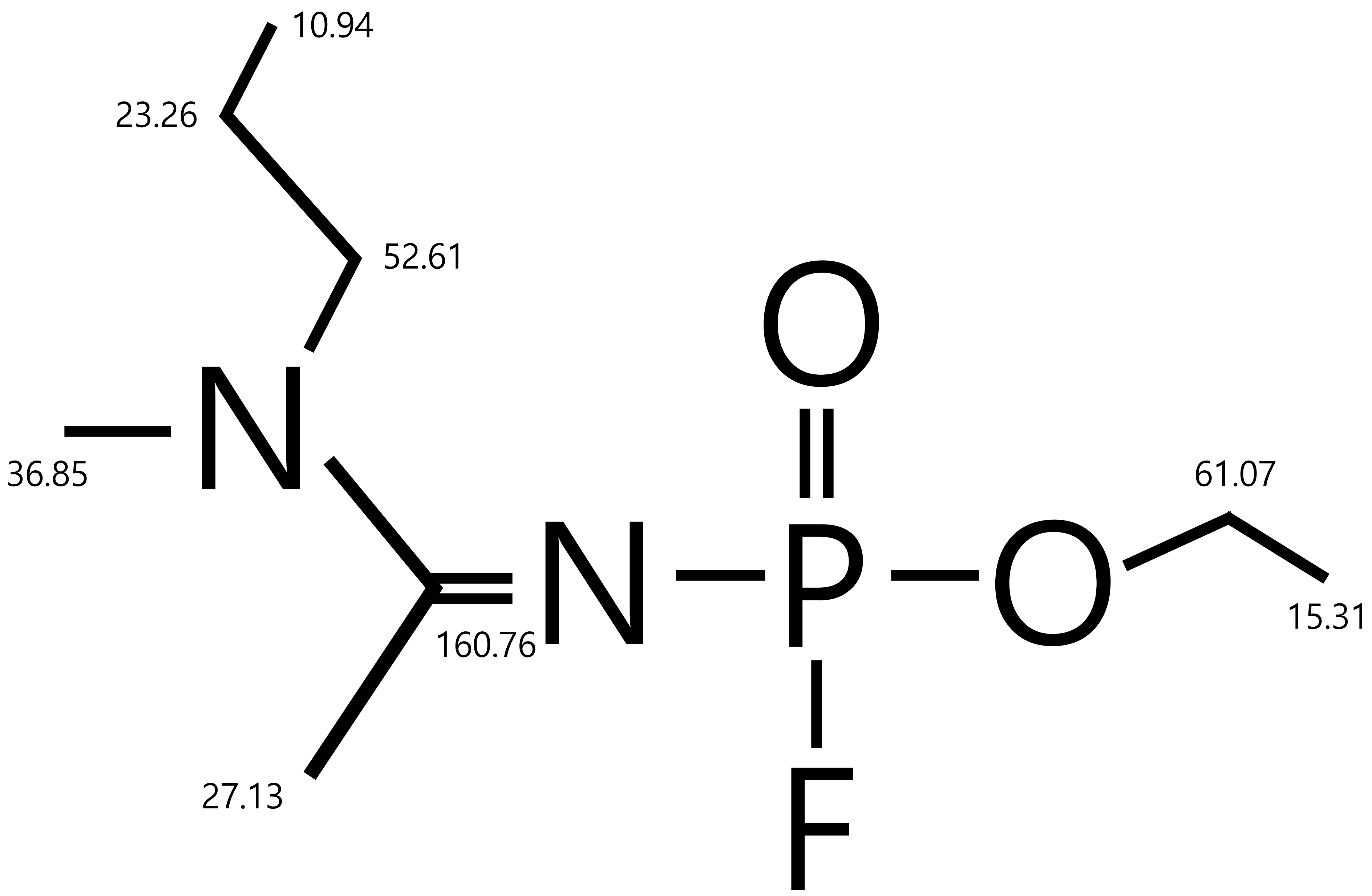

Figure S30. Structure 2113 and its 13C chemical shift

2121 C

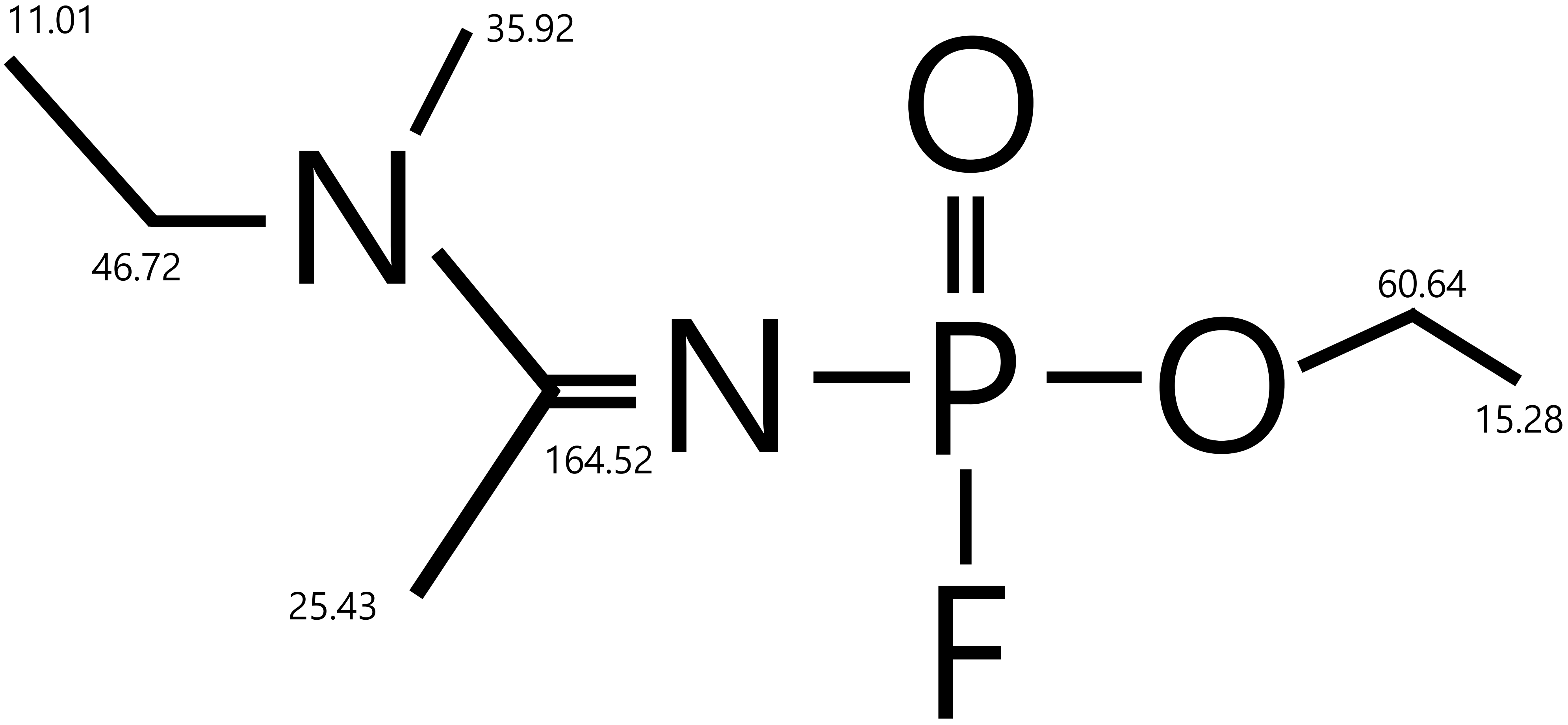

Figure S31. Structure 2121 and its ^{13}C chemical shift

2122 C

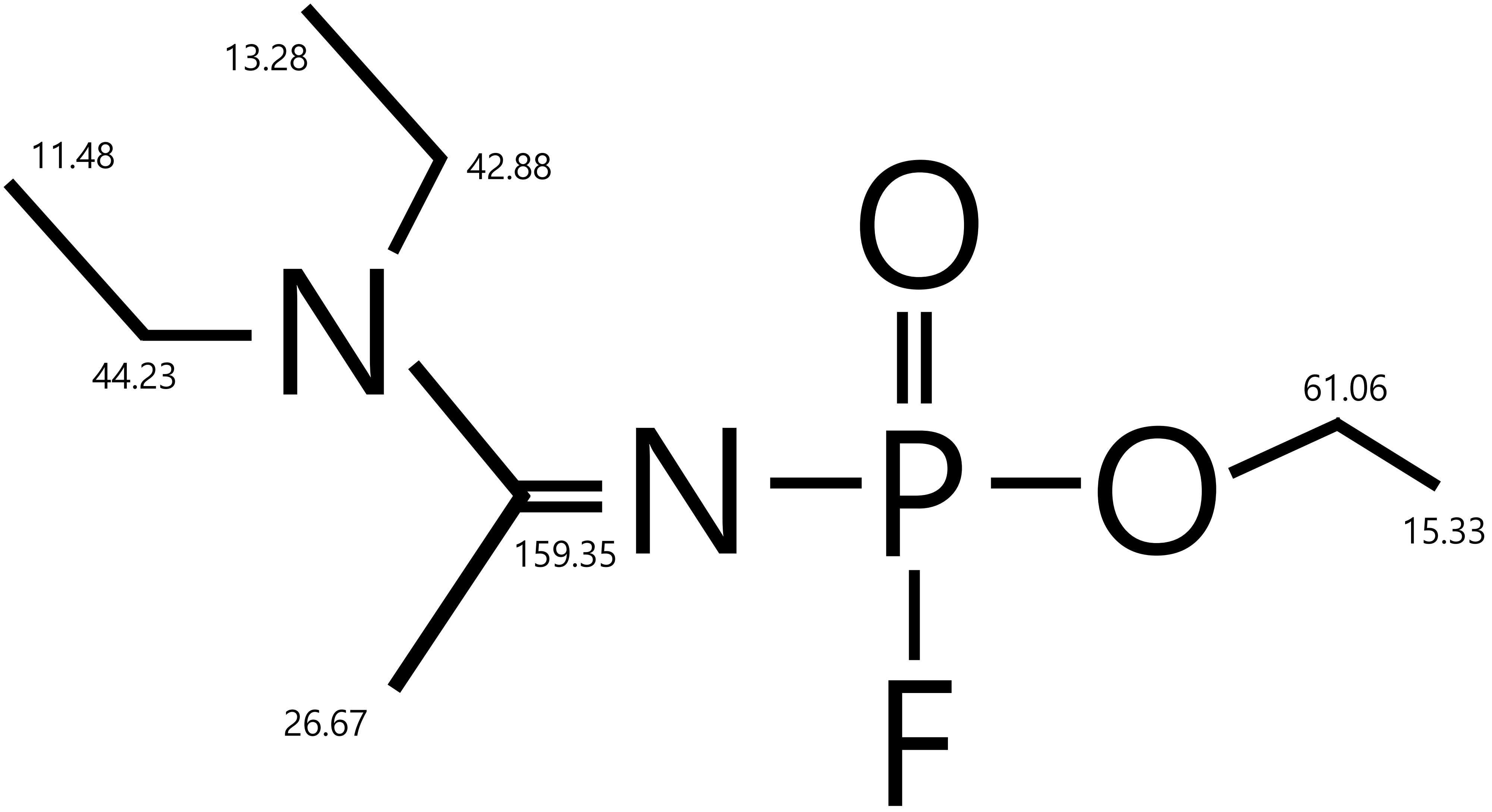

Figure S32. Structure 2122 and its ^{13}C chemical shift

2123 C

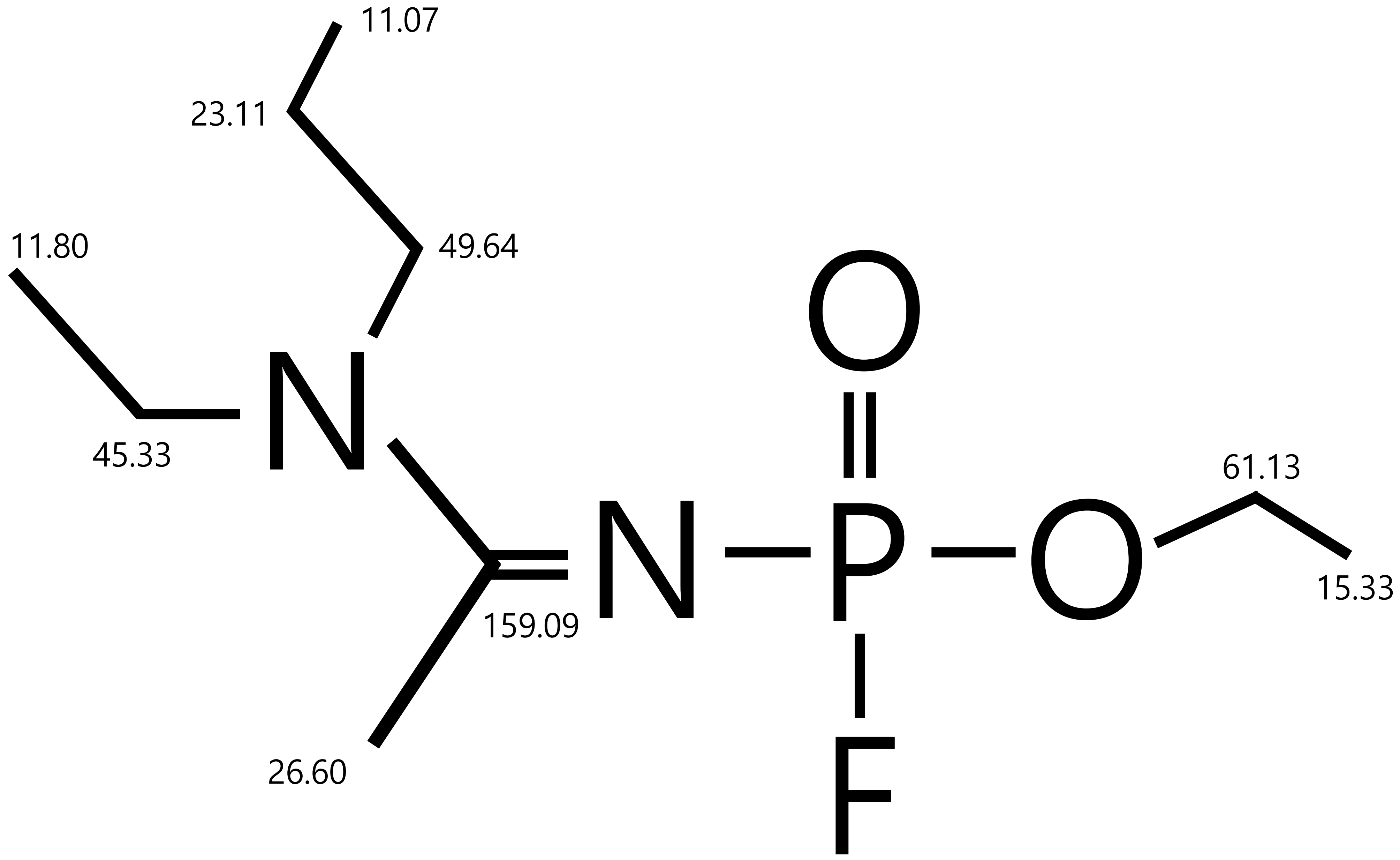

Figure S33. Structure 2123 and its ^{13}C chemical shift

2131 C

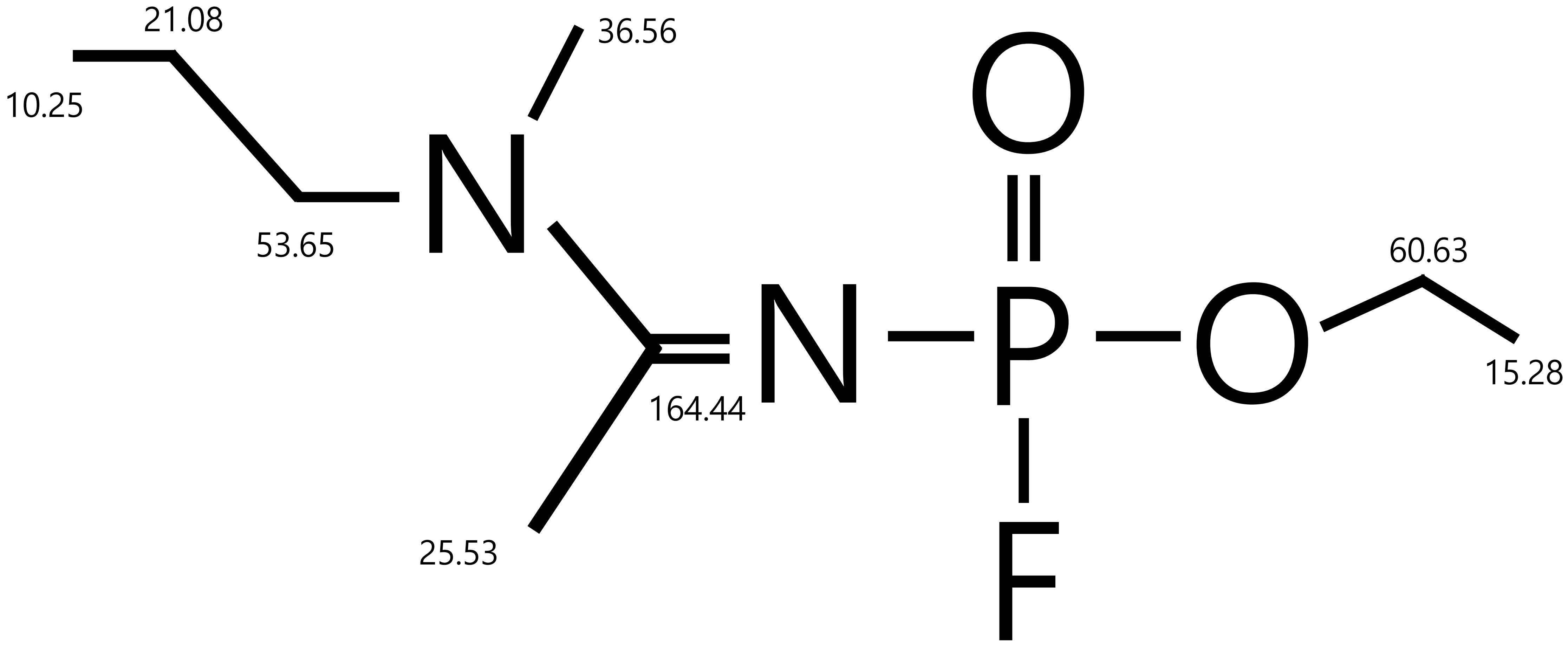

Figure S34. Structure 2131 and its ^{13}C chemical shift

2132 C

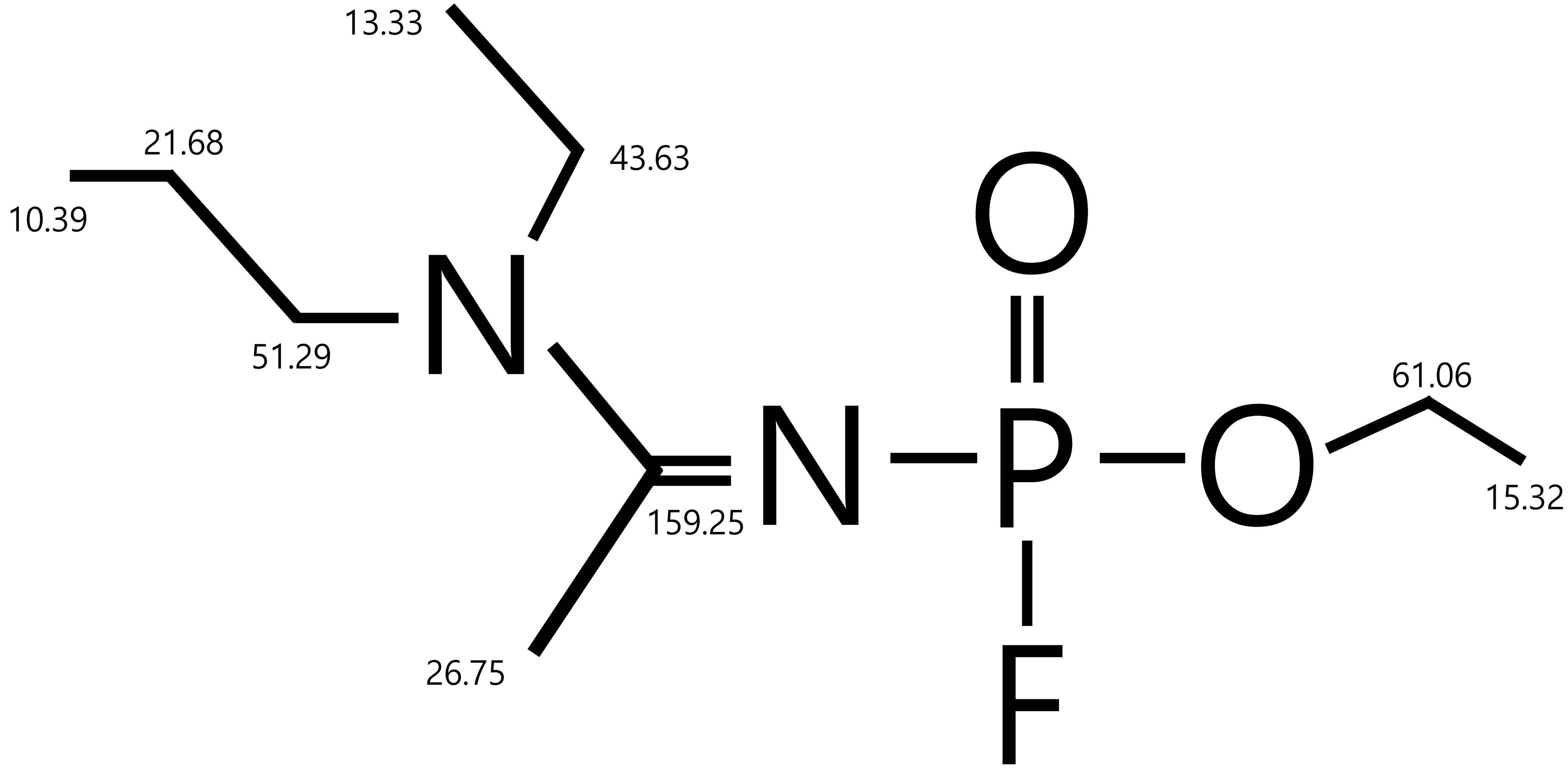

Figure S35. Structure 2132 and its ^{13}C chemical shift

2133 C

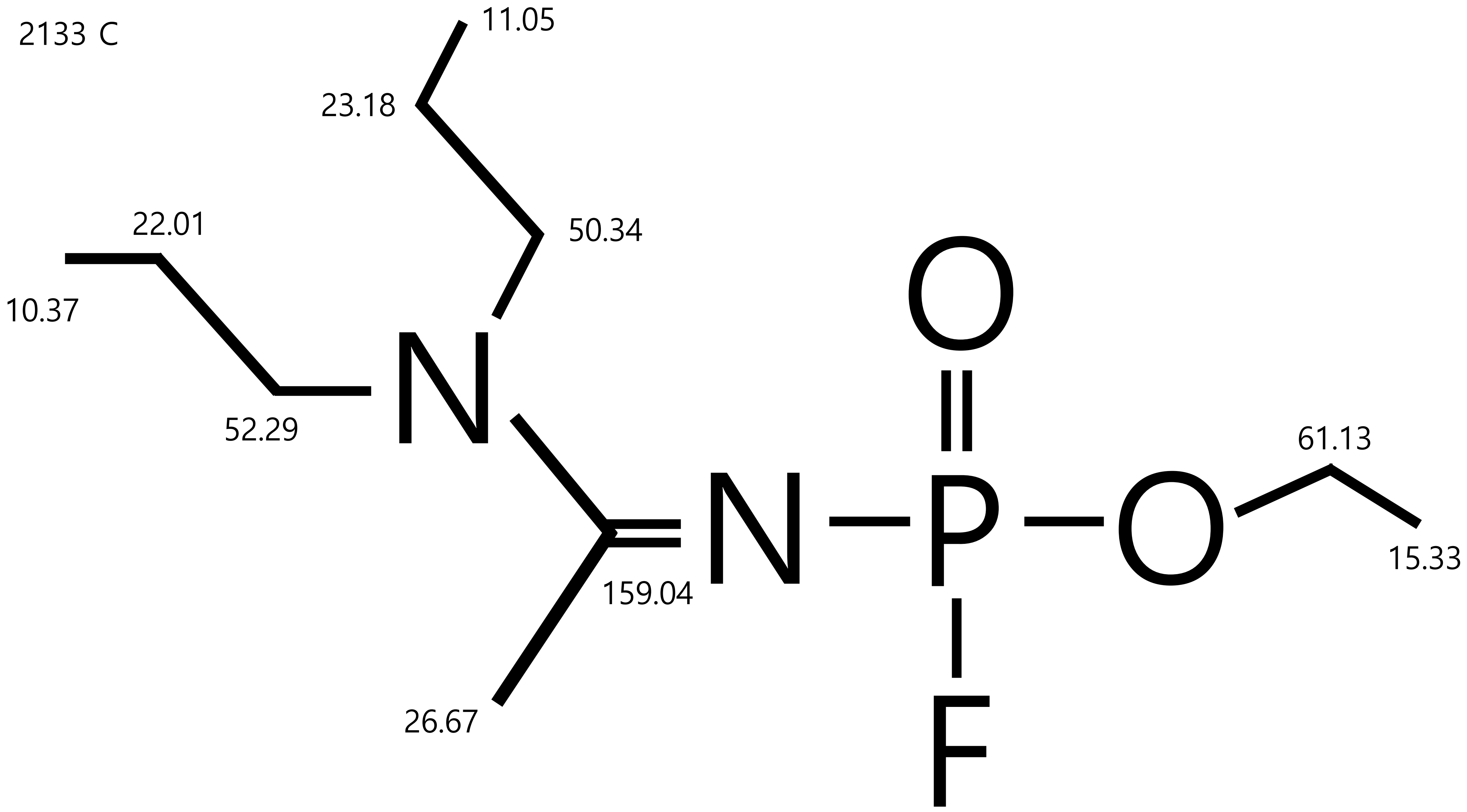

Figure S36. Structure 2133 and its ^{13}C chemical shift

2211 C

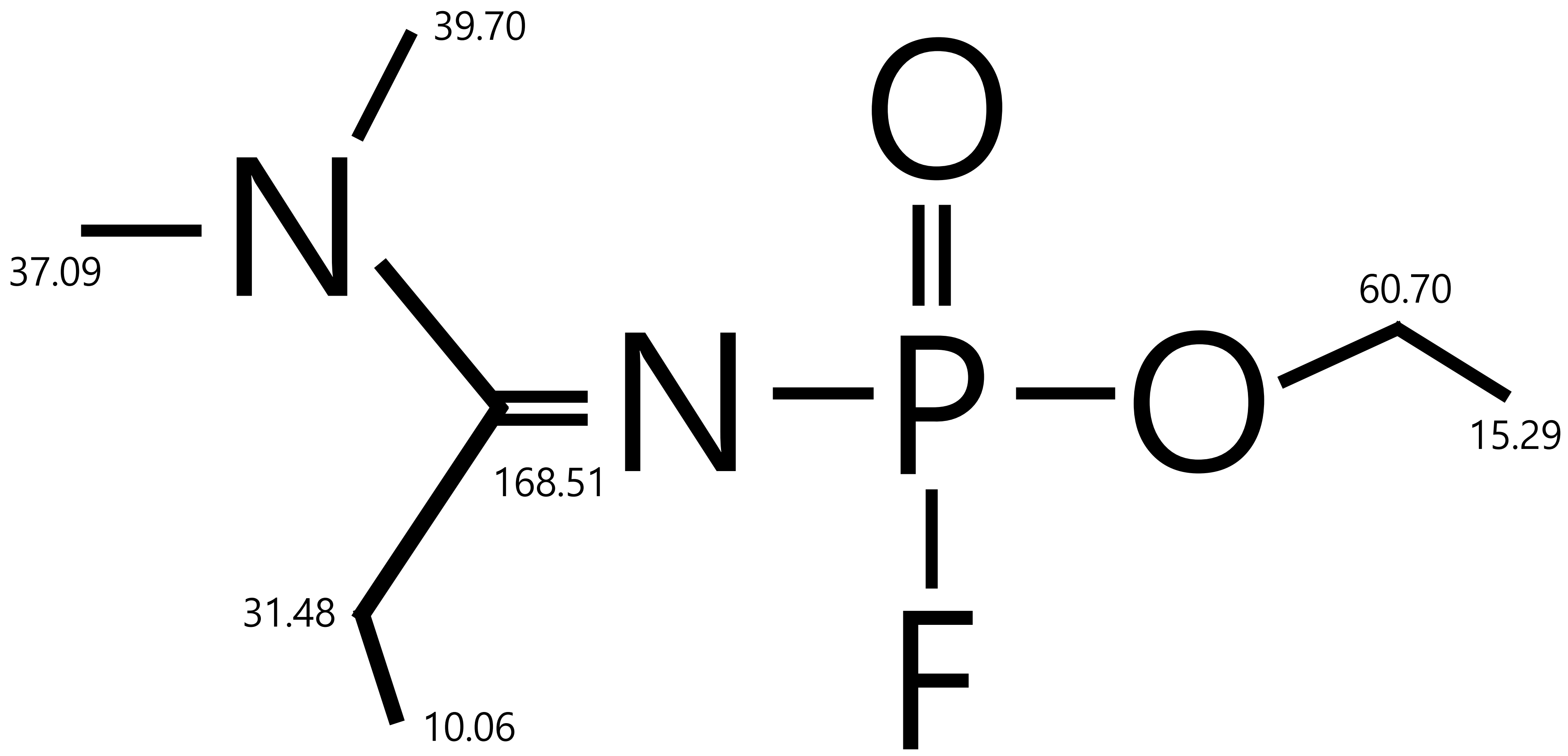

Figure S37. Structure 2211 and its ^{13}C chemical shift

2212 C

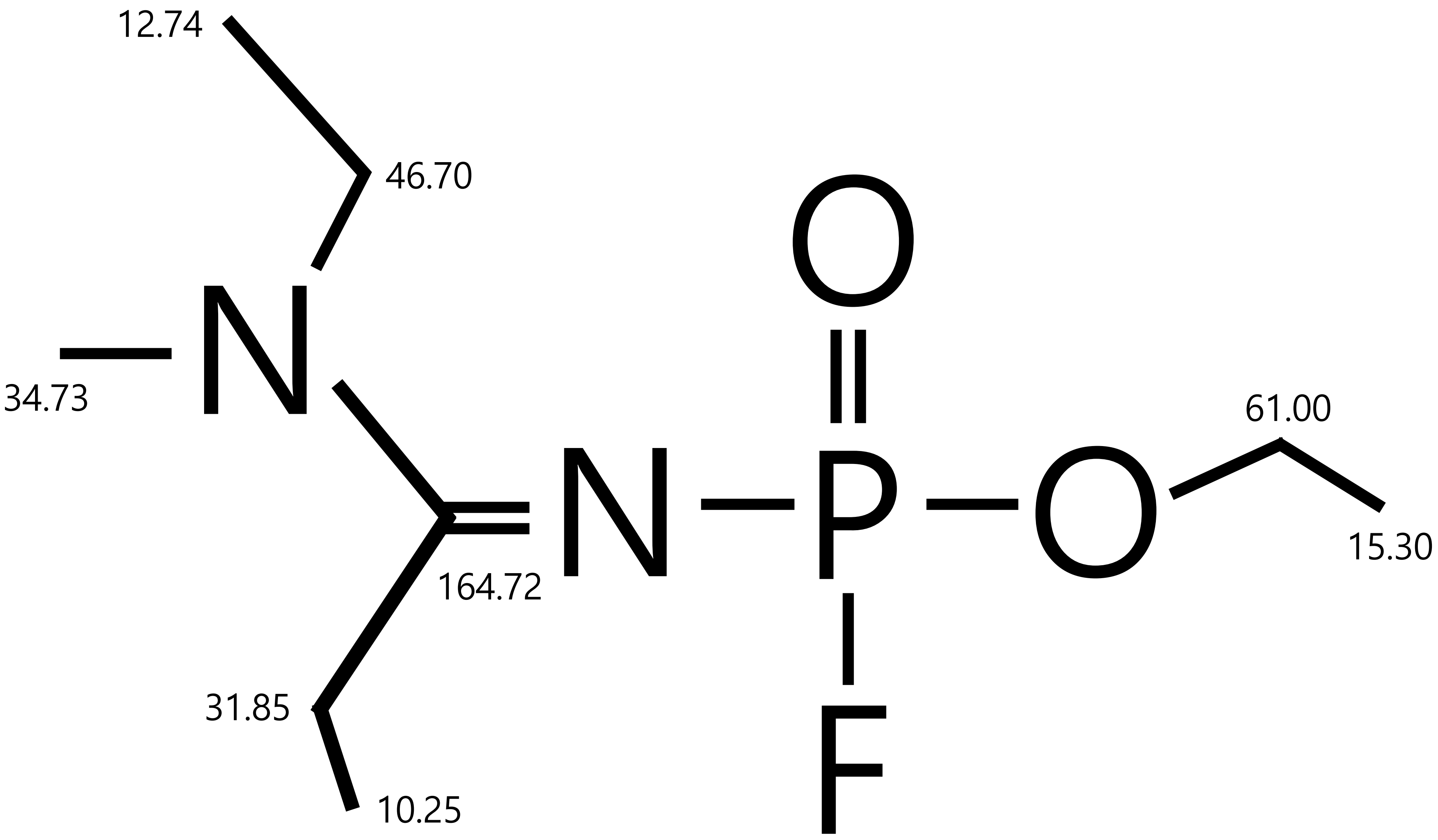

Figure S38. Structure 2212 and its ^{13}C chemical shift

2213 C

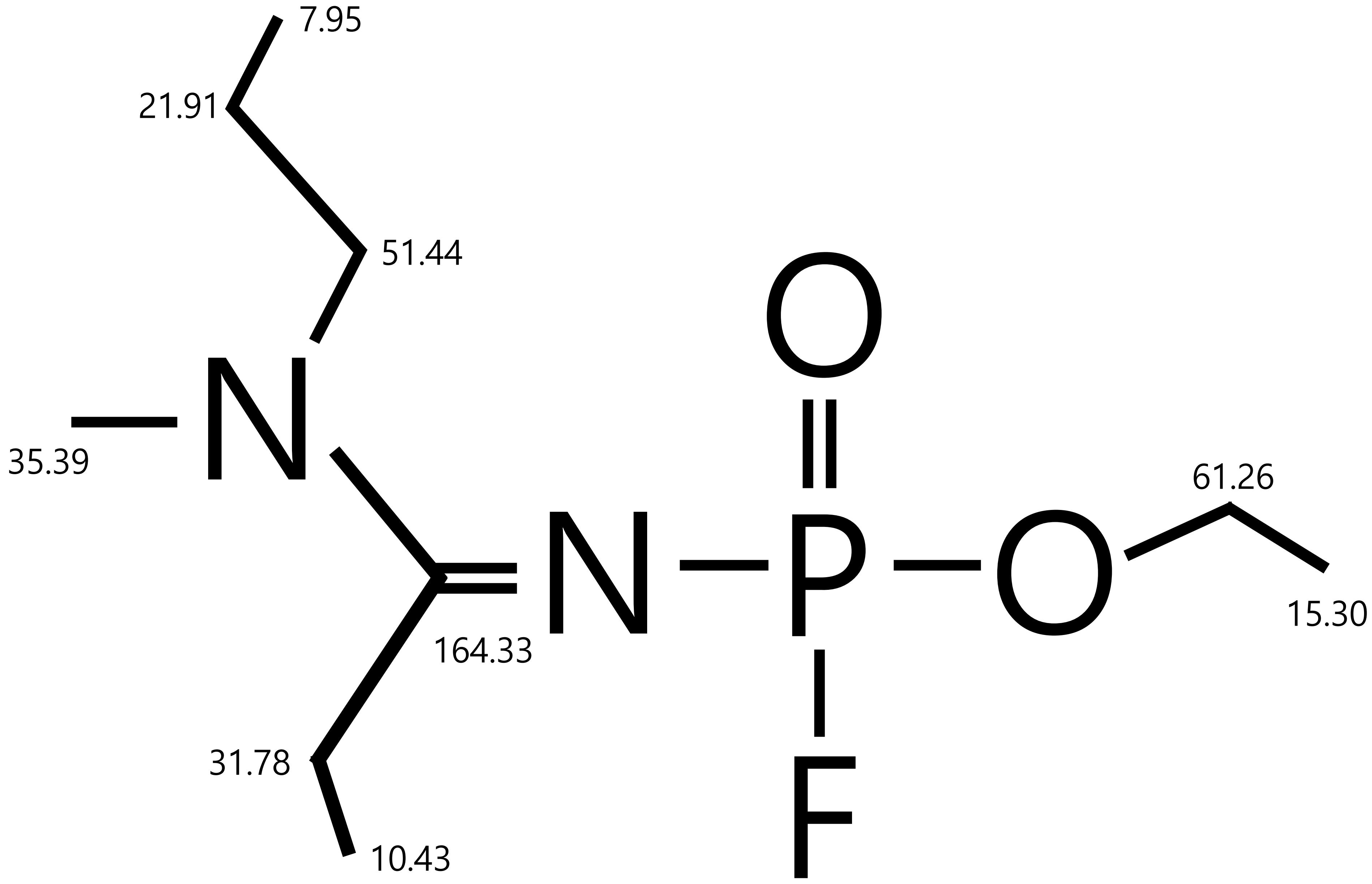

Figure S39. Structure 2213 and its ^{13}C chemical shift

2221 C

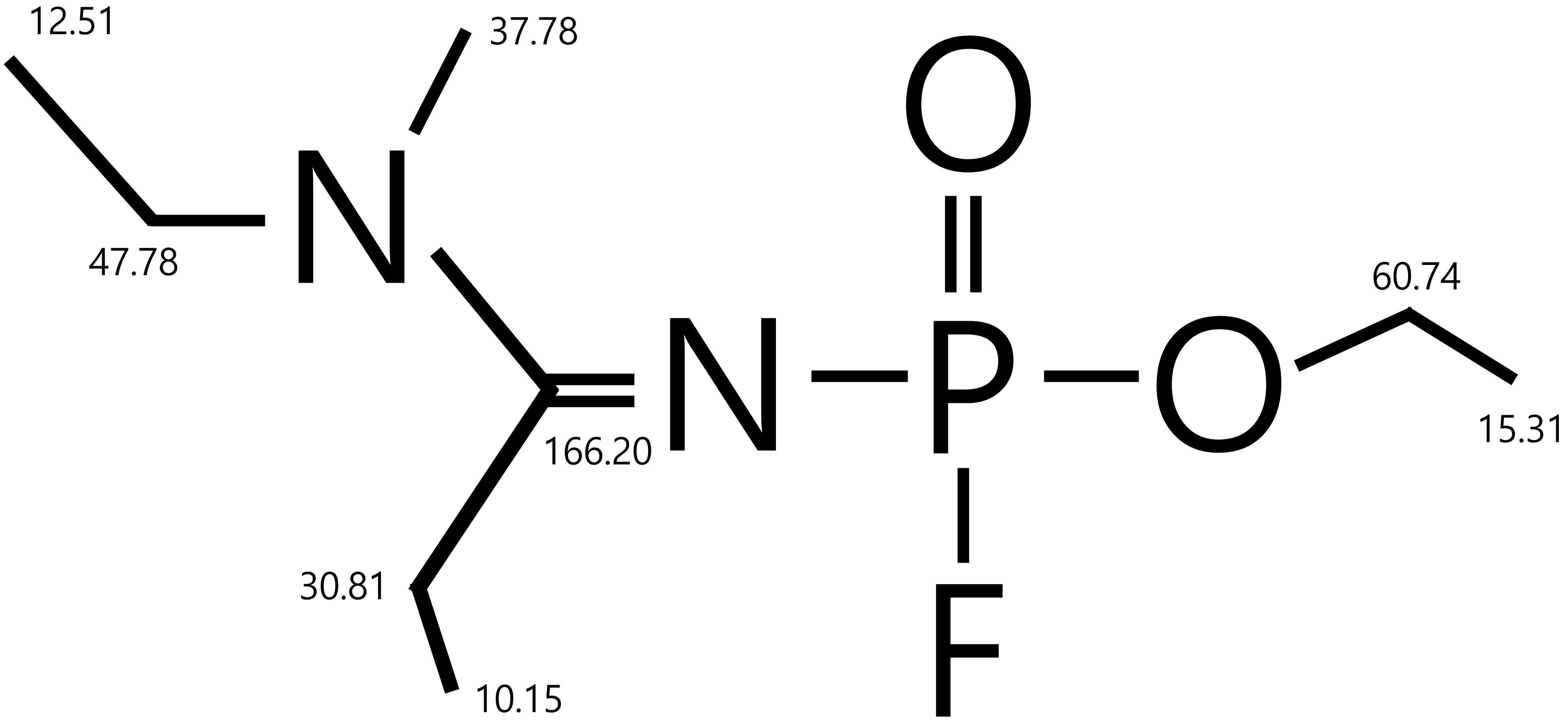

Figure S40. Structure 2221 and its ^{13}C chemical shift

2222 C

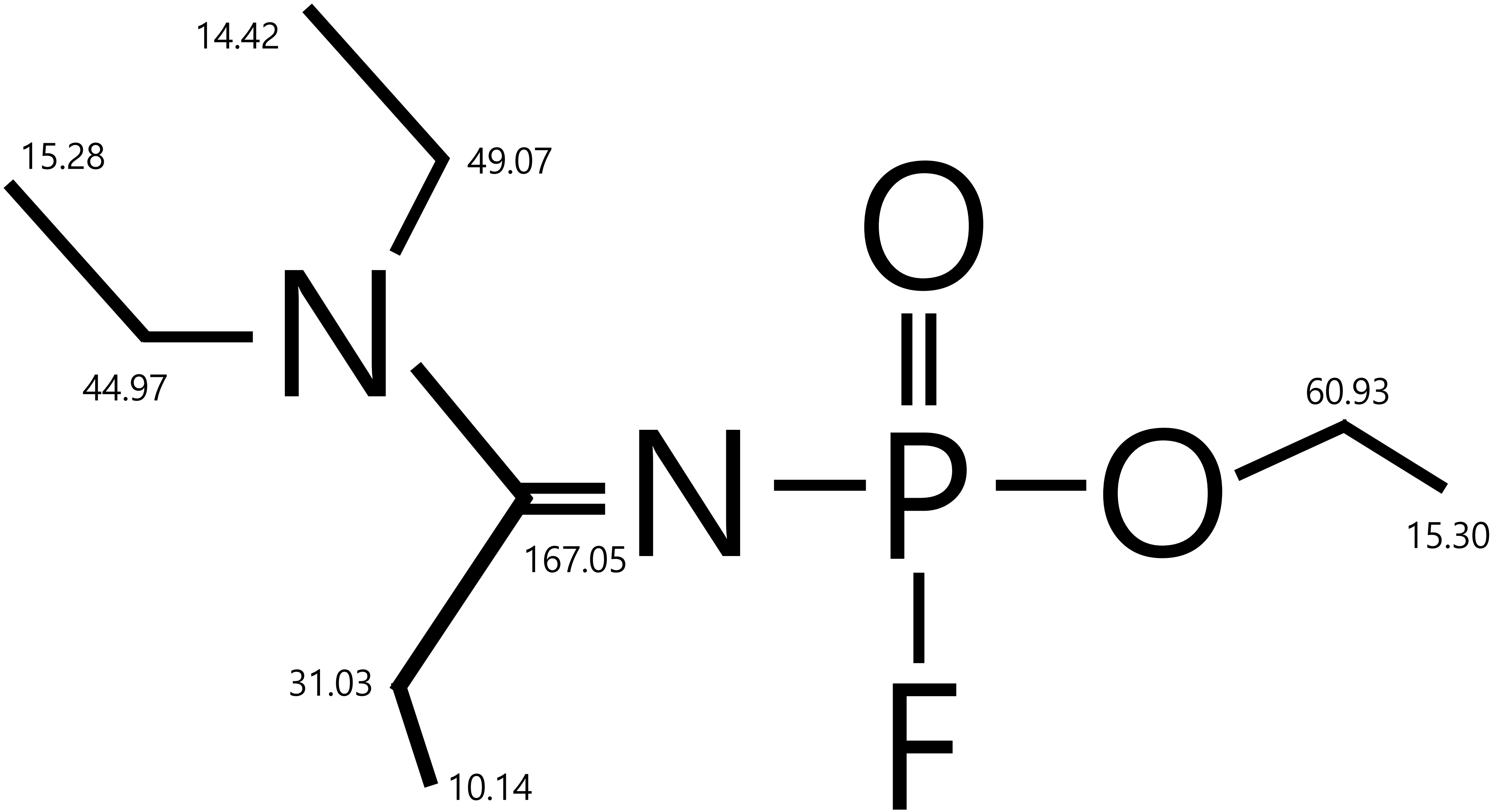

Figure S41. Structure 2222 and its ^{13}C chemical shift

2223 C

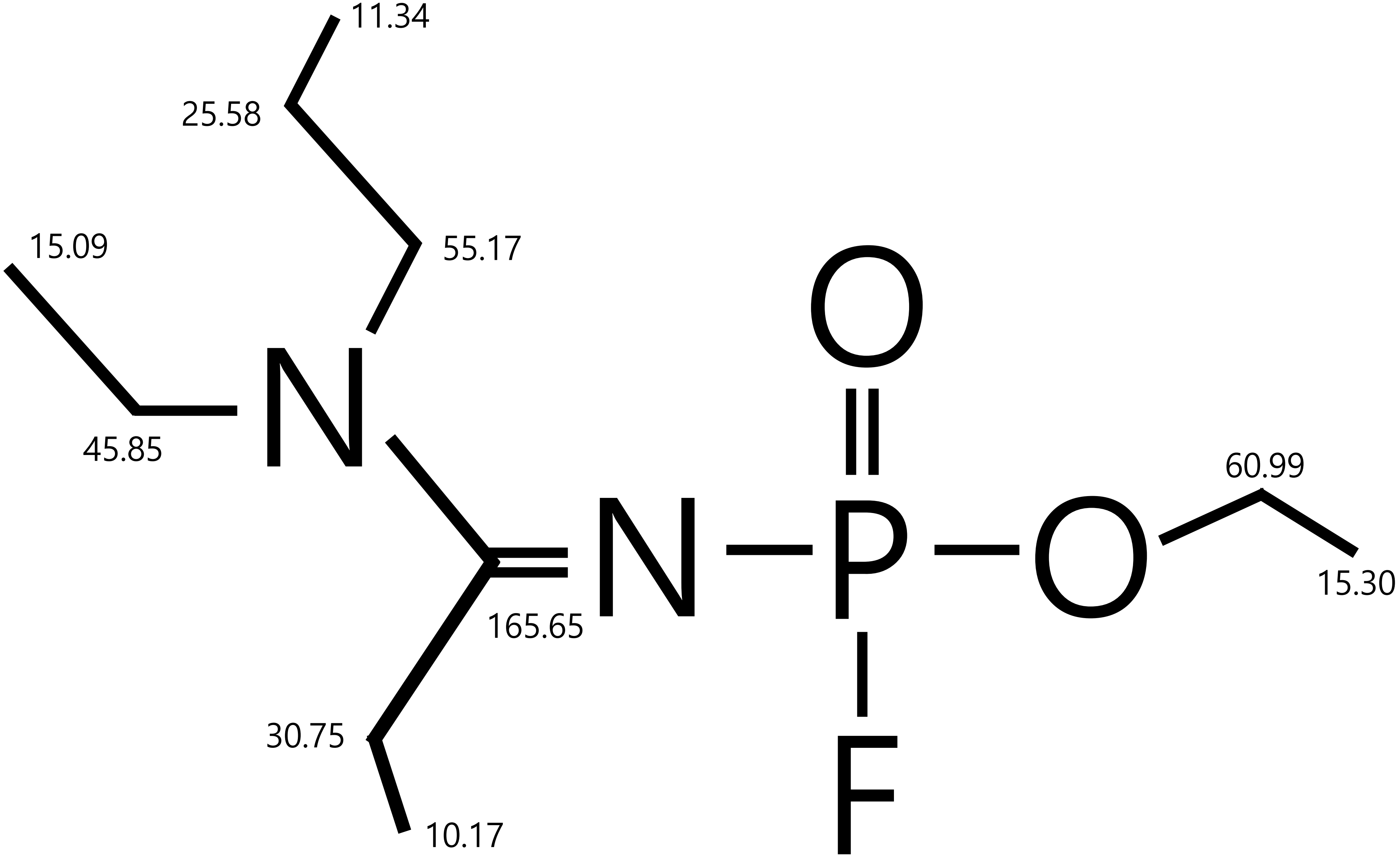

Figure S42. Structure 2223 and its ^{13}C chemical shift

2231 C

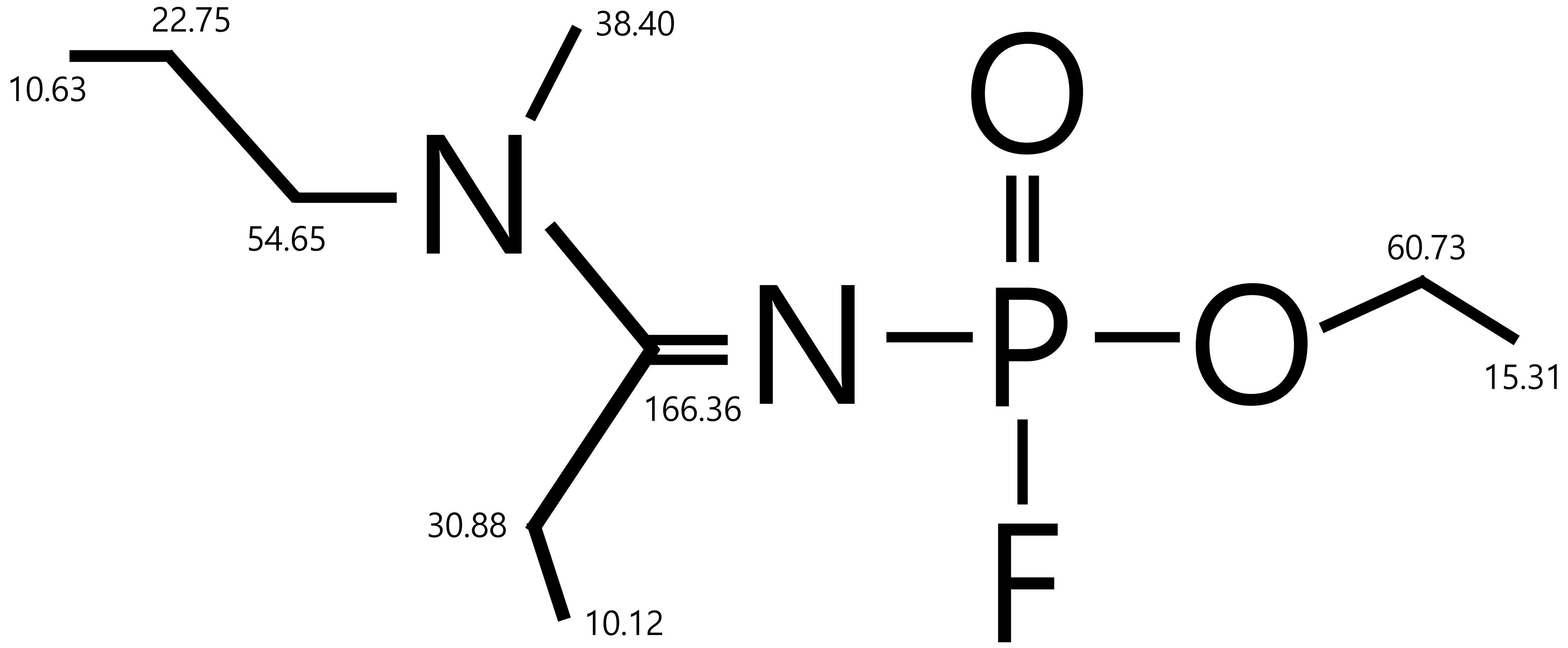

Figure S43. Structure 2231 and its ^{13}C chemical shift

2232 C

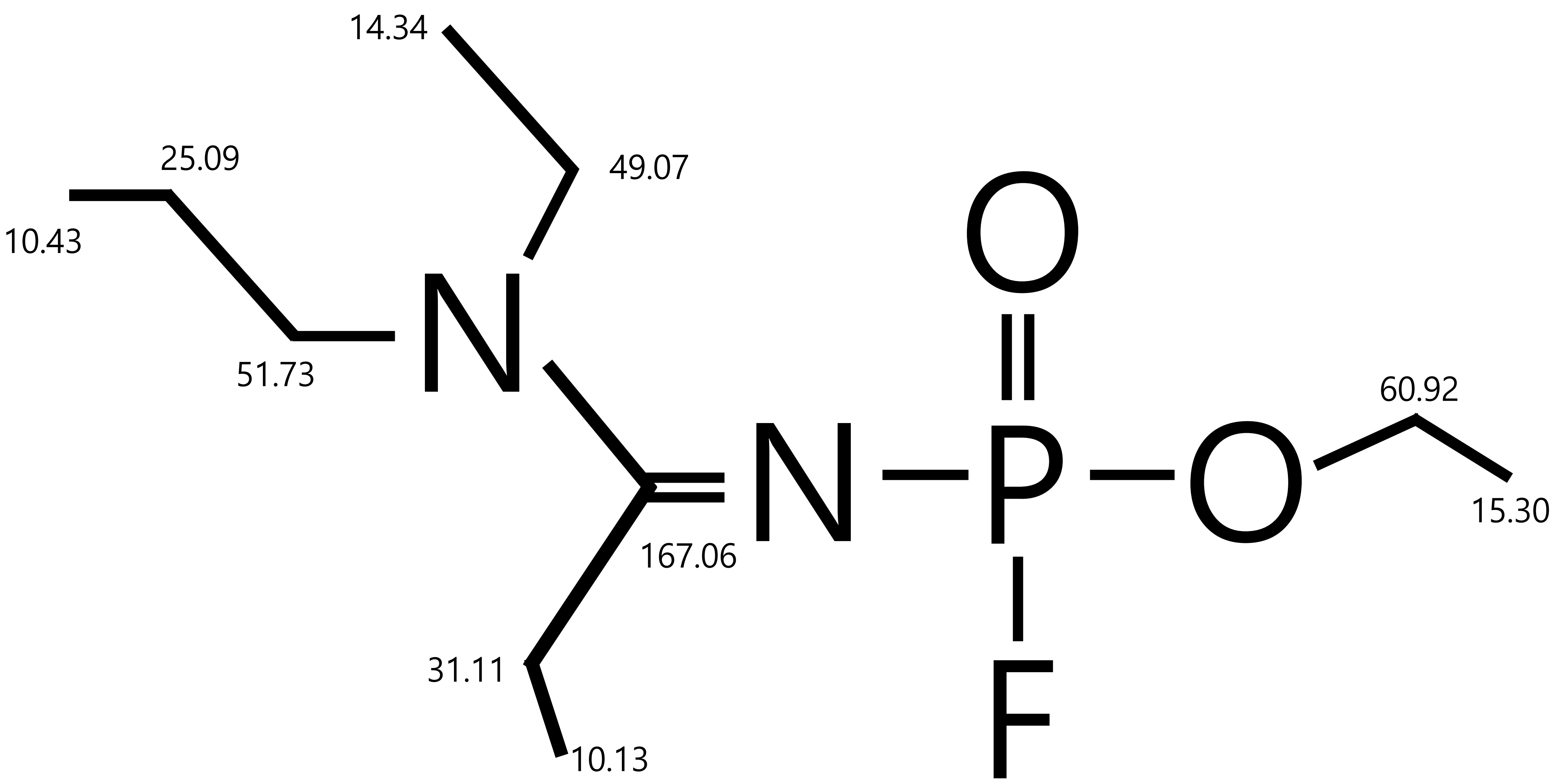

Figure S44. Structure 2232 and its 13C chemical shift

2233 C

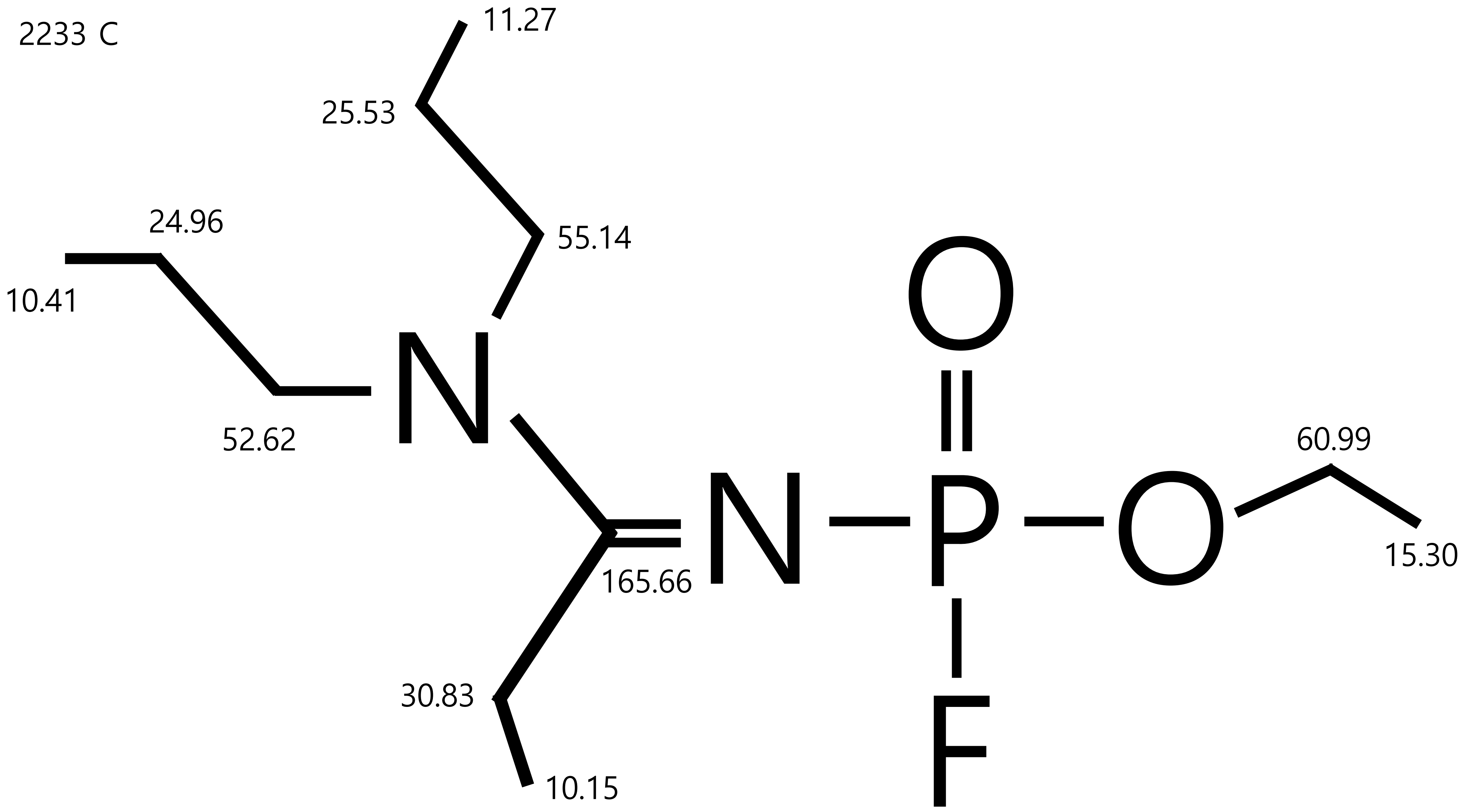

Figure S45. Structure 2233 and its ^{13}C chemical shift

2311 C

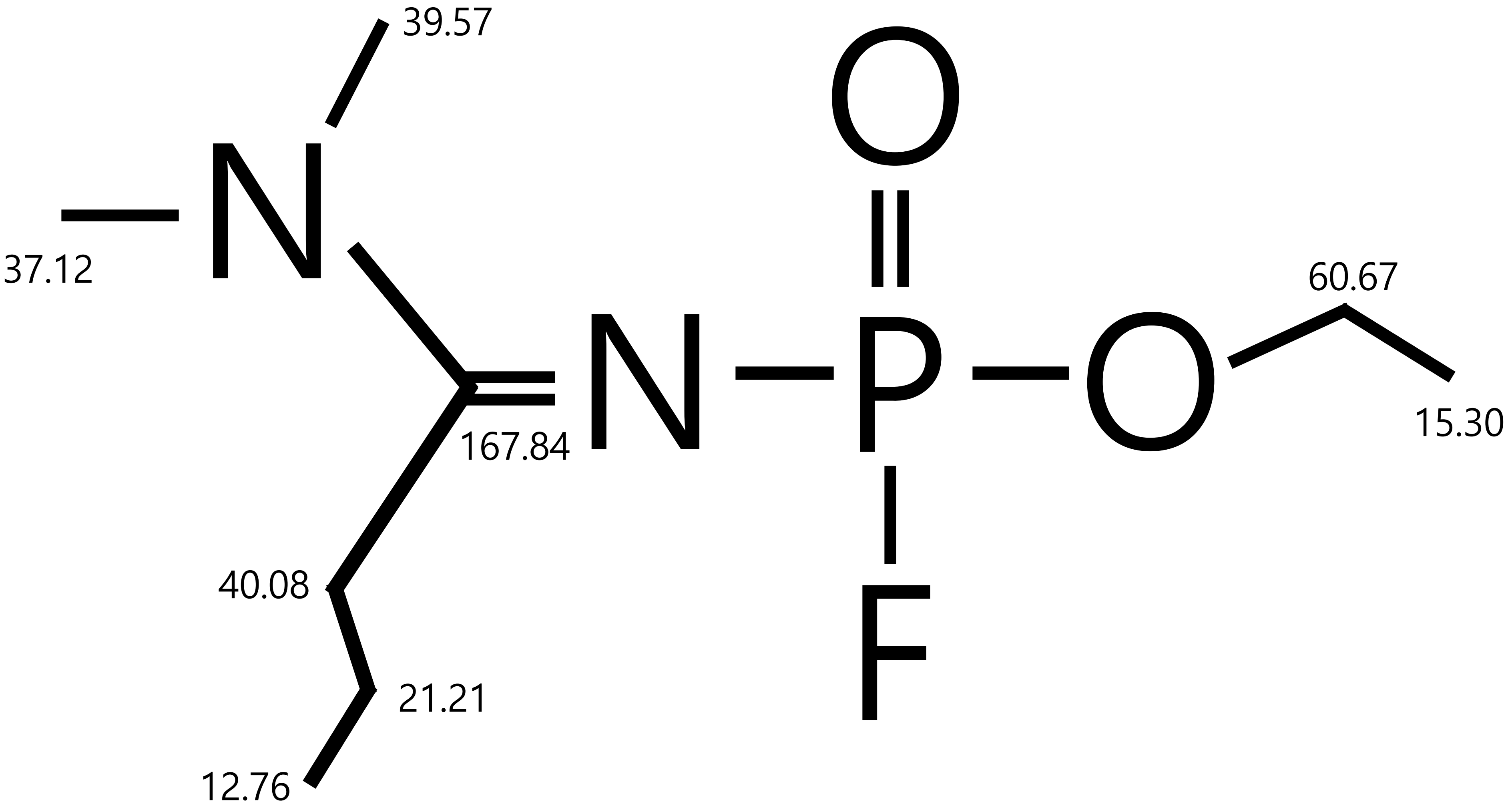

Figure S46. Structure 2311 and its ^{13}C chemical shift

2312 C

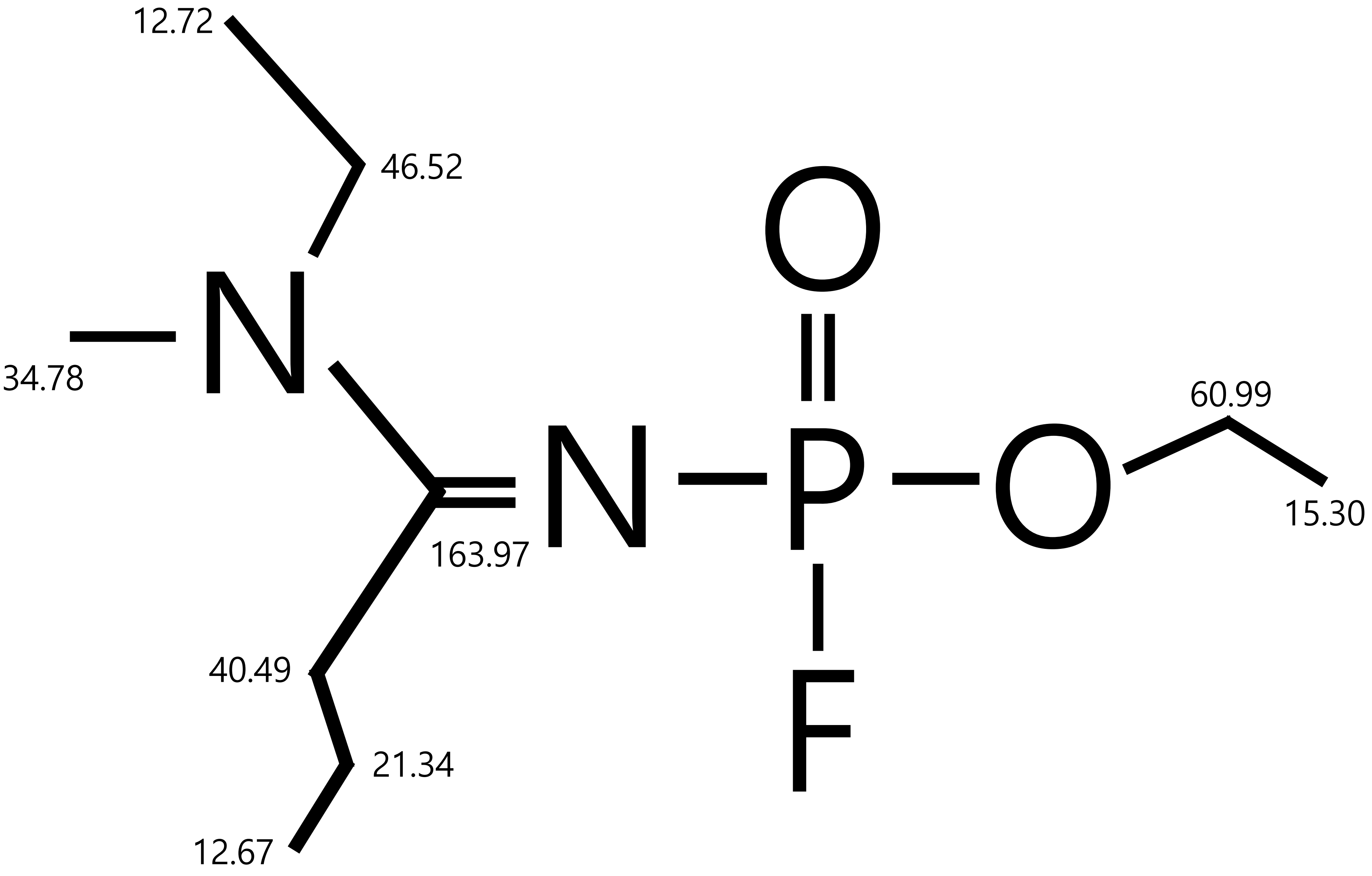

Figure S47. Structure 2312 and its ^{13}C chemical shift

2313 C

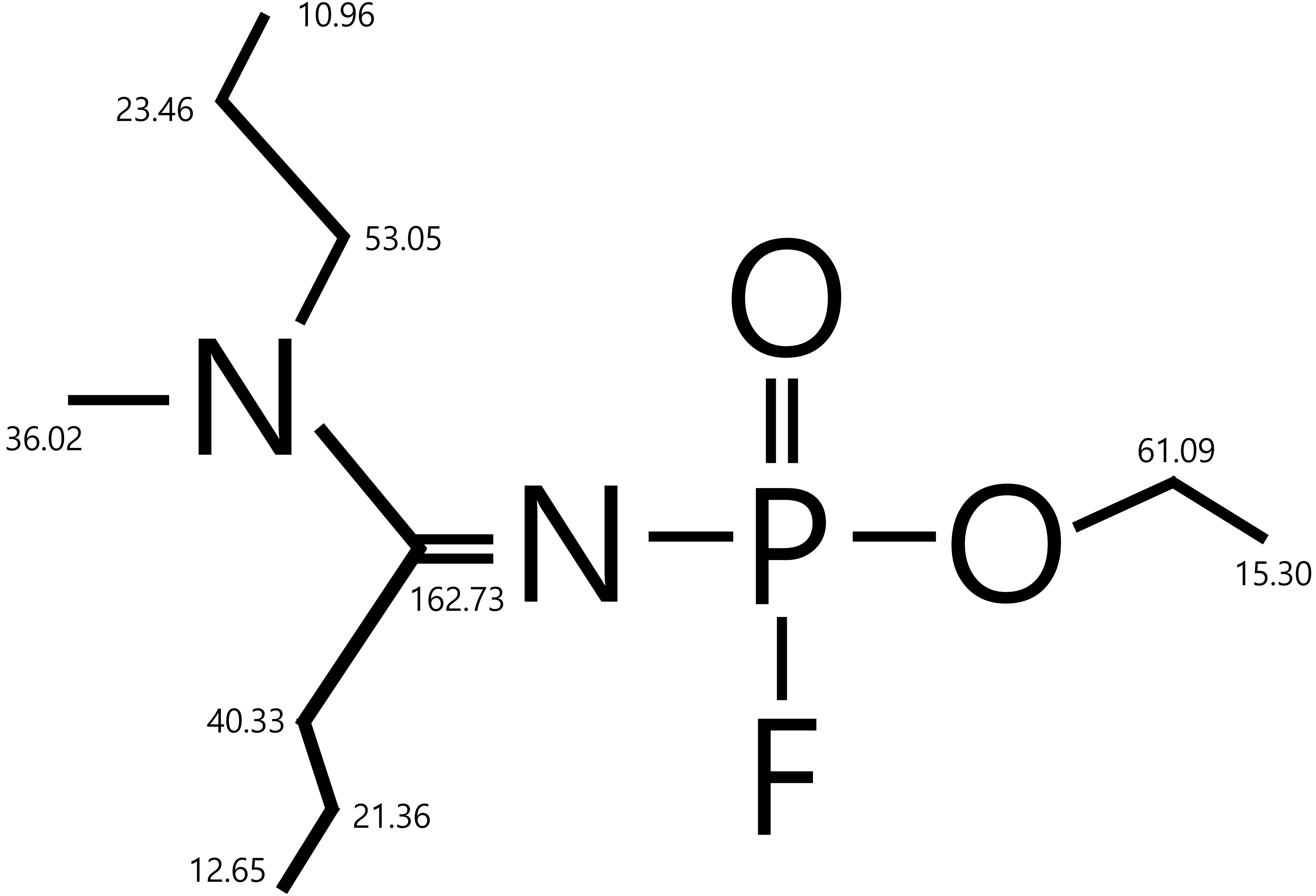

Figure S48. Structure 2313 and its ^{13}C chemical shift

2321 C

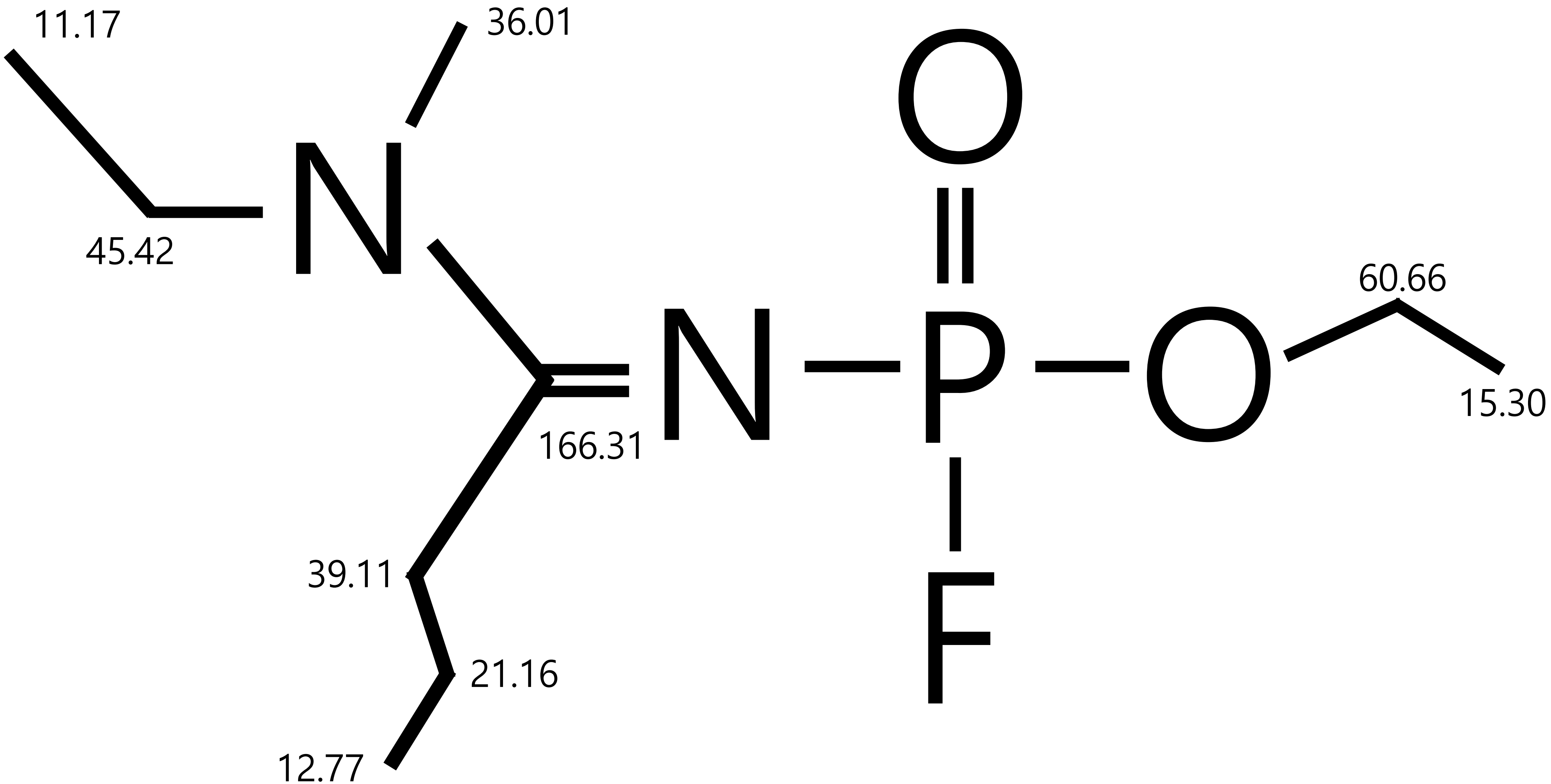

Figure S49. Structure 2321 and its ^{13}C chemical shift

2322 C

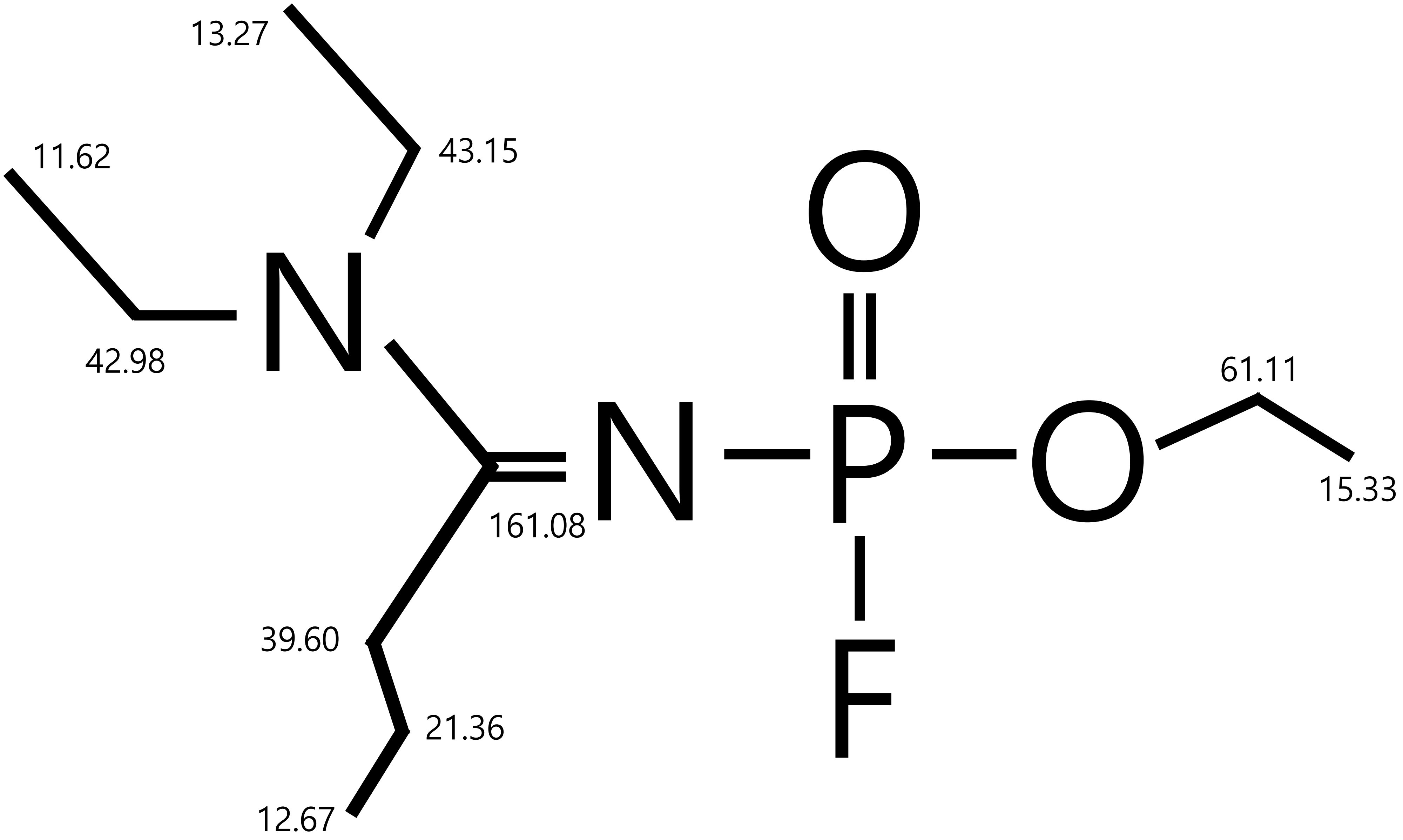

Figure S50. Structure 2322 and its ^{13}C chemical shift

2323 C

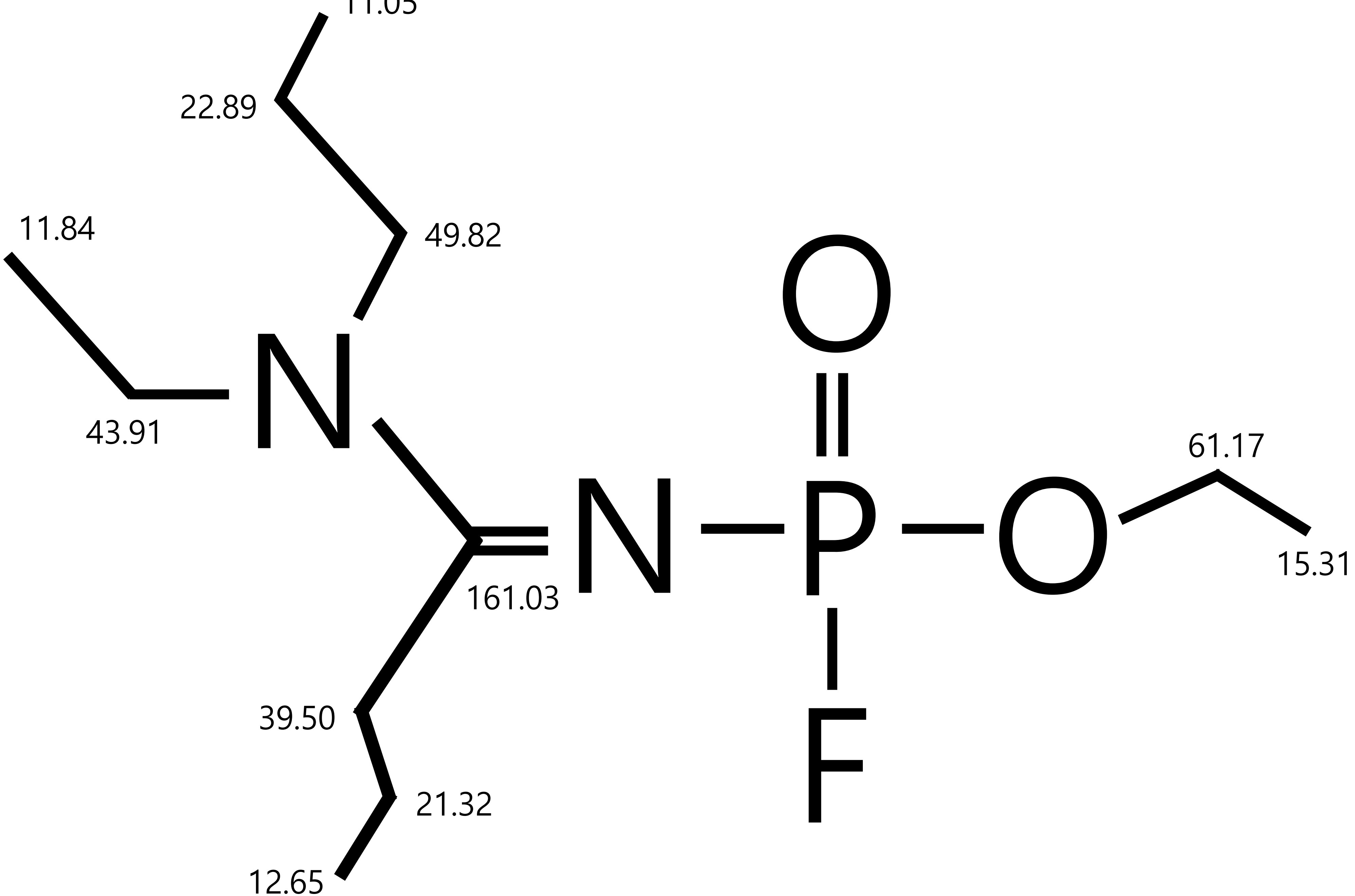

Figure S51. Structure 2323 and its ^{13}C chemical shift

2331 C

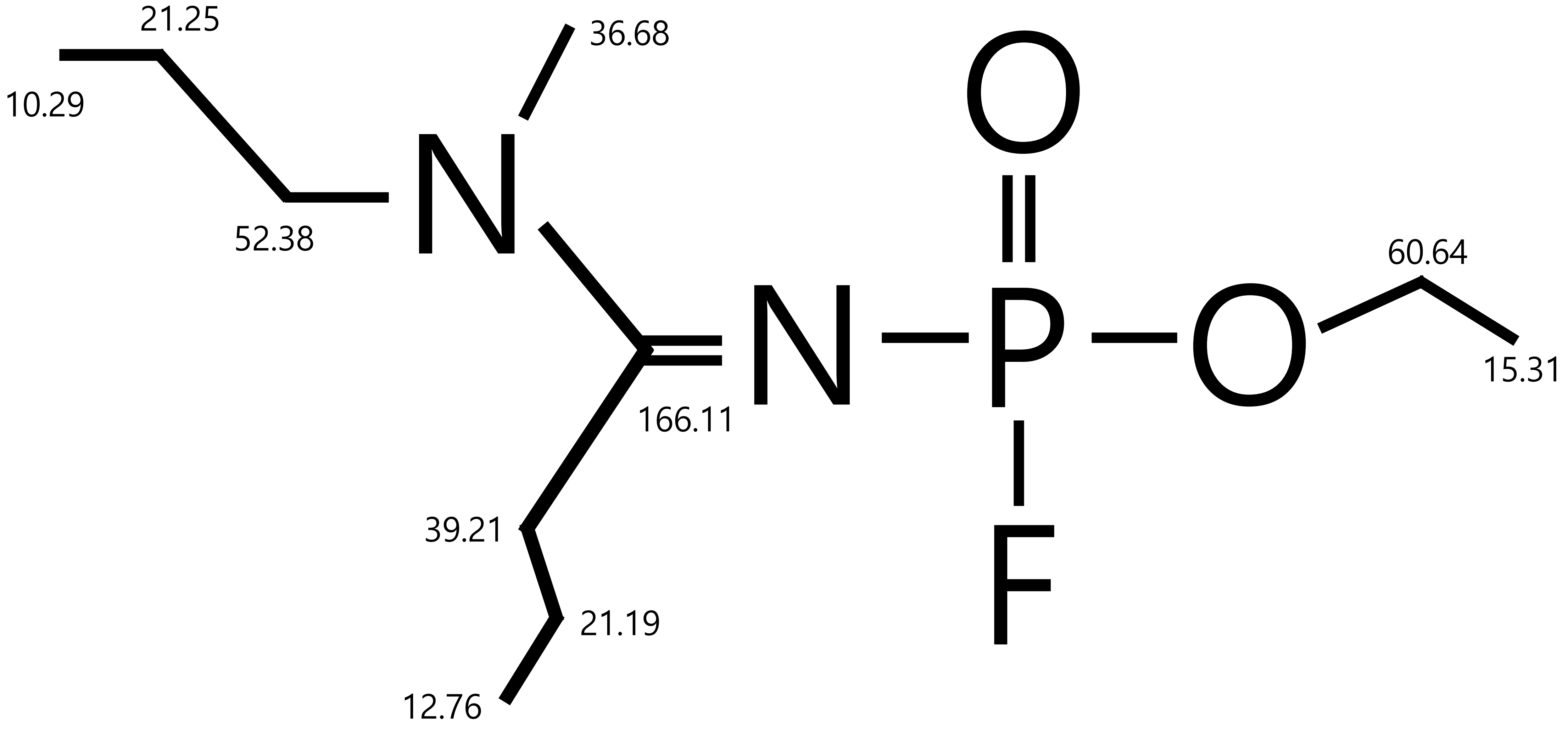

Figure S52. Structure 2331 and its ^{13}C chemical shift

2332 C

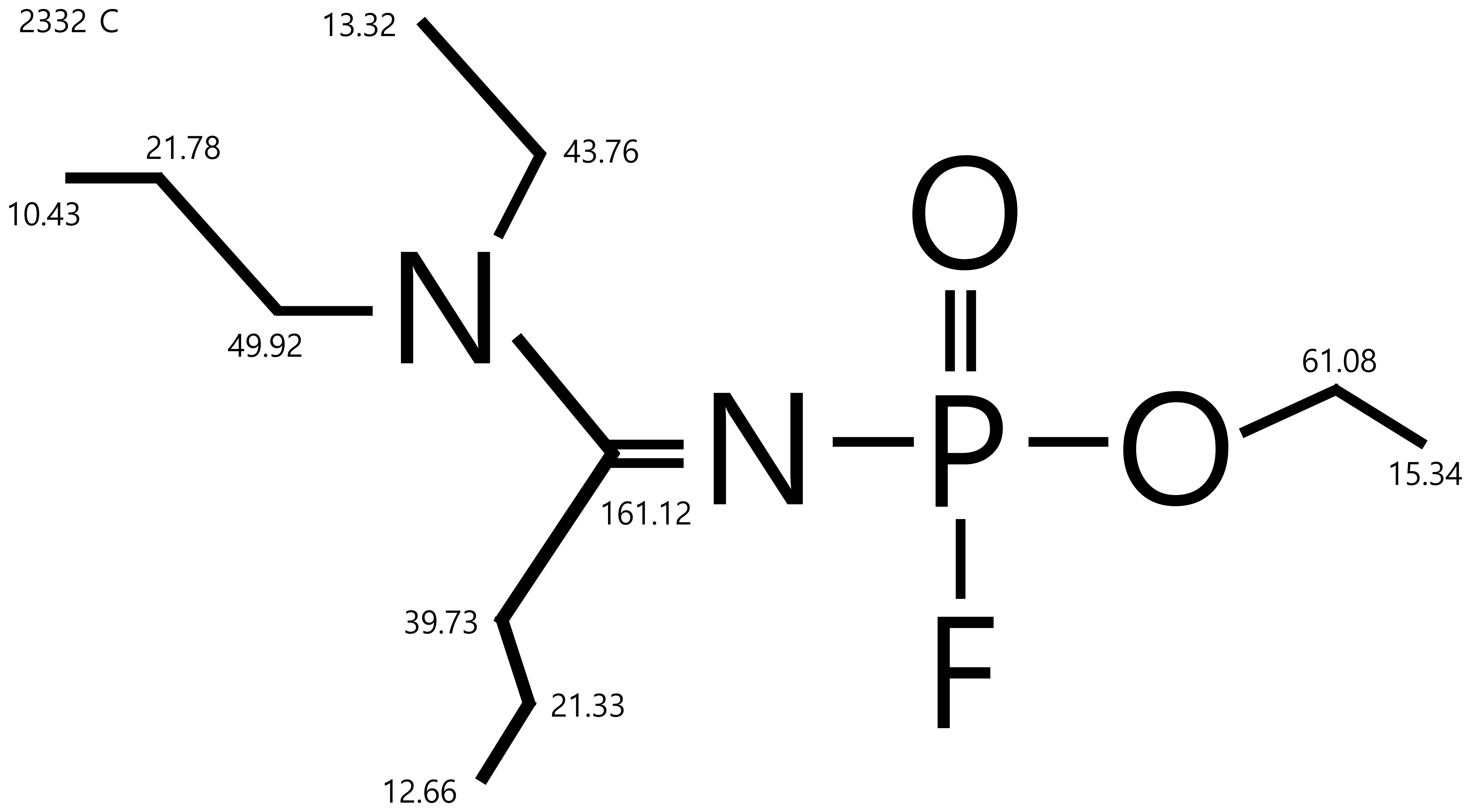

Figure S53. Structure 2332 and its ^{13}C chemical shift

2333 C

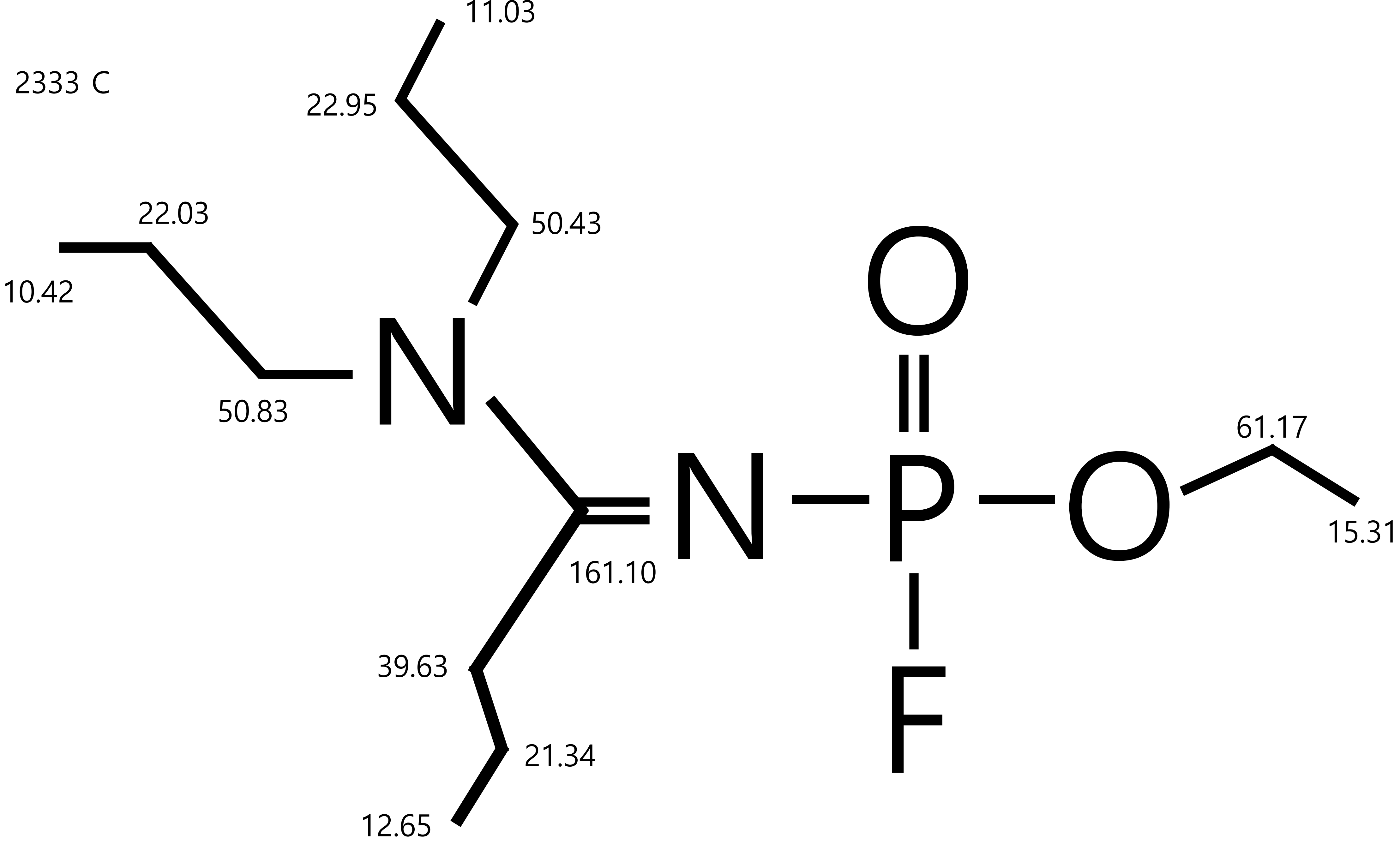

Figure S54. Structure 2333 and its ^{13}C chemical shift

3111 C

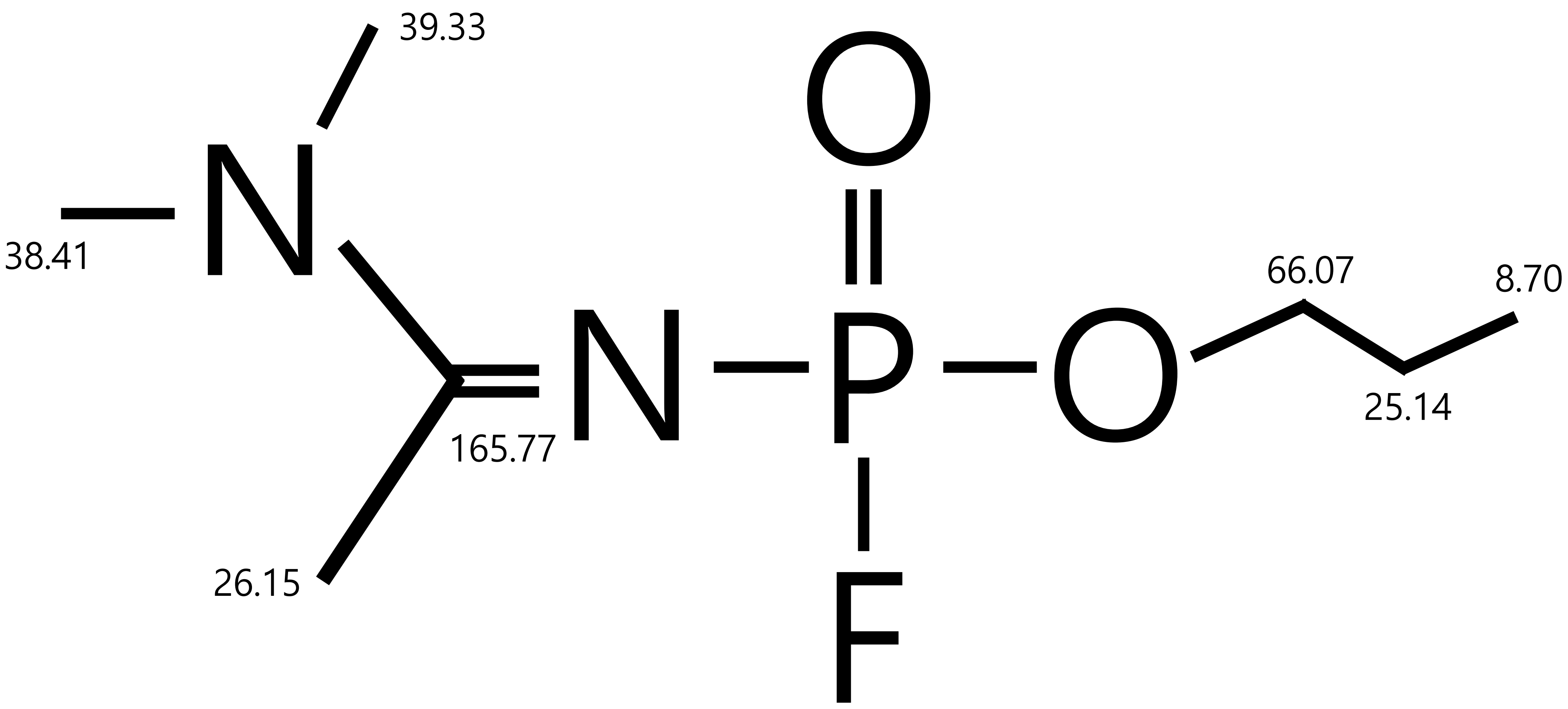

Figure S55. Structure 3111 and its ^{13}C chemical shift

3112 C

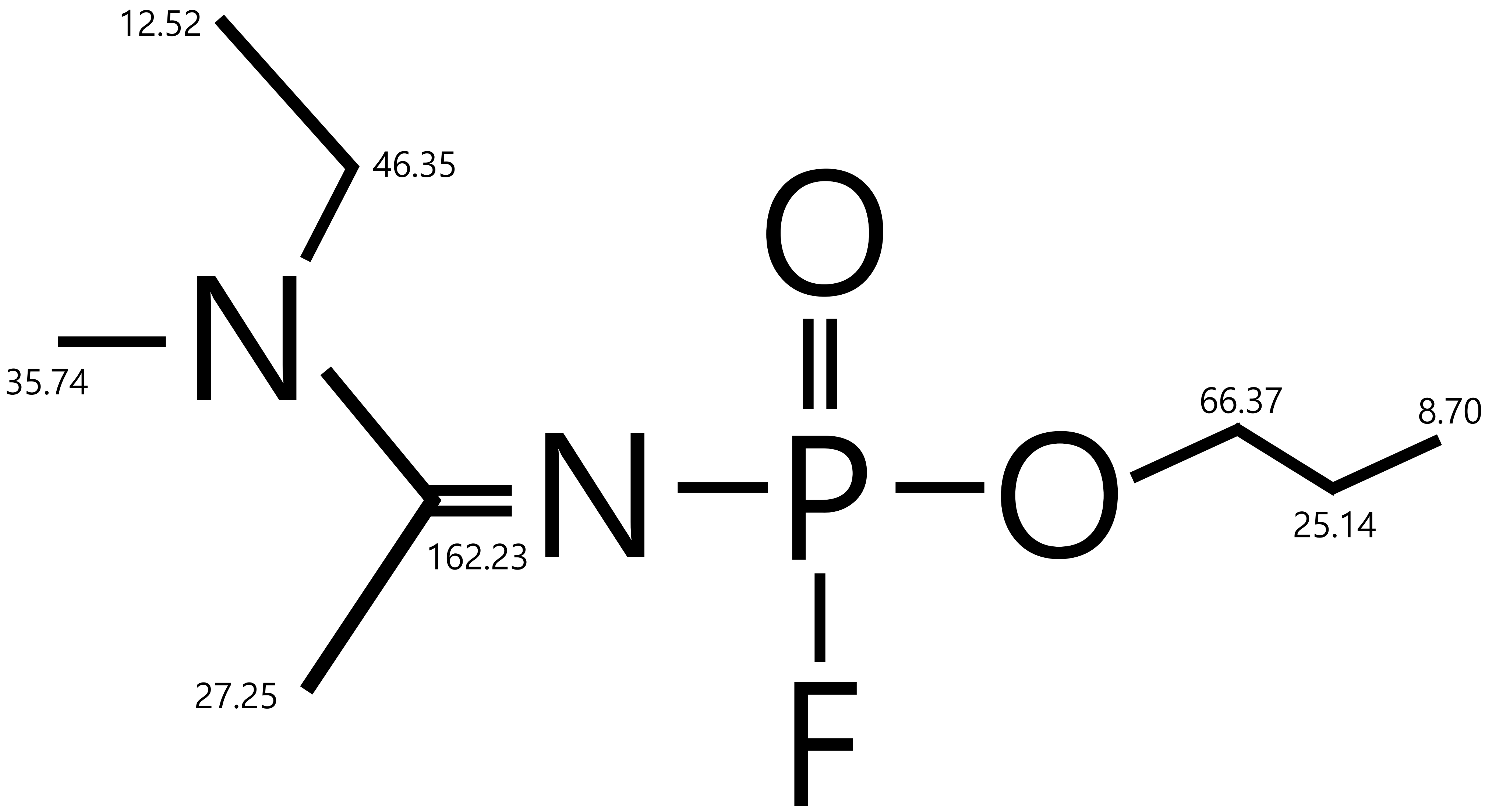

Figure S56. Structure 3112 and its ^{13}C chemical shift

3113 C

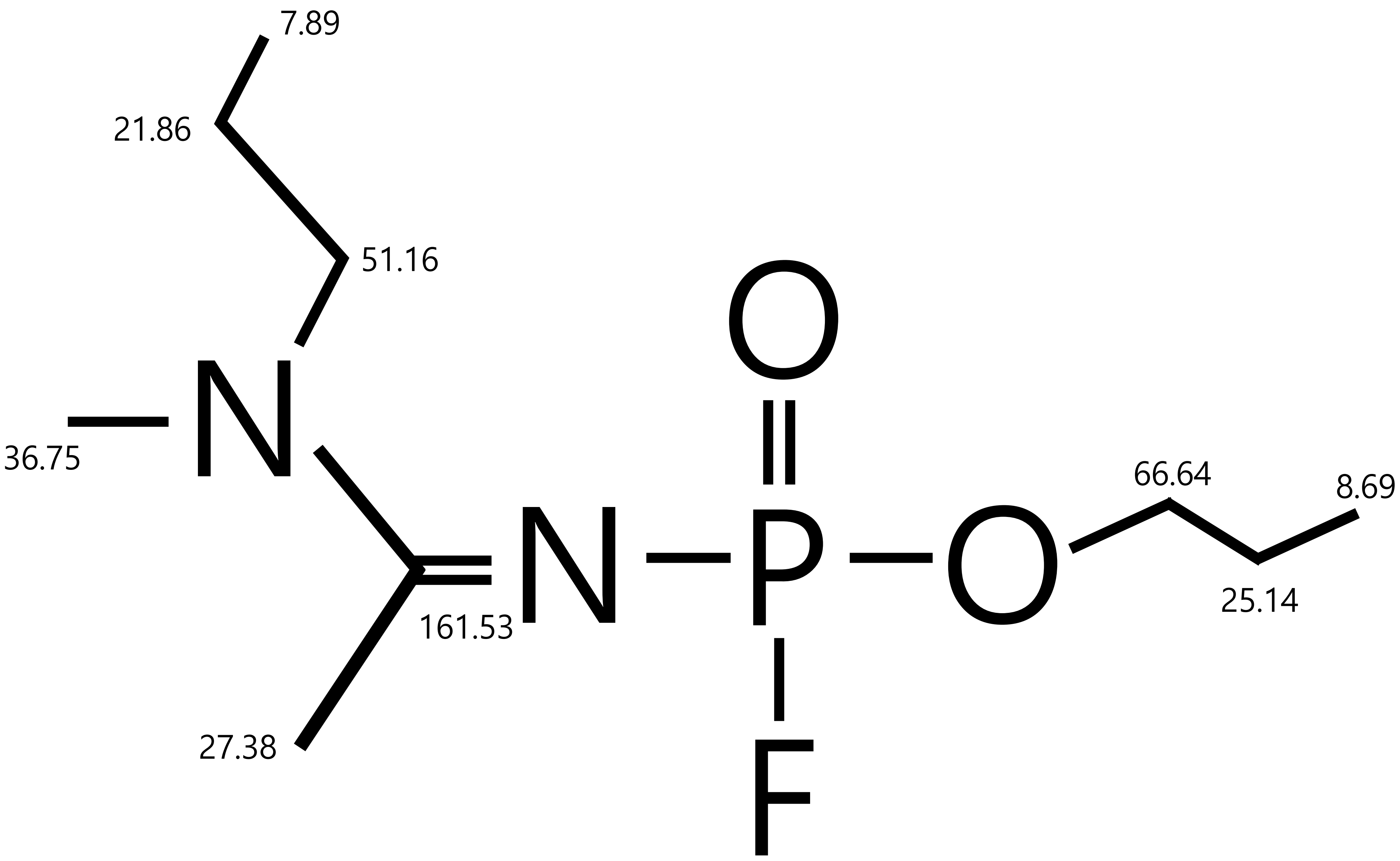

Figure S57. Structure 3113 and its ^{13}C chemical shift

3121 C

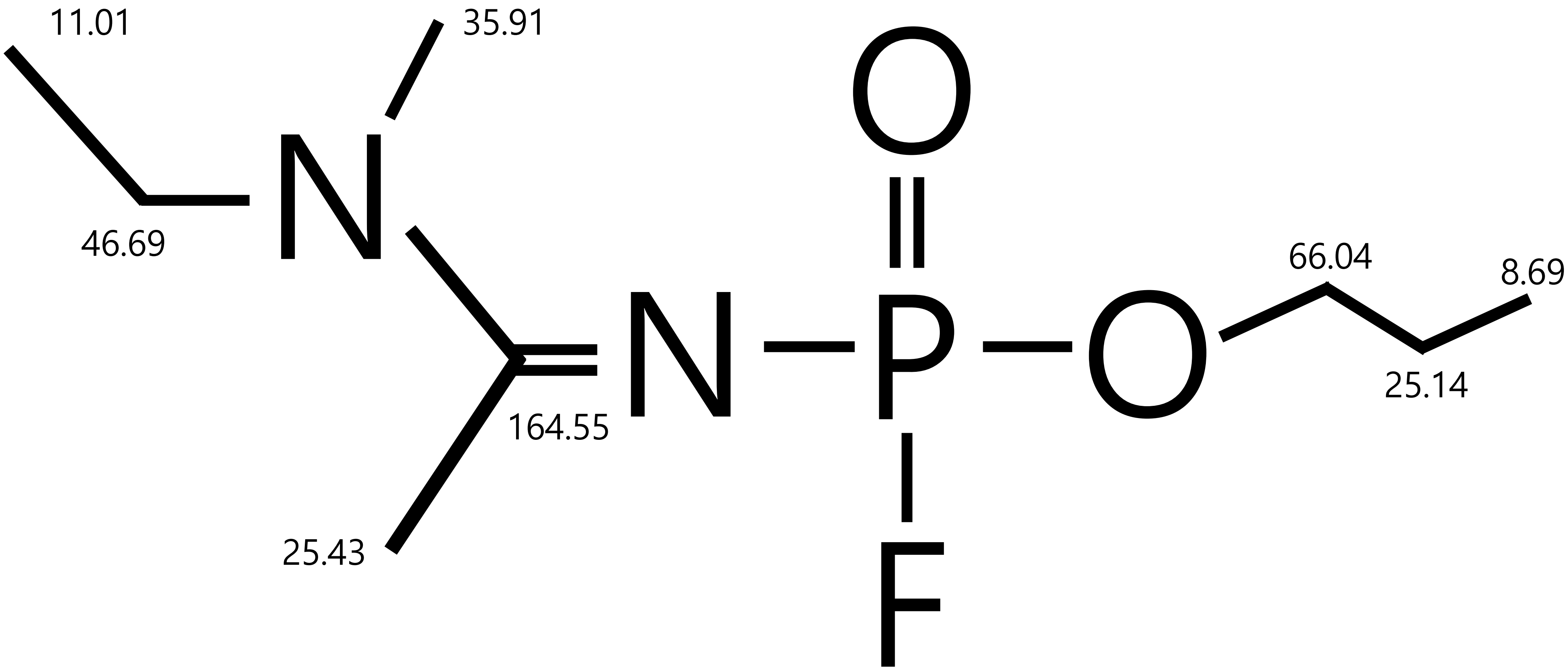

Figure S58. Structure 3121 and its ^{13}C chemical shift

3122 C

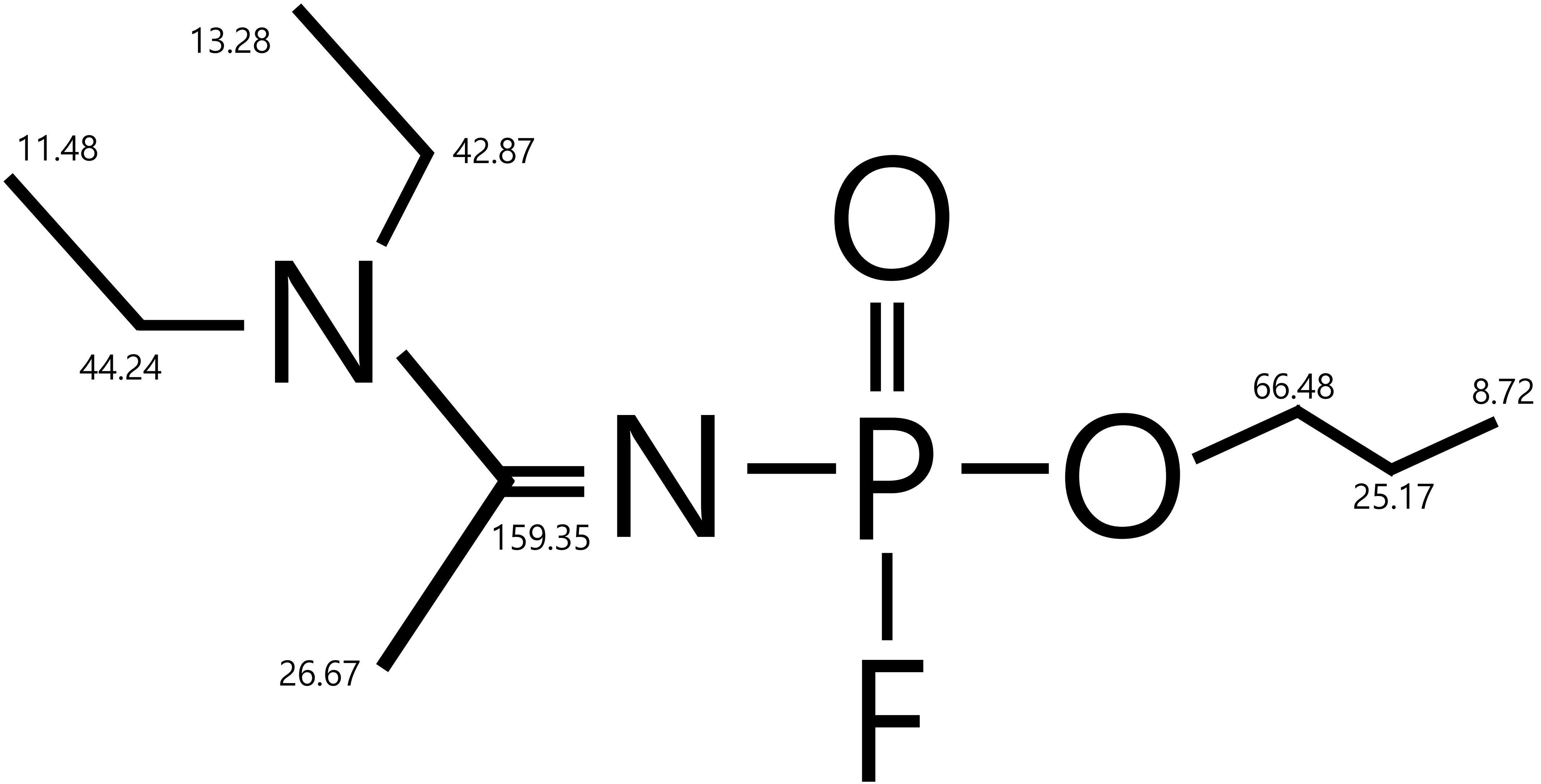

Figure S59. Structure 3122 and its ^{13}C chemical shift

3123 C

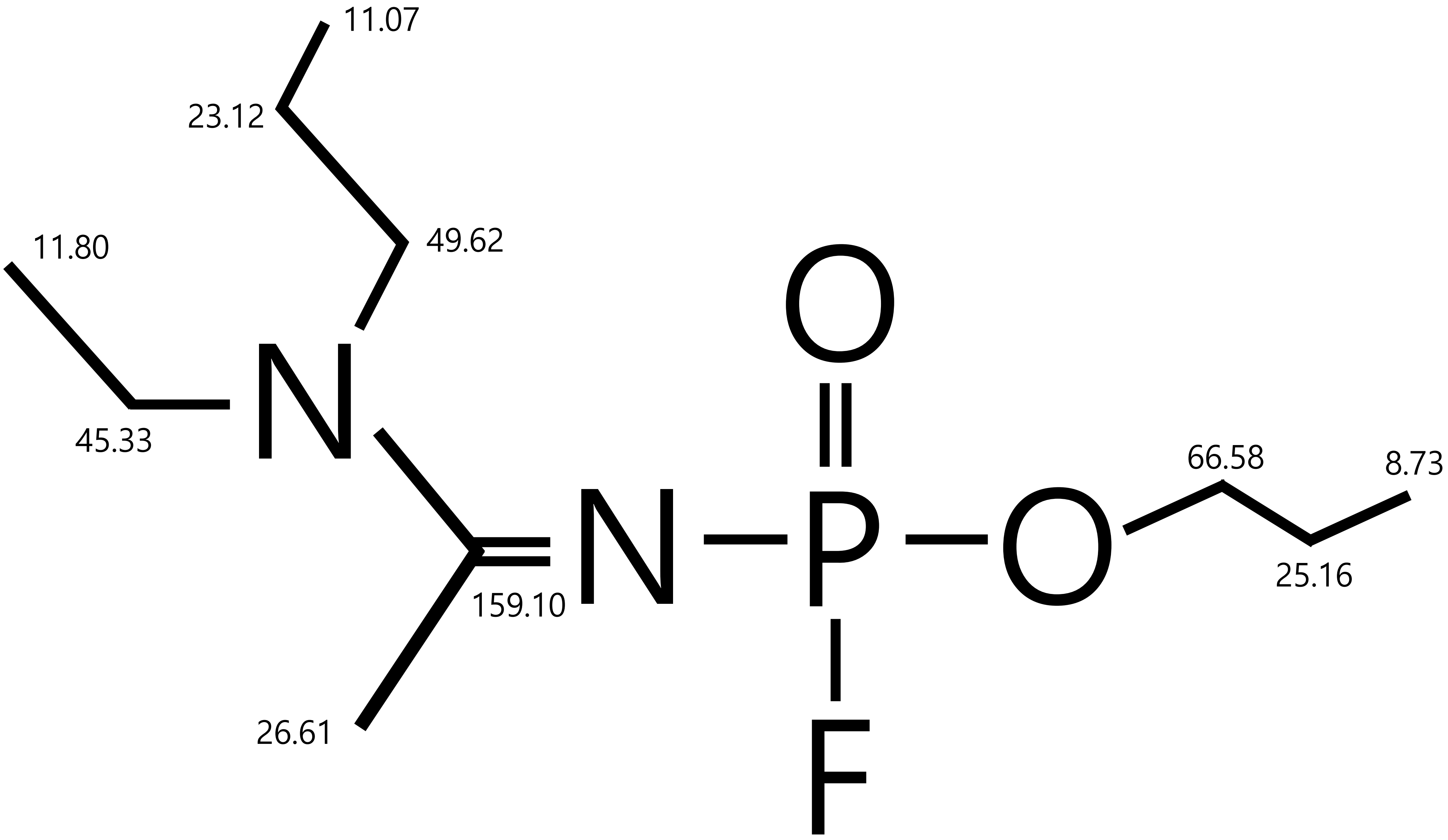

Figure S60. Structure 3123 and its ^{13}C chemical shift

3131 C

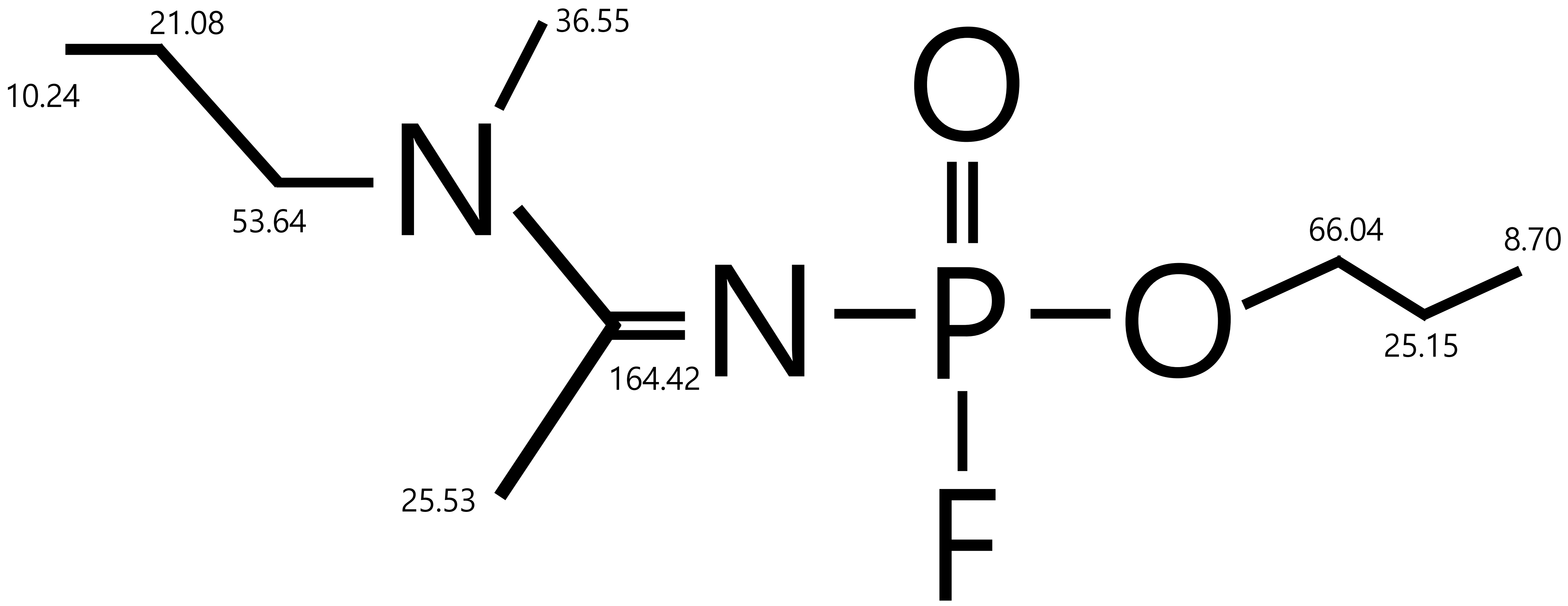

Figure S61. Structure 3131 and its ^{13}C chemical shift

3132 C

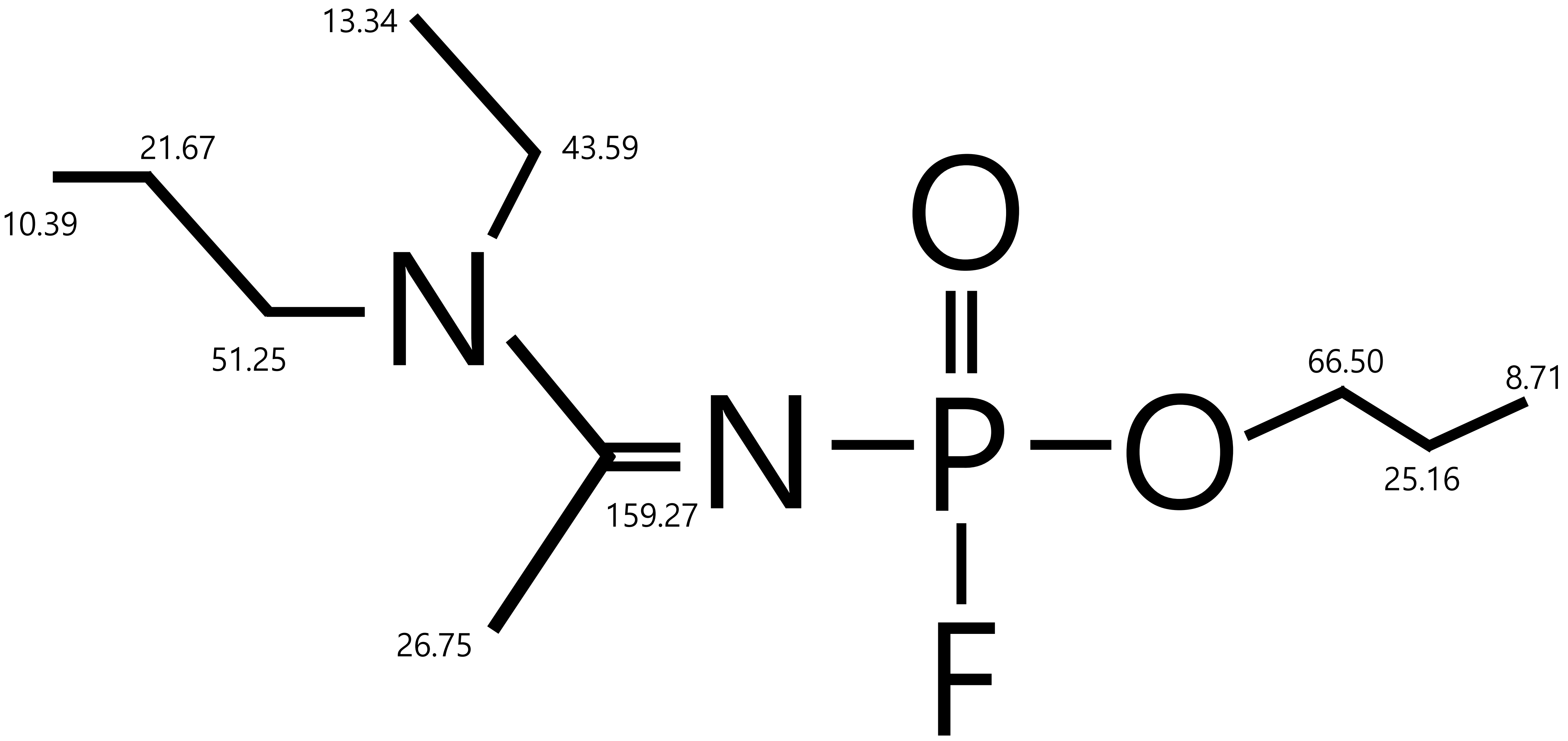

Figure S62. Structure 3132 and its ^{13}C chemical shift

3133 C

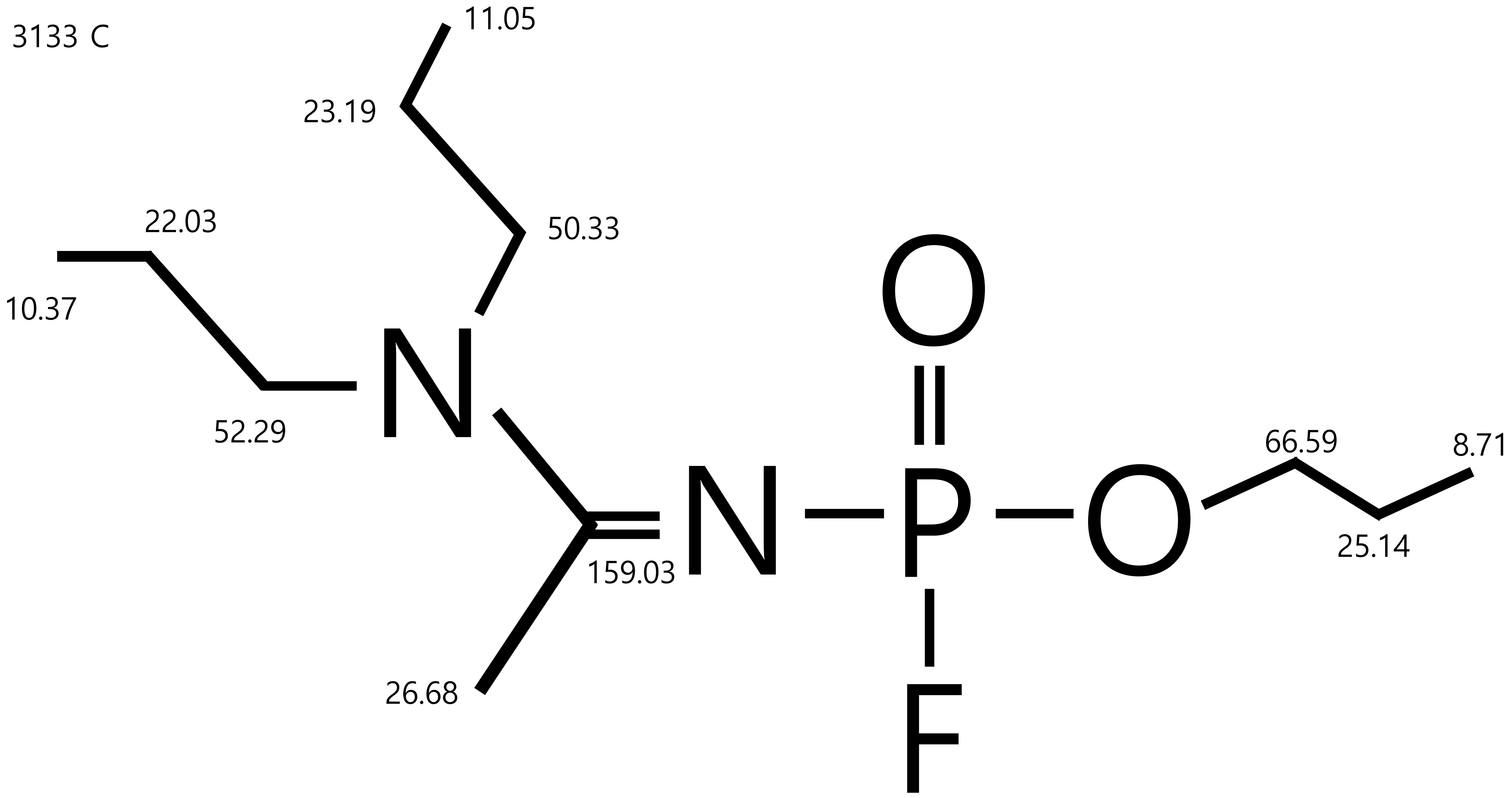

Figure S63. Structure 3133 and its ^{13}C chemical shift

3211 C

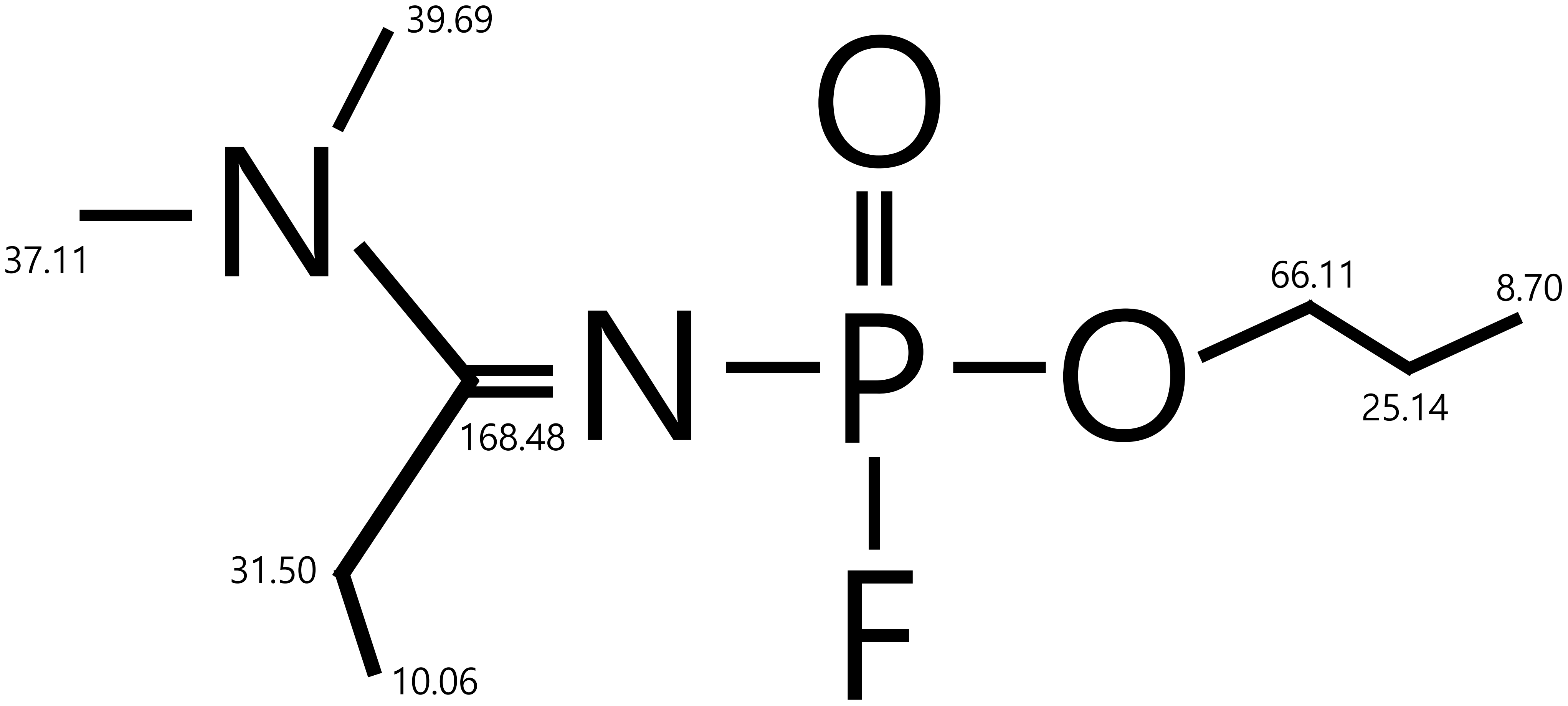

Figure S64. Structure 3211 and its ^{13}C chemical shift

3212 C

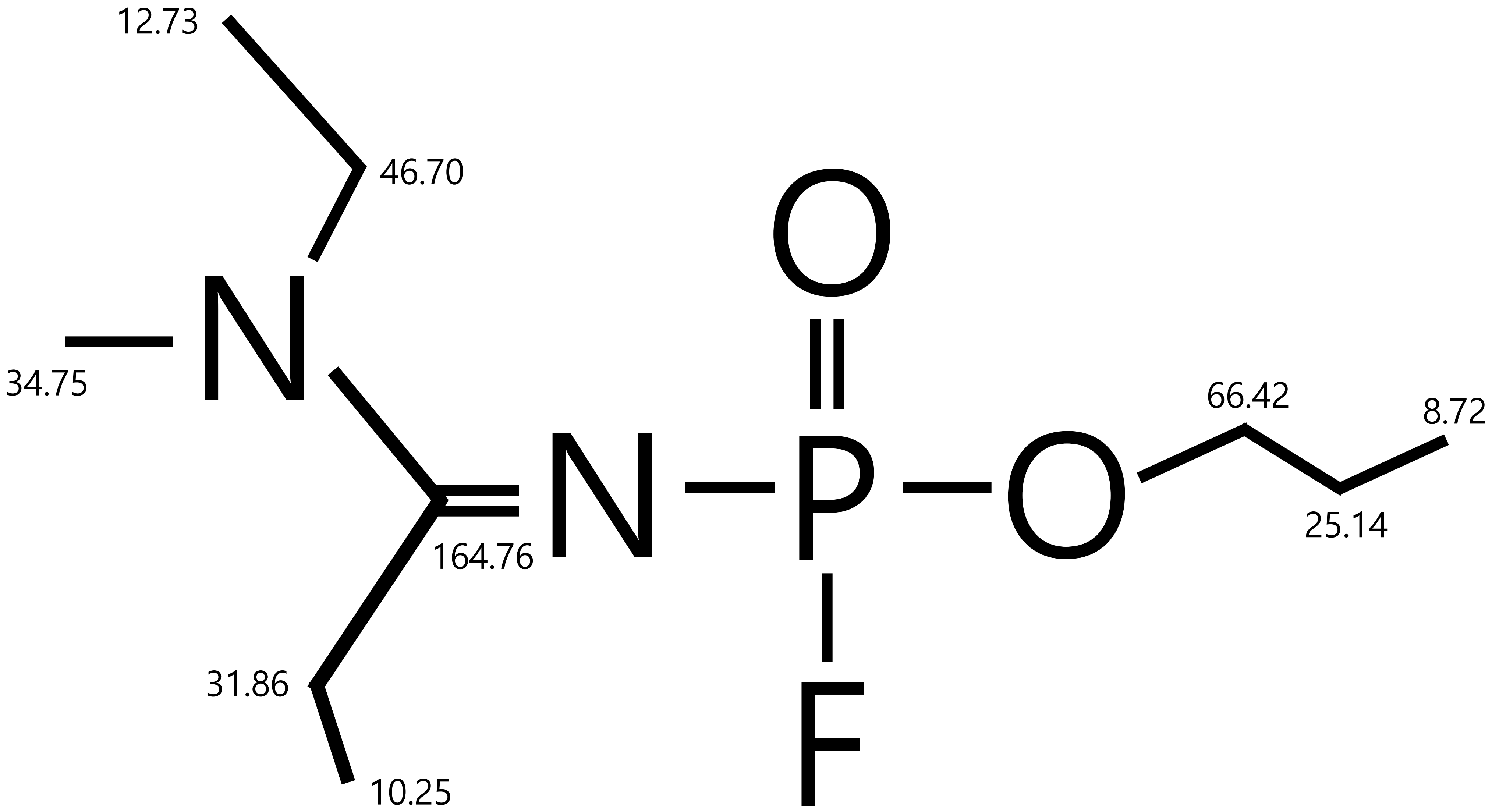

Figure S65. Structure 3212 and its ^{13}C chemical shift

3213 C

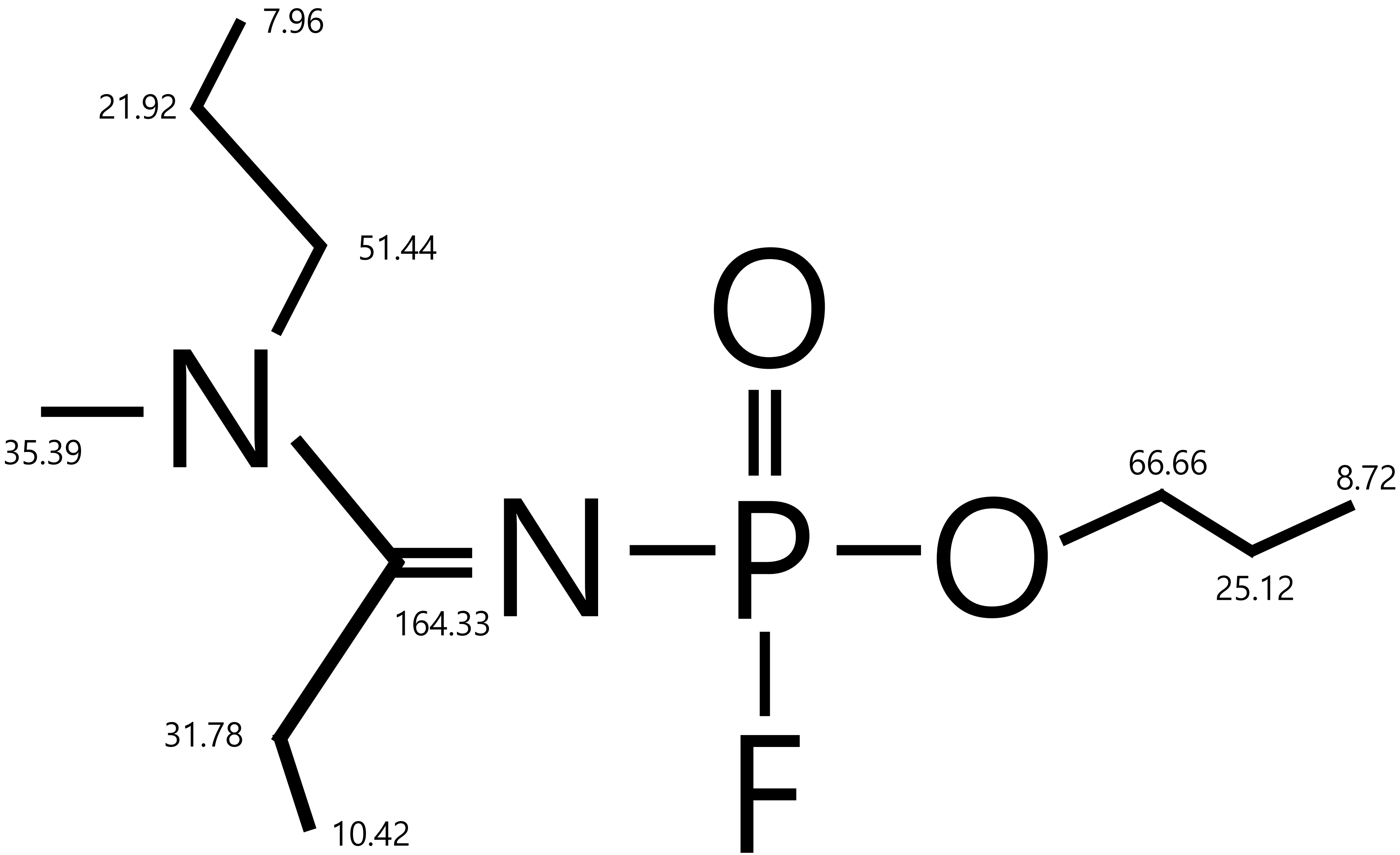

Figure S66. Structure 3213 and its ^{13}C chemical shift

3221 C

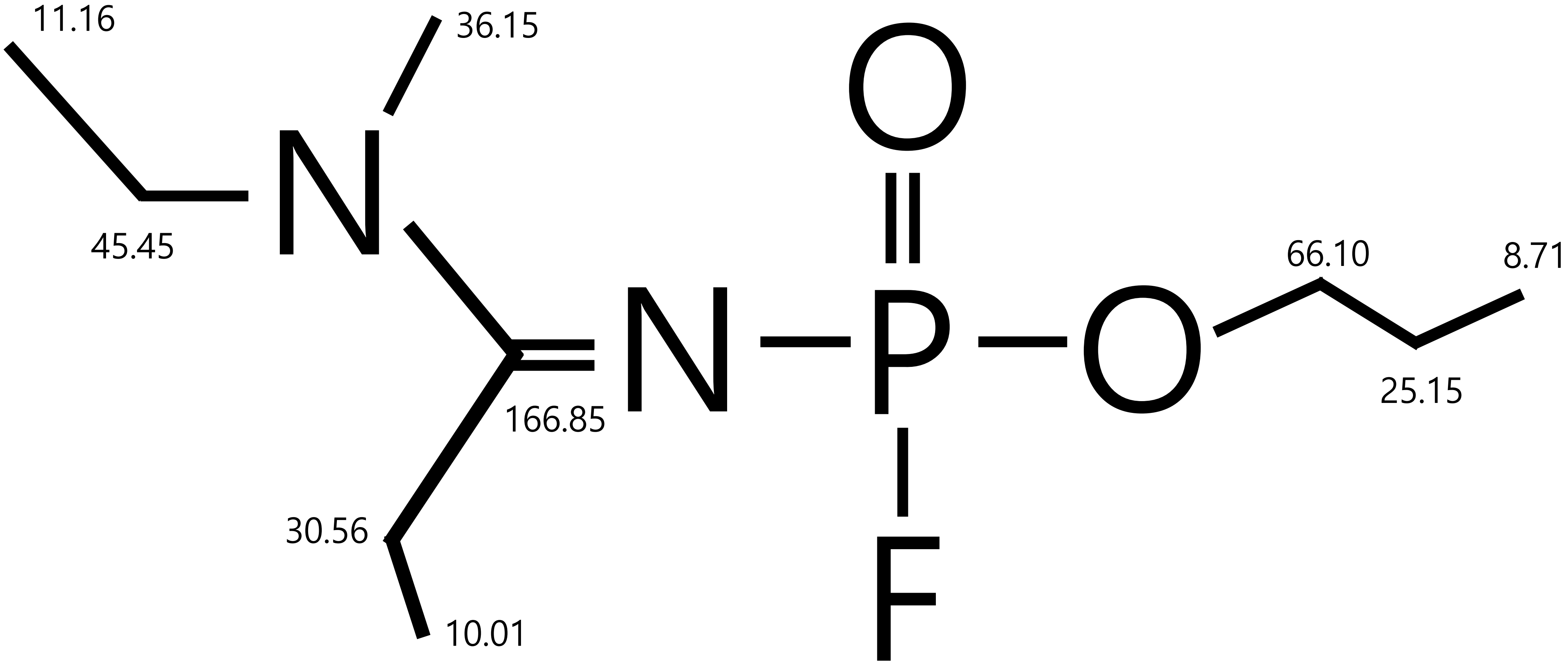

Figure S67. Structure 3221 and its ^{13}C chemical shift

3222 C

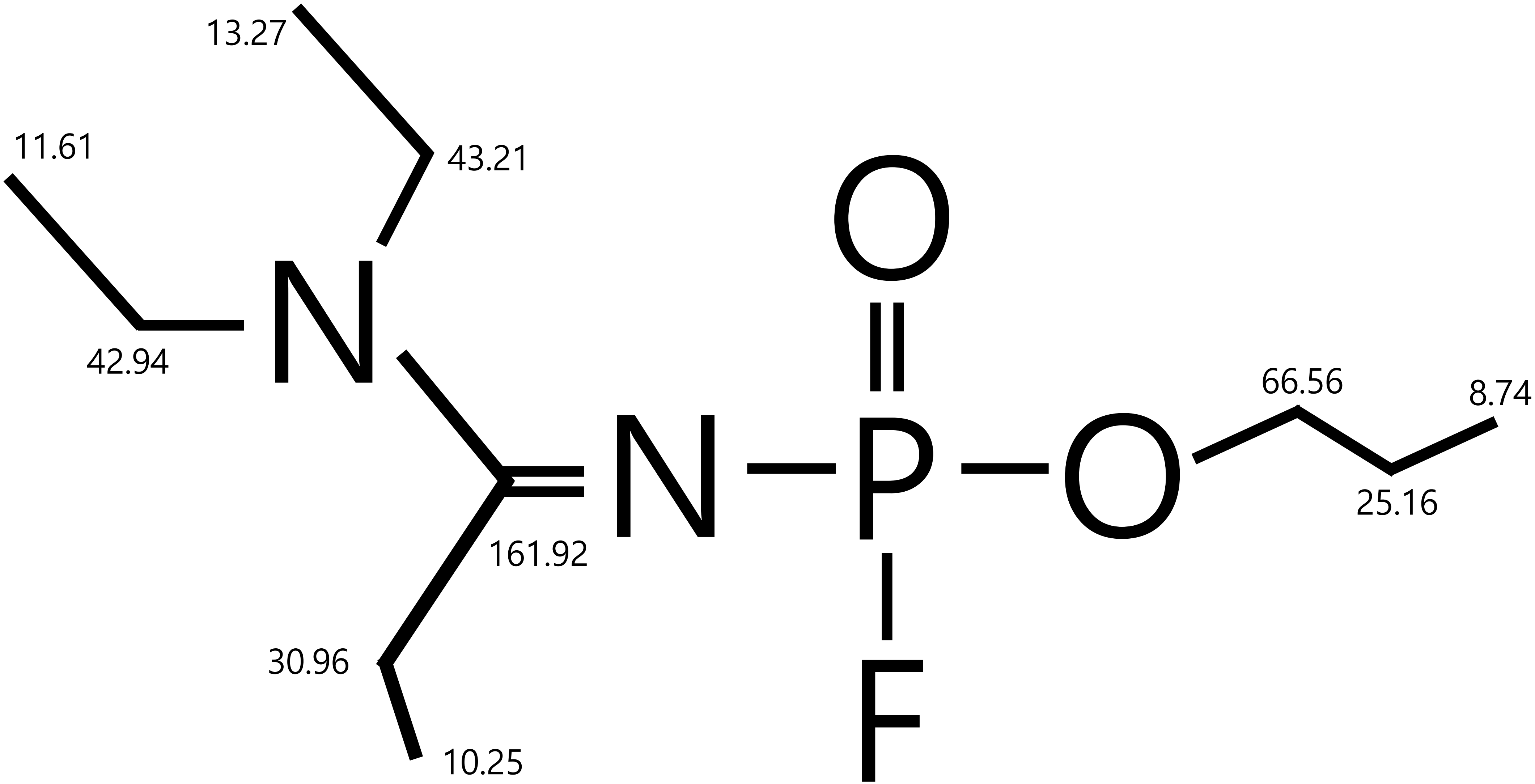

Figure S68. Structure 3222 and its ^{13}C chemical shift

3223 C

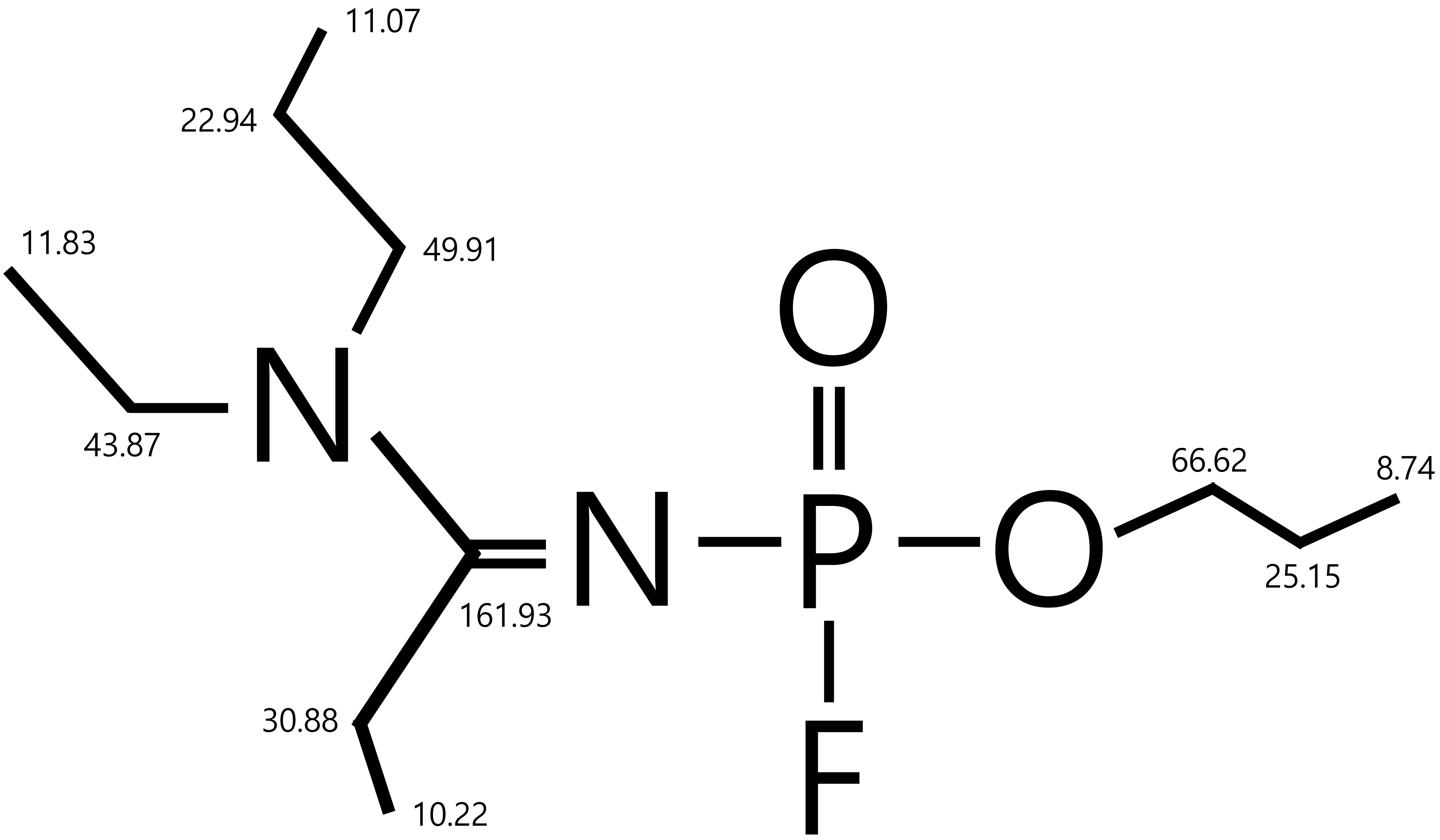

Figure S69. Structure 3223 and its ^{13}C chemical shift

3231 C

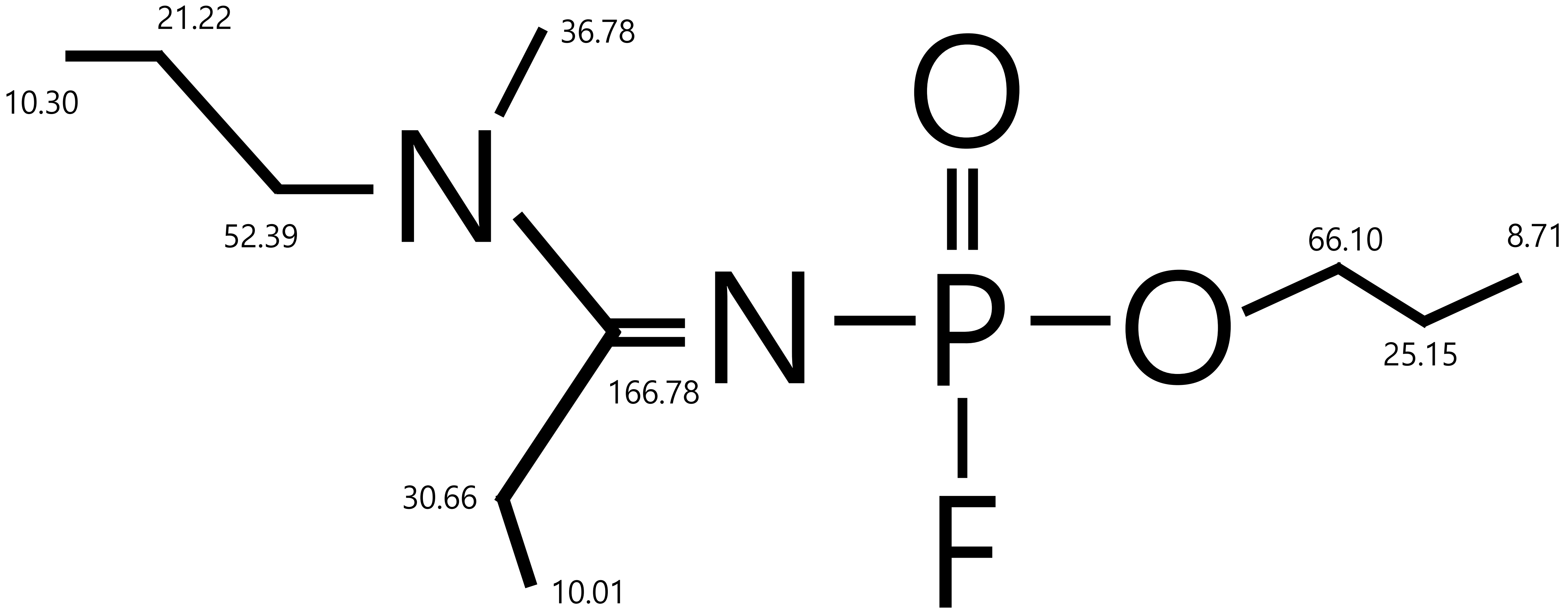

Figure S70. Structure 3231 and its ^{13}C chemical shift

3232 C

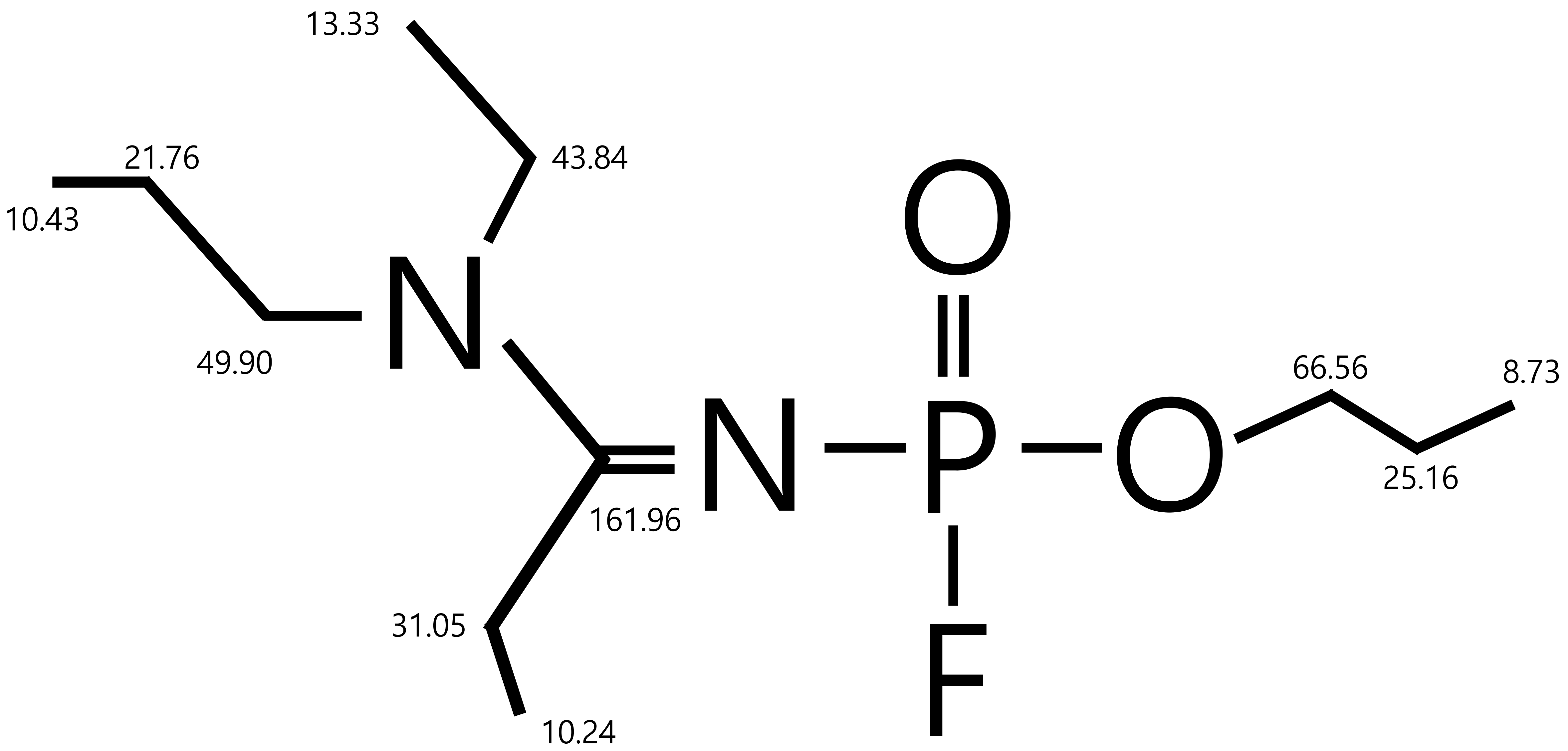

Figure S71. Structure 3232 and its ^{13}C chemical shift

3233 C

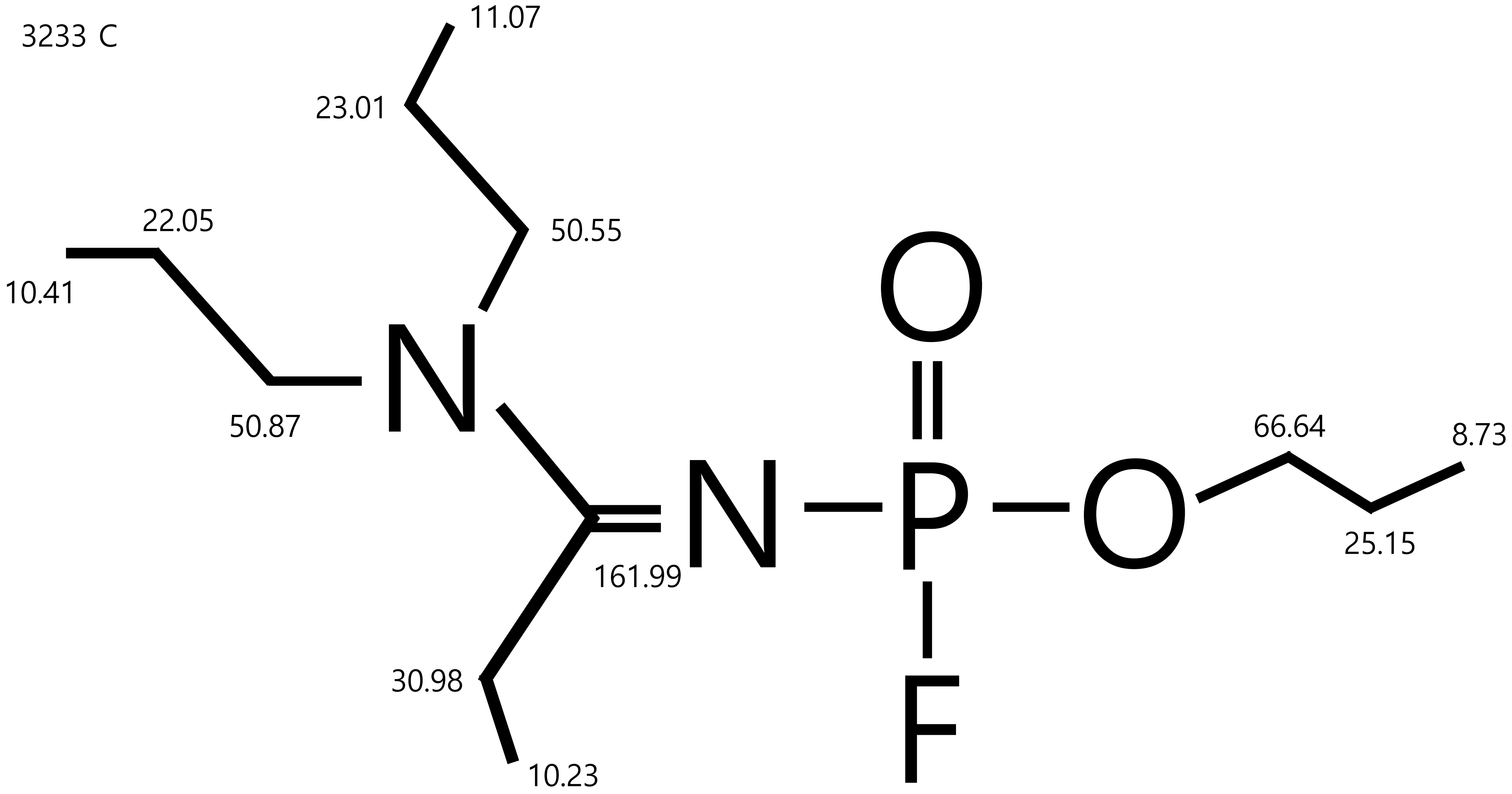

Figure S72. Structure 3233 and its ^{13}C chemical shift

3311 C

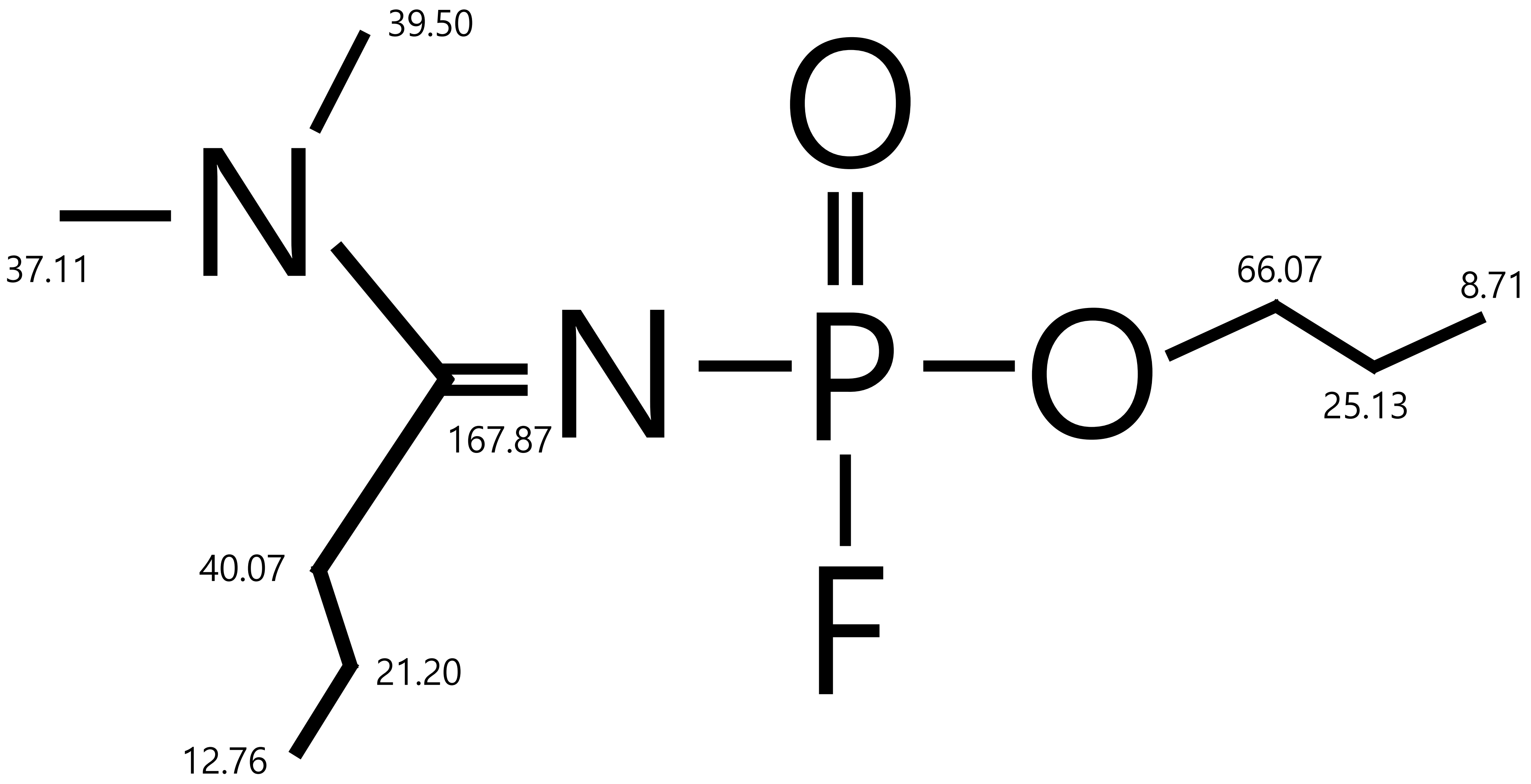

Figure S73. Structure 3311 and its ^{13}C chemical shift

3312 C

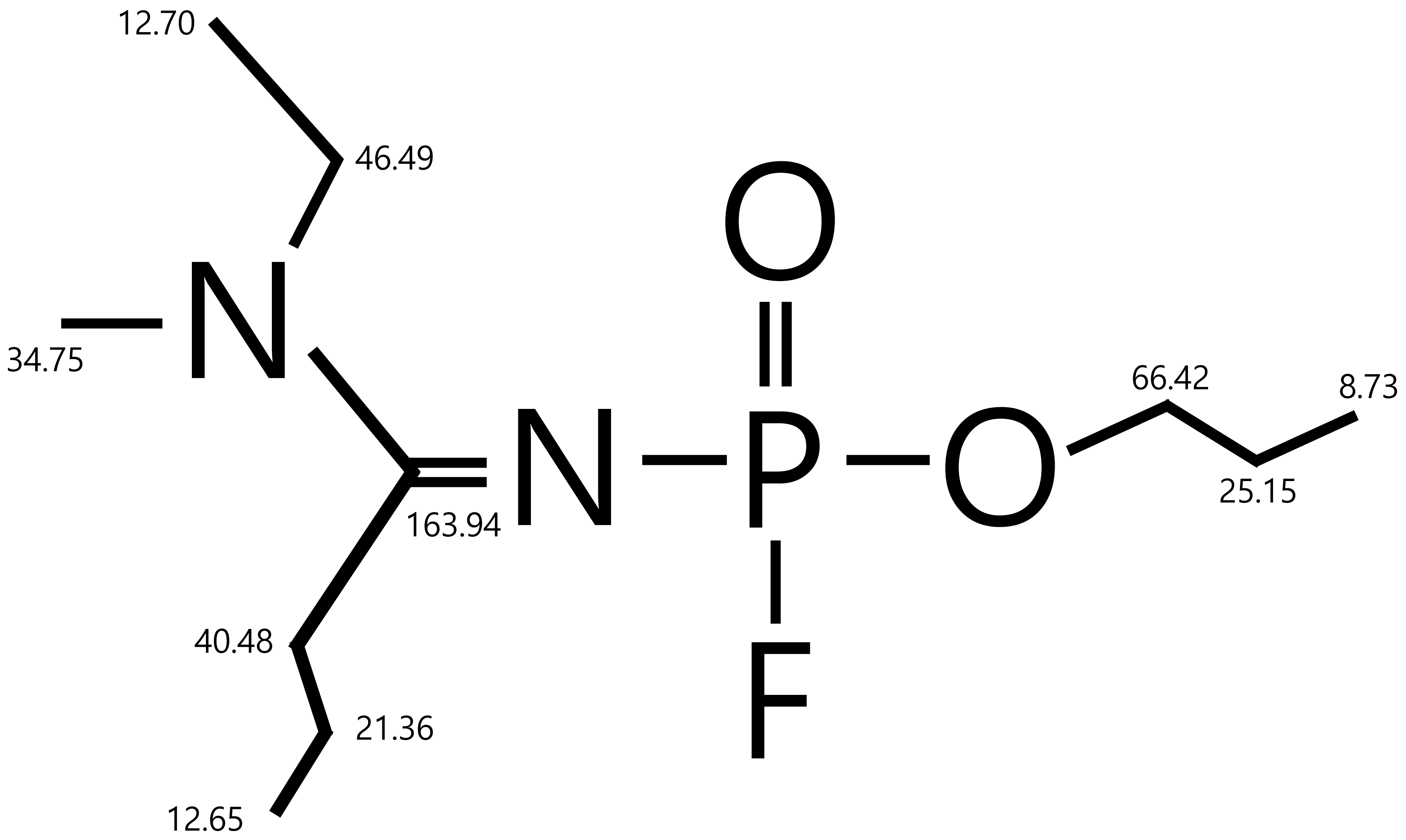

Figure S74. Structure 3312 and its ^{13}C chemical shift

3313 C

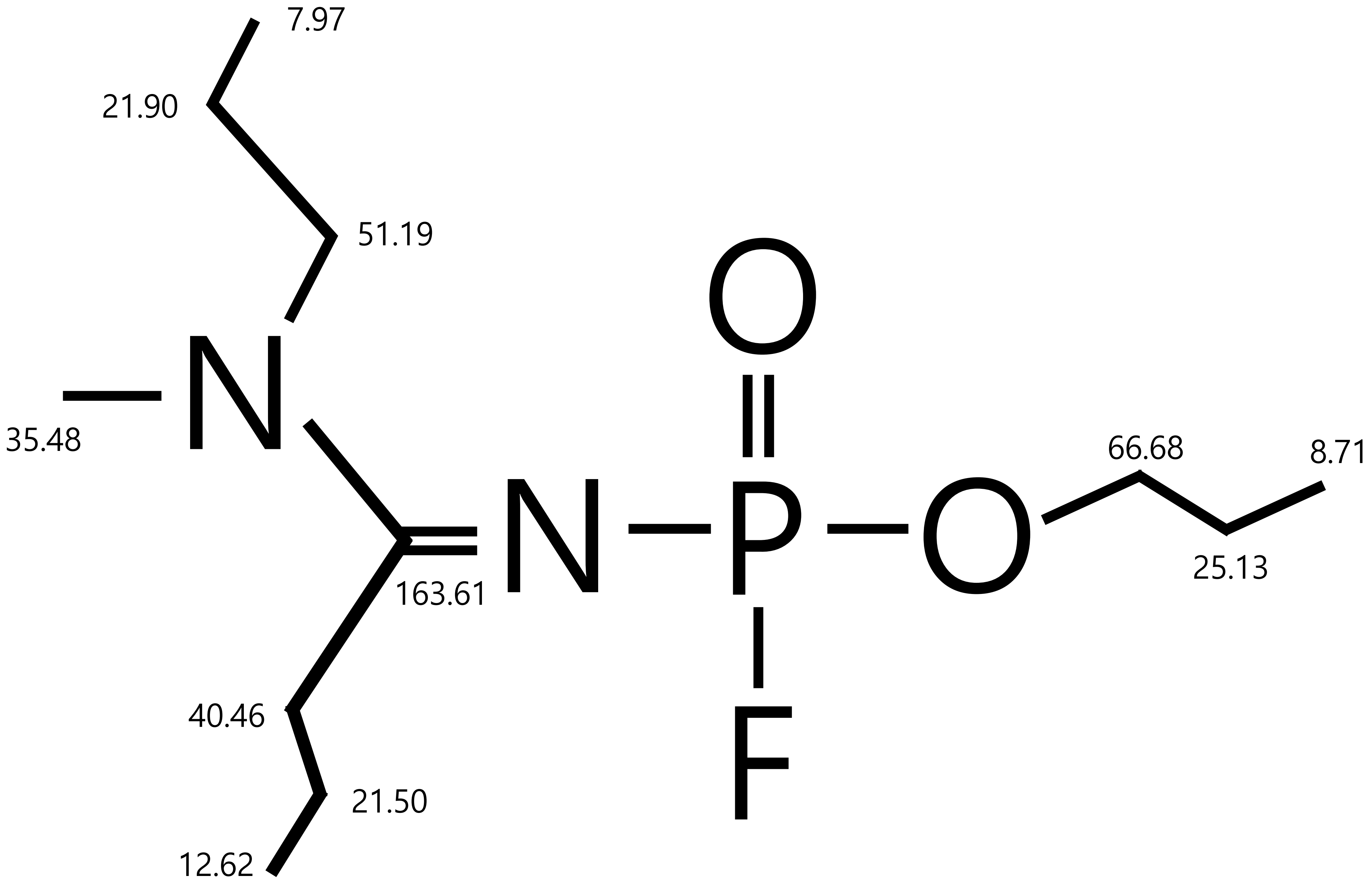

Figure S75. Structure 3313 and its ^{13}C chemical shift

3321 C

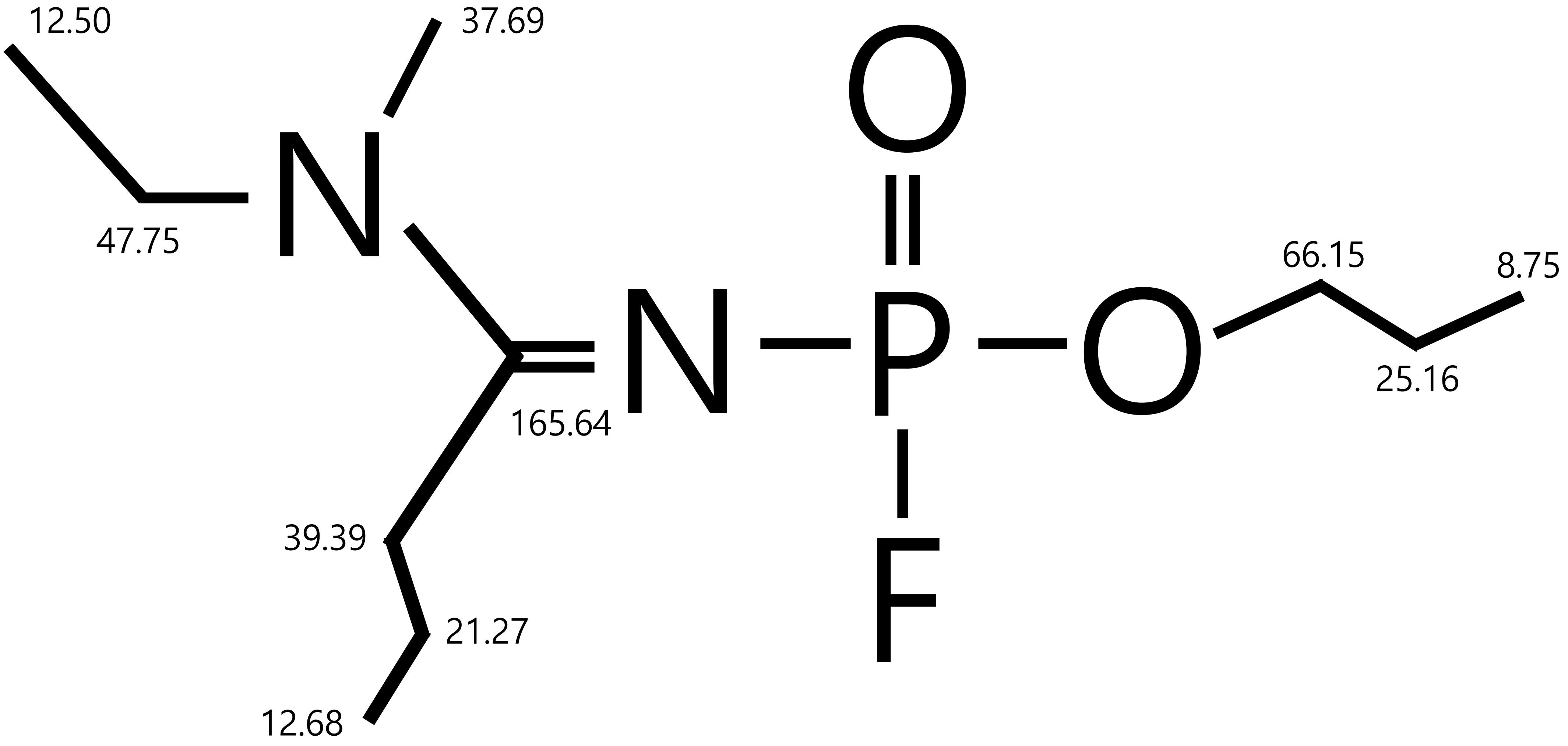

Figure S76. Structure 3321 and its ^{13}C chemical shift

3322 C

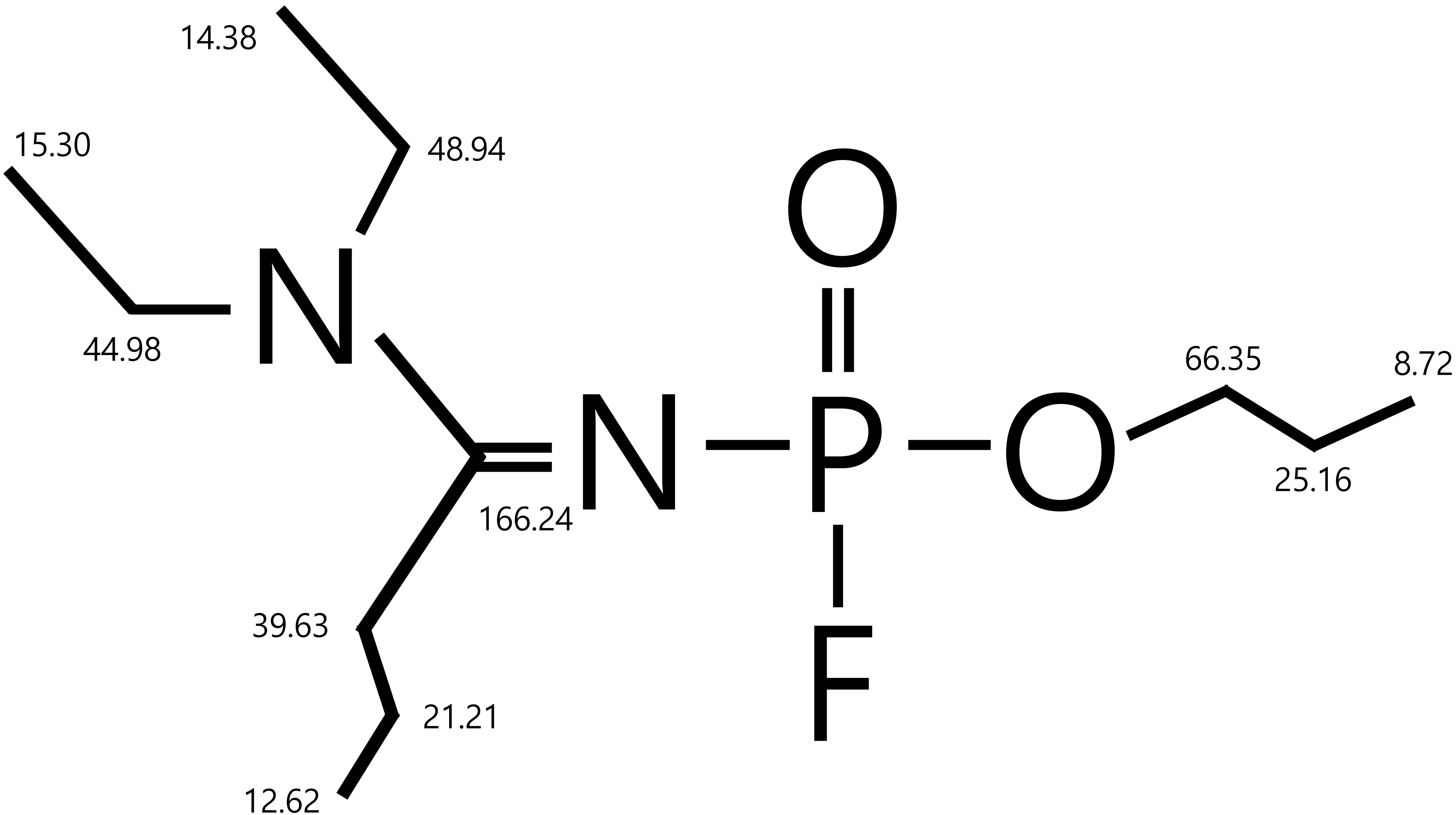

Figure S77. Structure 3322 and its ^{13}C chemical shift

3323 C

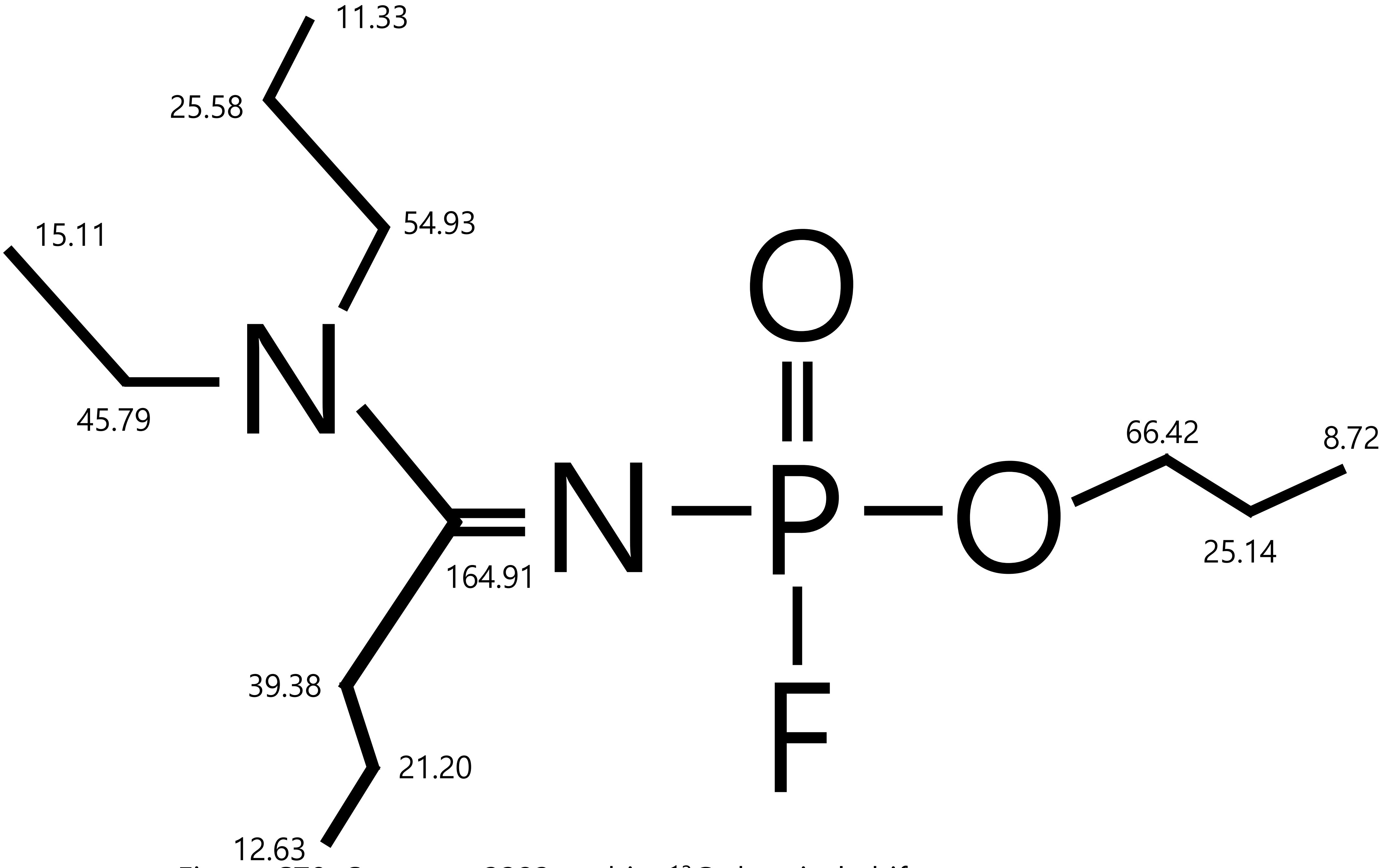

Figure S78. Structure 3323 and its ^{13}C chemical shift

3331 C

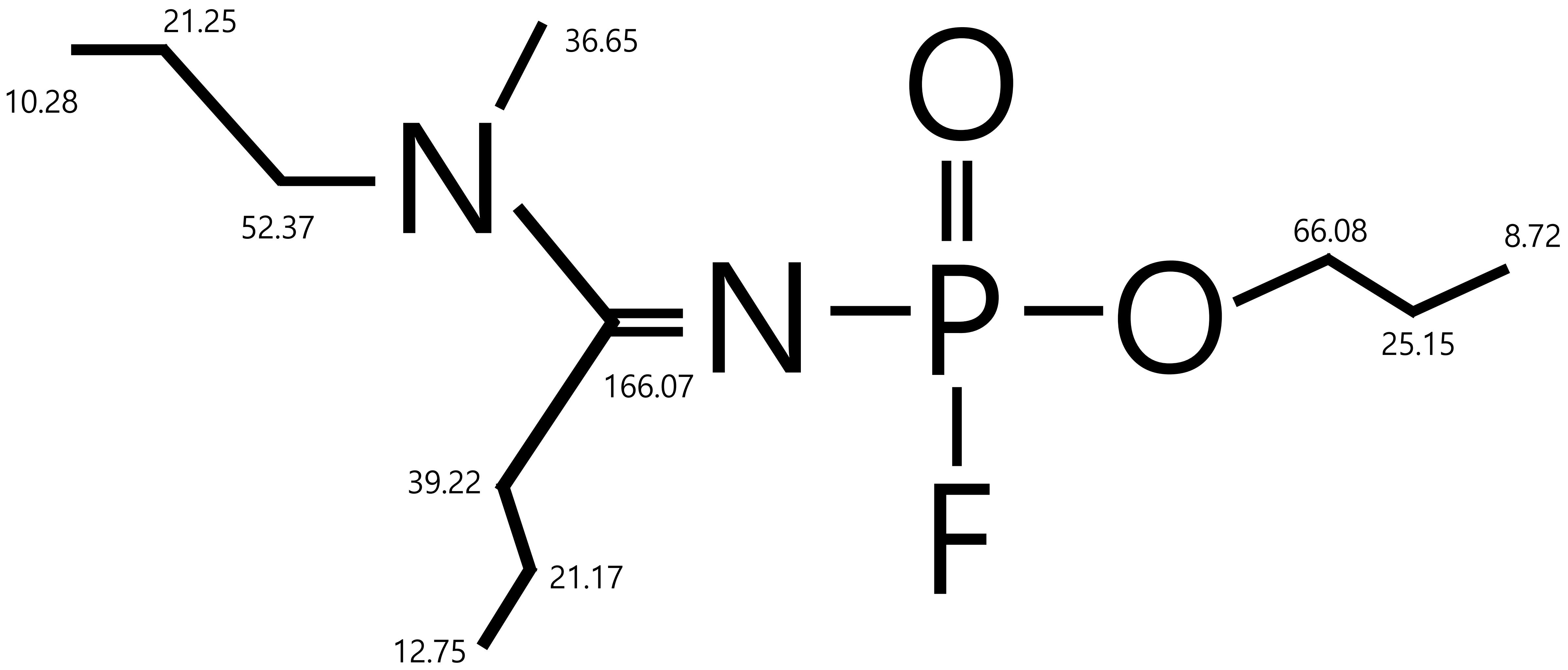

Figure S79. Structure 3331 and its ^{13}C chemical shift

3332 C

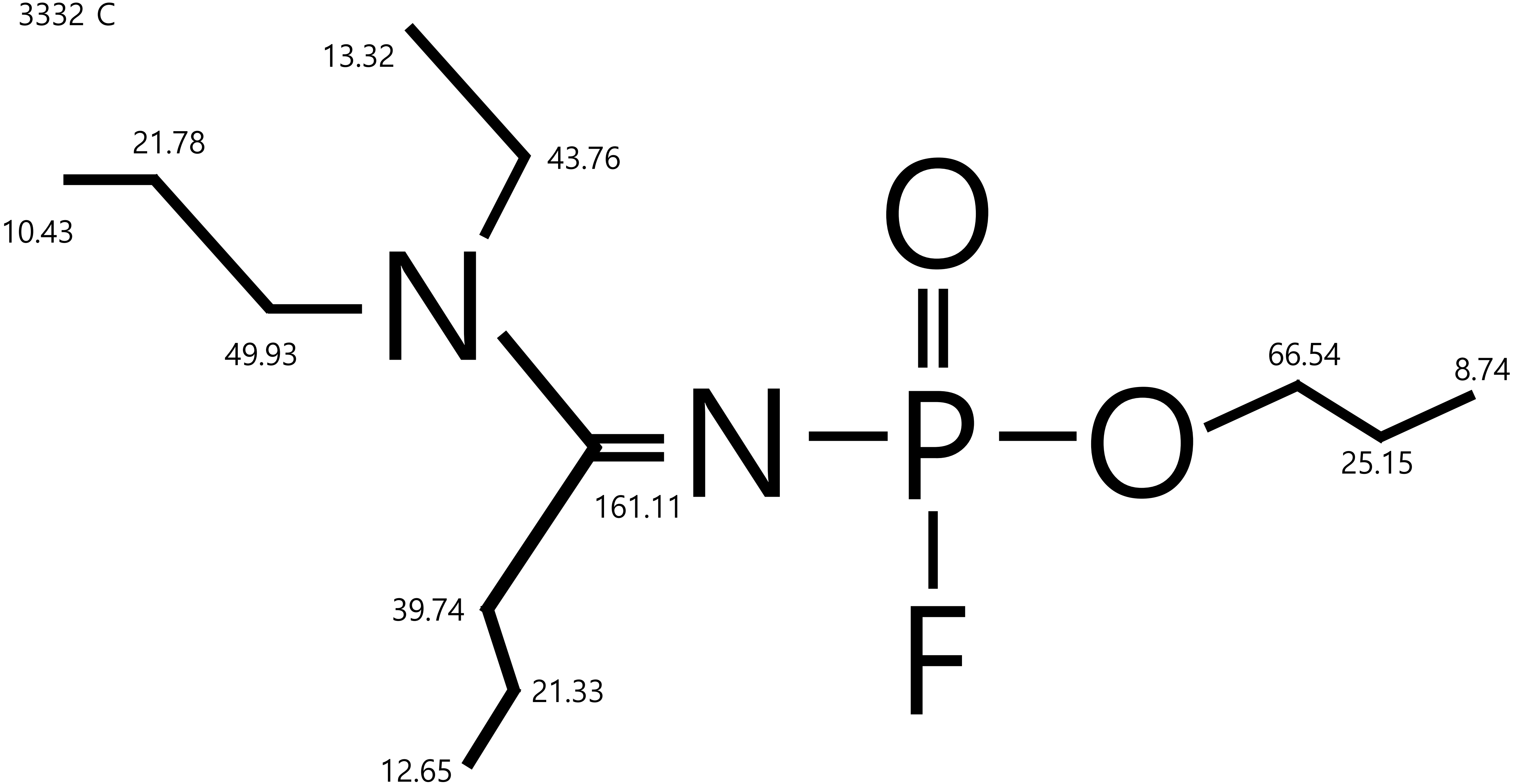

Figure S80. Structure 3332 and its ^{13}C chemical shift

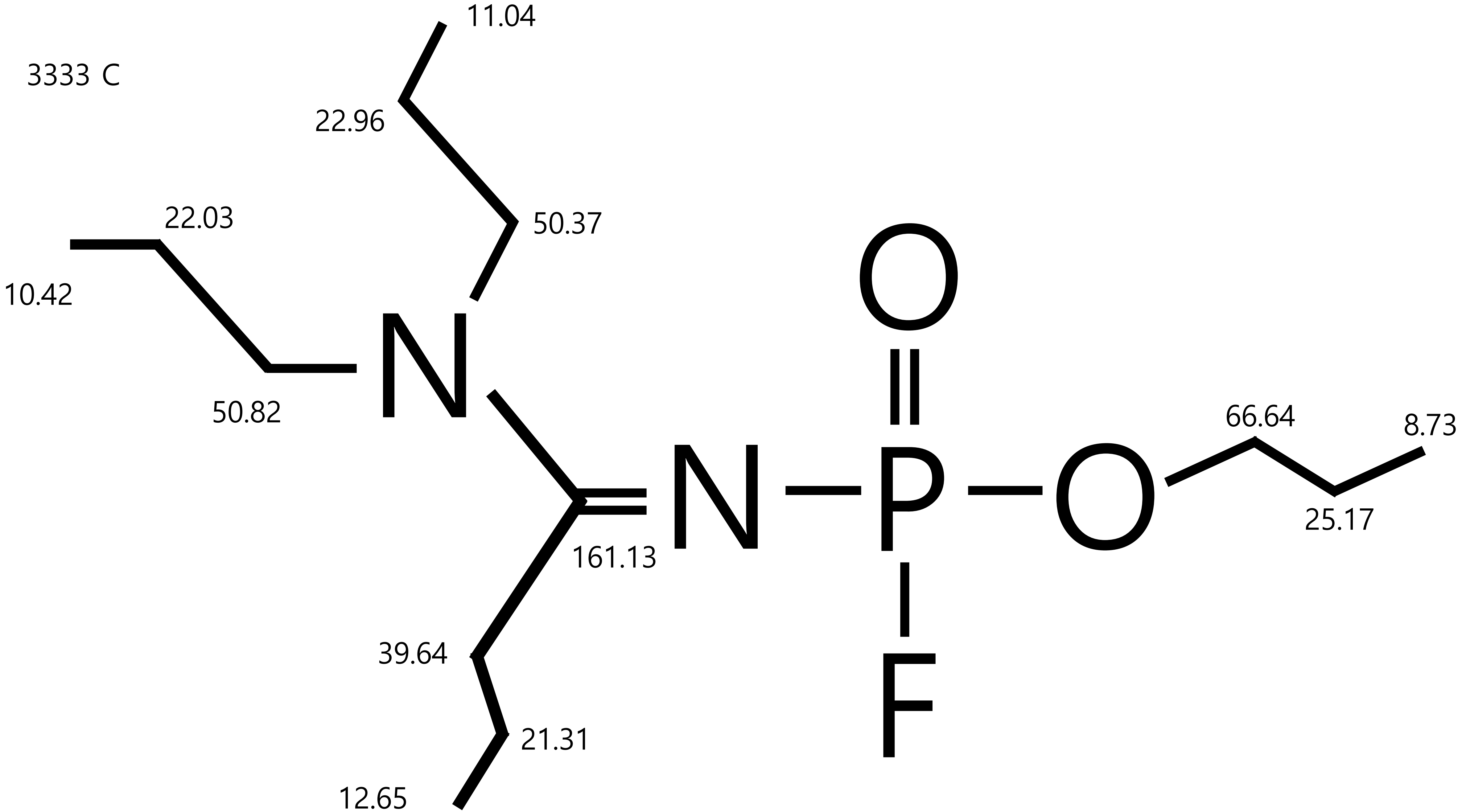

Figure S81. Structure 3333 and its ^{13}C chemical shift

A242 C

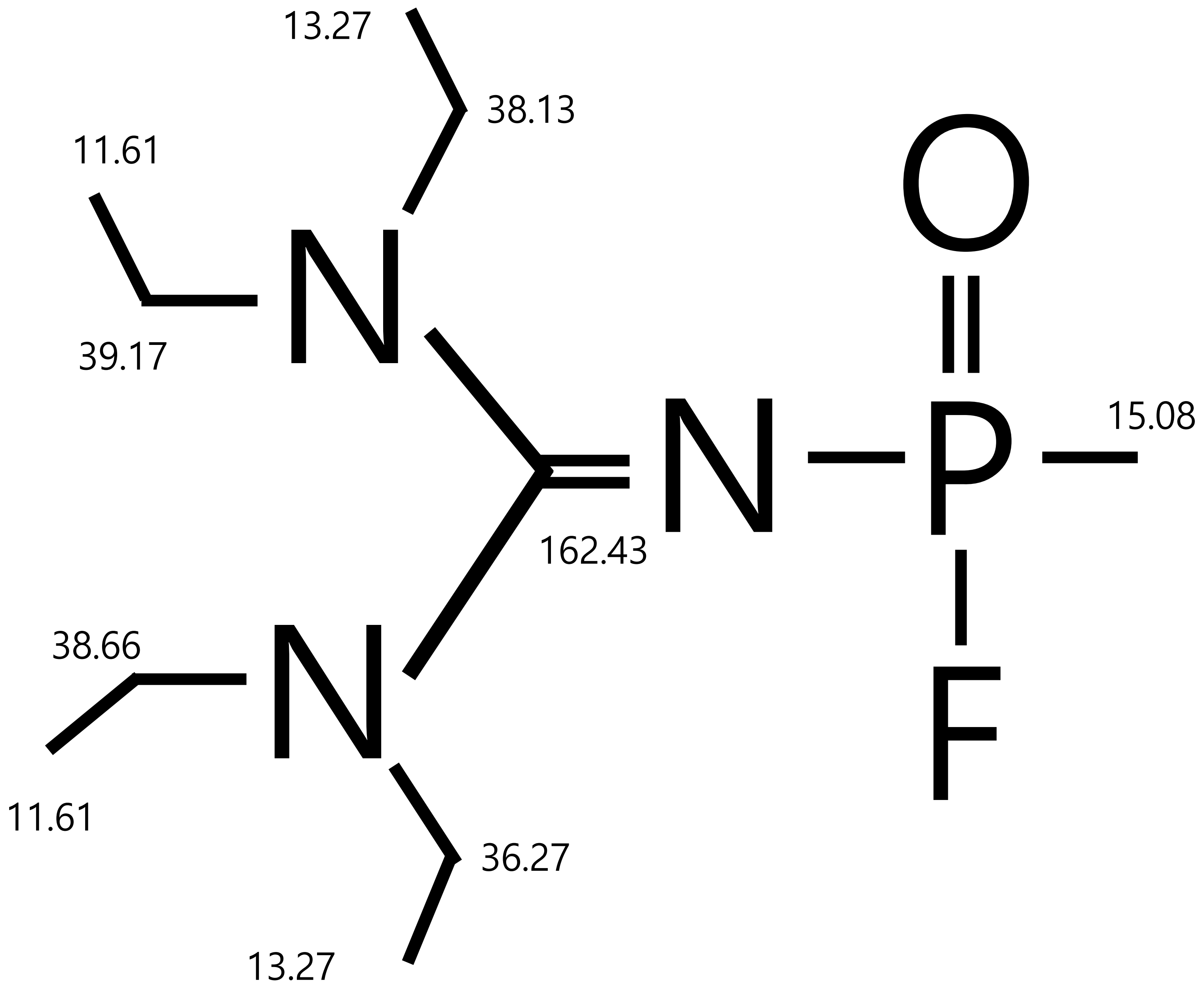

Figure S82. Structure A242 and its ^{13}C chemical shift

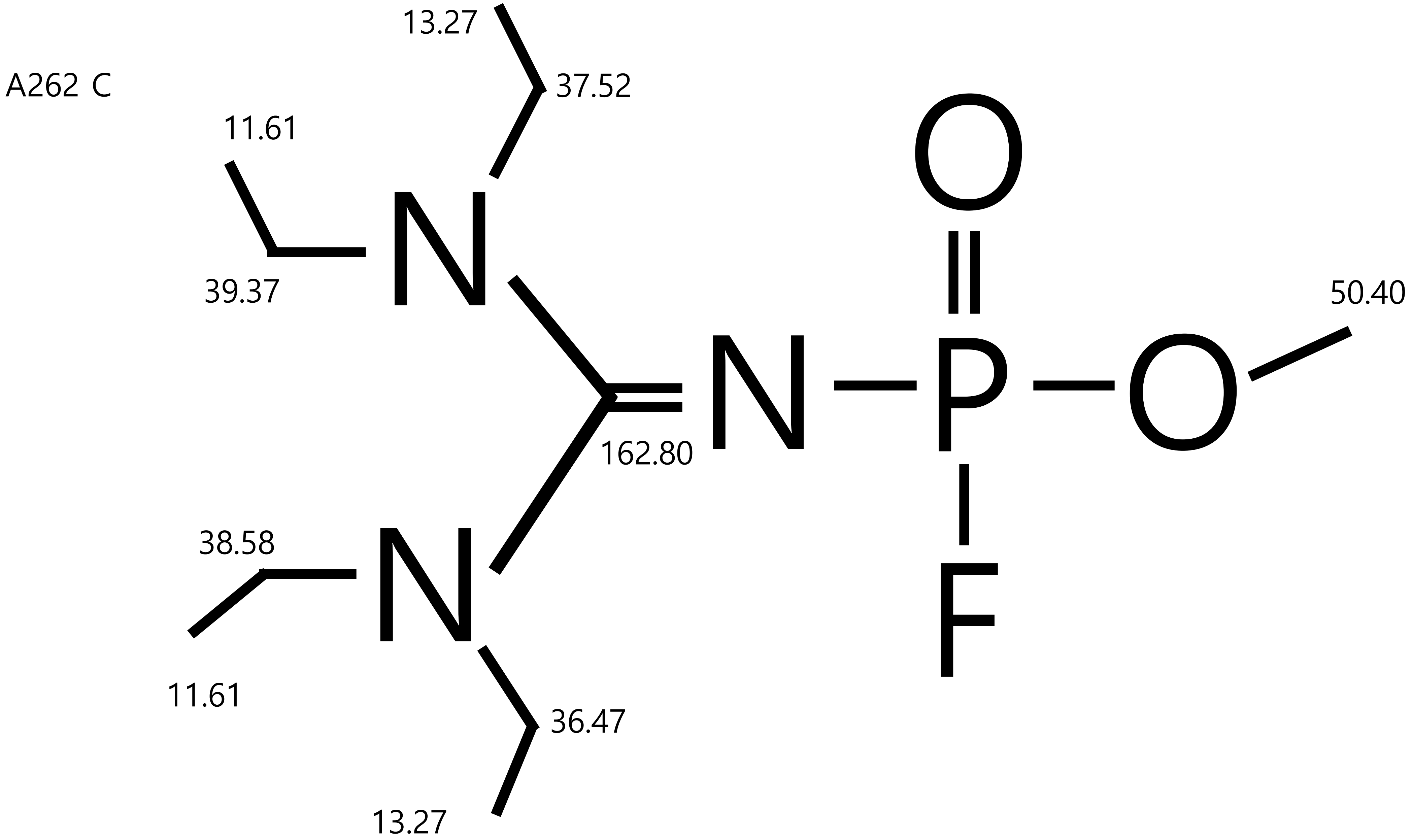

Figure S83. Structure A262 and its ^{13}C chemical shift

1111 H

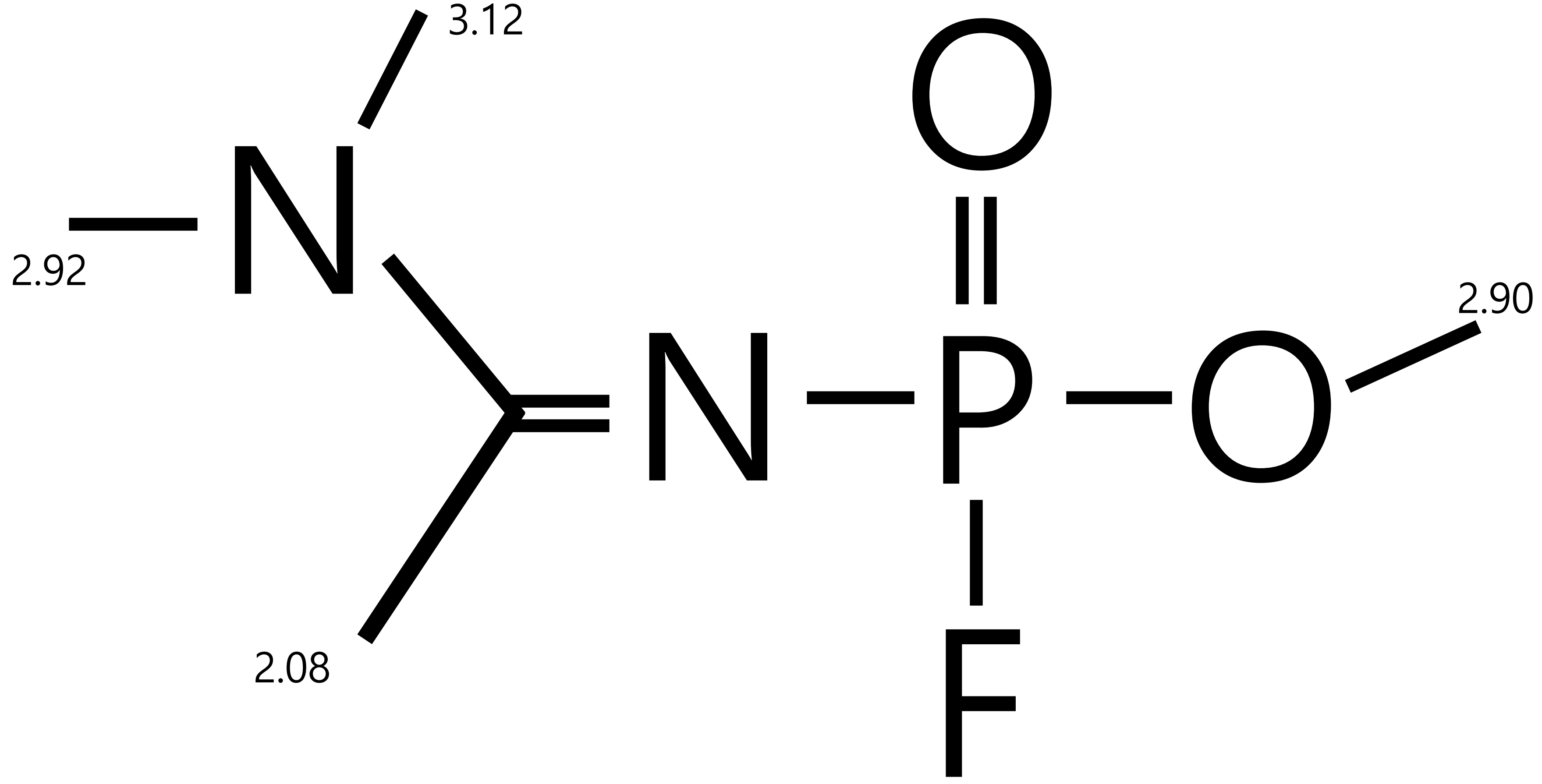

Figure S84. Structure 1111 and its ^{1}H chemical shift

1112 H

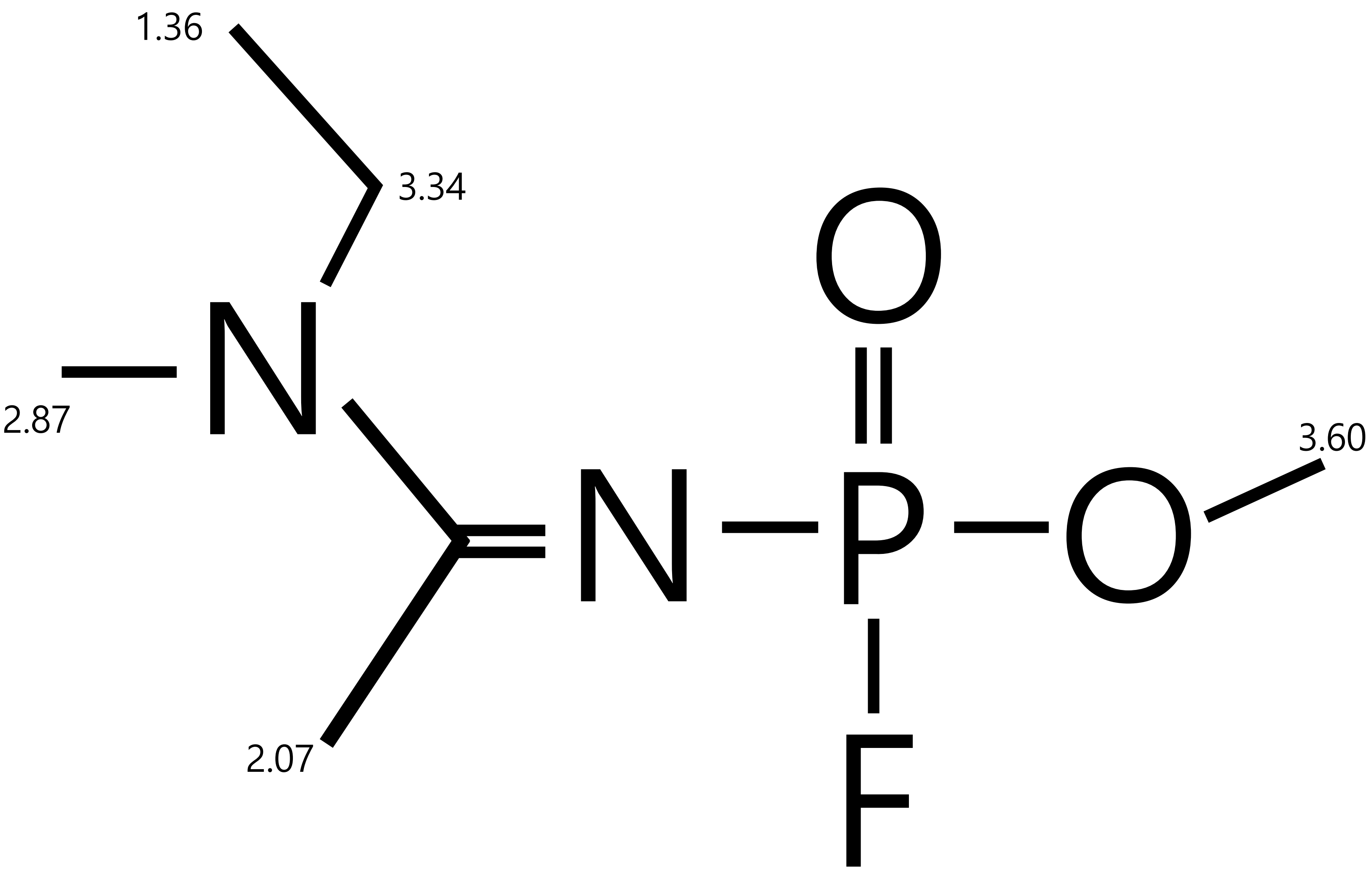

Figure S85. Structure 1112 and its ^{1}H chemical shift

1113 H

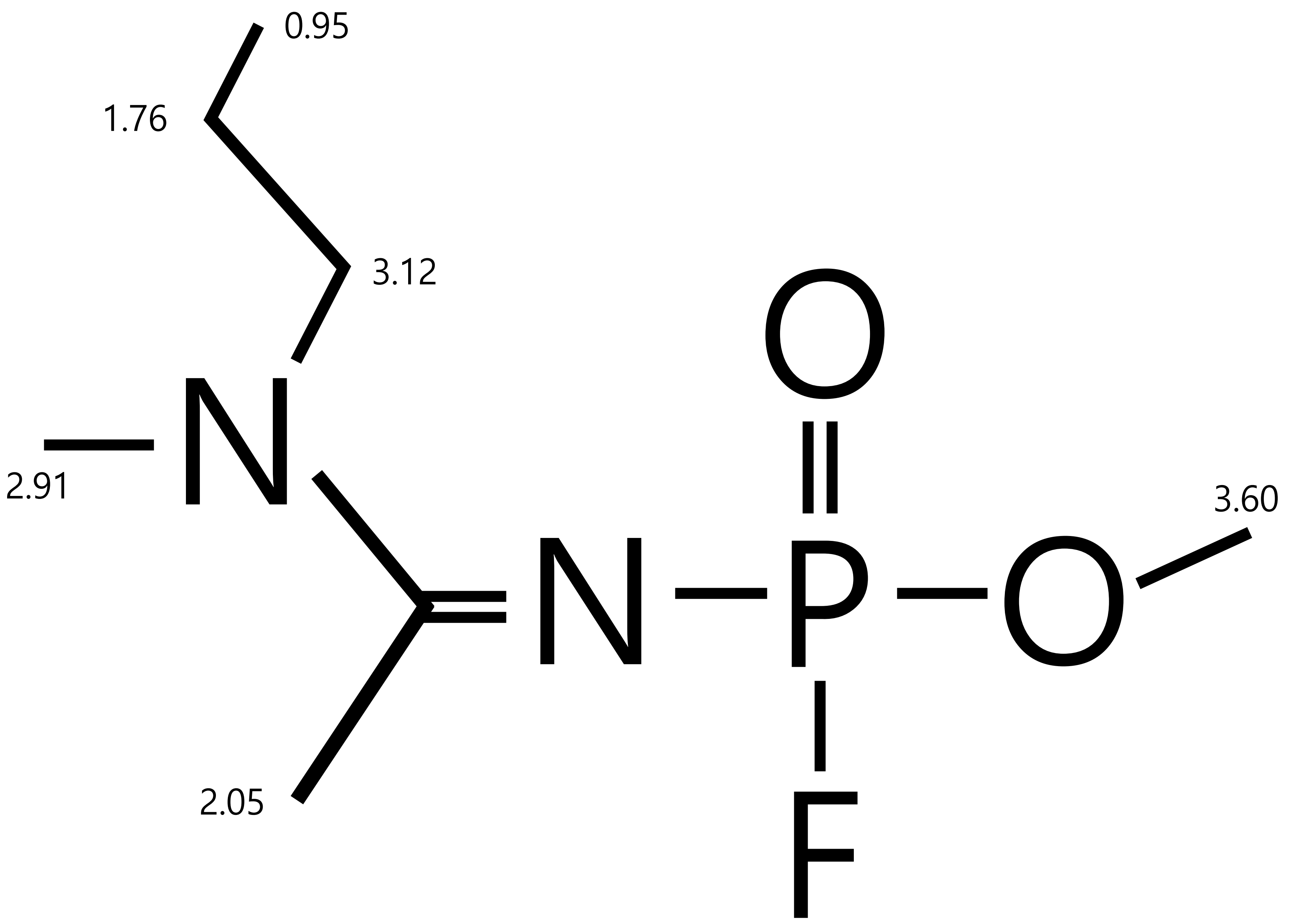

Figure S86. Structure 1113 and its ^{1}H chemical shift

1121 H

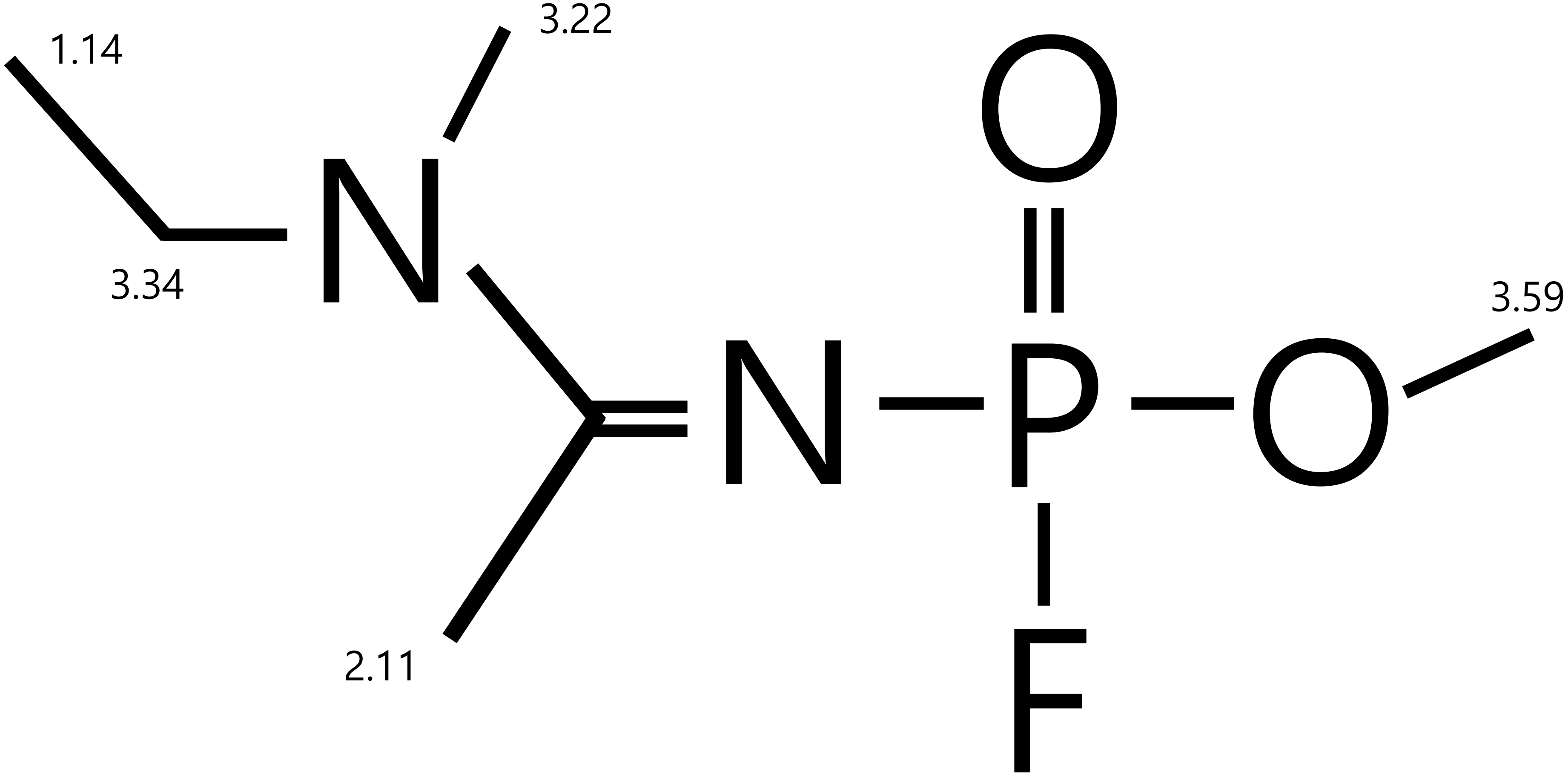

Figure S87. Structure 1121 and its ^{1}H chemical shift

1122 H

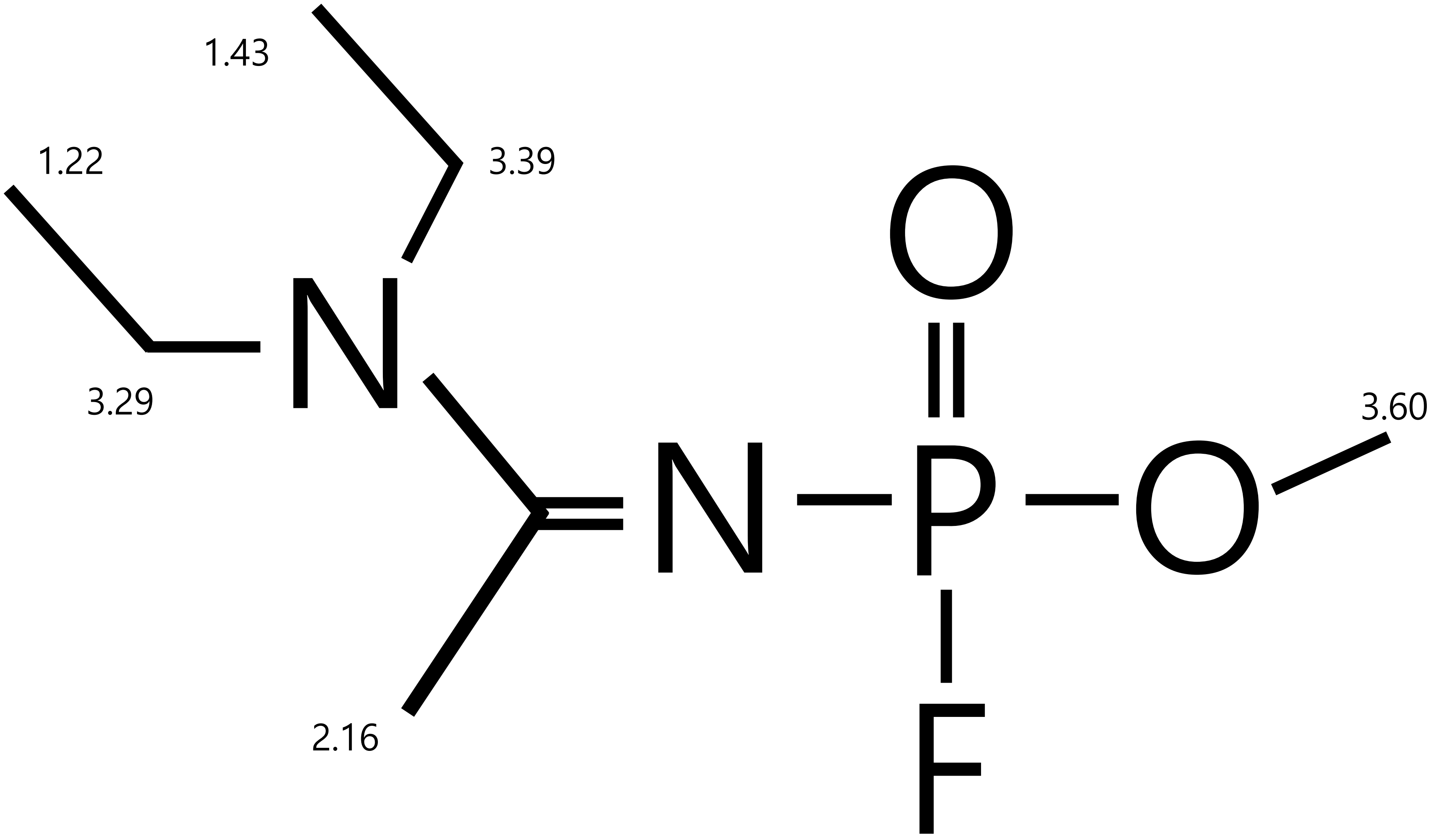

Figure S88. Structure 1122 and its ¹H chemical shift

1123 H

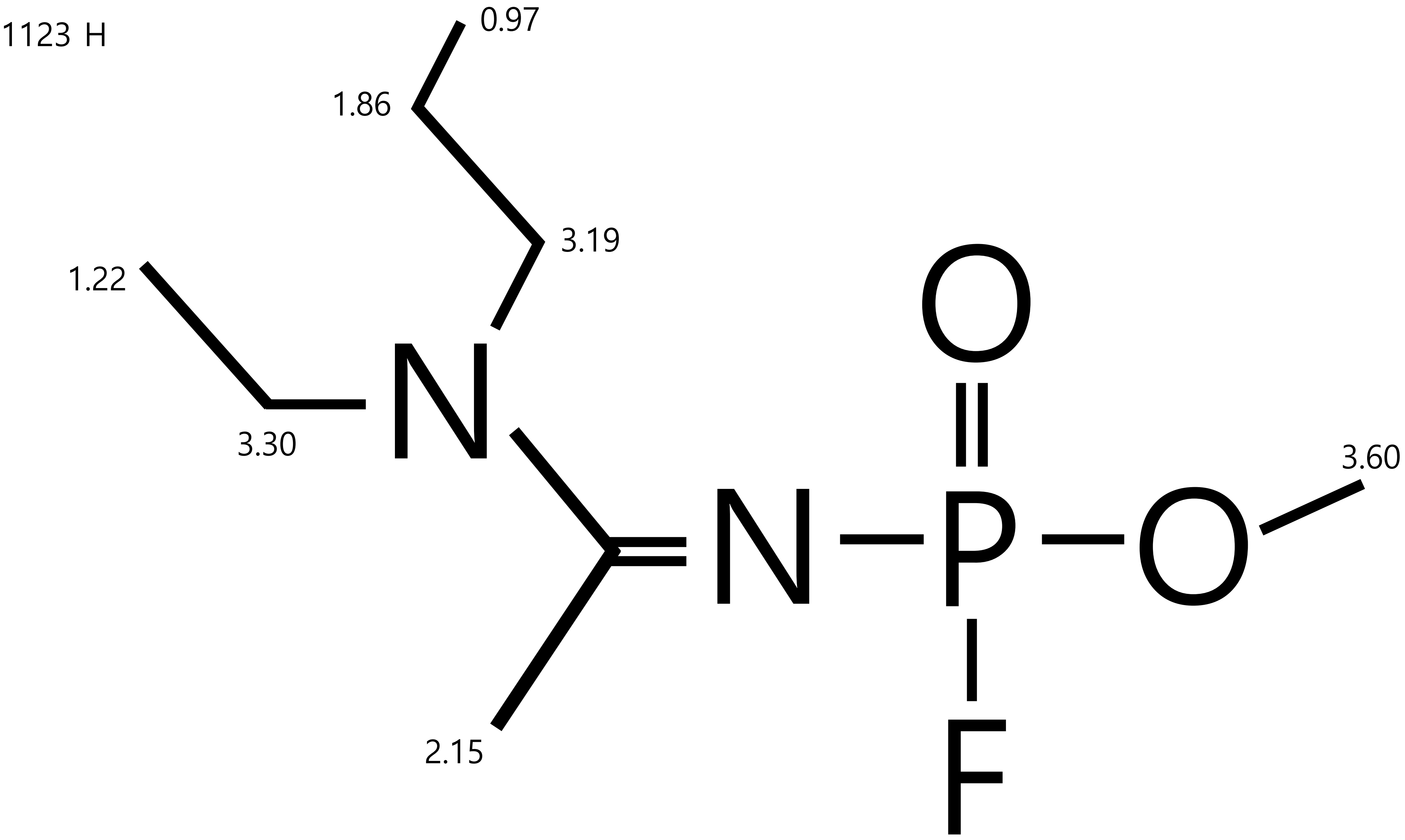

Figure S89. Structure 1123 and its ^{1}H chemical shift

1131 H

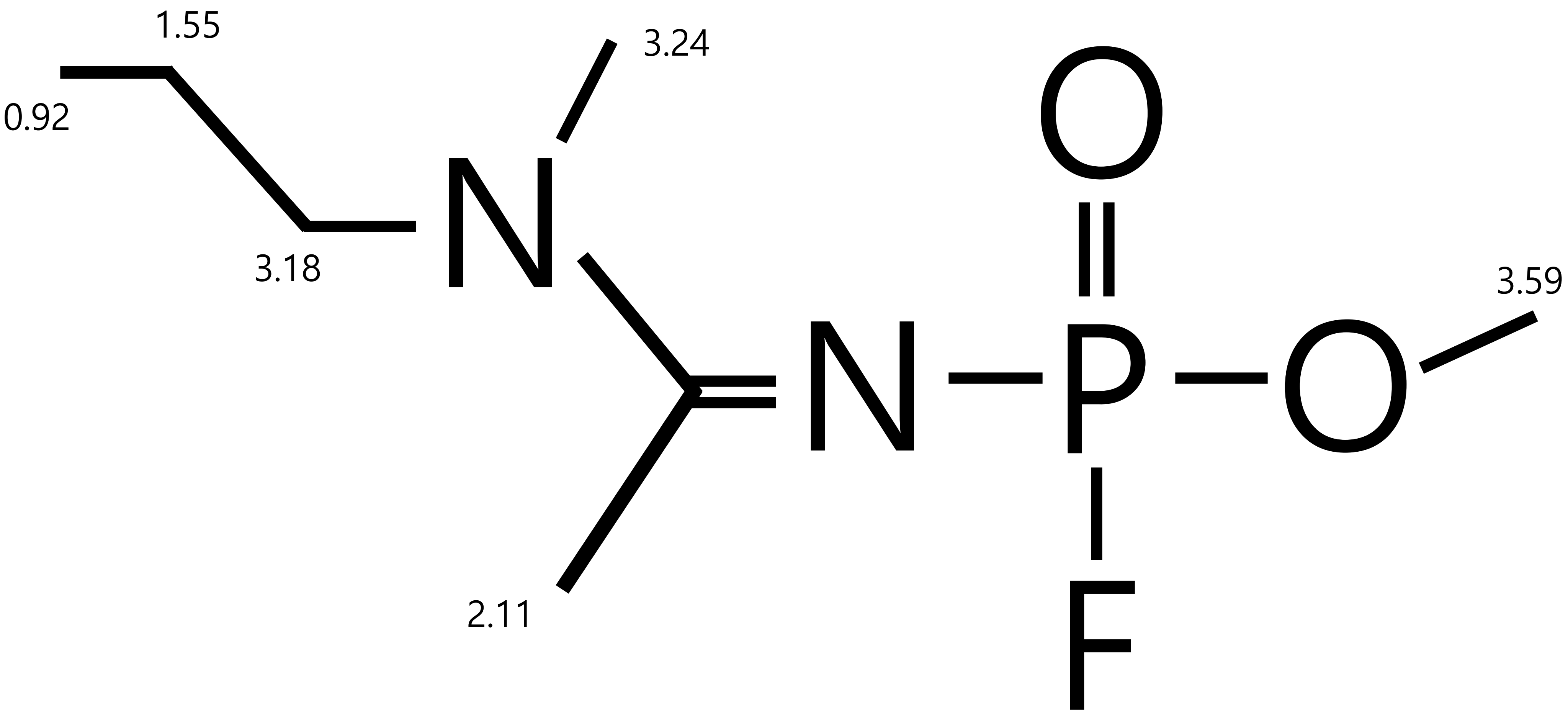

Figure S90. Structure 1131 and its ^{1}H chemical shift

1132 H

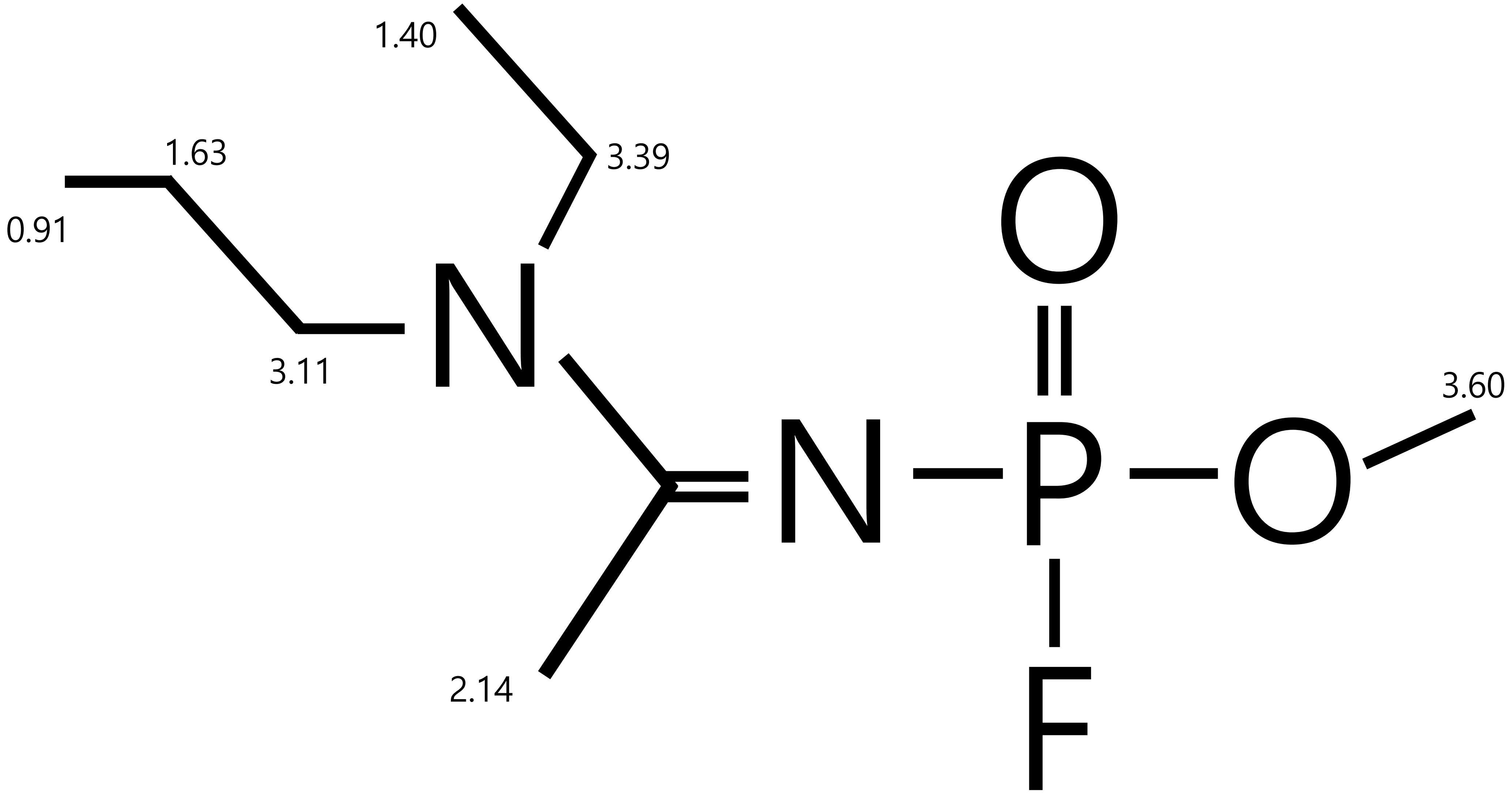

Figure S91. Structure 1132 and its ^{1}H chemical shift

1133 H

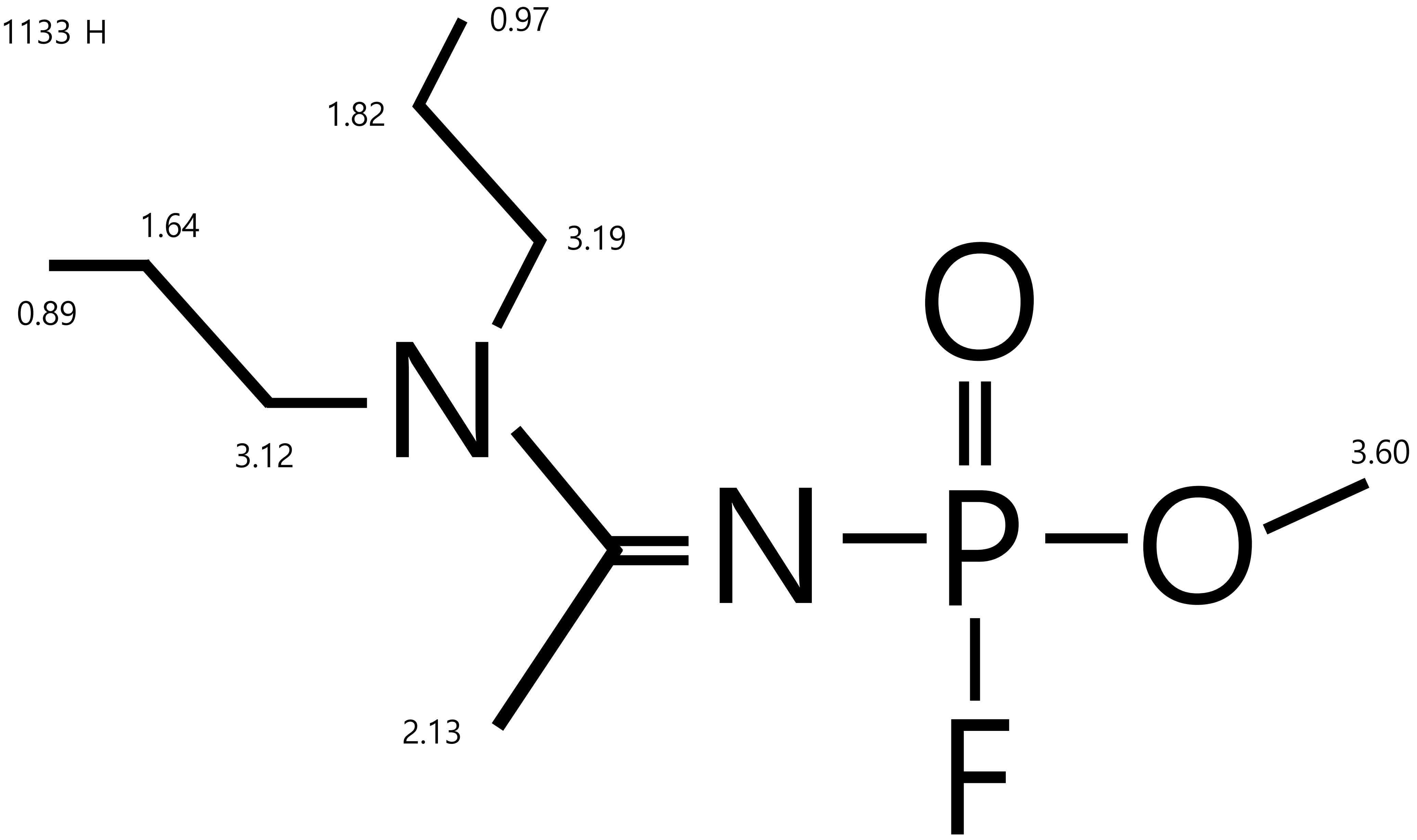

Figure S92. Structure 1133 and its ^{1}H chemical shift

1211 H

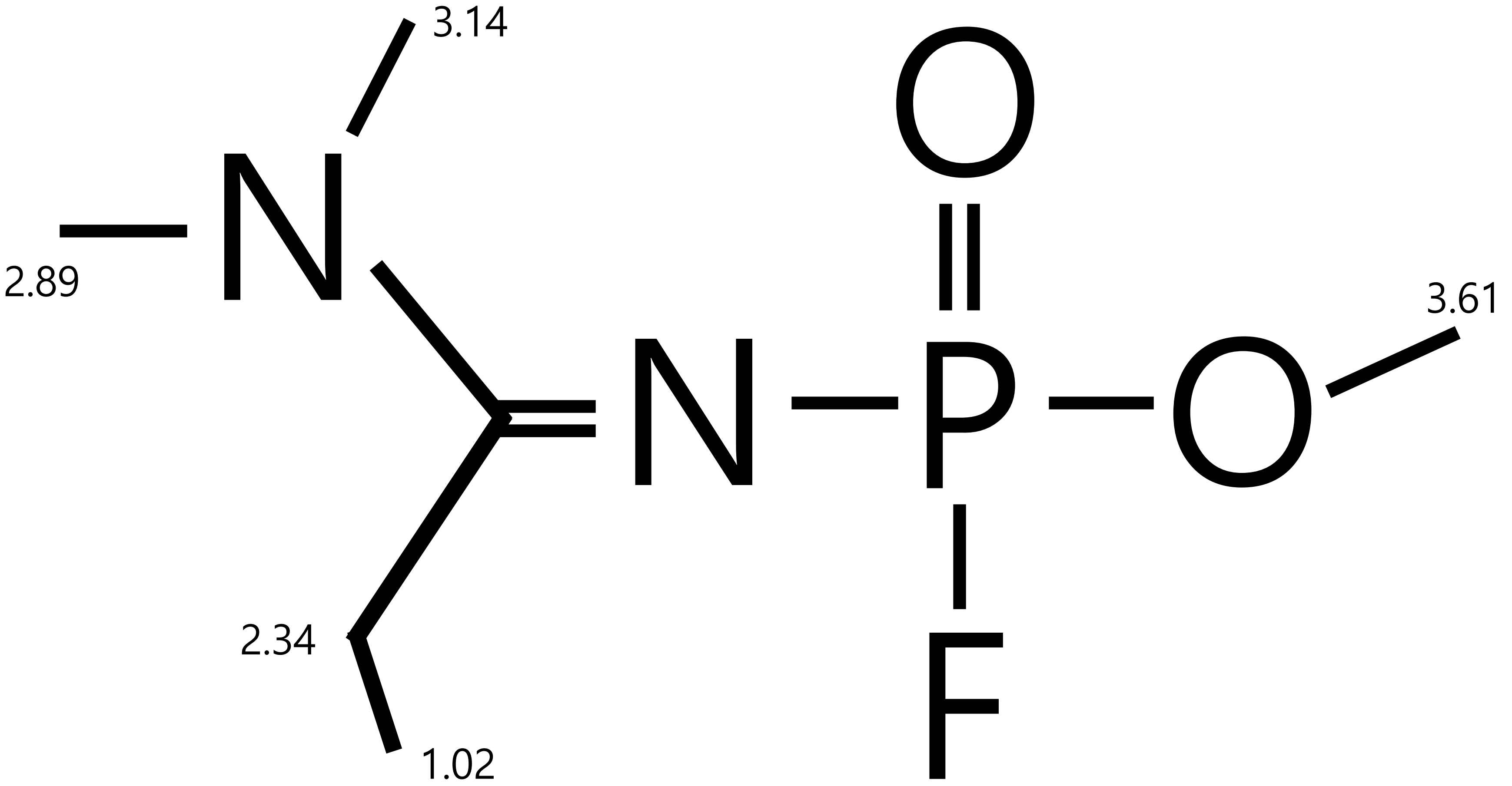

Figure S93. Structure 1211 and its ^{1}H chemical shift

1212 H

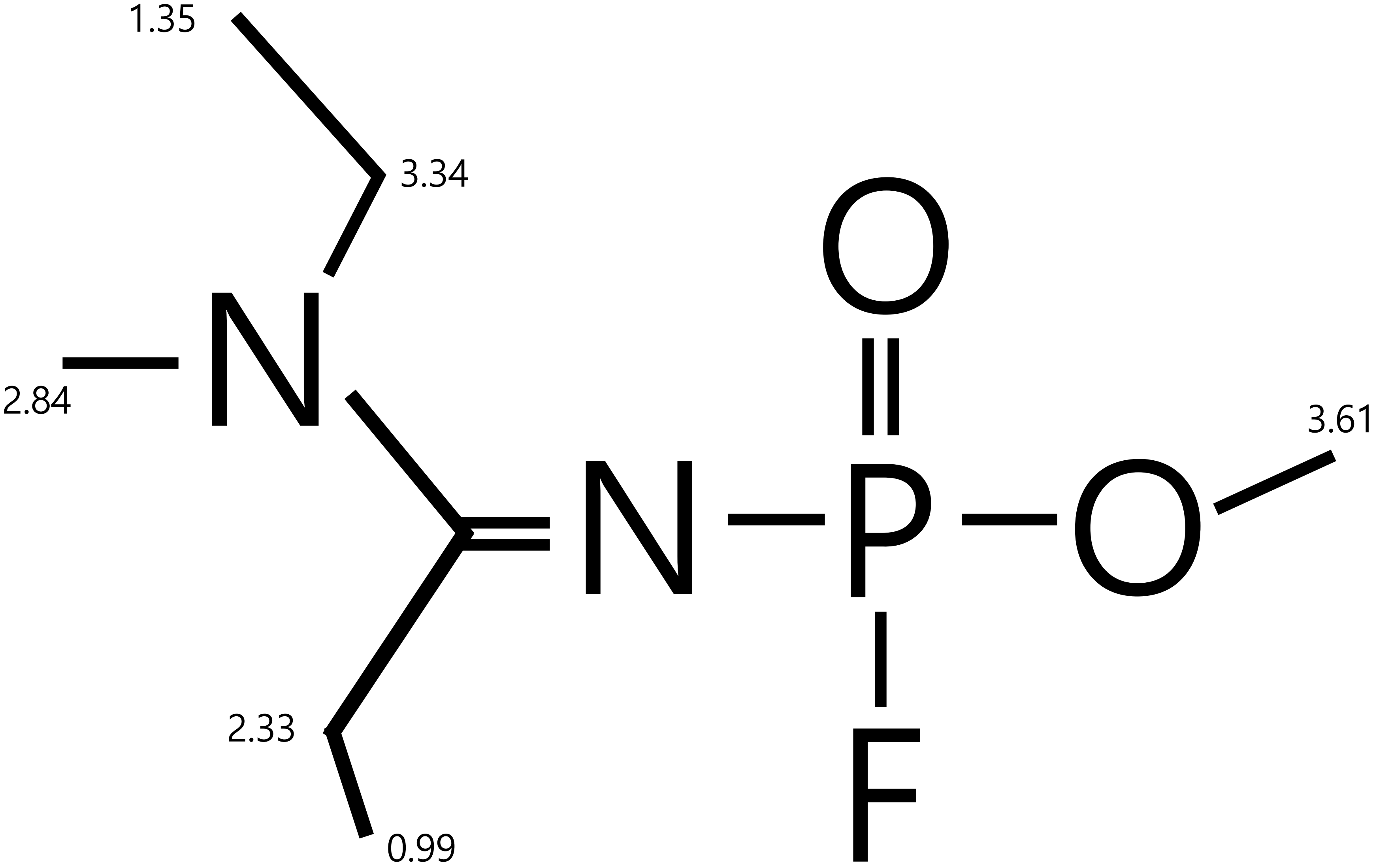

Figure S94. Structure 1212 and its ^{1}H chemical shift

1213 H

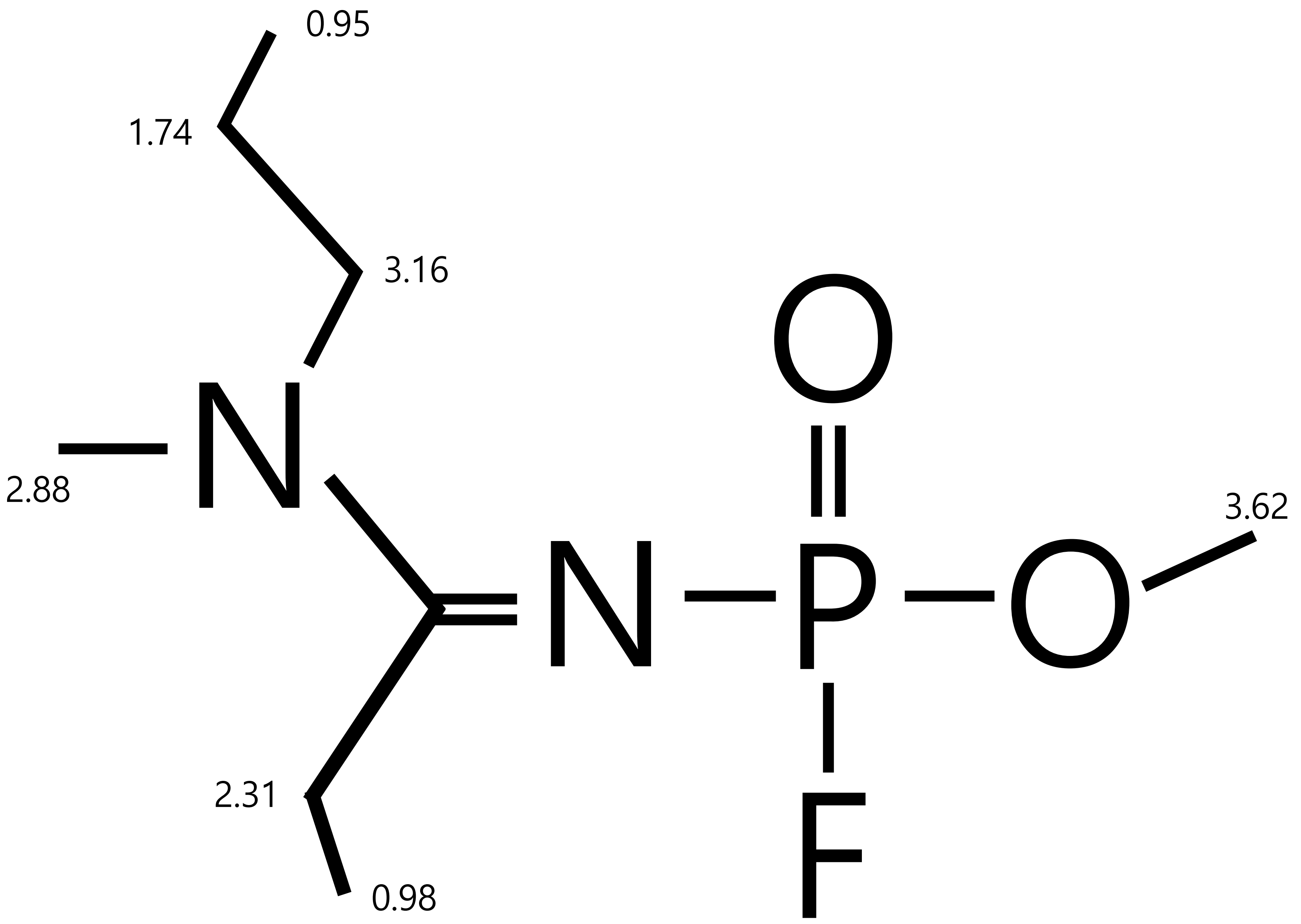

Figure S95. Structure 1213 and its ^{1}H chemical shift

1221 H

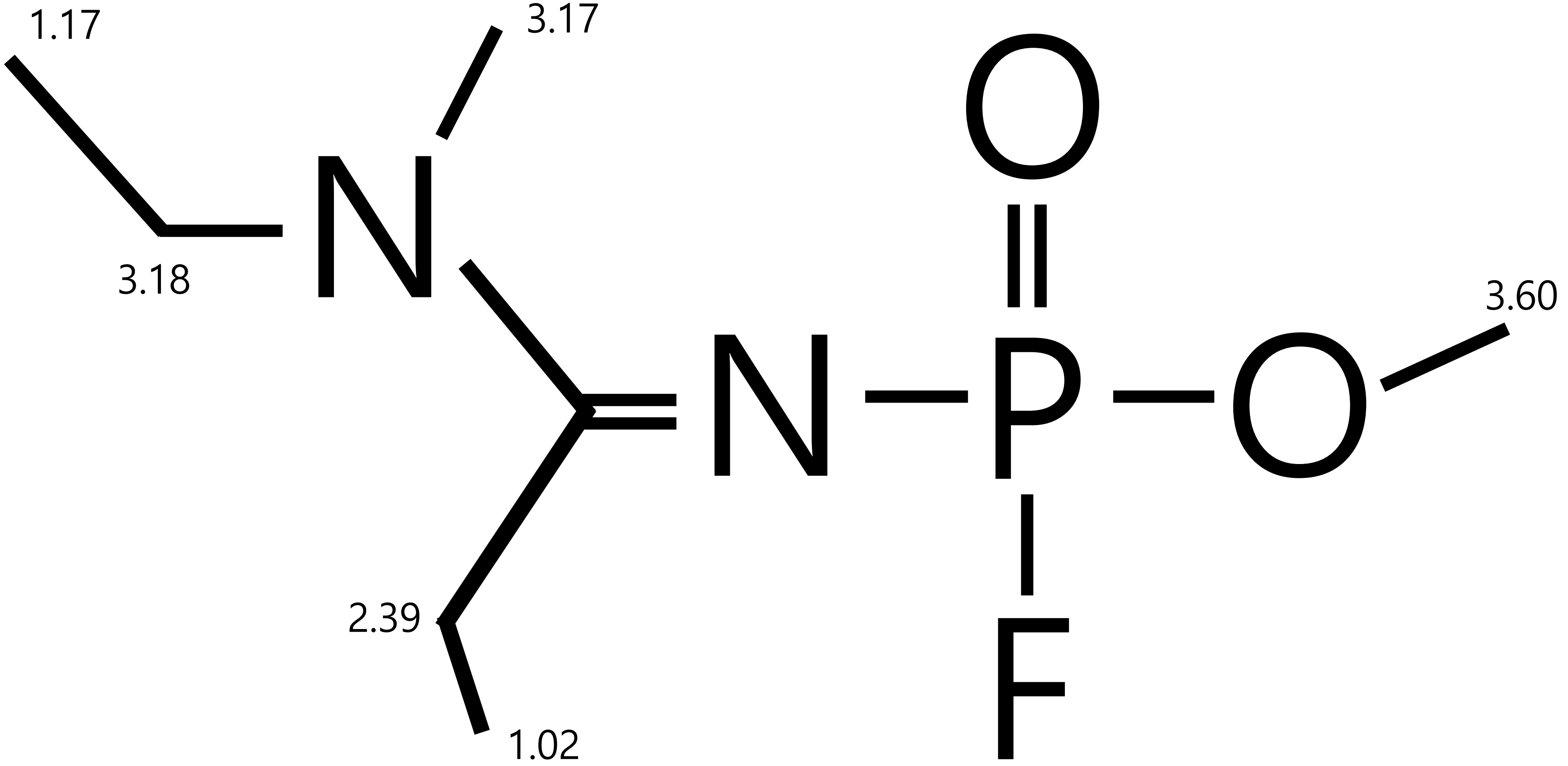

Figure S96. Structure 1221 and its ^{1}H chemical shift

1222 H

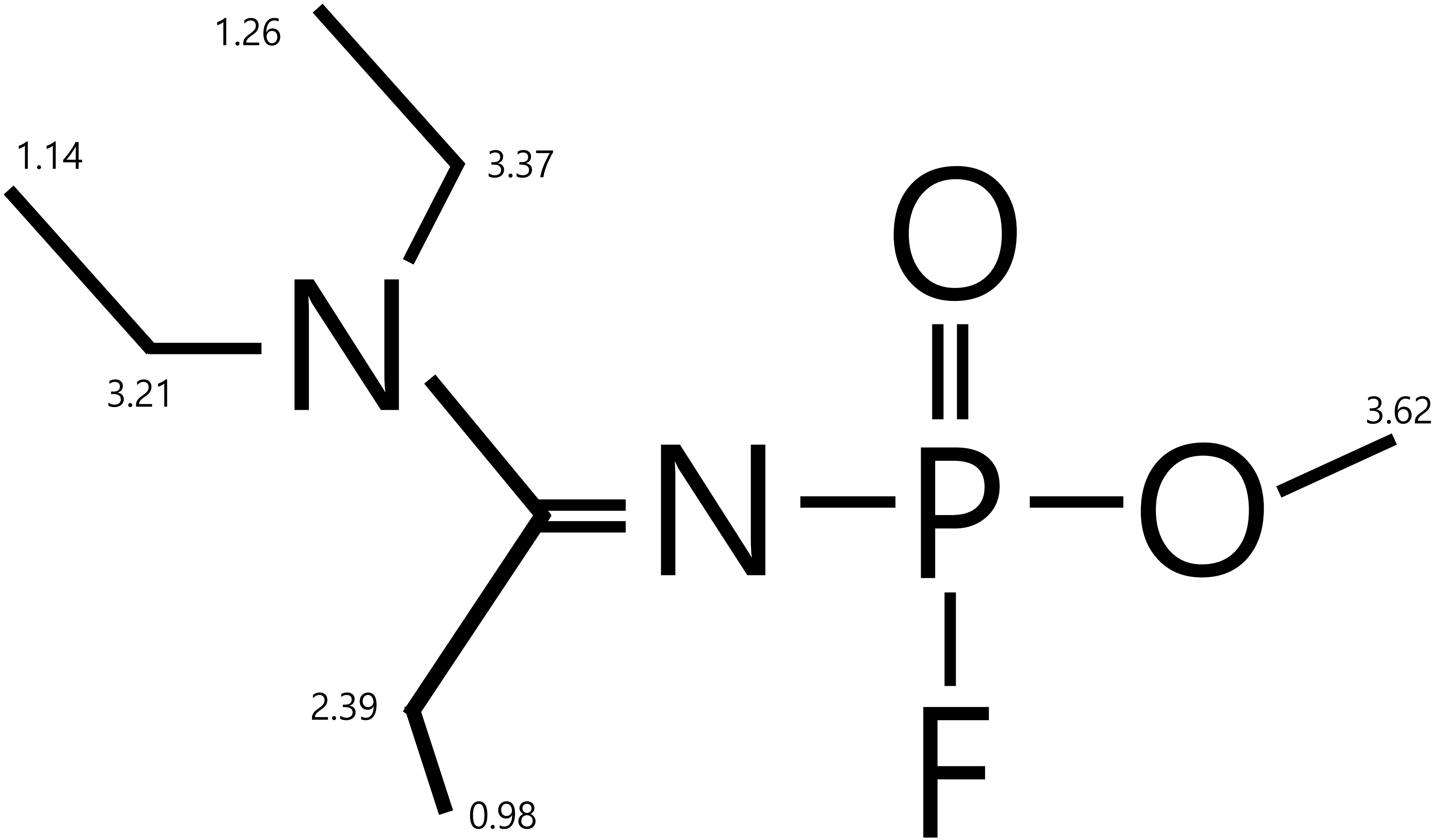

Figure S97. Structure 1222 and its ^{1}H chemical shift

1223 H

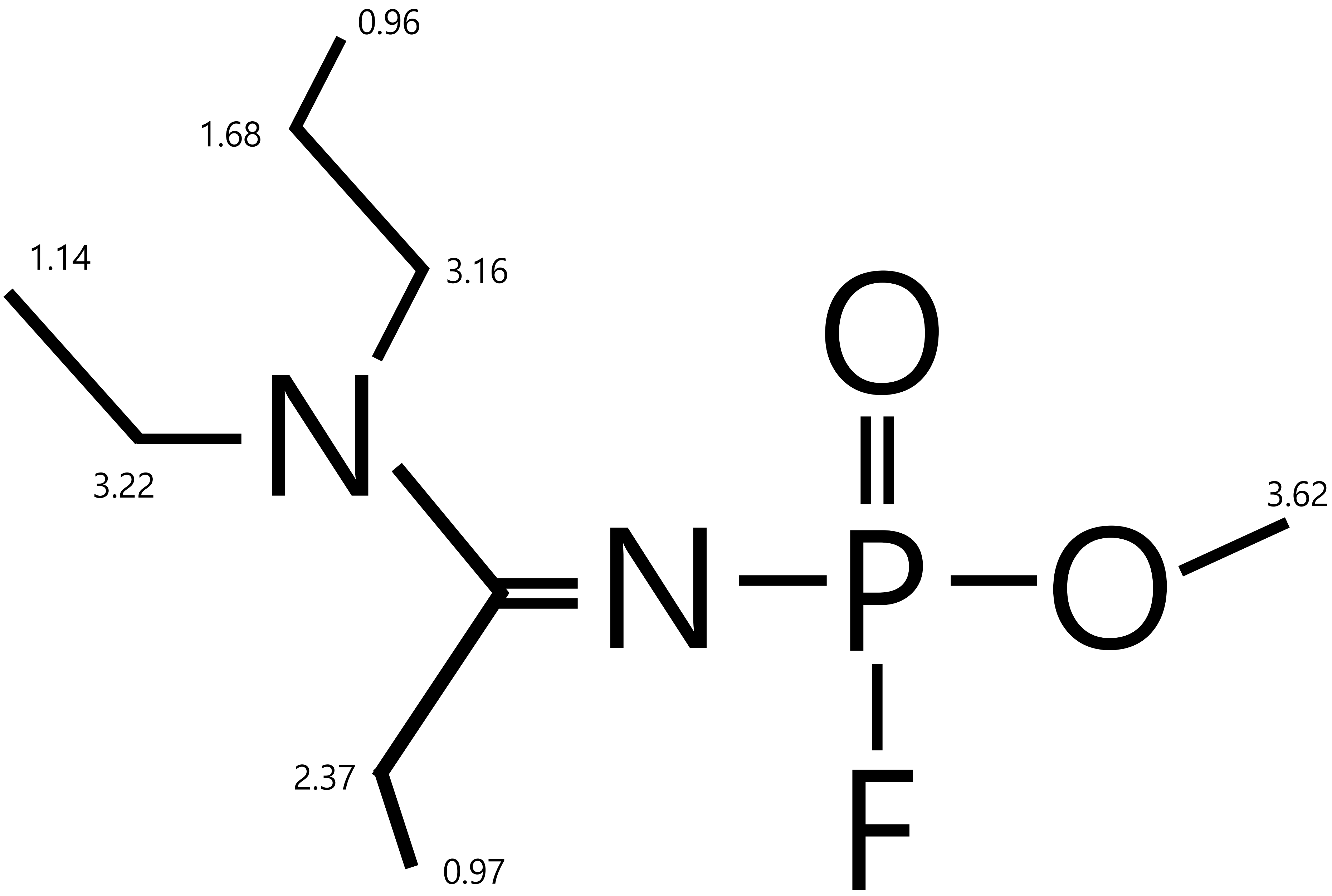

Figure S98. Structure 1223 and its ^{1}H chemical shift

1231 H

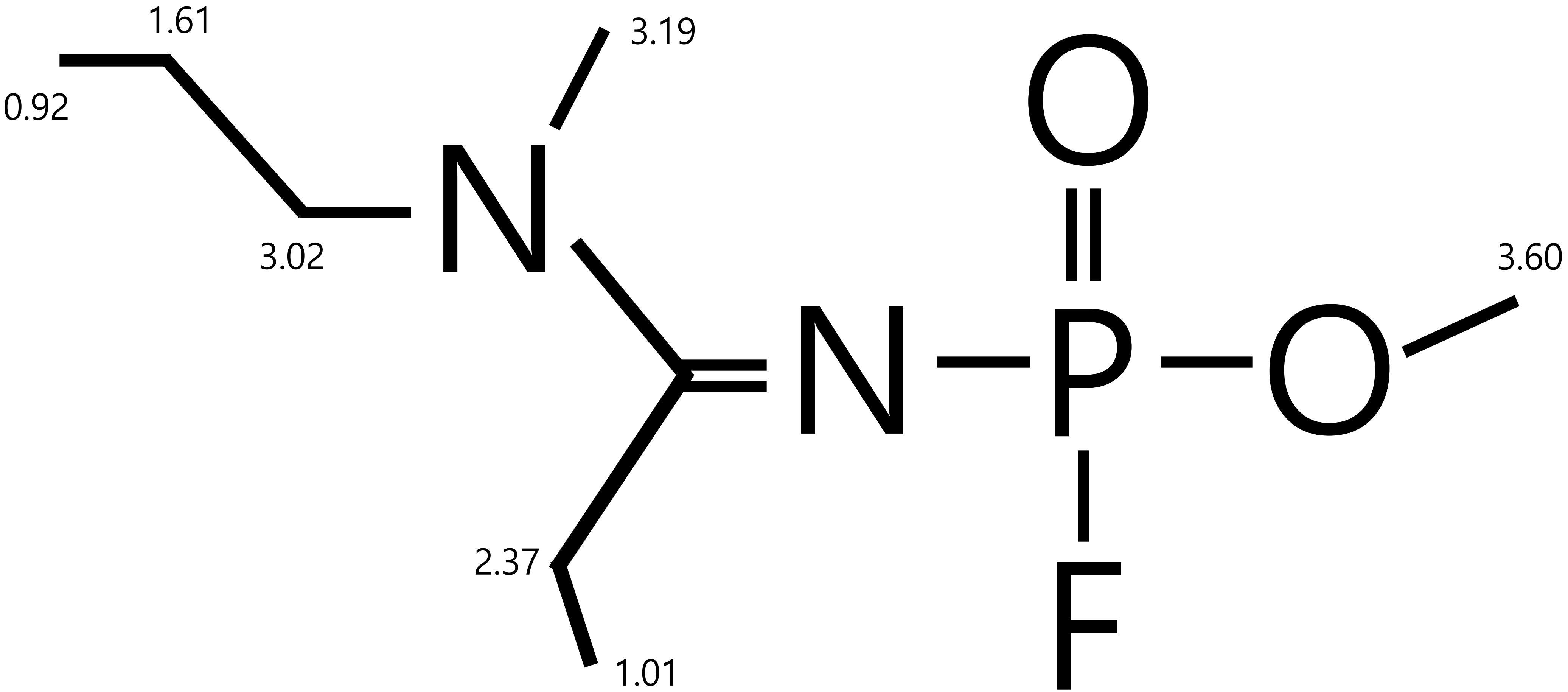

Figure S99. Structure 1231 and its ^{1}H chemical shift

1232 H

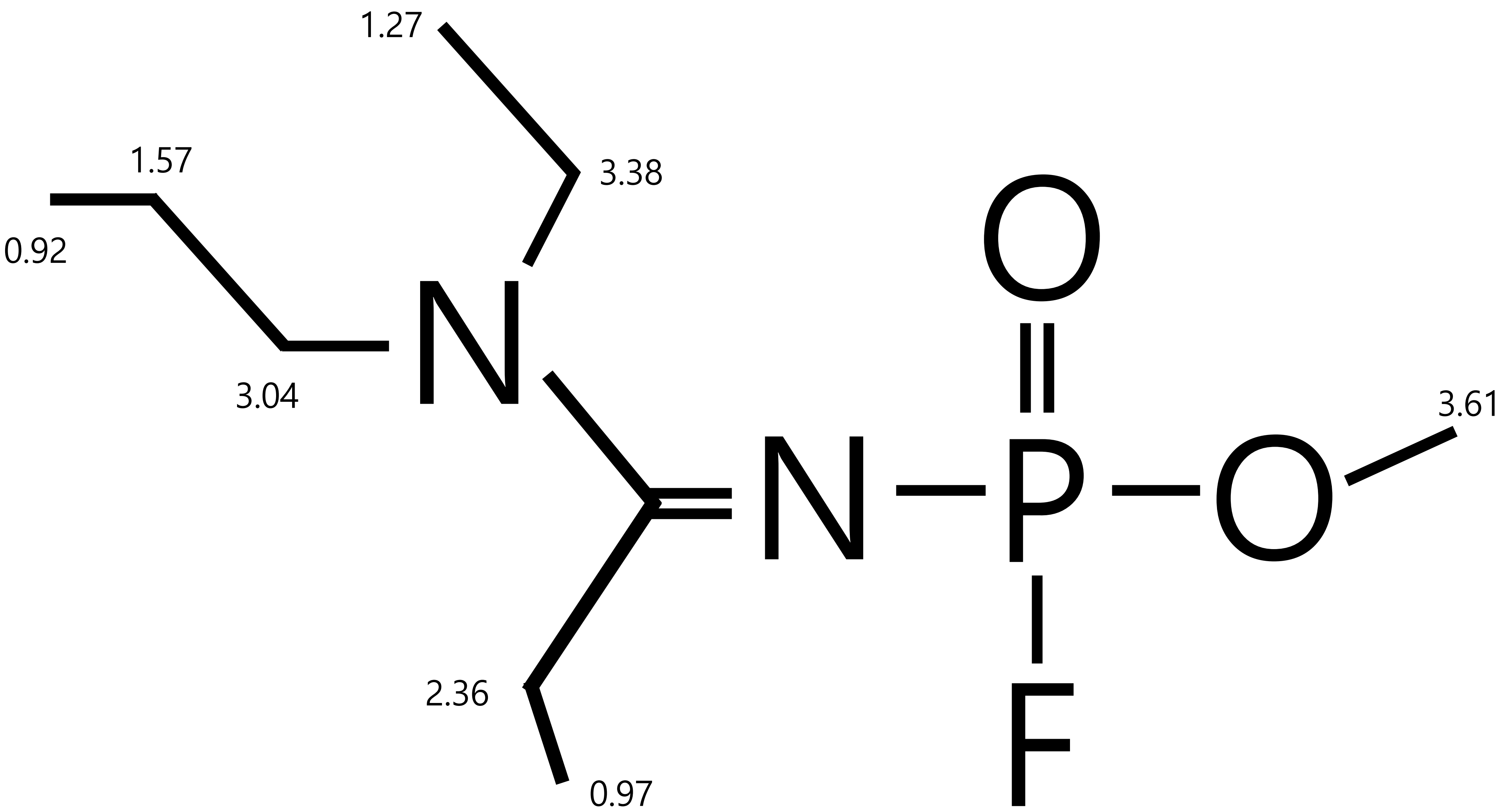

Figure S100. Structure 1232 and its ^{1}H chemical shift

1233 H

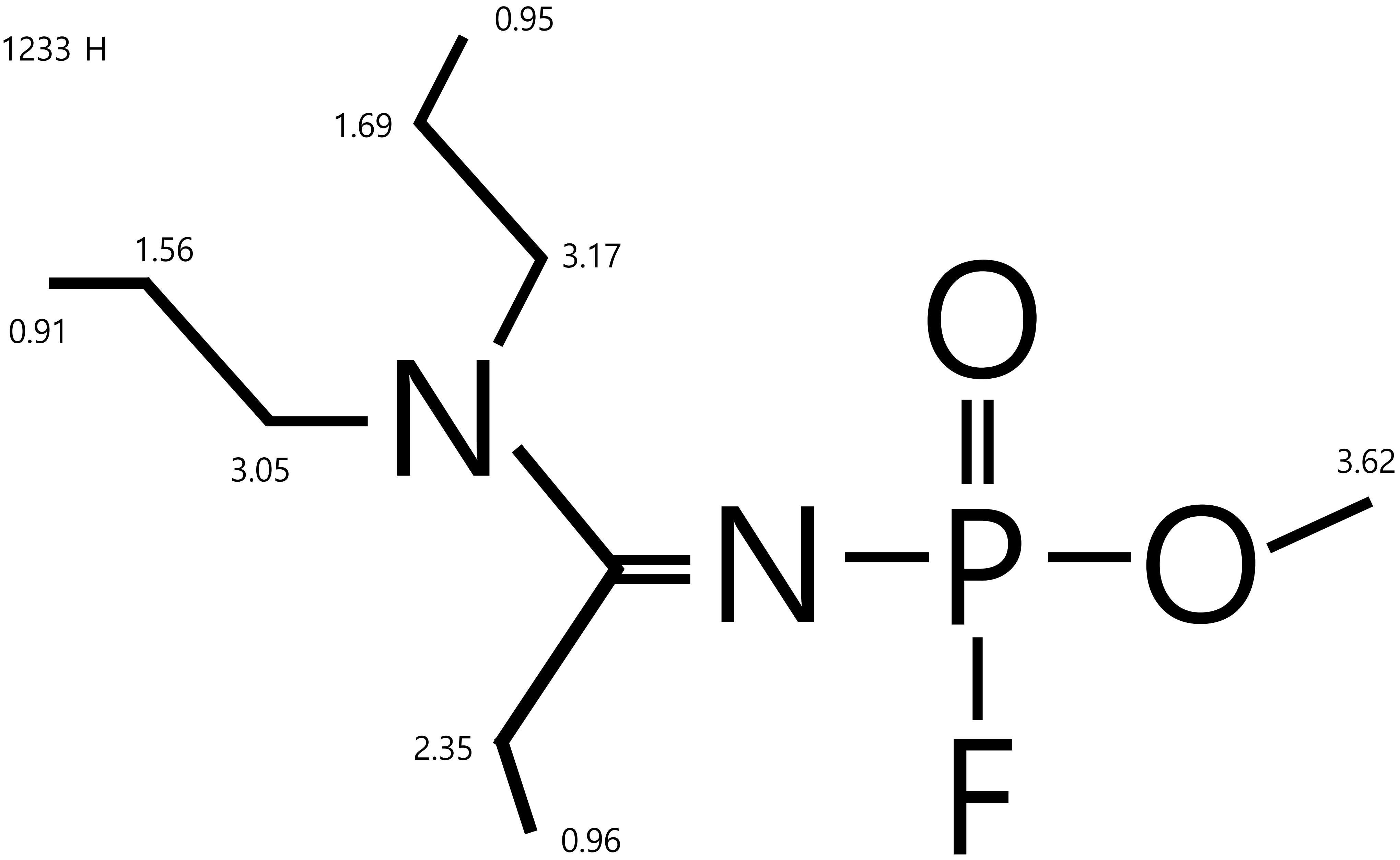

Figure S101. Structure 1233 and its ^{1}H chemical shift

1311 H

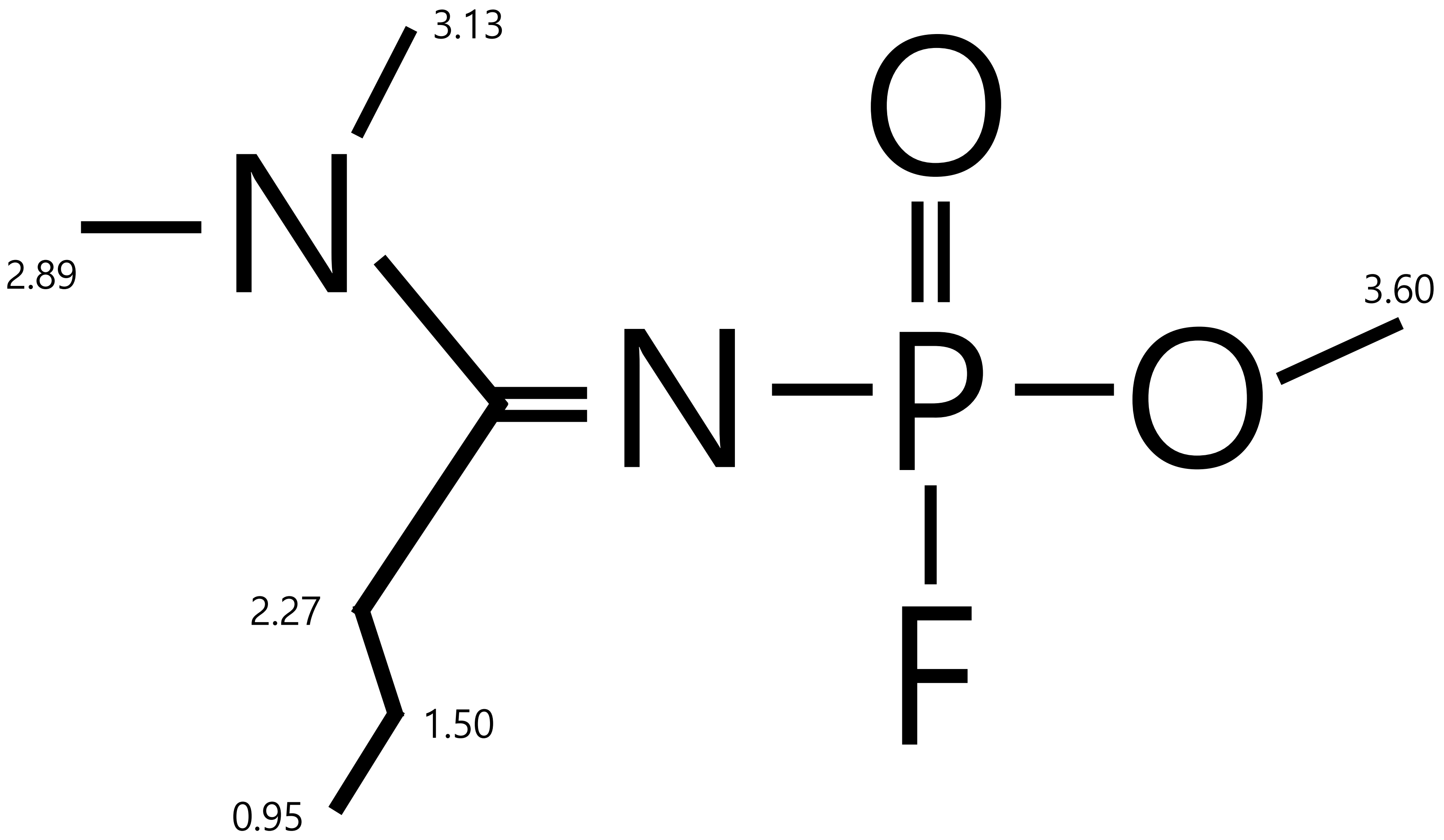

Figure S102. Structure 1311 and its ^{1}H chemical shift

1312 H

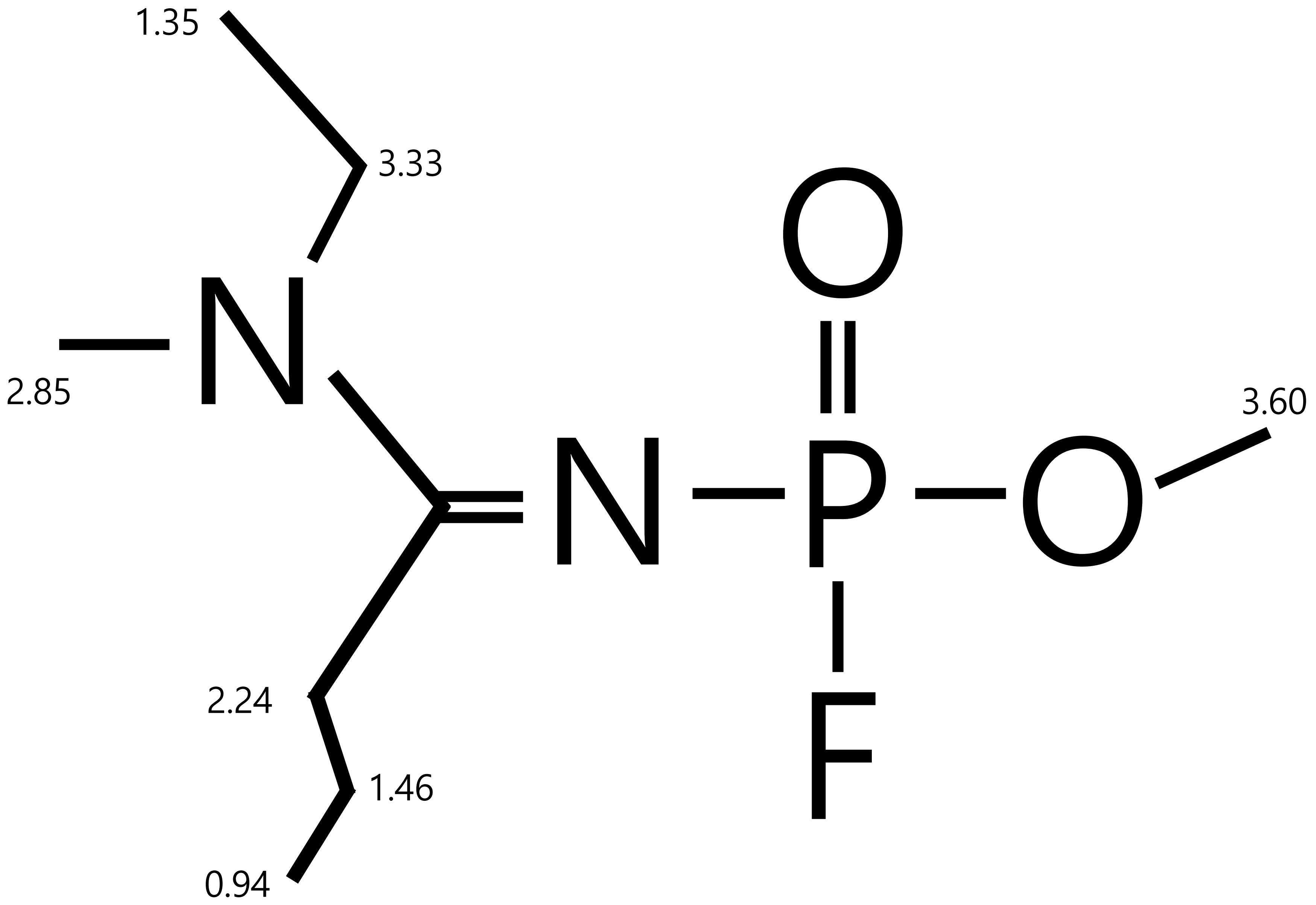

Figure S104. Structure 1312 and its ^{1}H chemical shift

1313 H

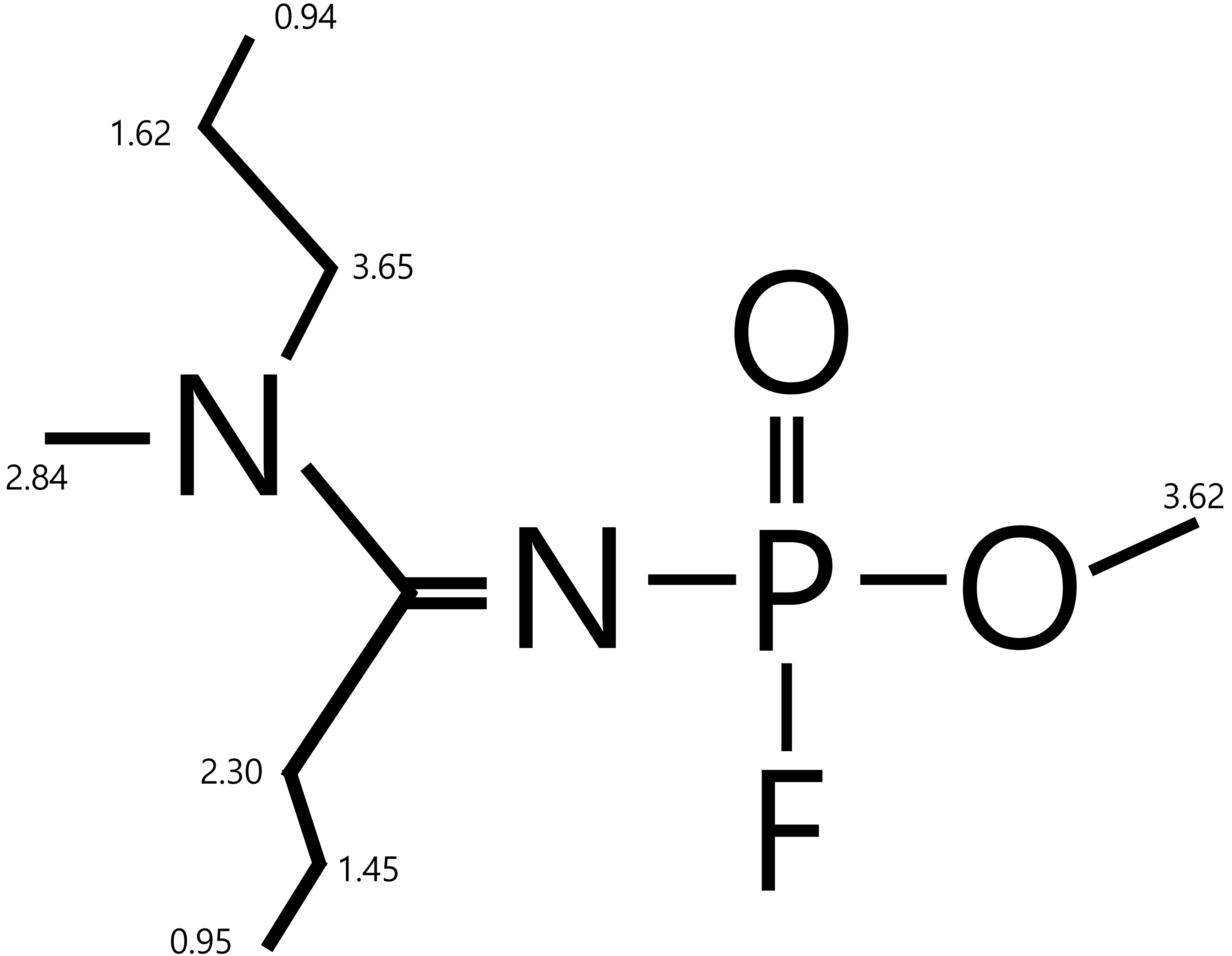

Figure S104. Structure 1313 and its ^{1}H chemical shift

1321 H

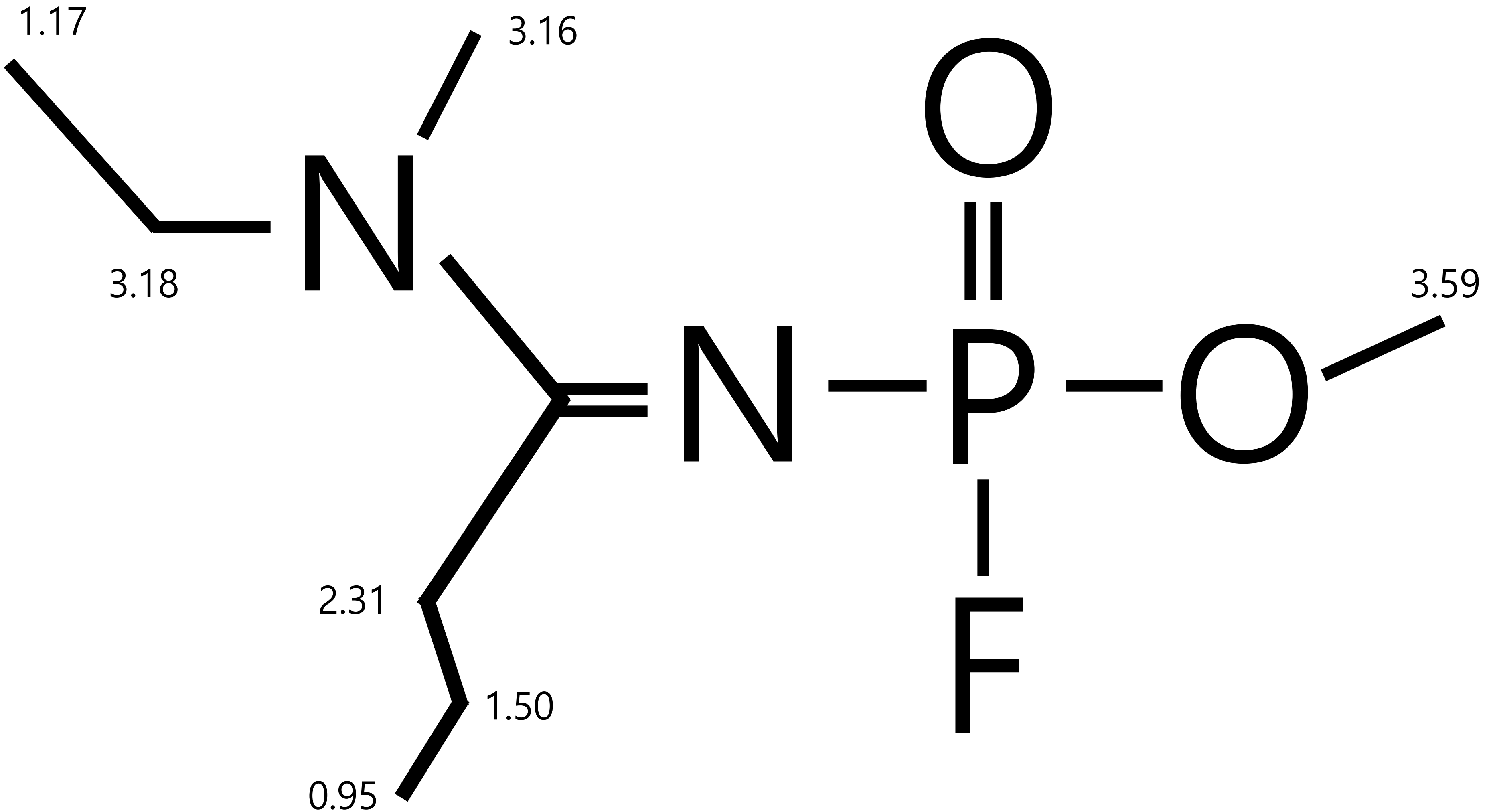

Figure S105. Structure 1321 and its ¹H chemical shift

1322 H

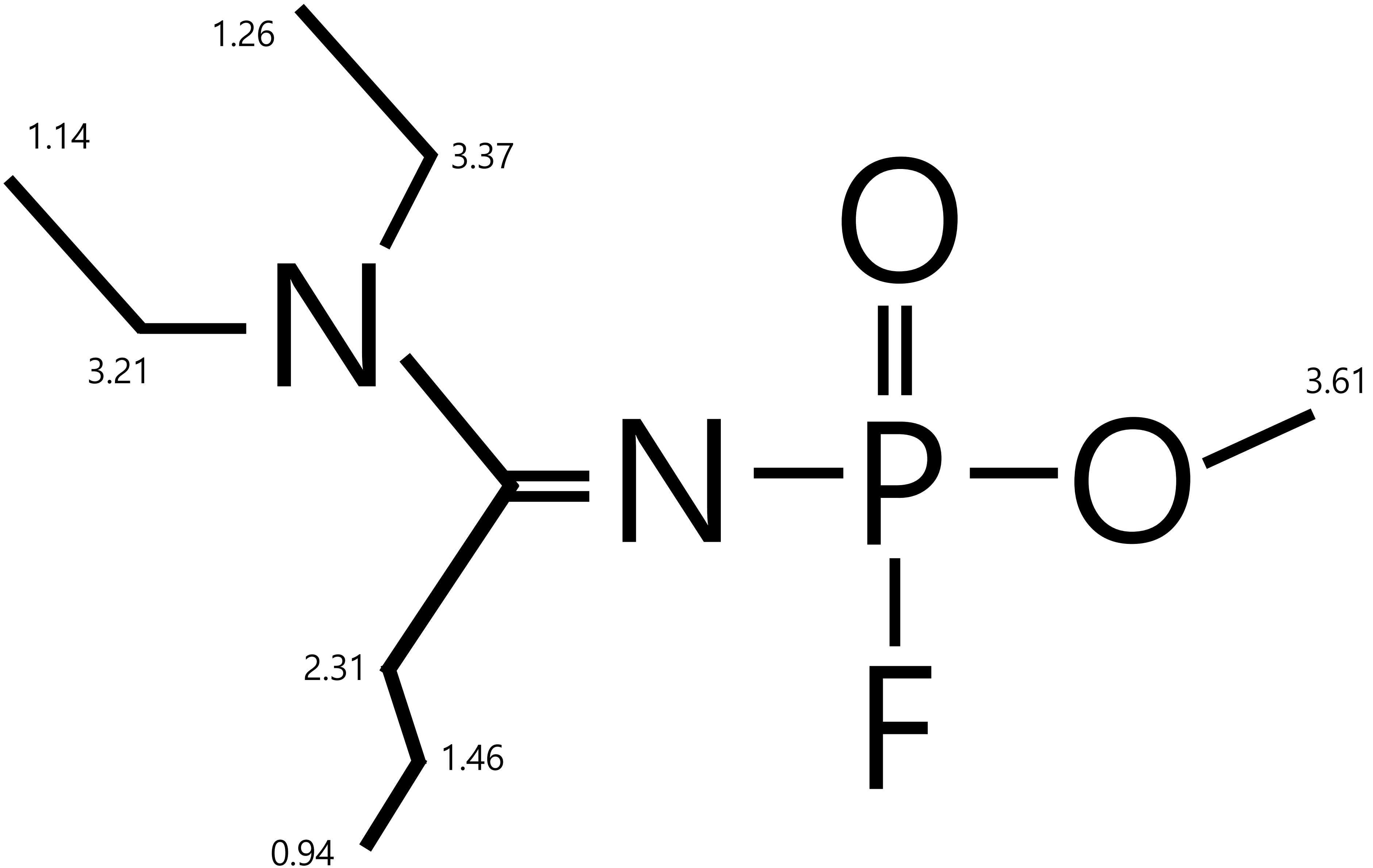

Figure S106. Structure 1322 and its ^{1}H chemical shift

1323 H

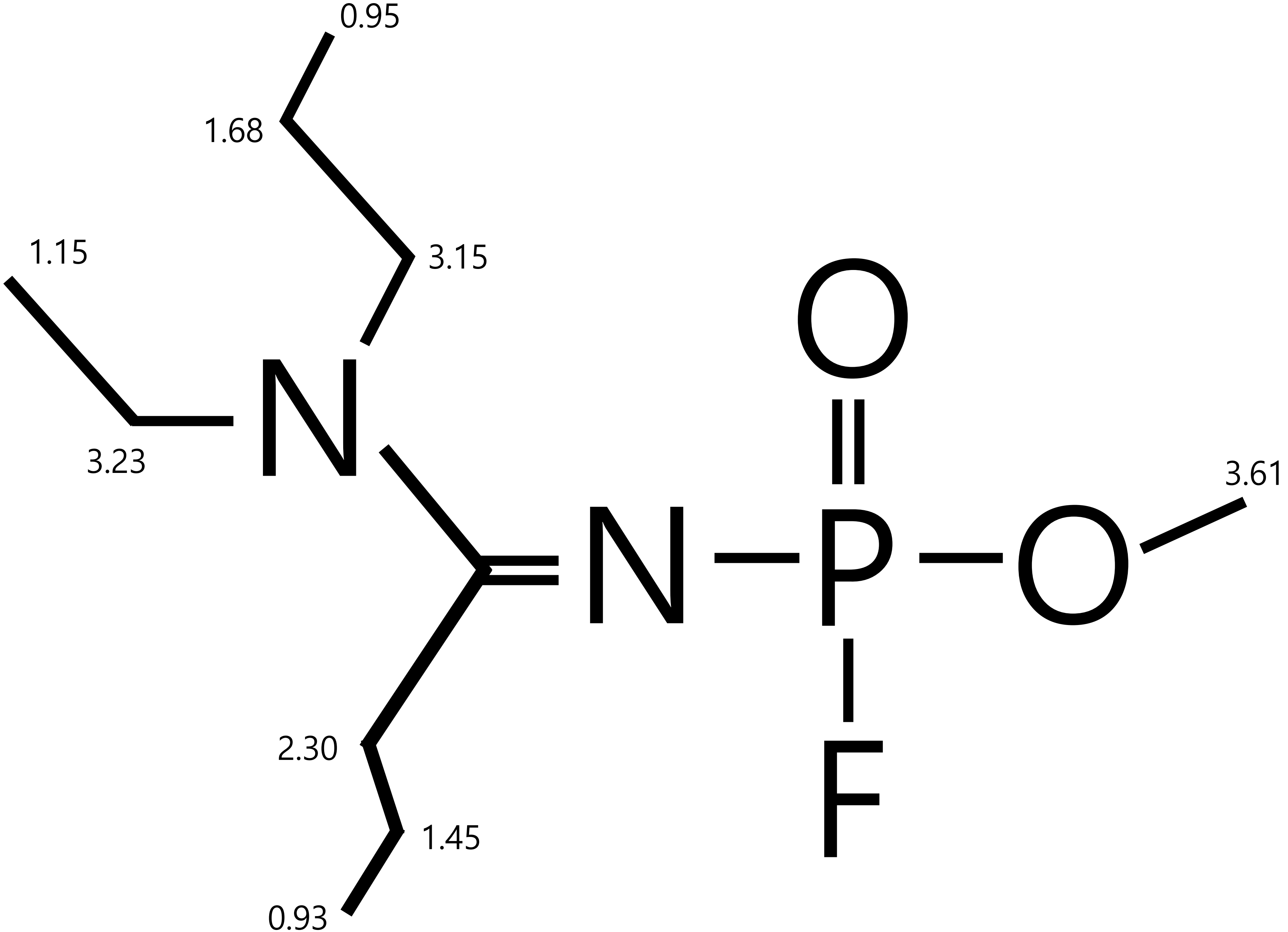

Figure S107. Structure 1323 and its ¹H chemical shift

1331 H

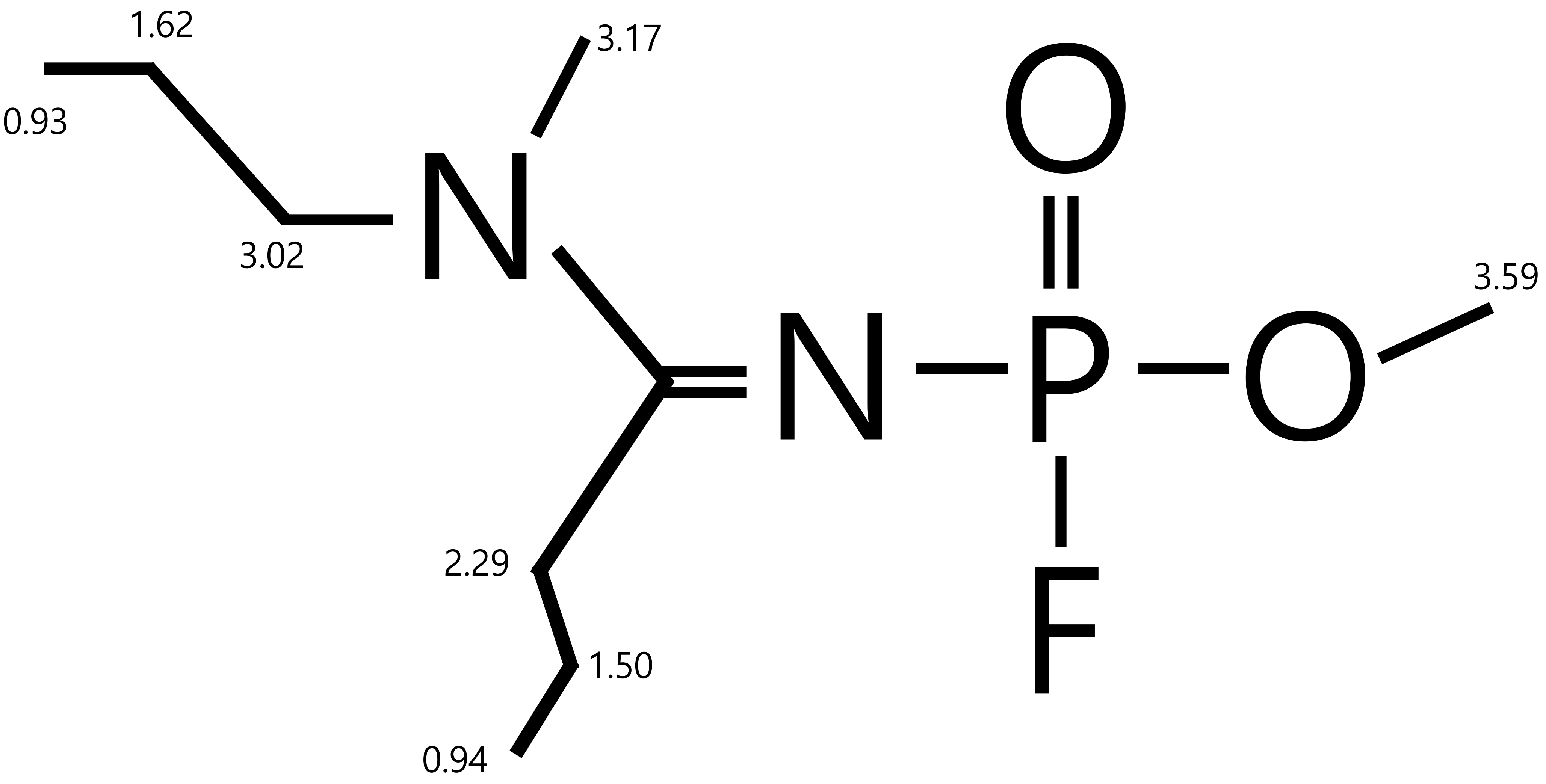

Figure S108. Structure 1331 and its ^{1}H chemical shift

1332 H

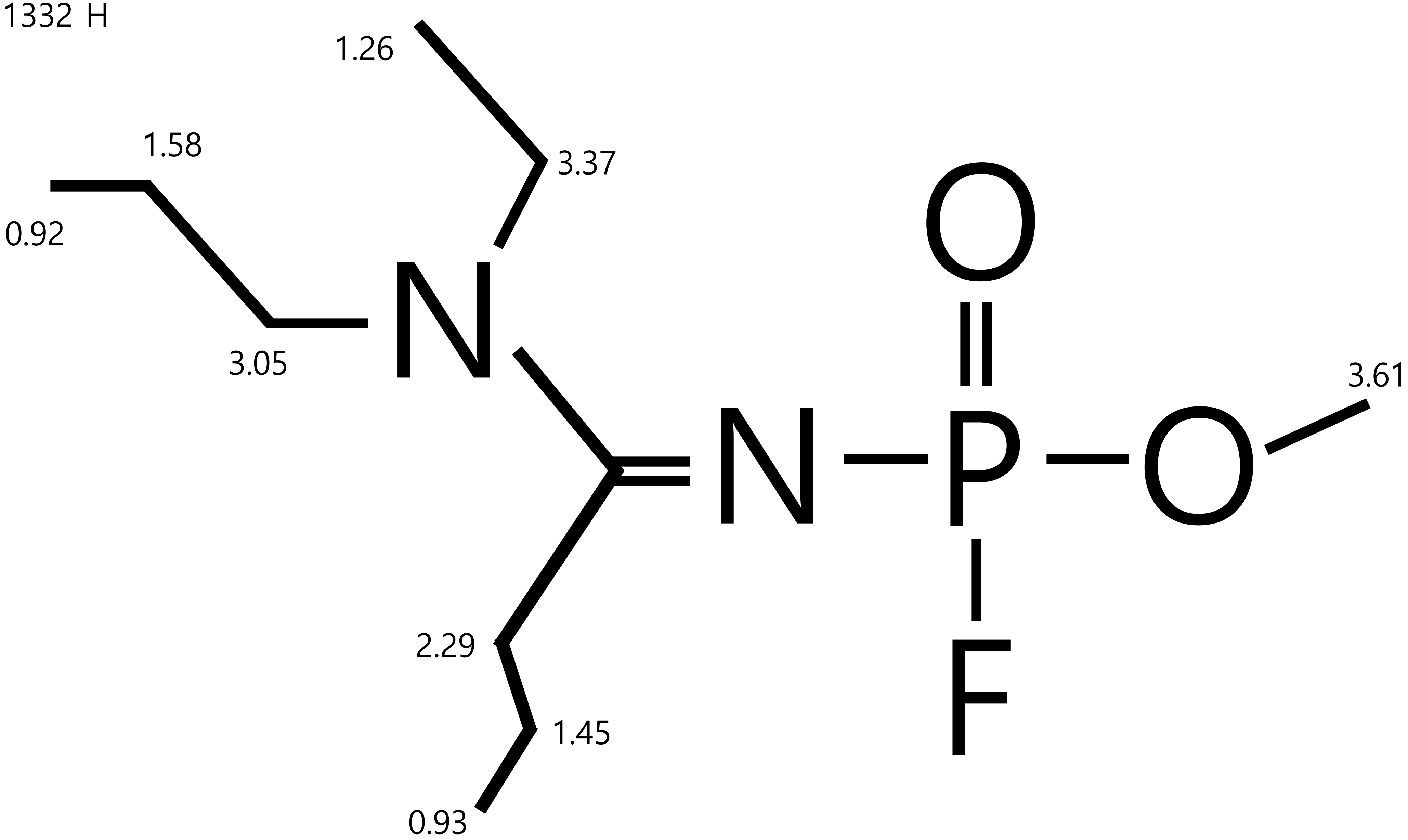

Figure S109. Structure 1332 and its ^{1}H chemical shift

1333 H

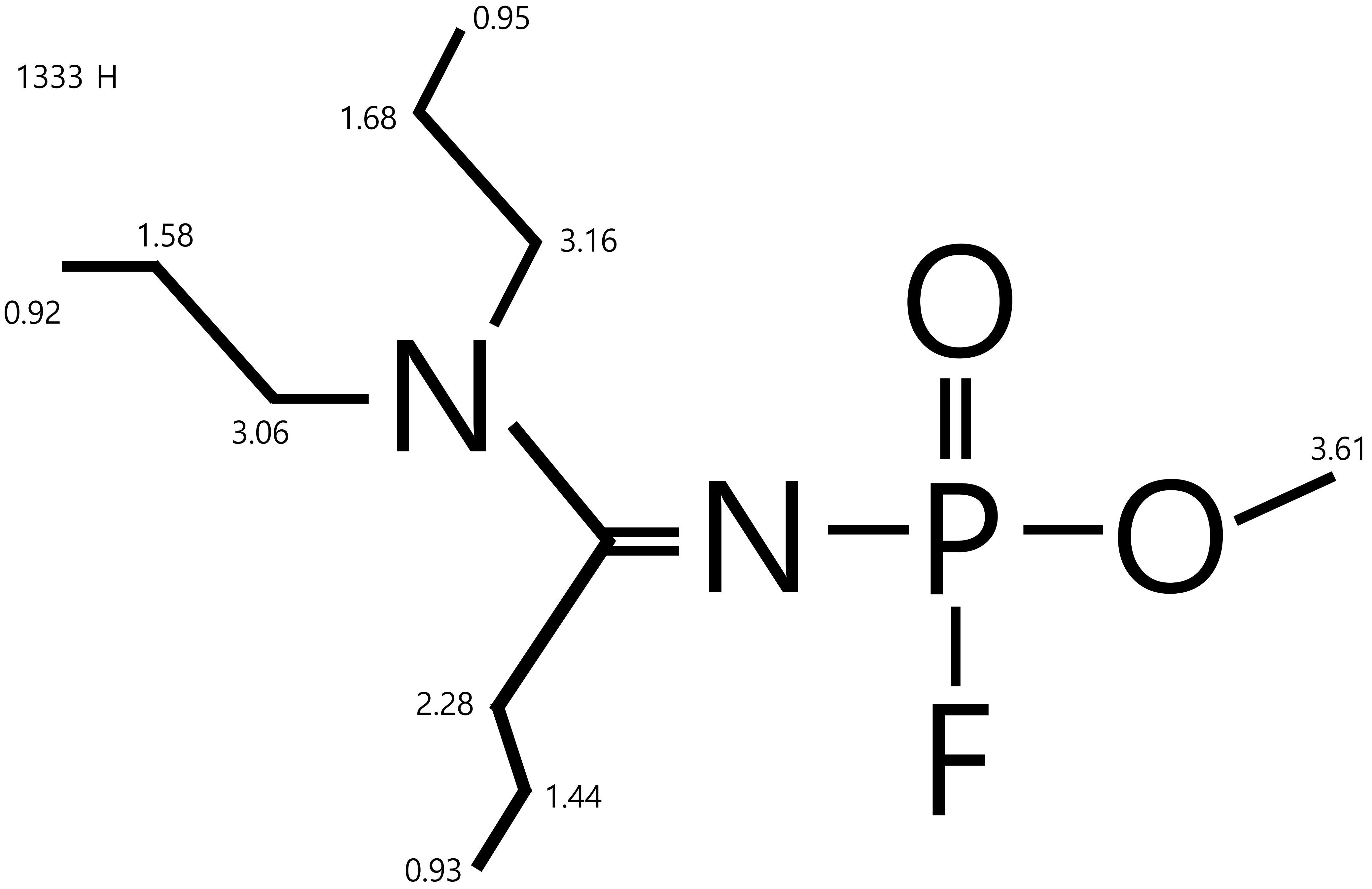

Figure S110. Structure 1333 and its ^{1}H chemical shift

2111 H

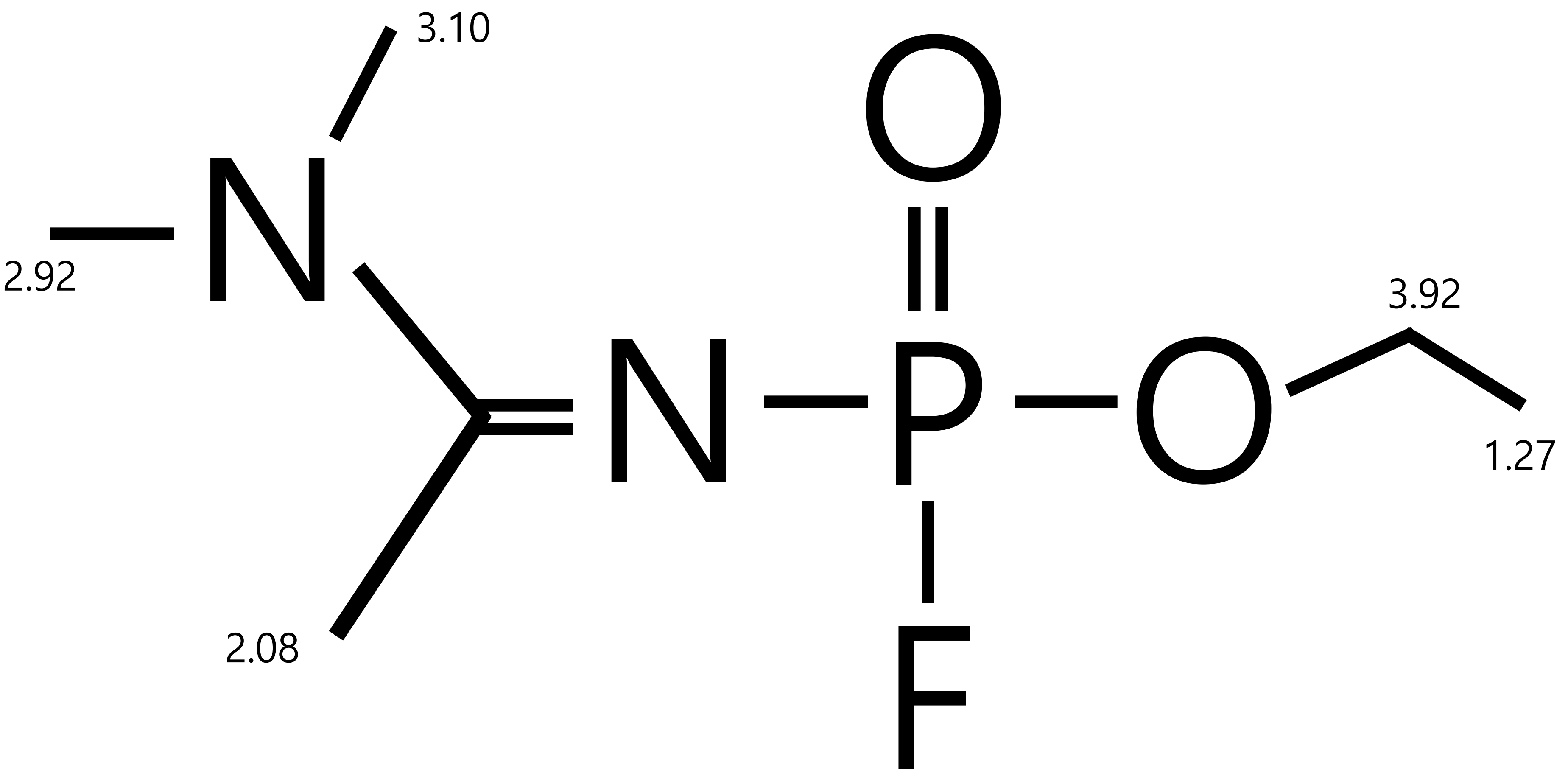

Figure S111. Structure 2111 and its ^{1}H chemical shift

2112 H

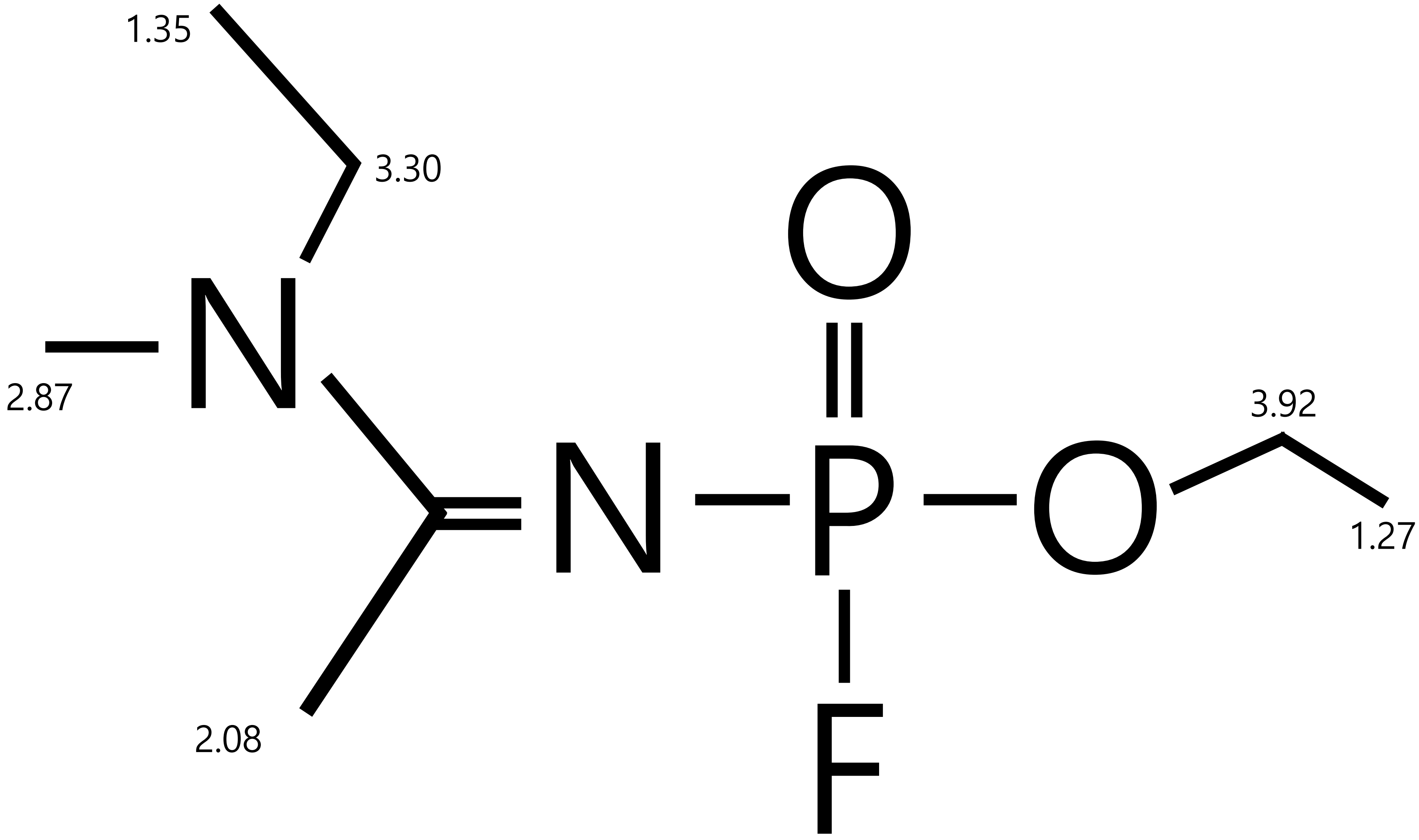

Figure S112. Structure 2112 and its ^{1}H chemical shift

2113 H

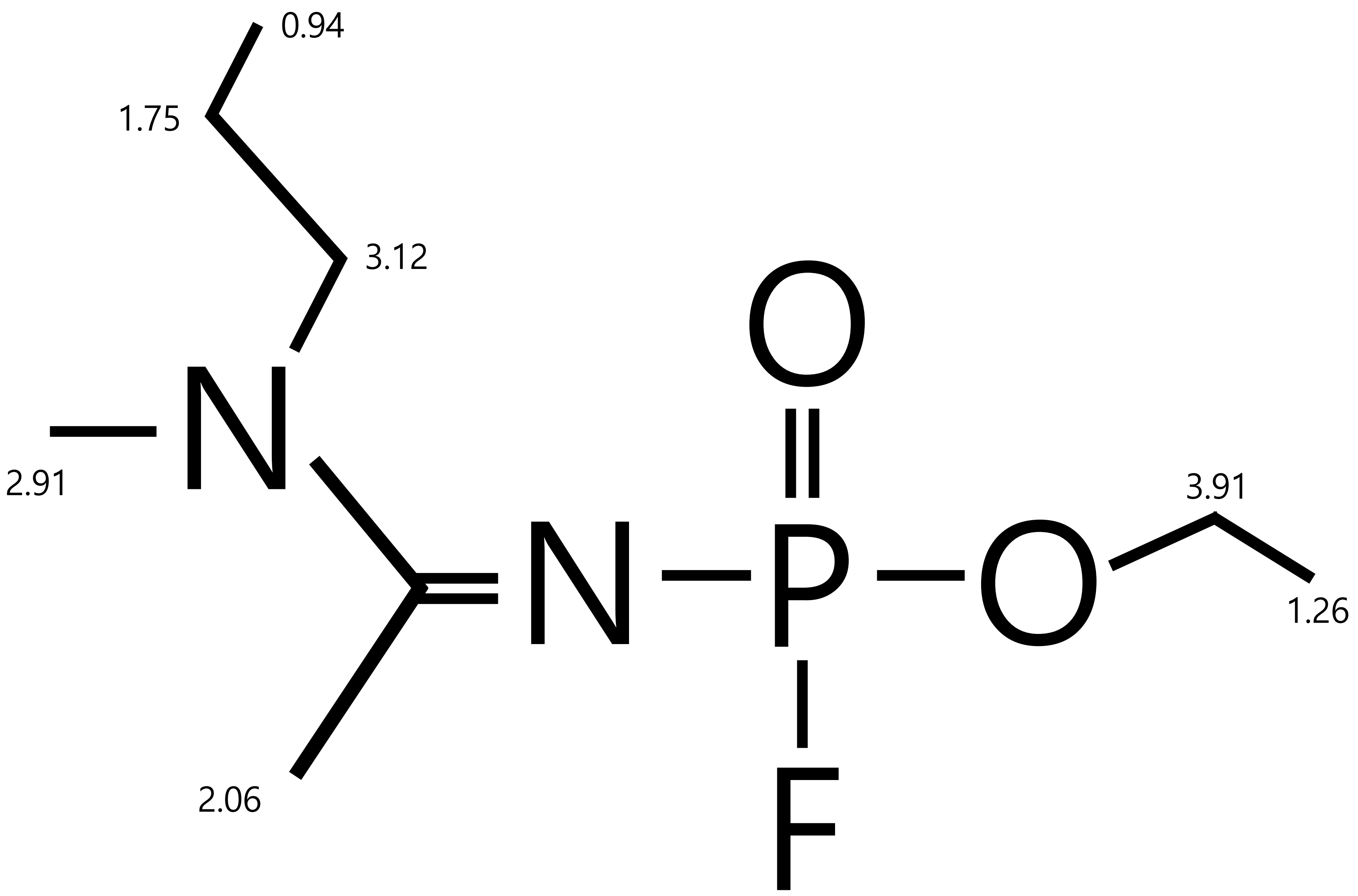

Figure S113. Structure 2113 and its ^{1}H chemical shift

2121 H

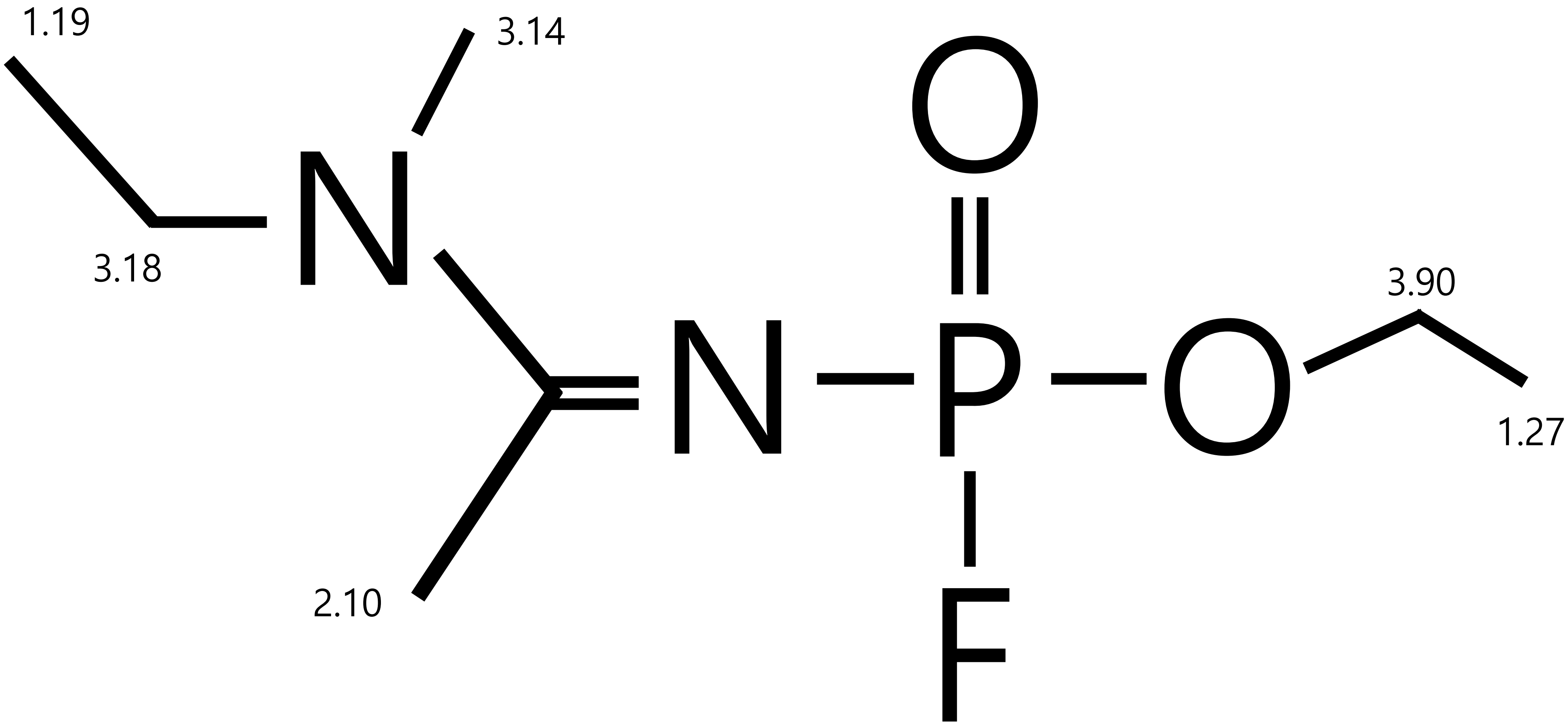

Figure S114. Structure 2121 and its ^{1}H chemical shift

2122 H

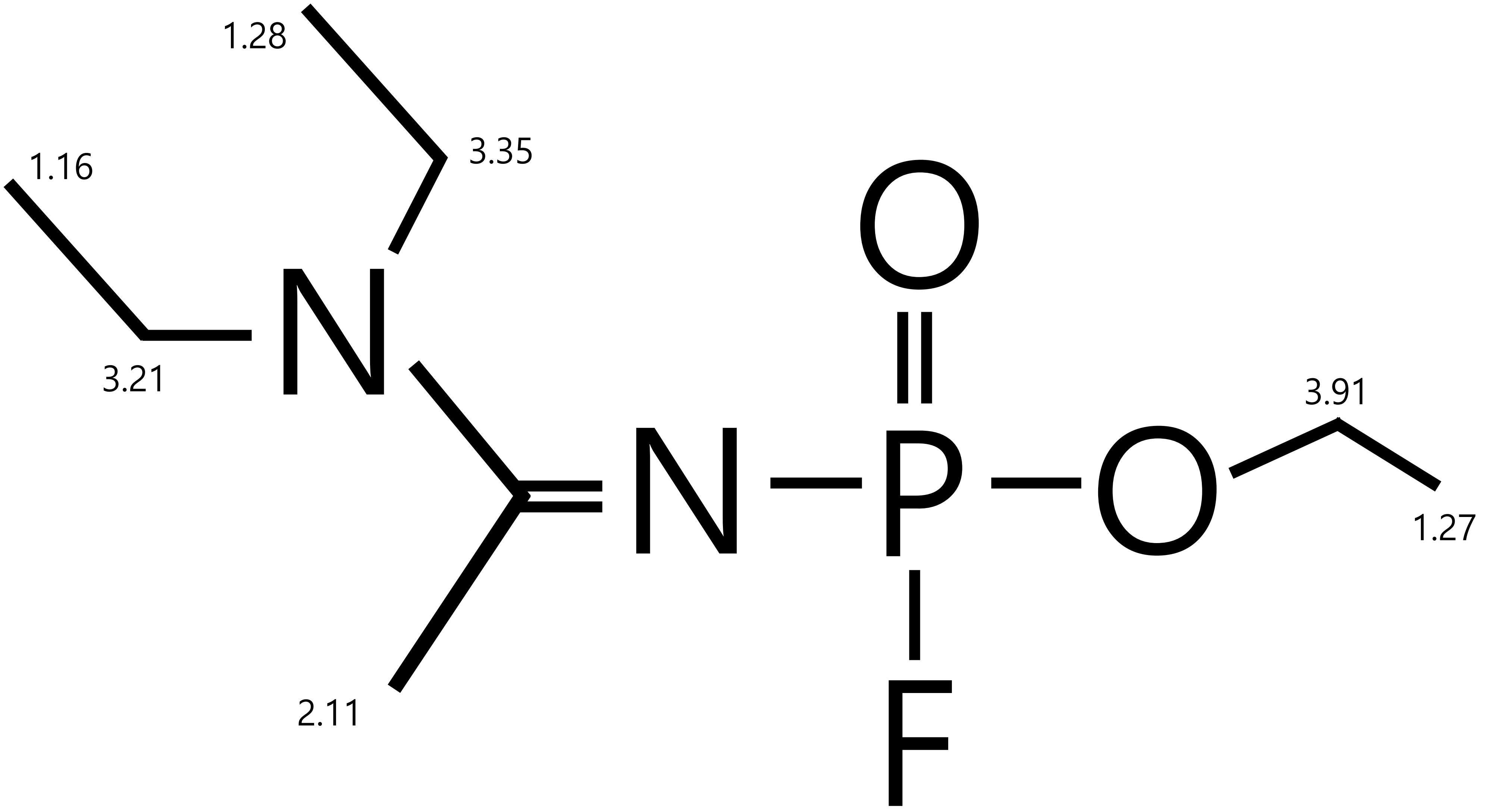

Figure S115. Structure 2122 and its ^{1}H chemical shift

2123 H

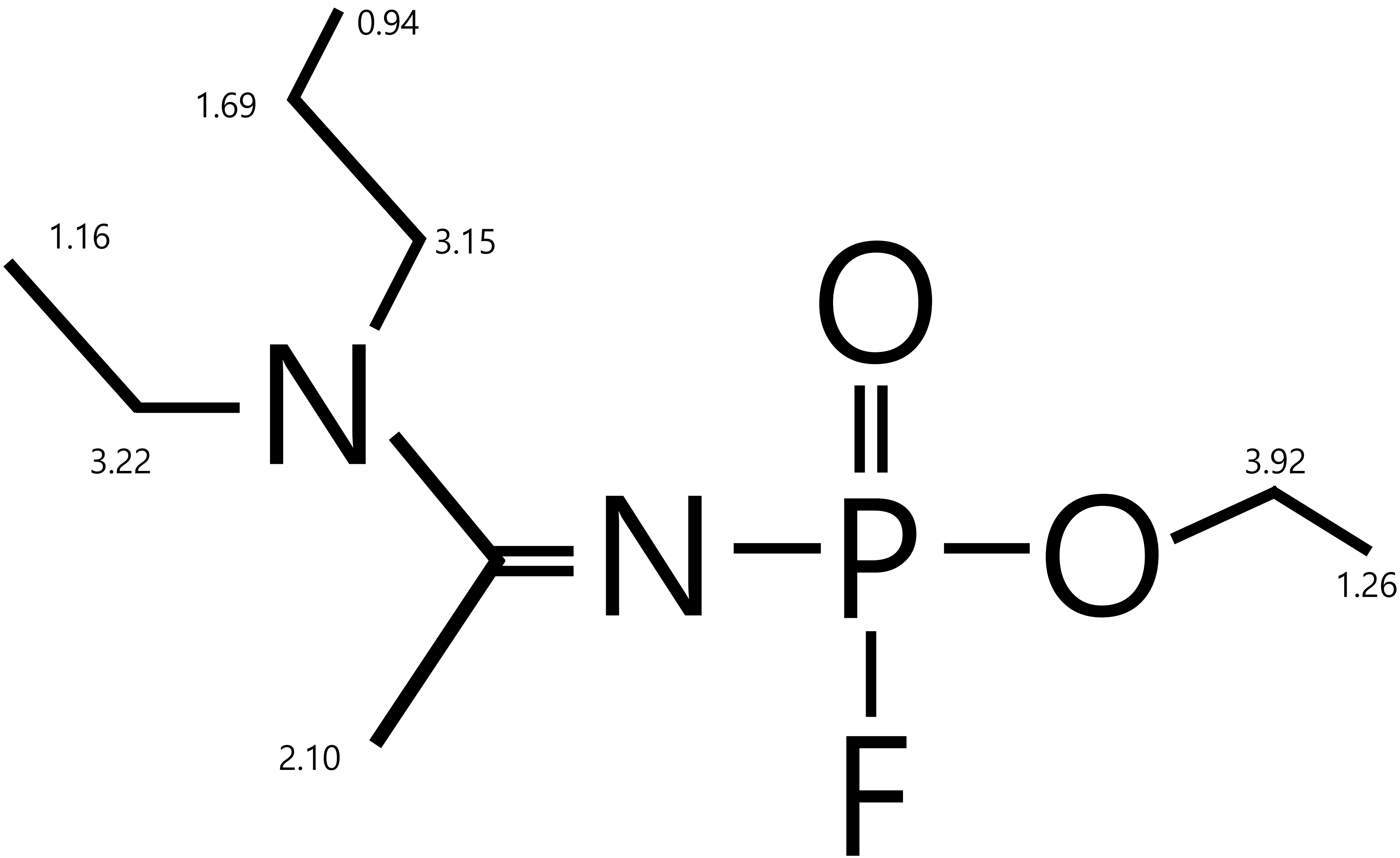

Figure S116. Structure 2123 and its ^{1}H chemical shift

2131 H

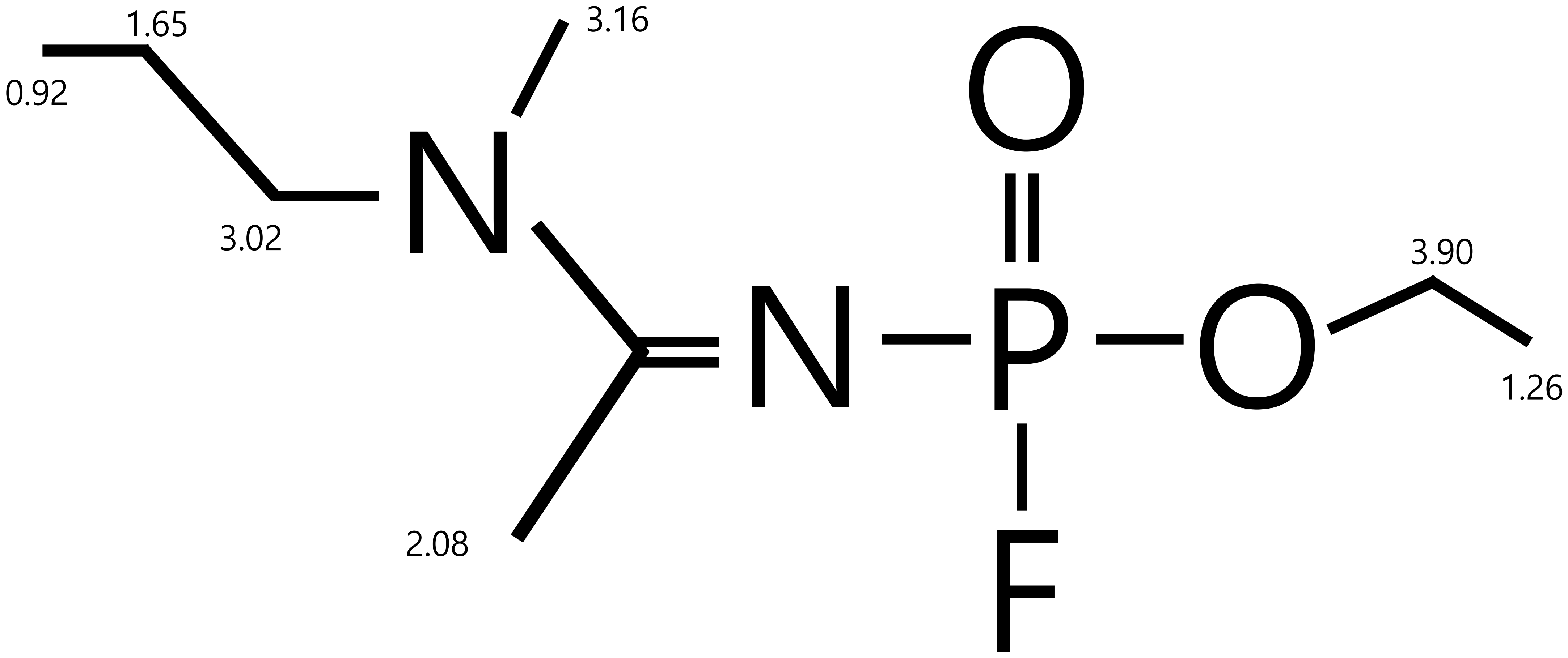

Figure S117. Structure 2131 and its ^{1}H chemical shift

2132 H

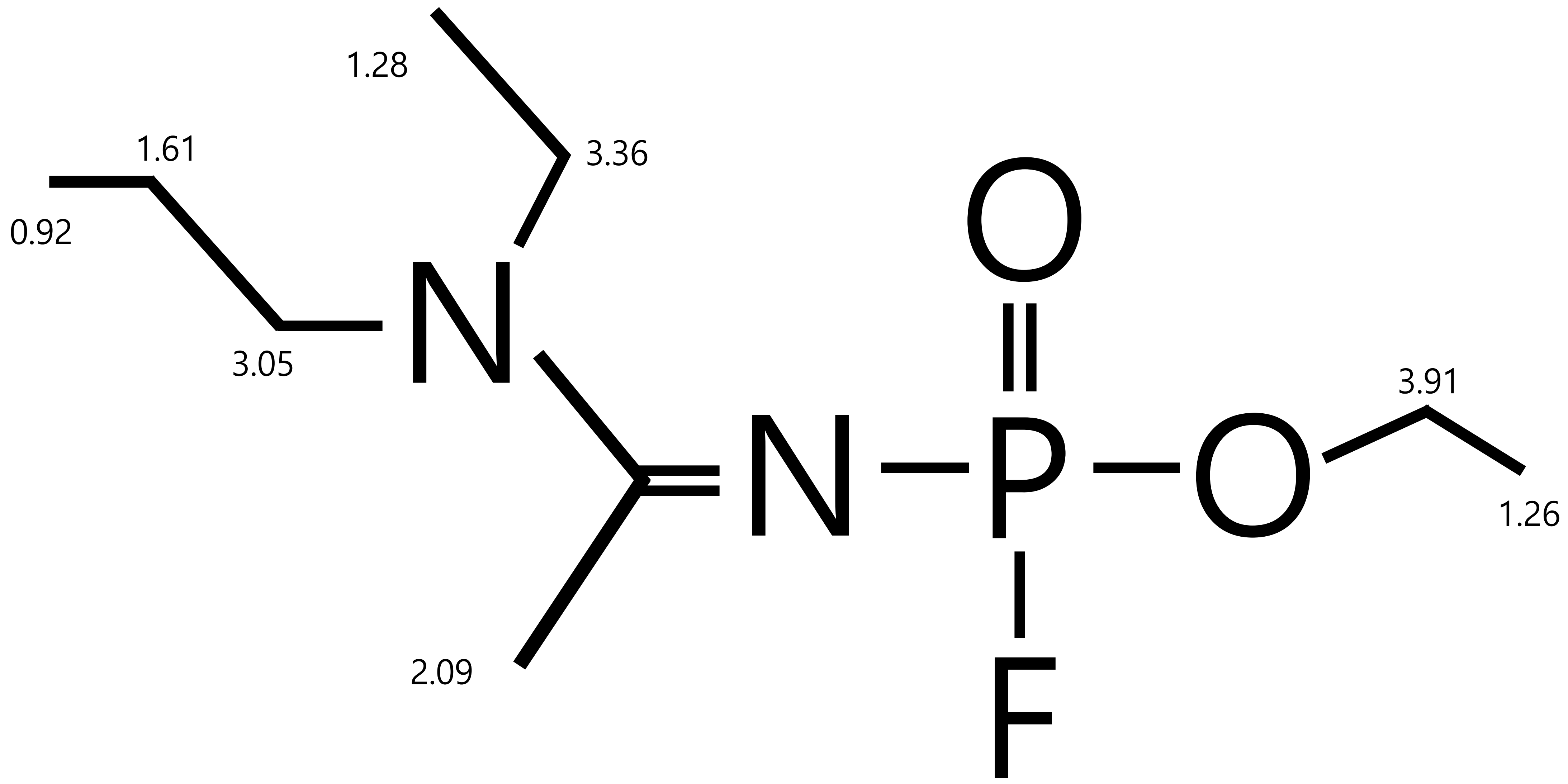

Figure S118. Structure 2132 and its ¹H chemical shift

2133 H

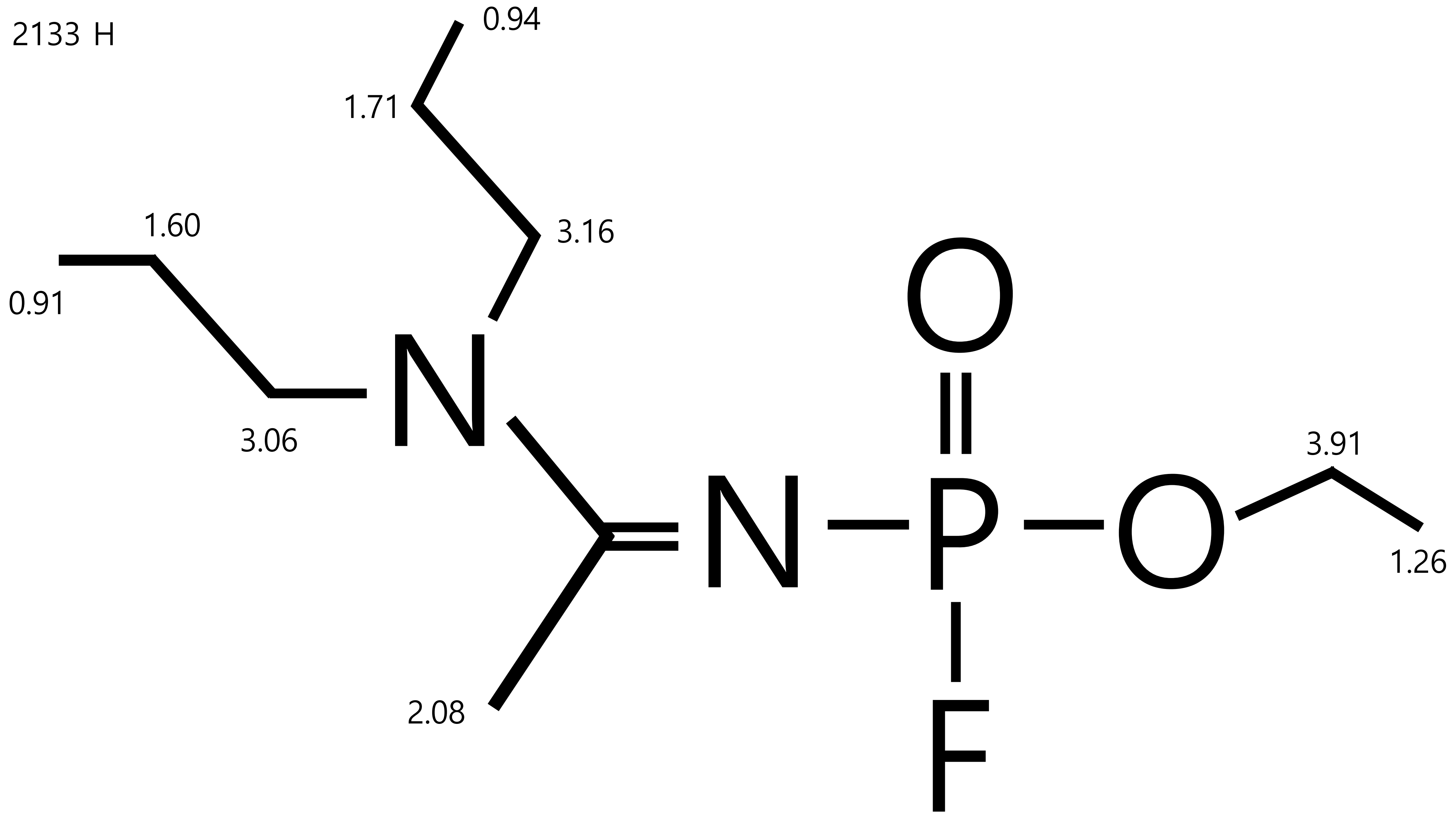

Figure S119. Structure 2133 and its ^{1}H chemical shift

2211 H

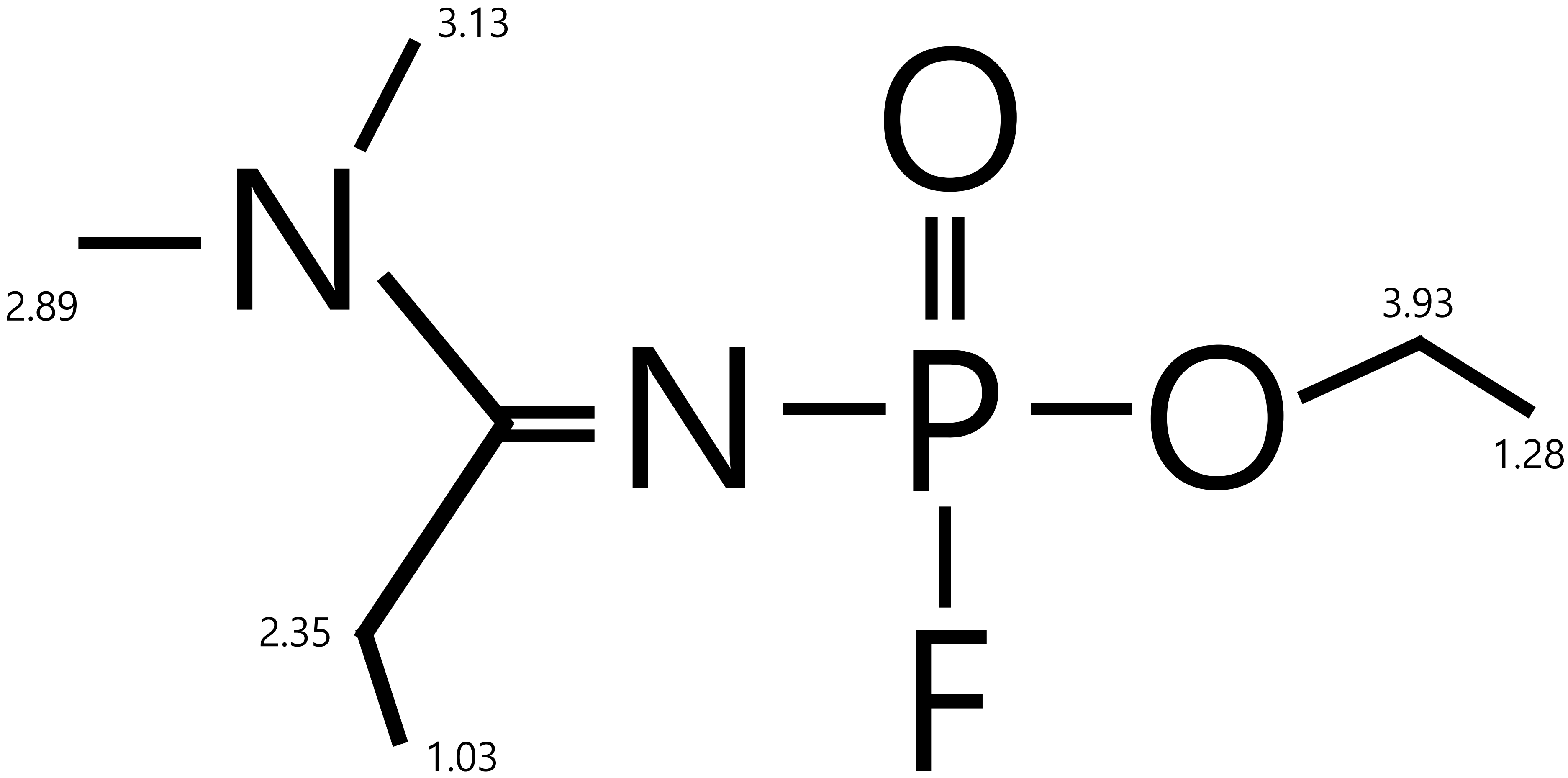

Figure S120. Structure 2211 and its ^{1}H chemical shift

2212 H

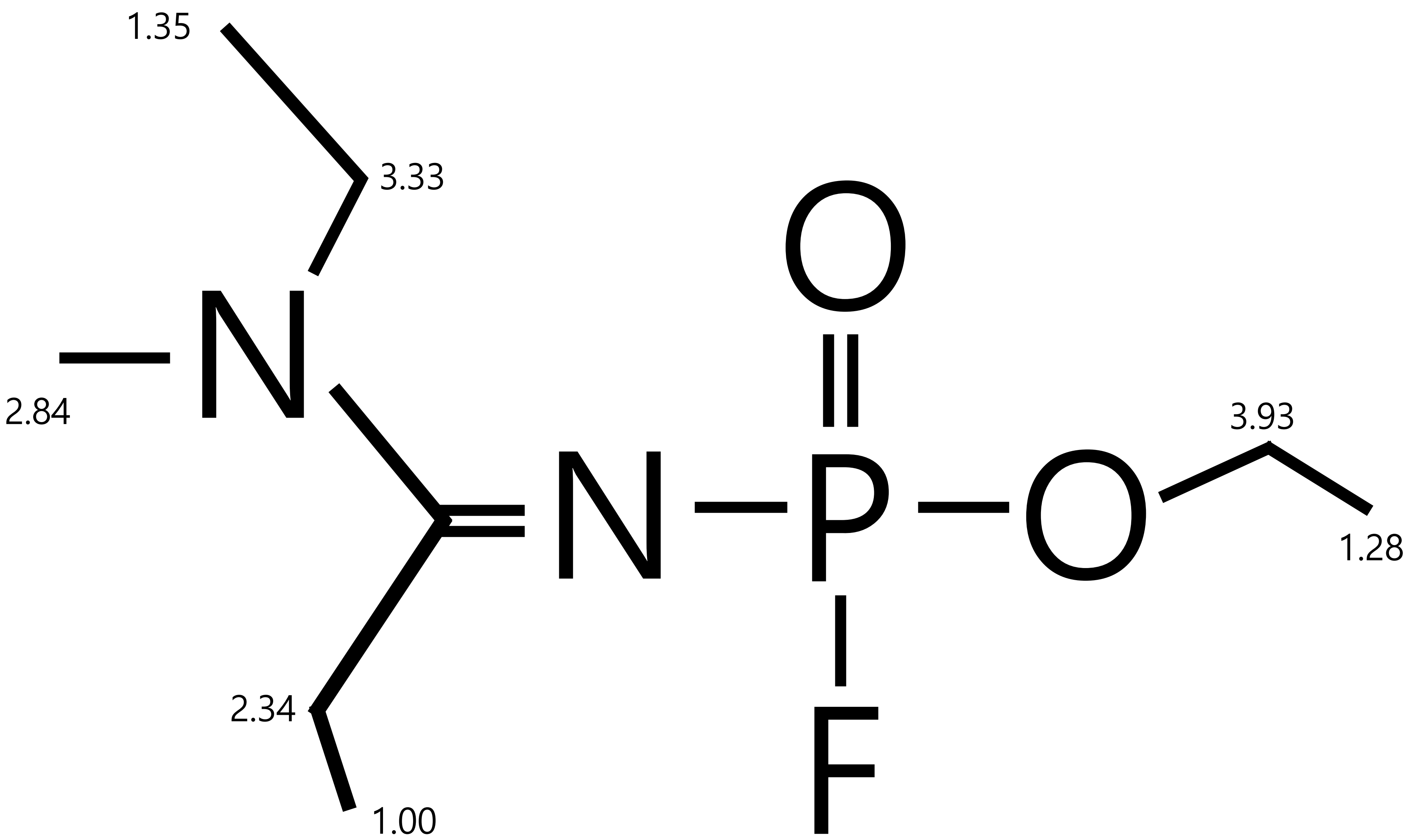

Figure S121. Structure 2212 and its ¹H chemical shift

2213 H

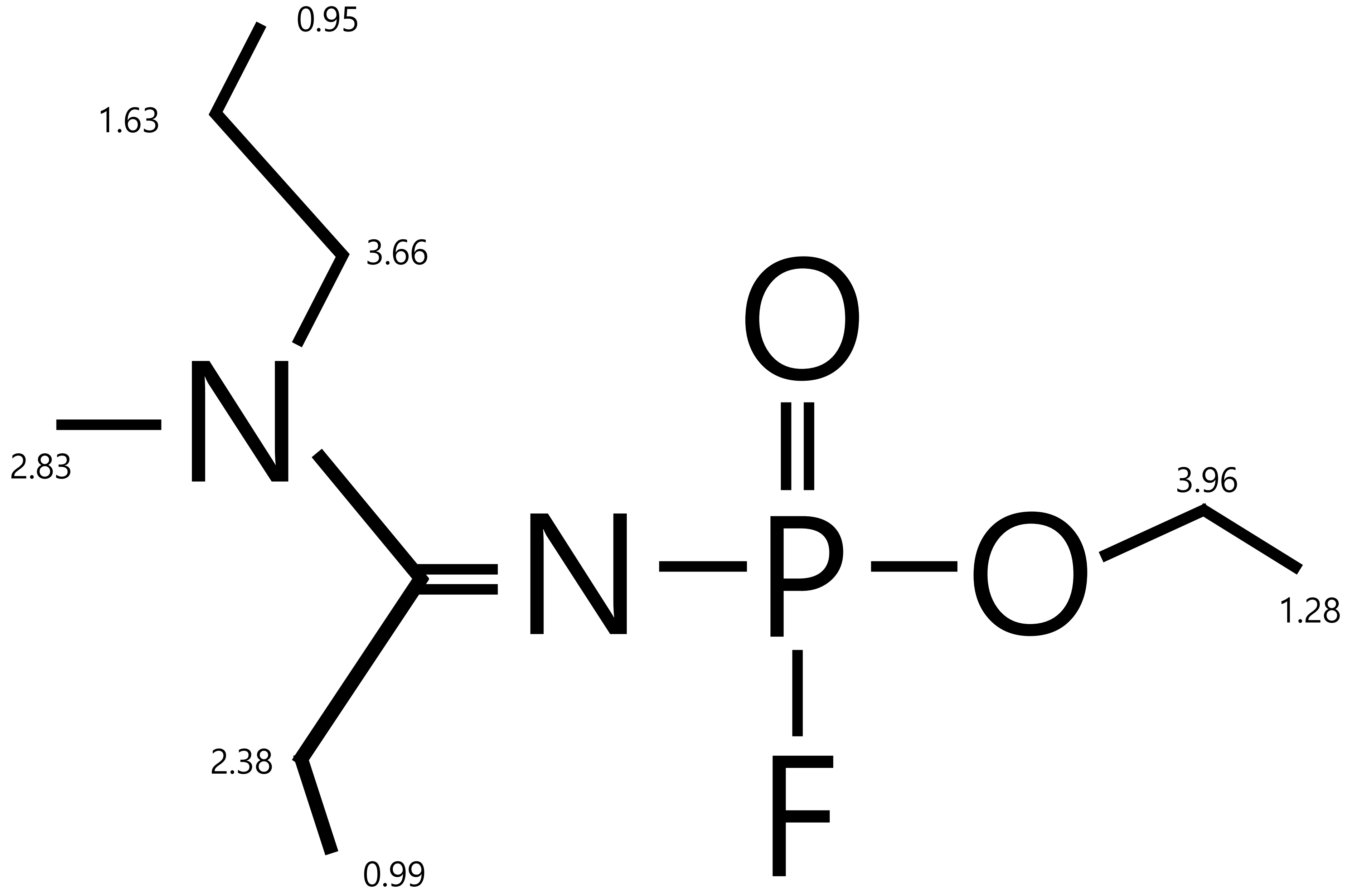

Figure S122. Structure 2213 and its ^{1}H chemical shift

2221 H

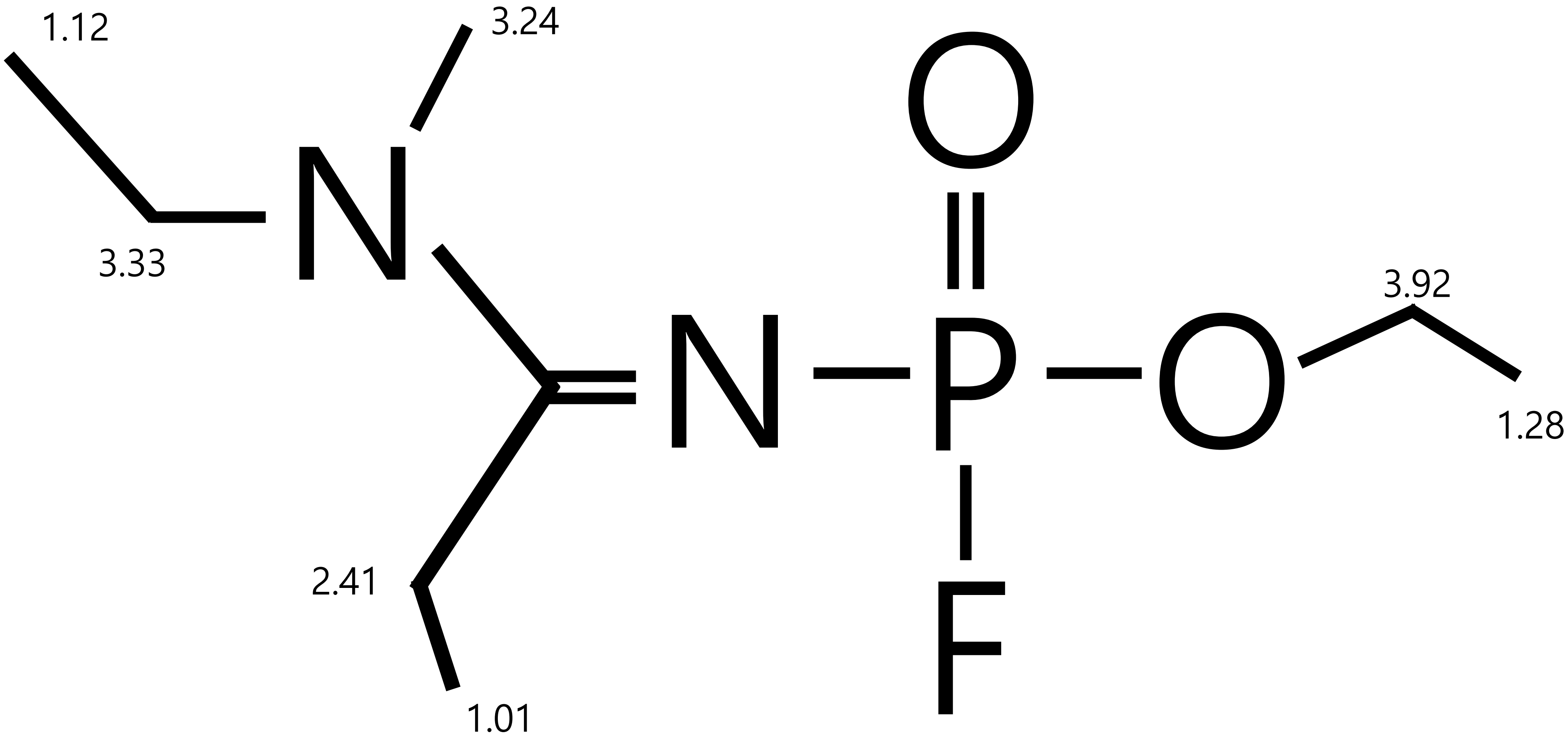

Figure S123. Structure 2221 and its ¹H chemical shift

2222 H

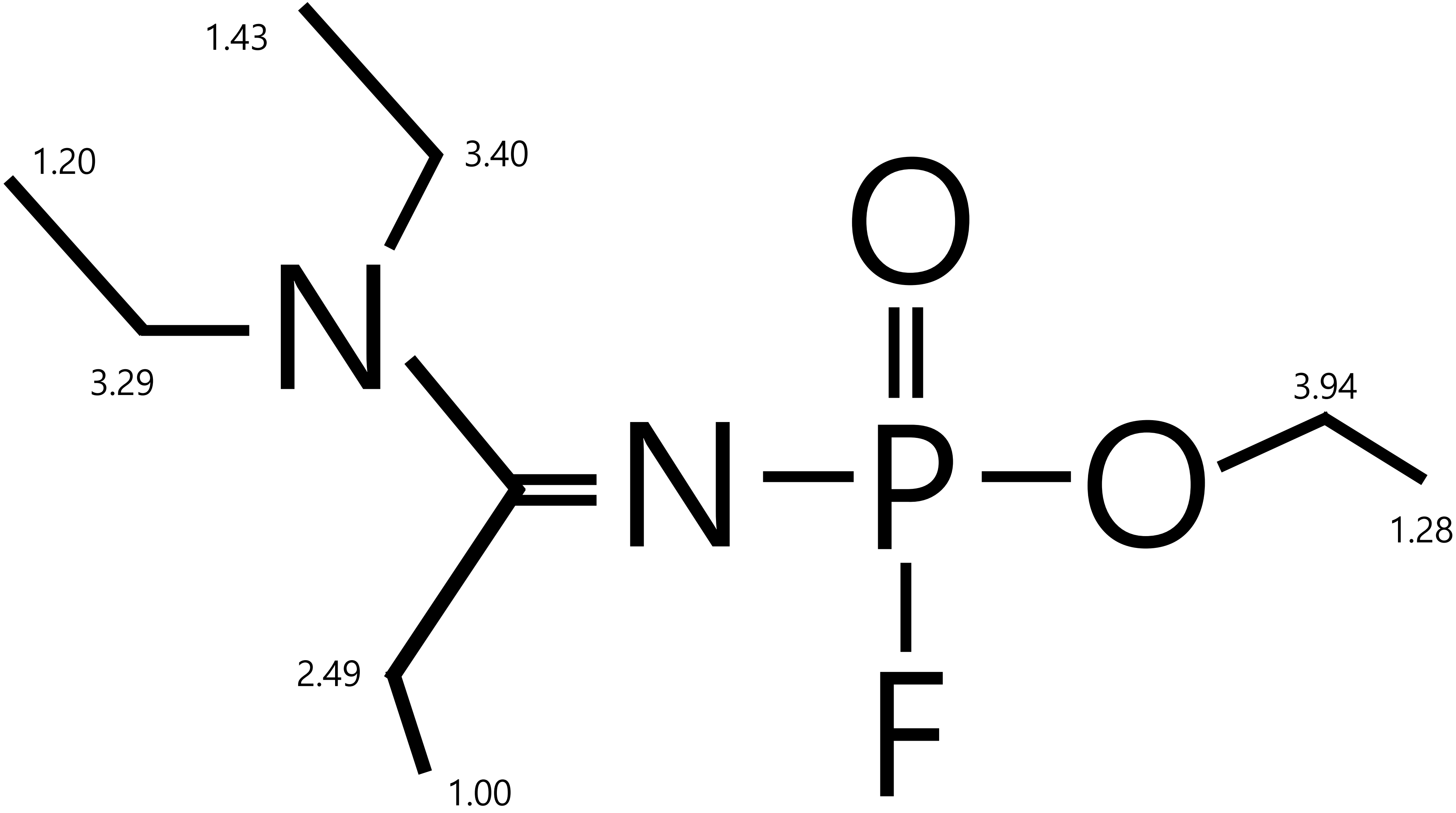

Figure S124. Structure 2222 and its ¹H chemical shift

2223 H

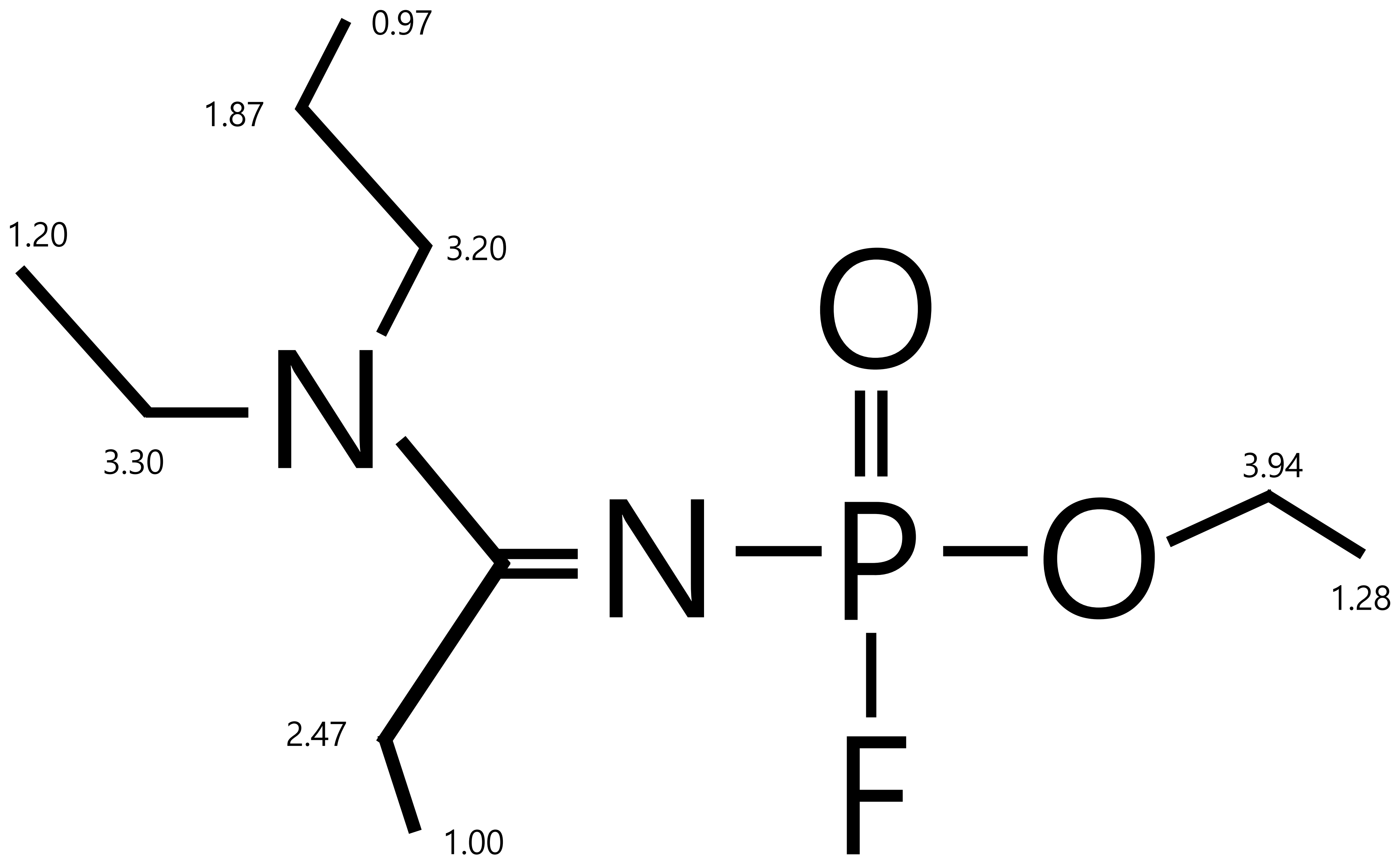

Figure S125. Structure 2223 and its ^{1}H chemical shift

2231 H

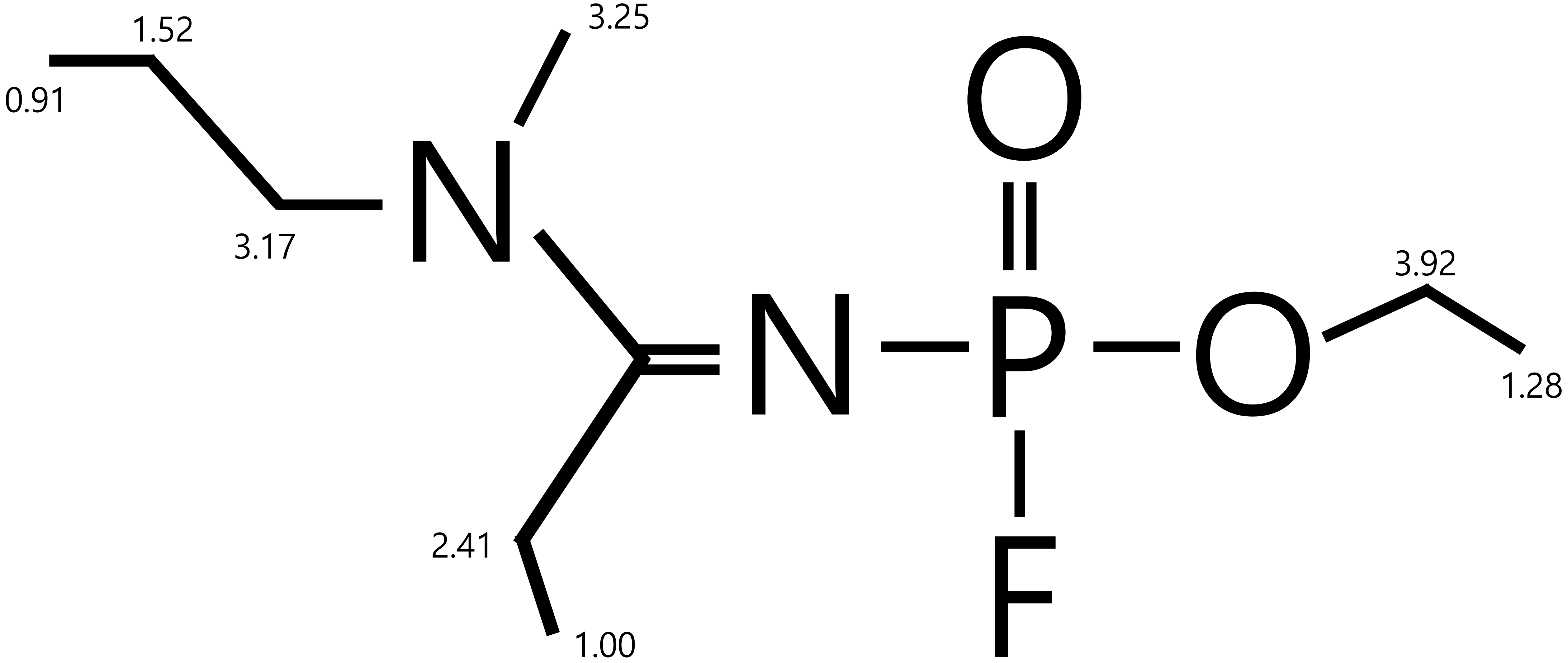

Figure S126. Structure 2231 and its ^{1}H chemical shift

2232 H

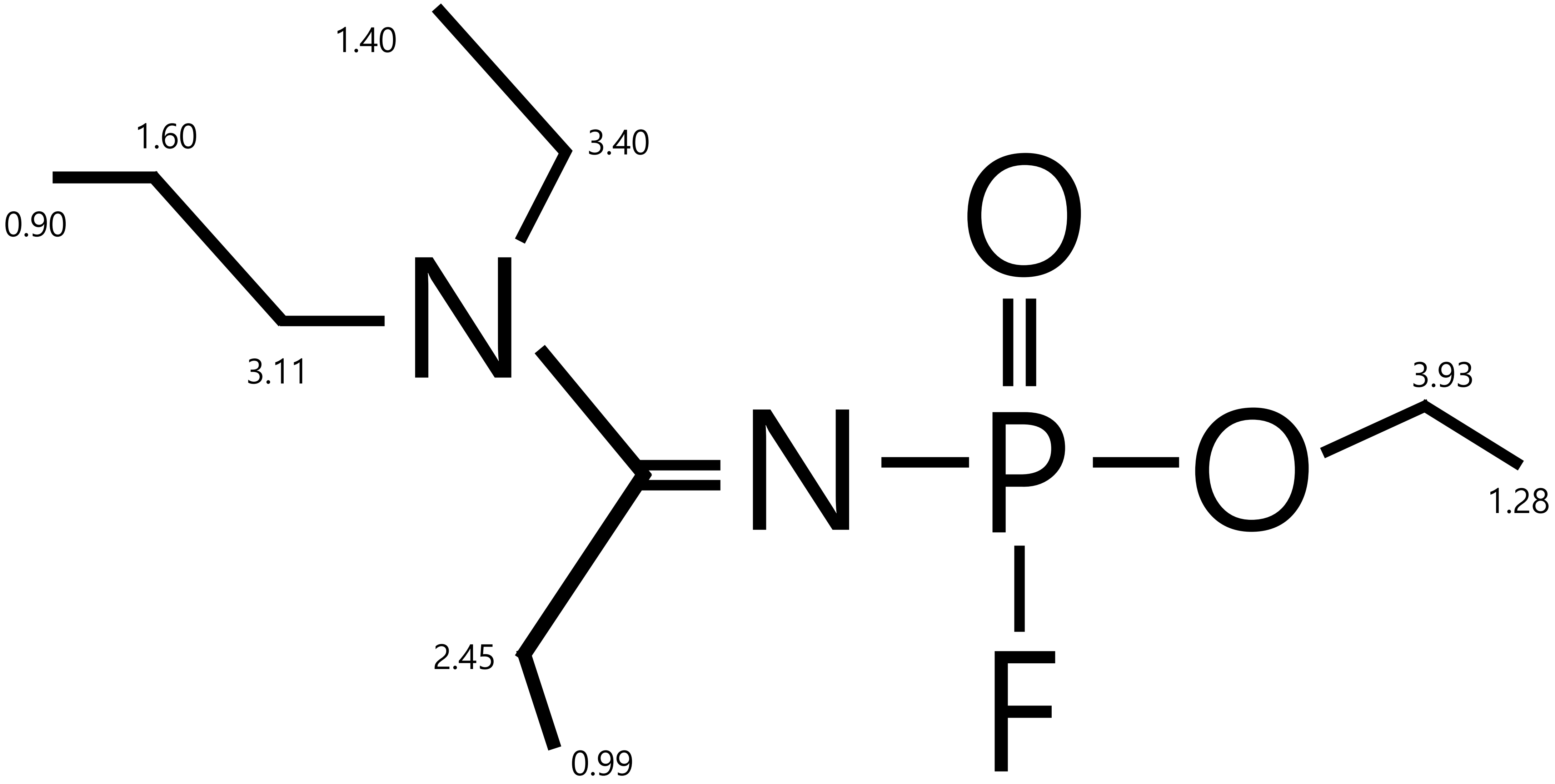

Figure S127. Structure 2232 and its ^{1}H chemical shift

2233 H

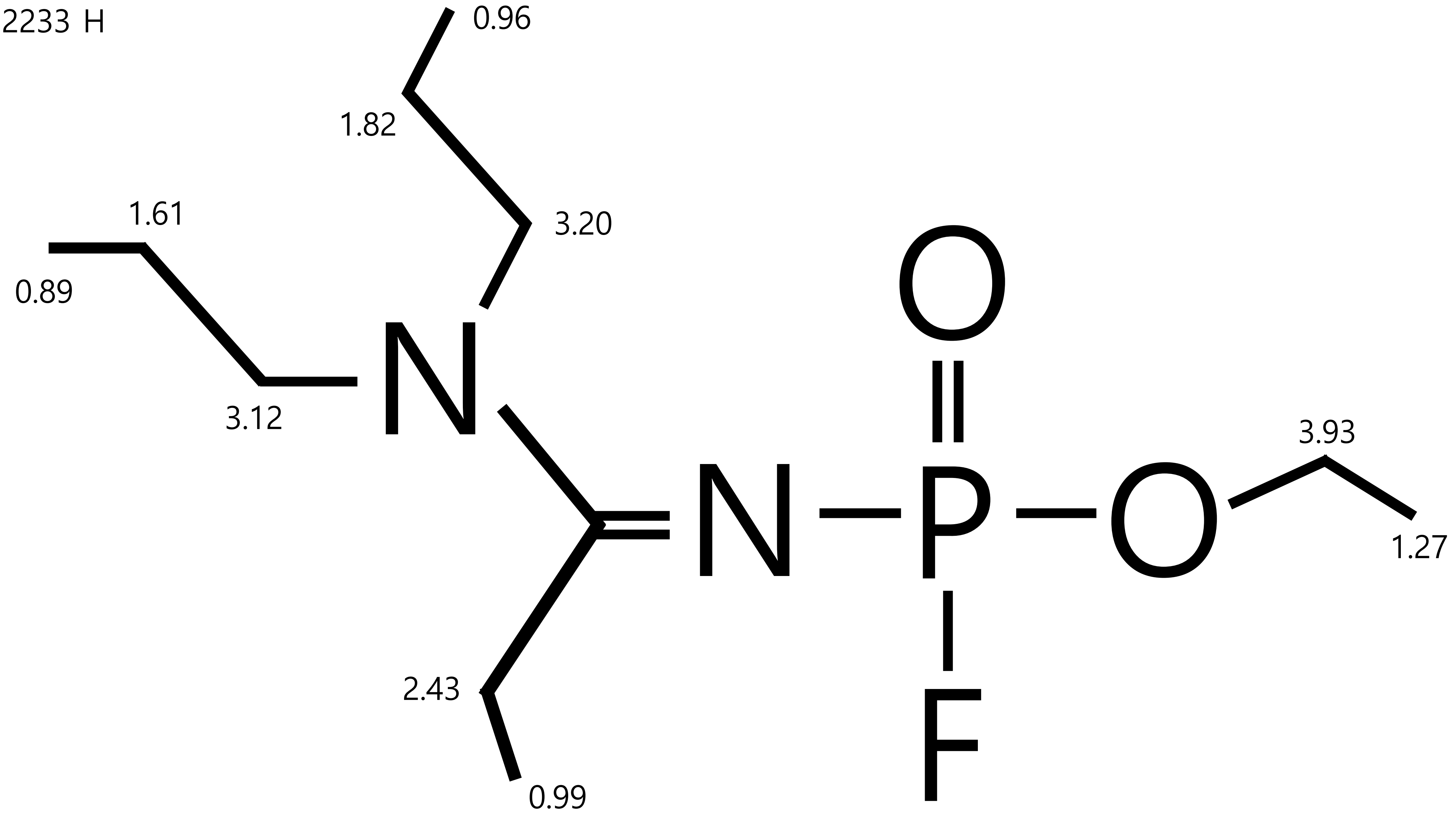

Figure S128. Structure 2233 and its ^{1}H chemical shift

2311 H

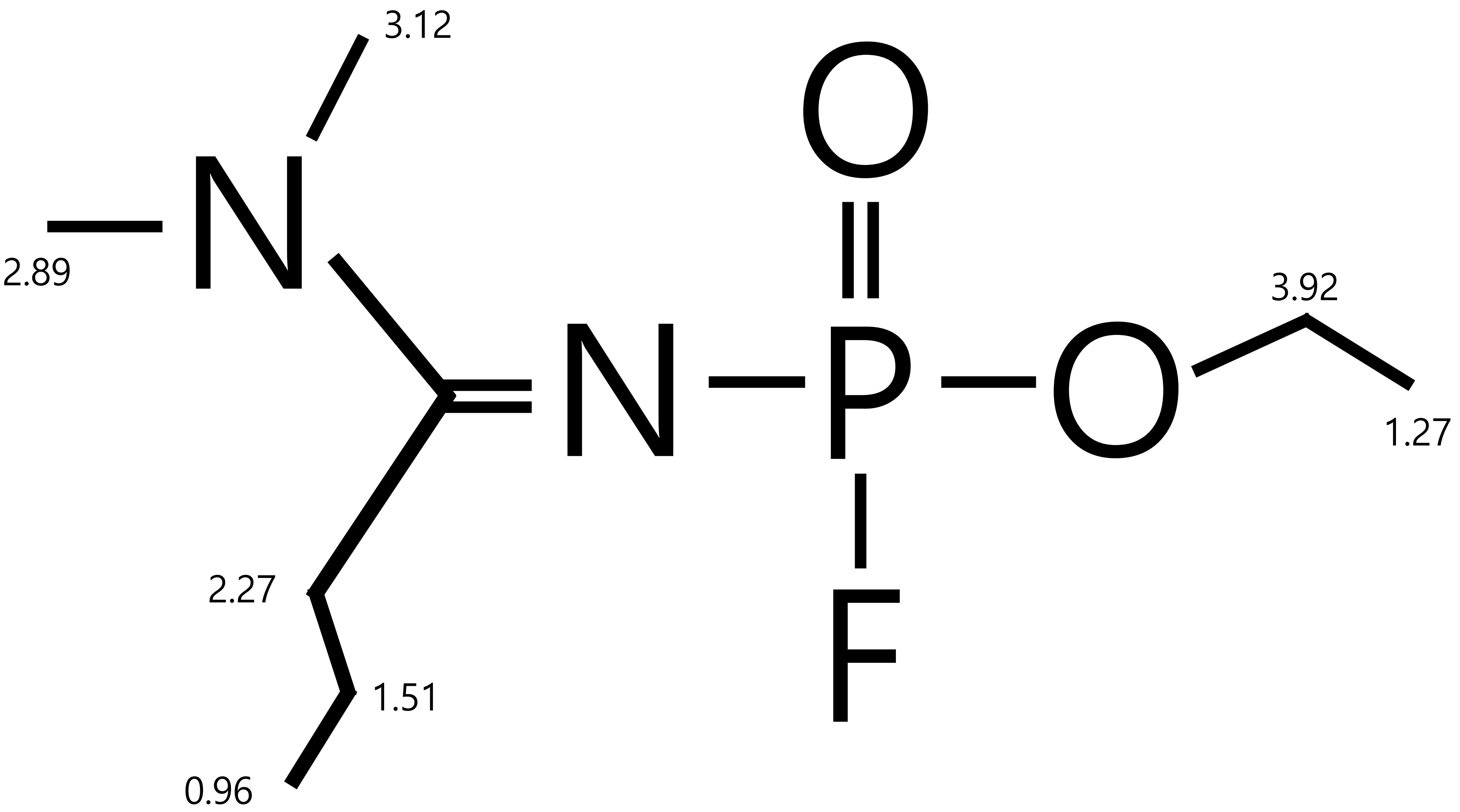

Figure S129. Structure 2311 and its ¹H chemical shift

2312 H

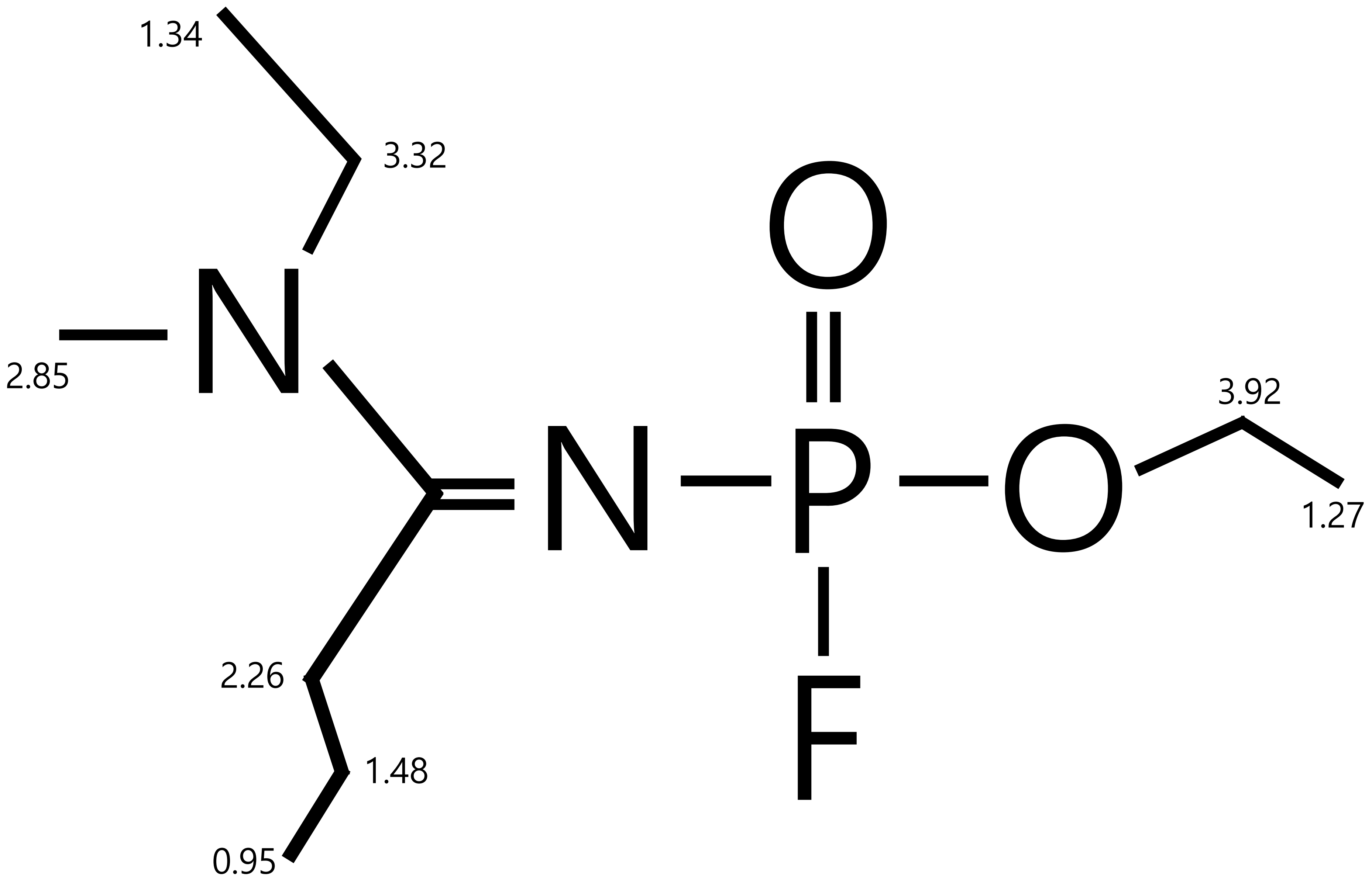

Figure S130. Structure 2312 and its ¹H chemical shift

2313 H

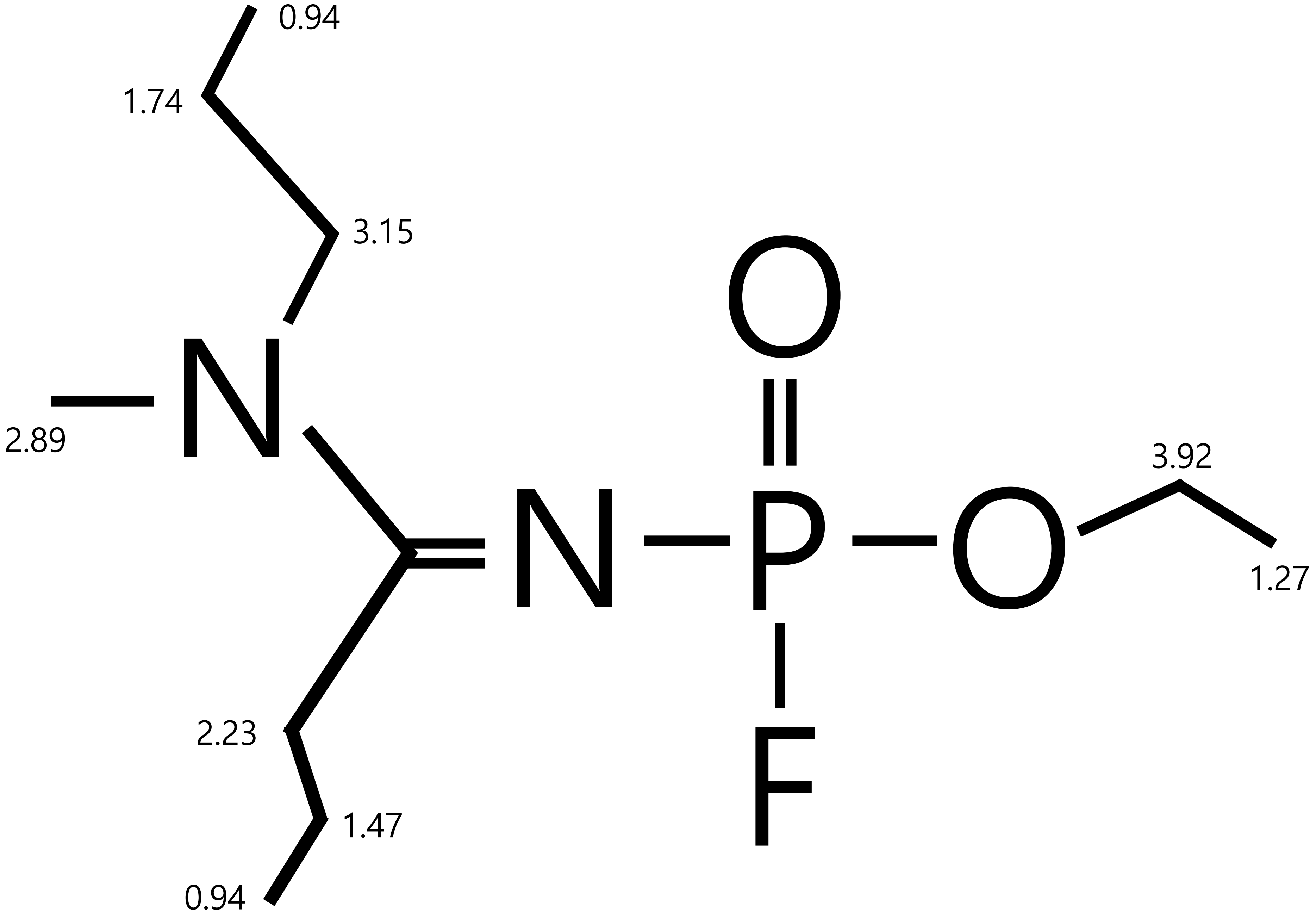

Figure S131. Structure 2313 and its ^{1}H chemical shift

2321 H

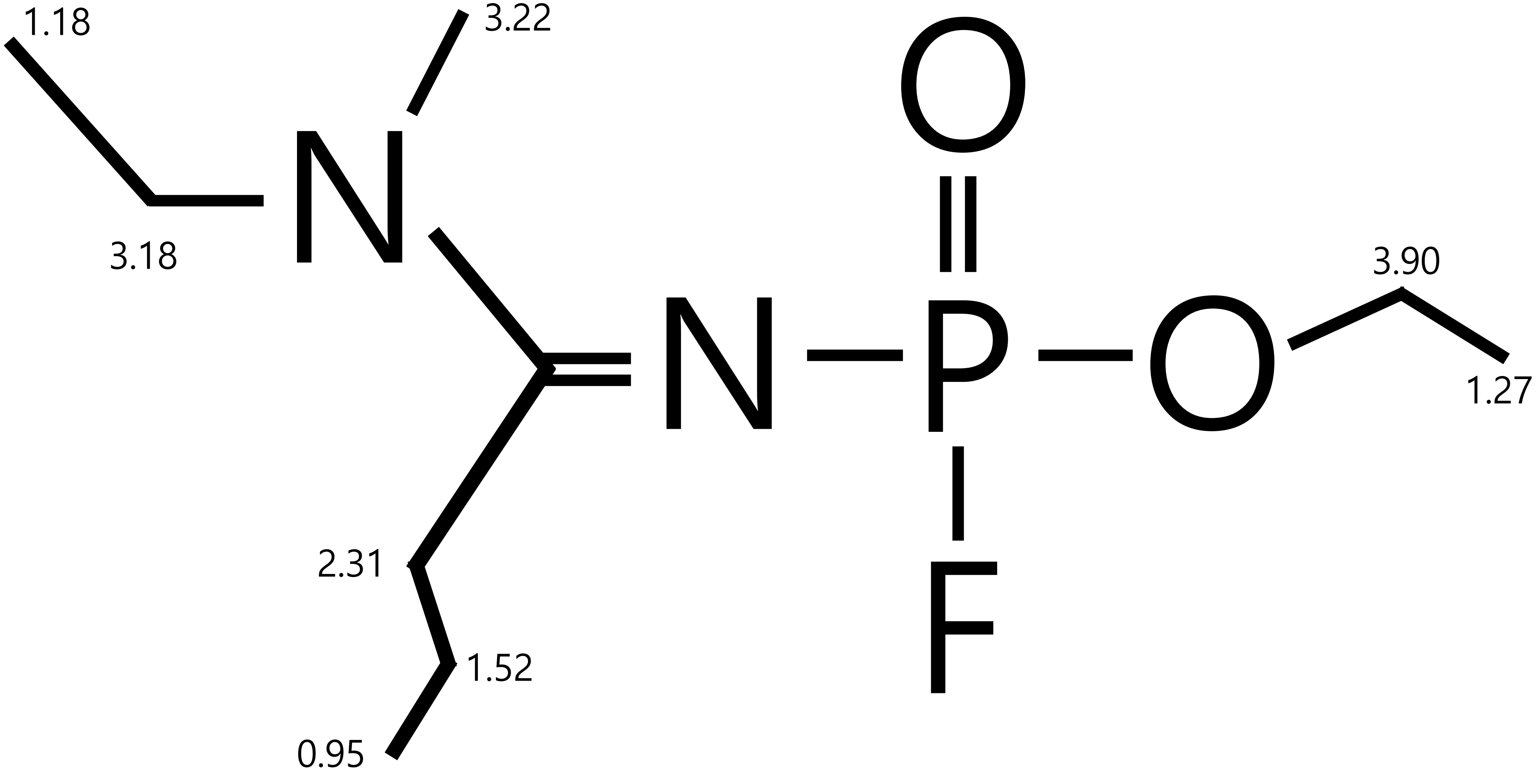

Figure S132. Structure 2321 and its ^{1}H chemical shift

2322 H

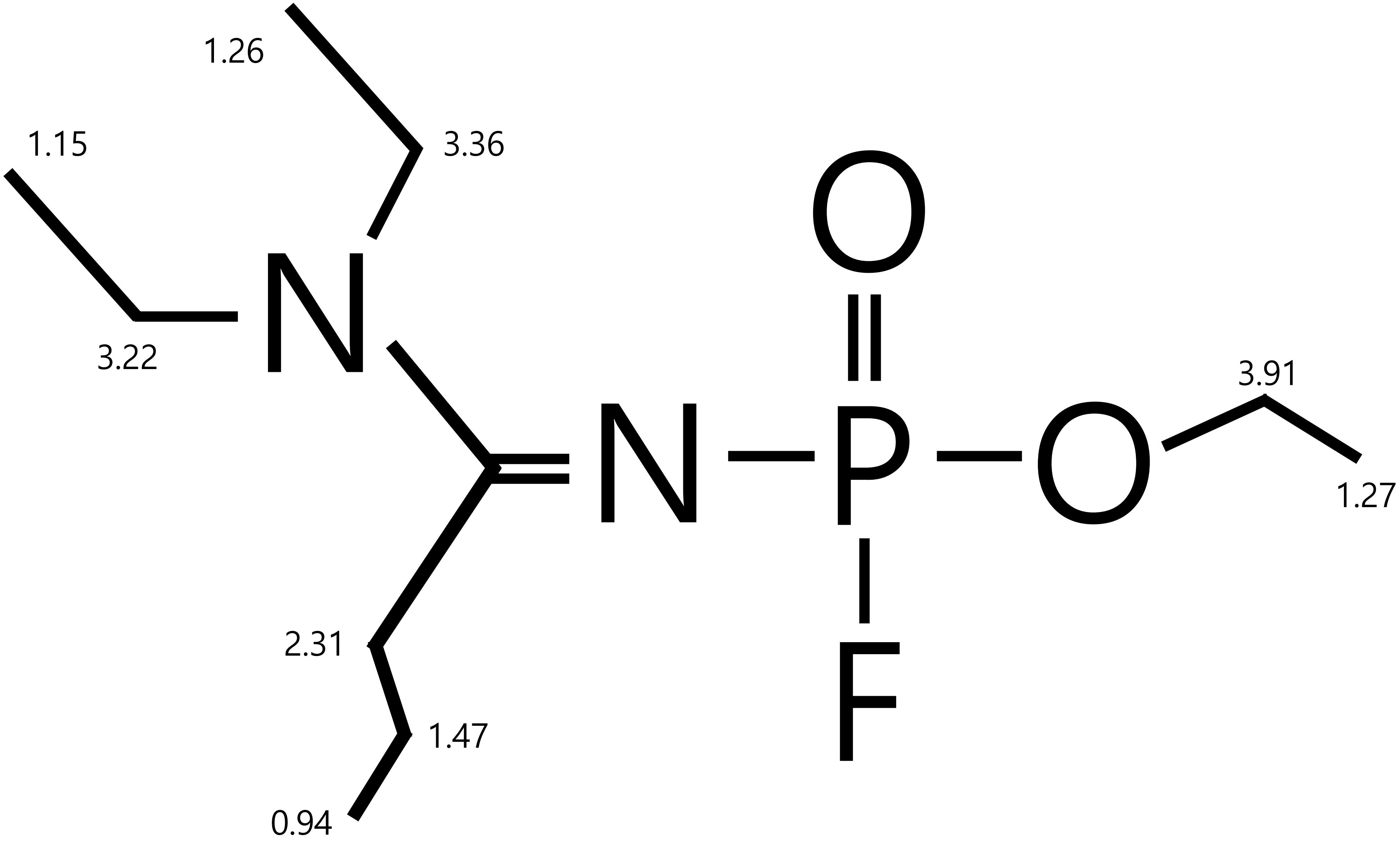

Figure S133. Structure 2322 and its ^{1}H chemical shift

2323 H

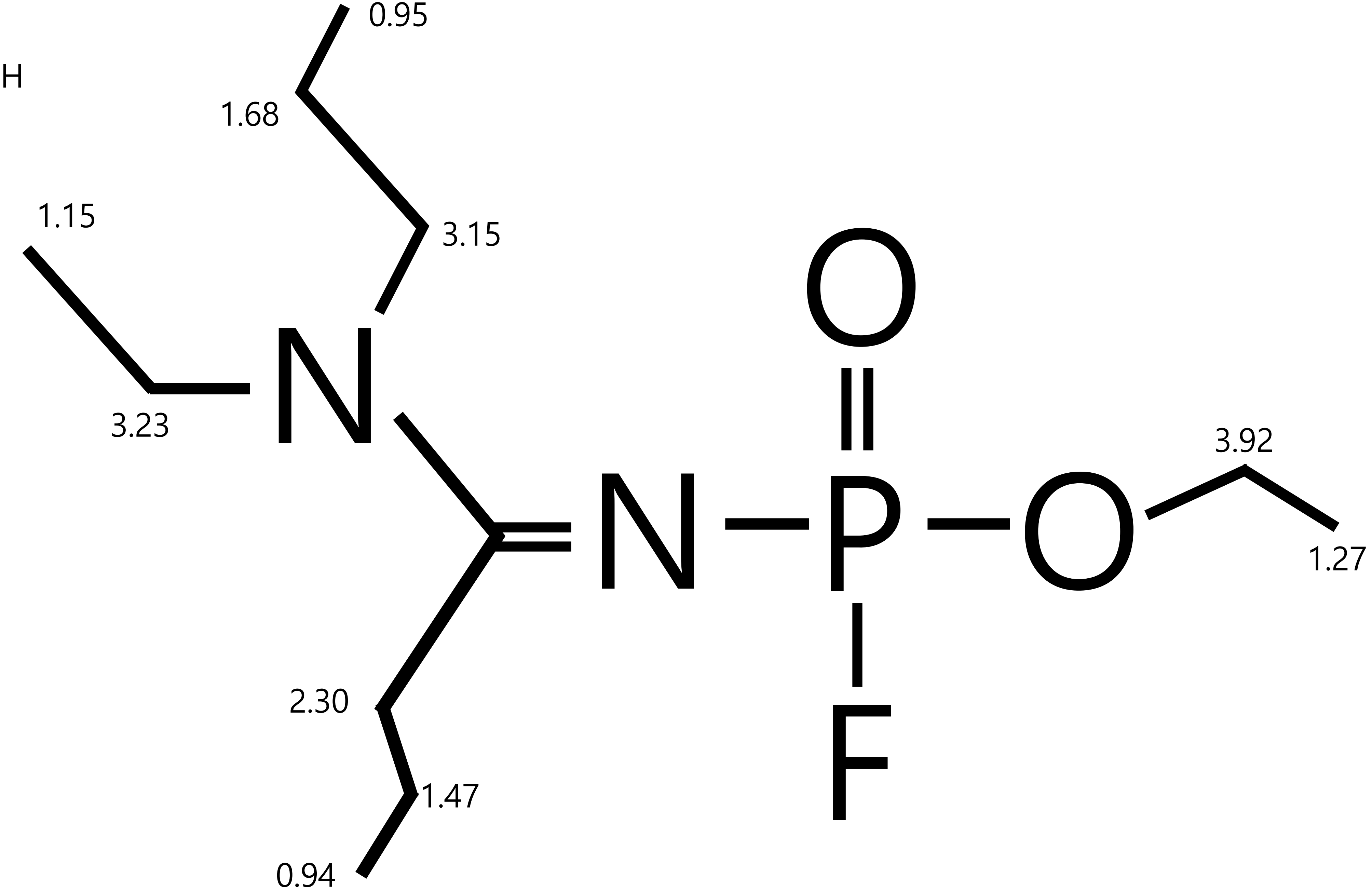

Figure S134. Structure 2323 and its ^{1}H chemical shift

2331 H

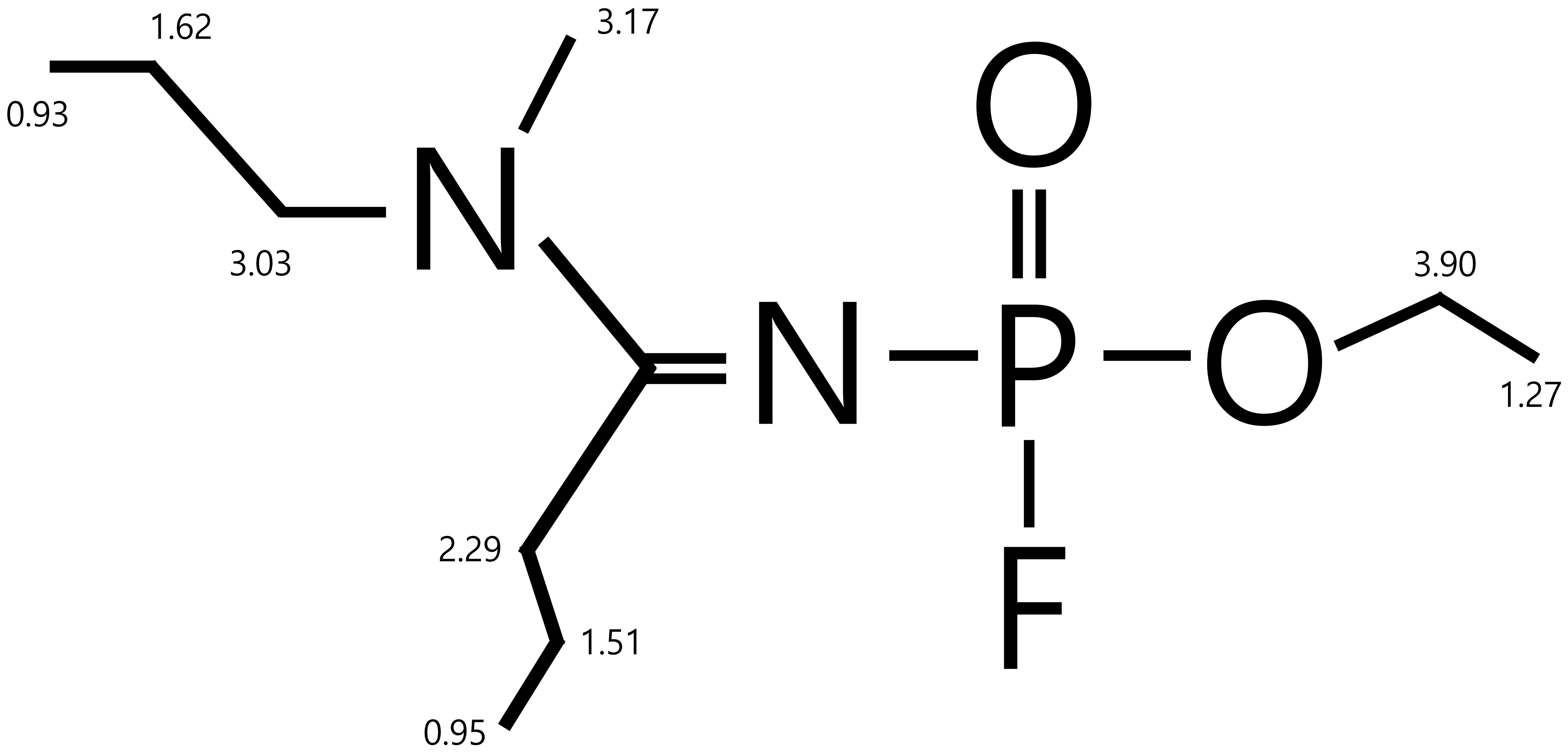

Figure S135. Structure 2331 and its ¹H chemical shift

2332 H

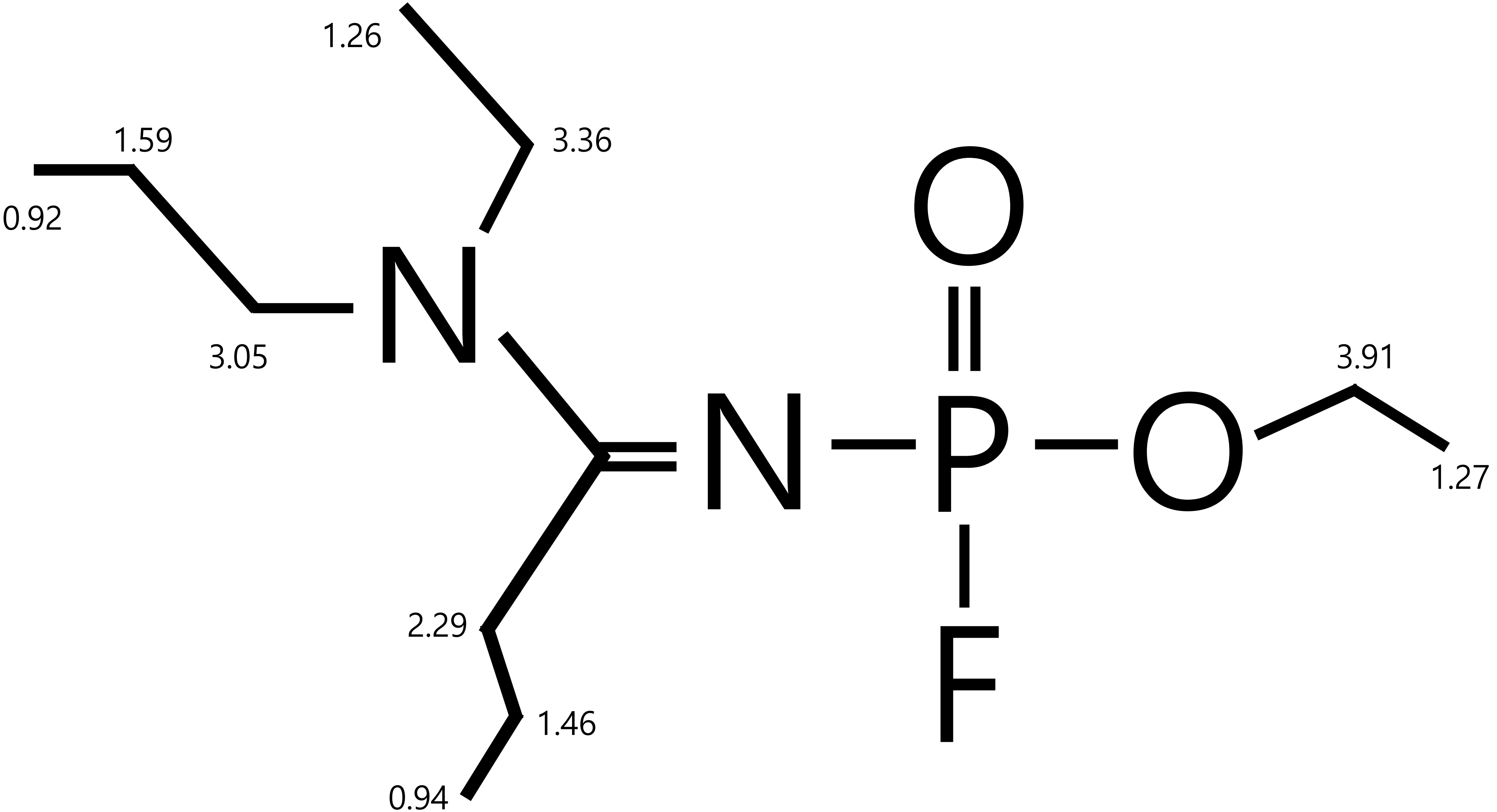

Figure S136. Structure 2332 and its ¹H chemical shift

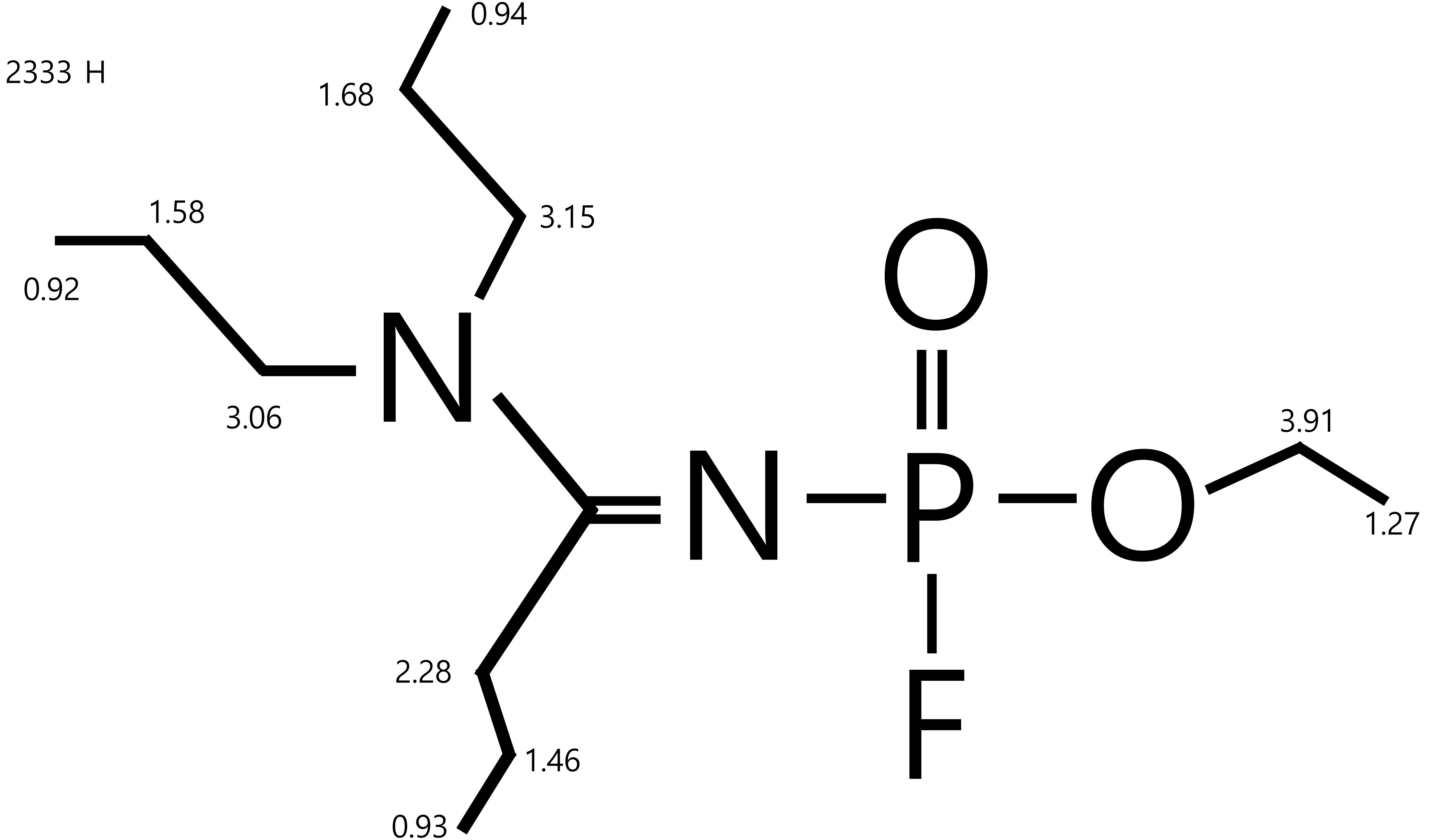

Figure S137. Structure 2333 and its ¹H chemical shift

3111 H

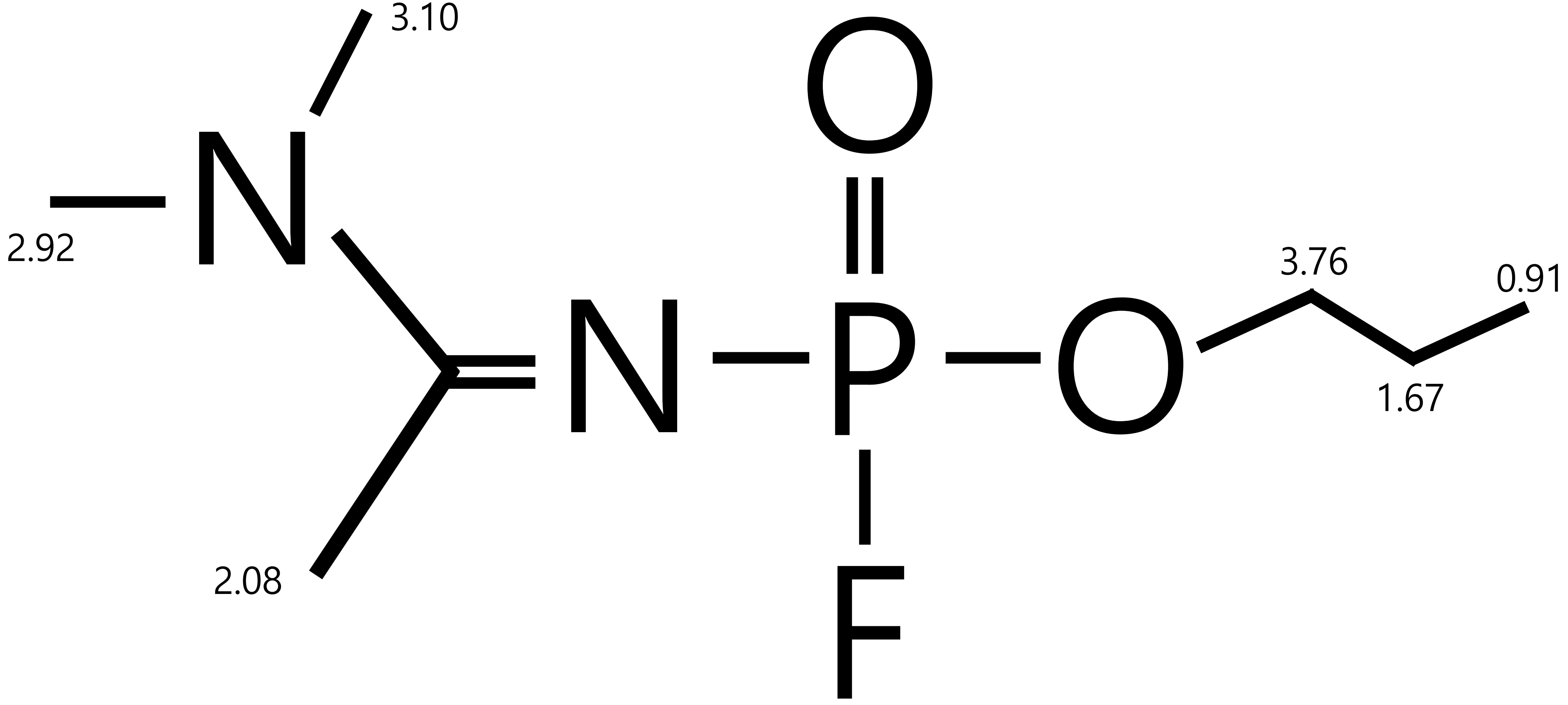

Figure S138. Structure 3111 and its ^{1}H chemical shift

3112 H

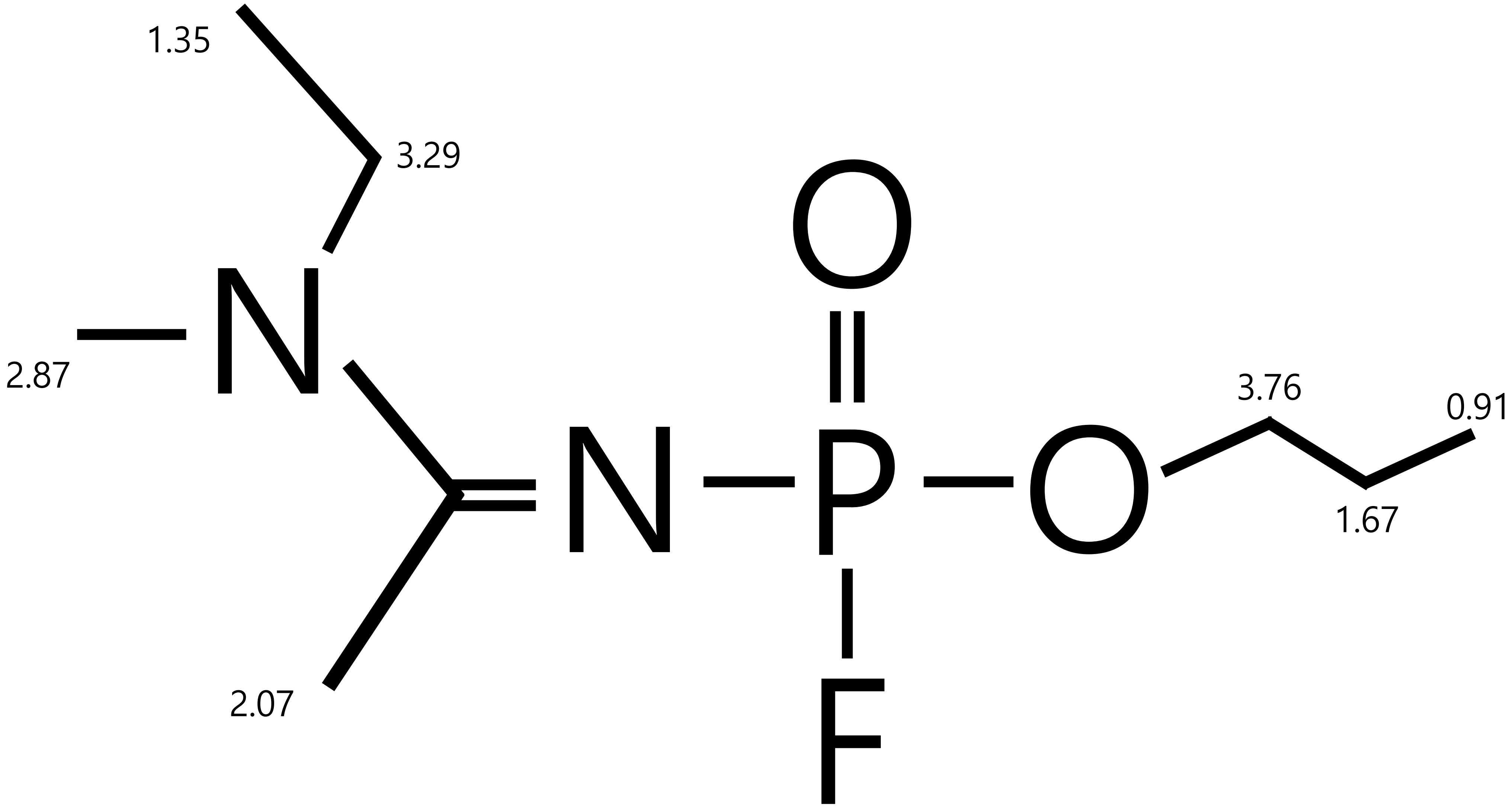

Figure S139. Structure 3112 and its ^{1}H chemical shift

3113 H

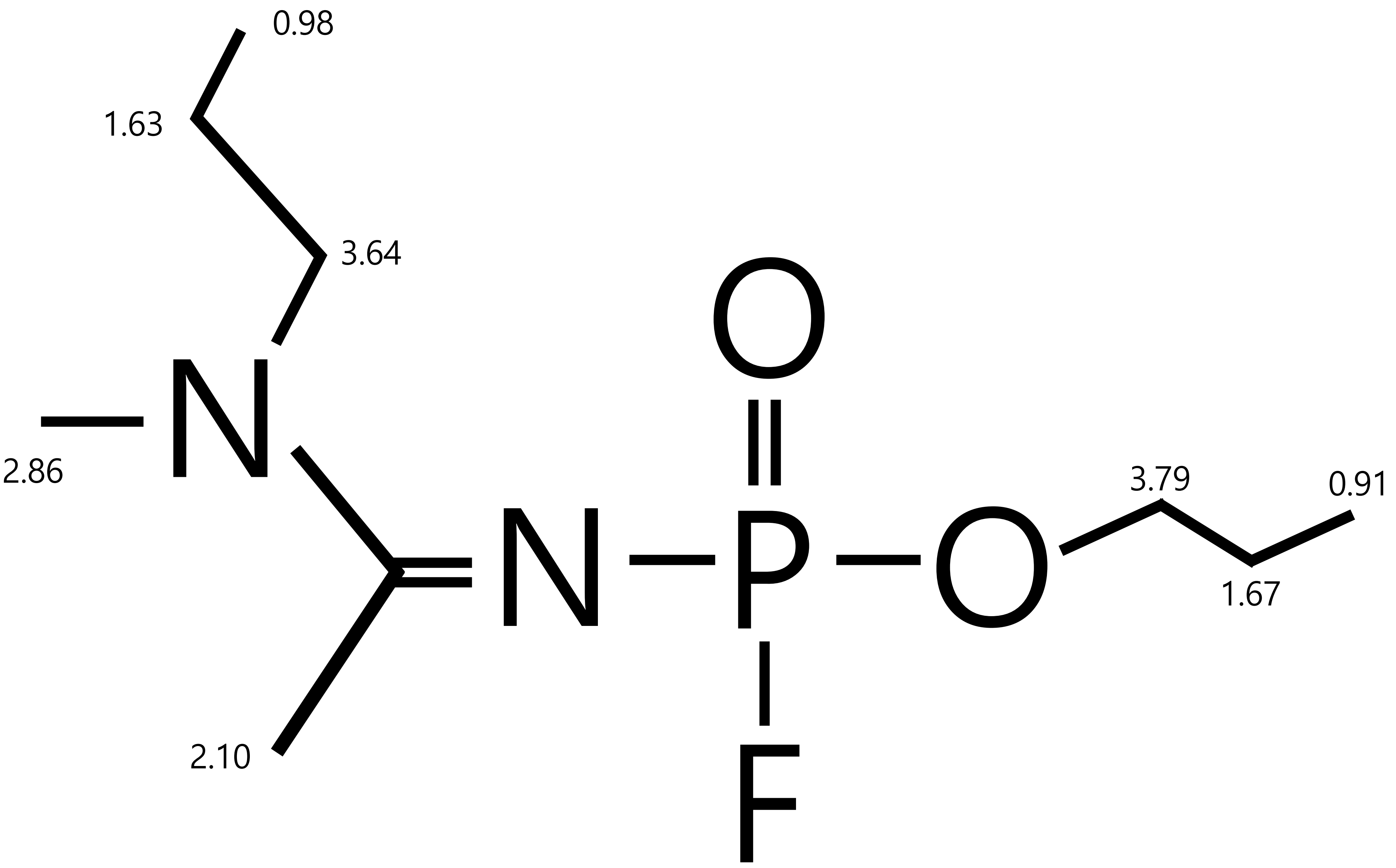

Figure S140. Structure 3113 and its ^{1}H chemical shift

3121 H

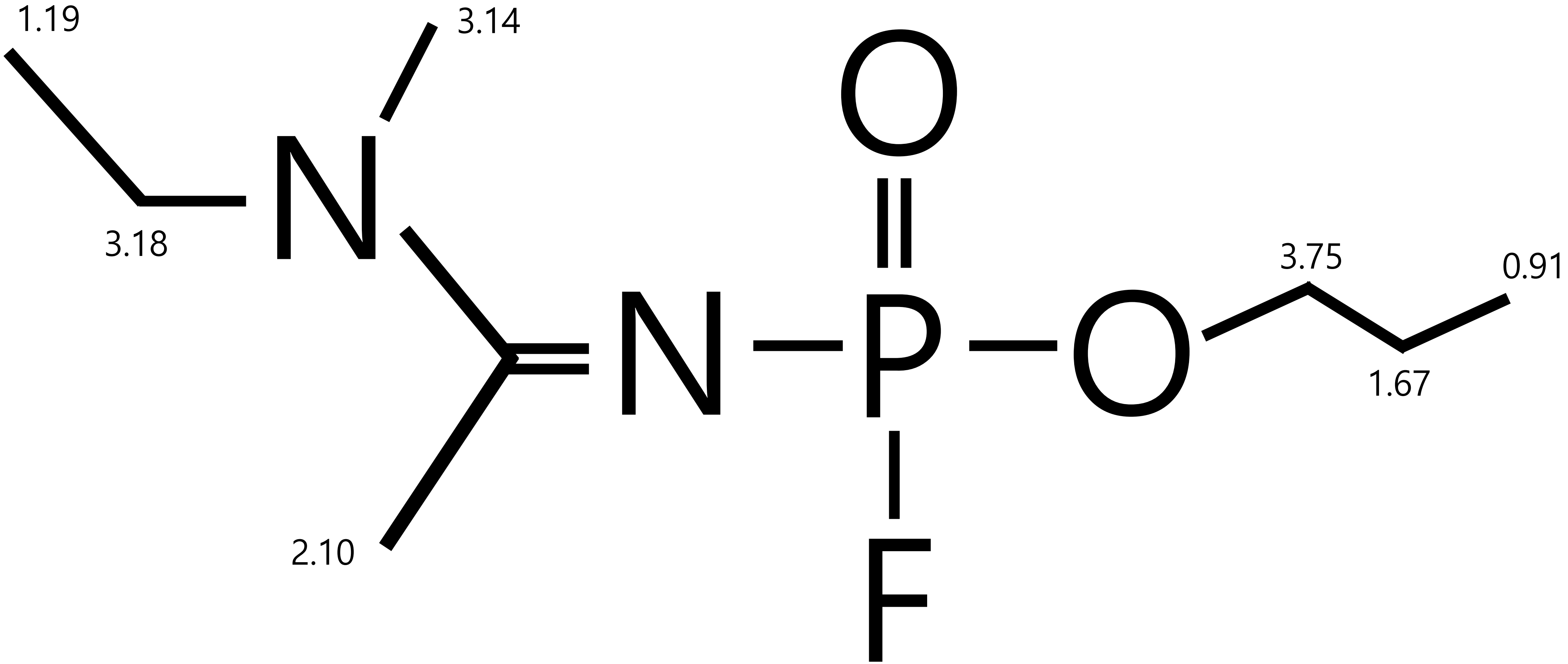

Figure S141. Structure 3121 and its ^{1}H chemical shift

3122 H

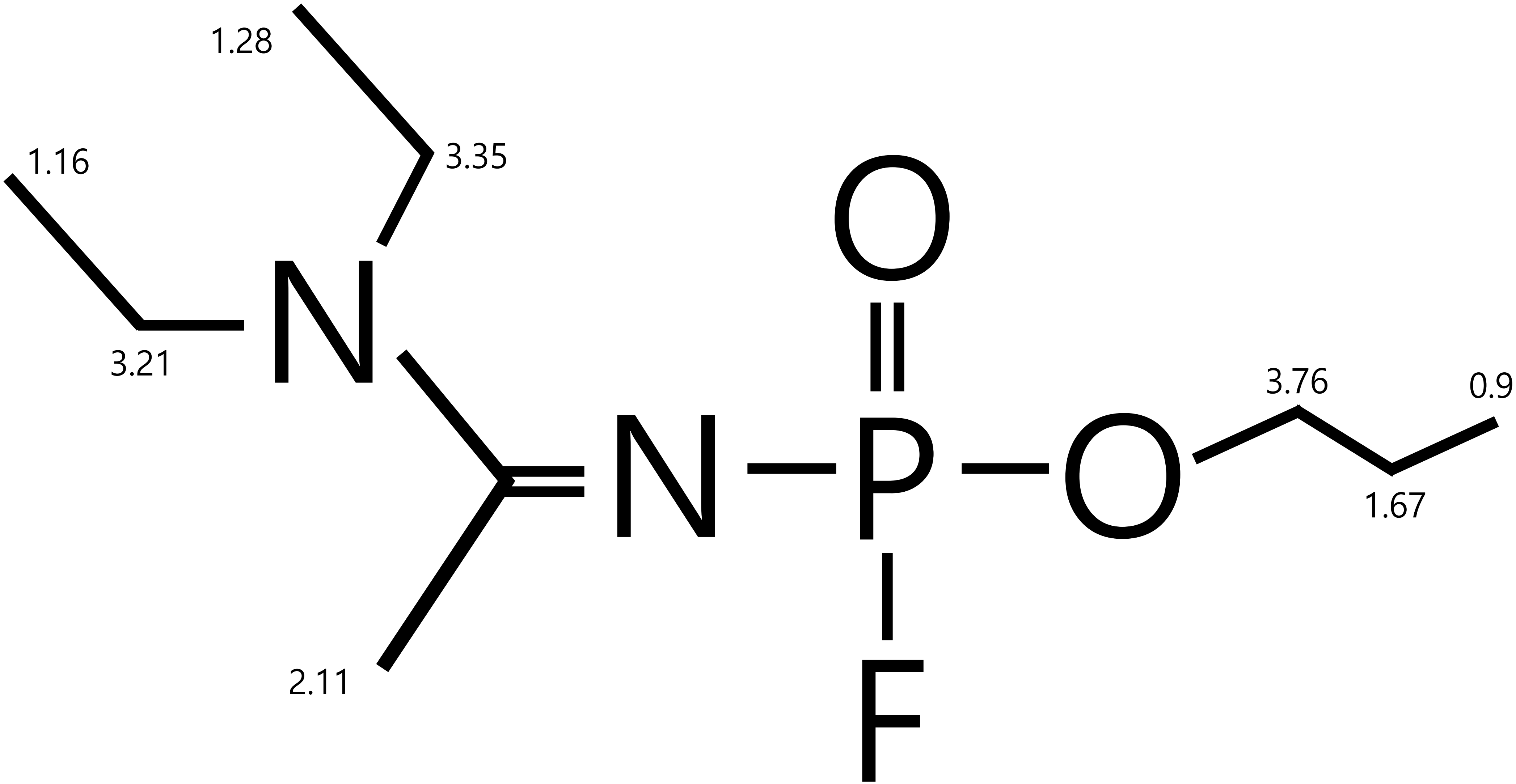

Figure S142. Structure 3122 and its ^1H chemical shift

3123 H

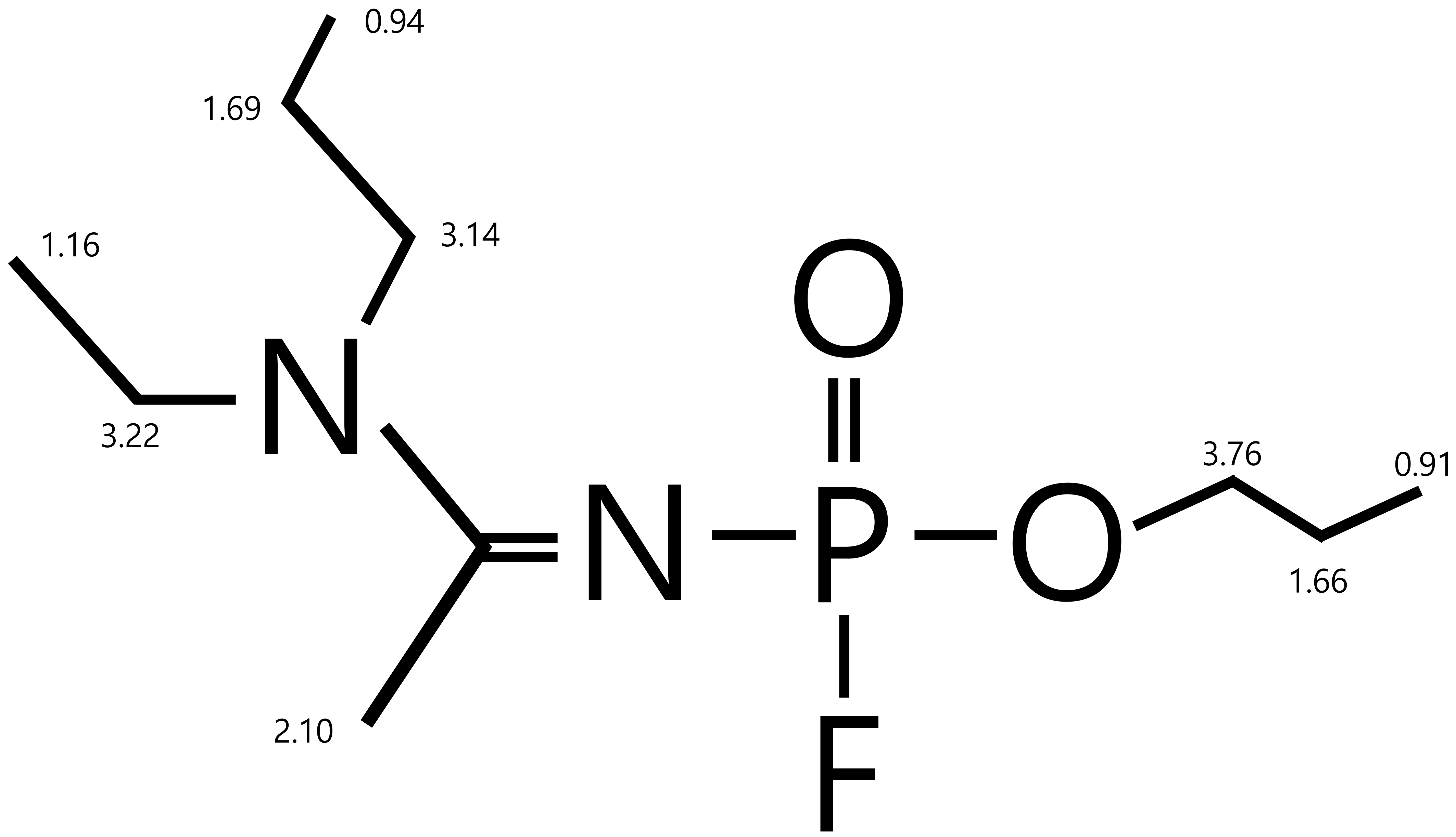

Figure S143. Structure 3123 and its ^{1}H chemical shift

3131 H

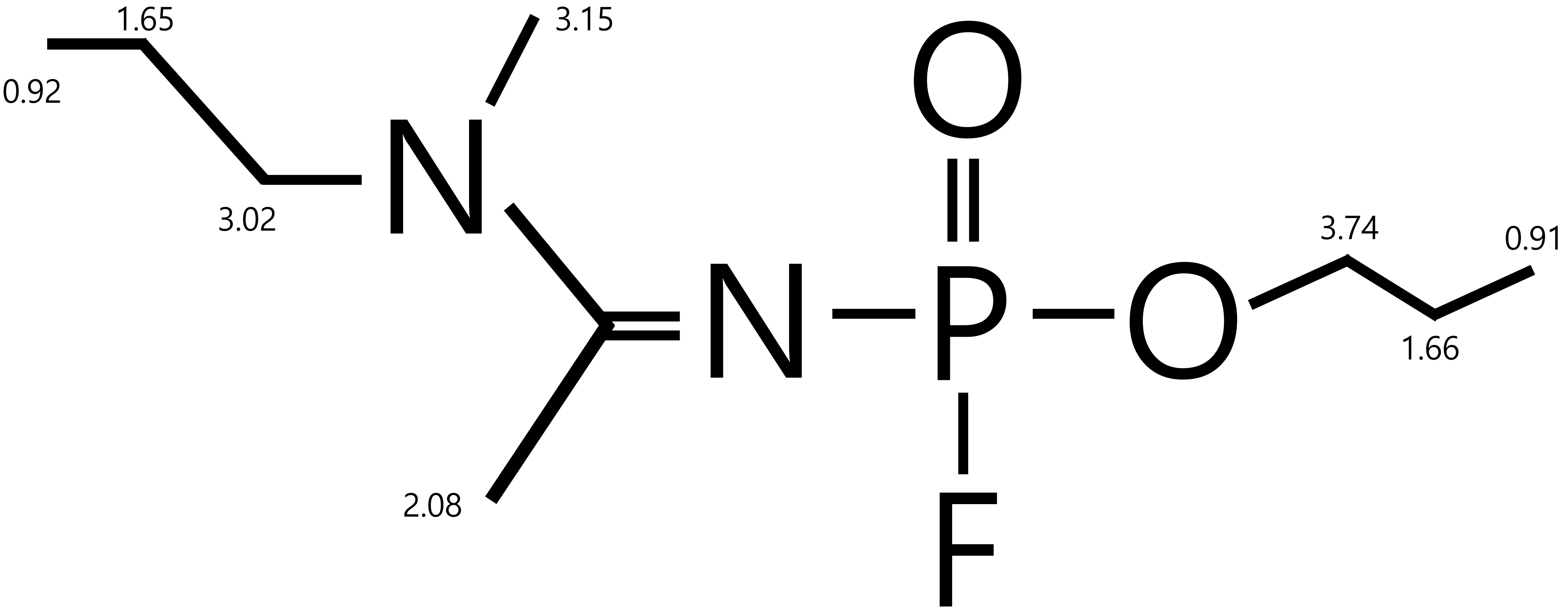

Figure S144. Structure 3131 and its ^{1}H chemical shift

3132 H

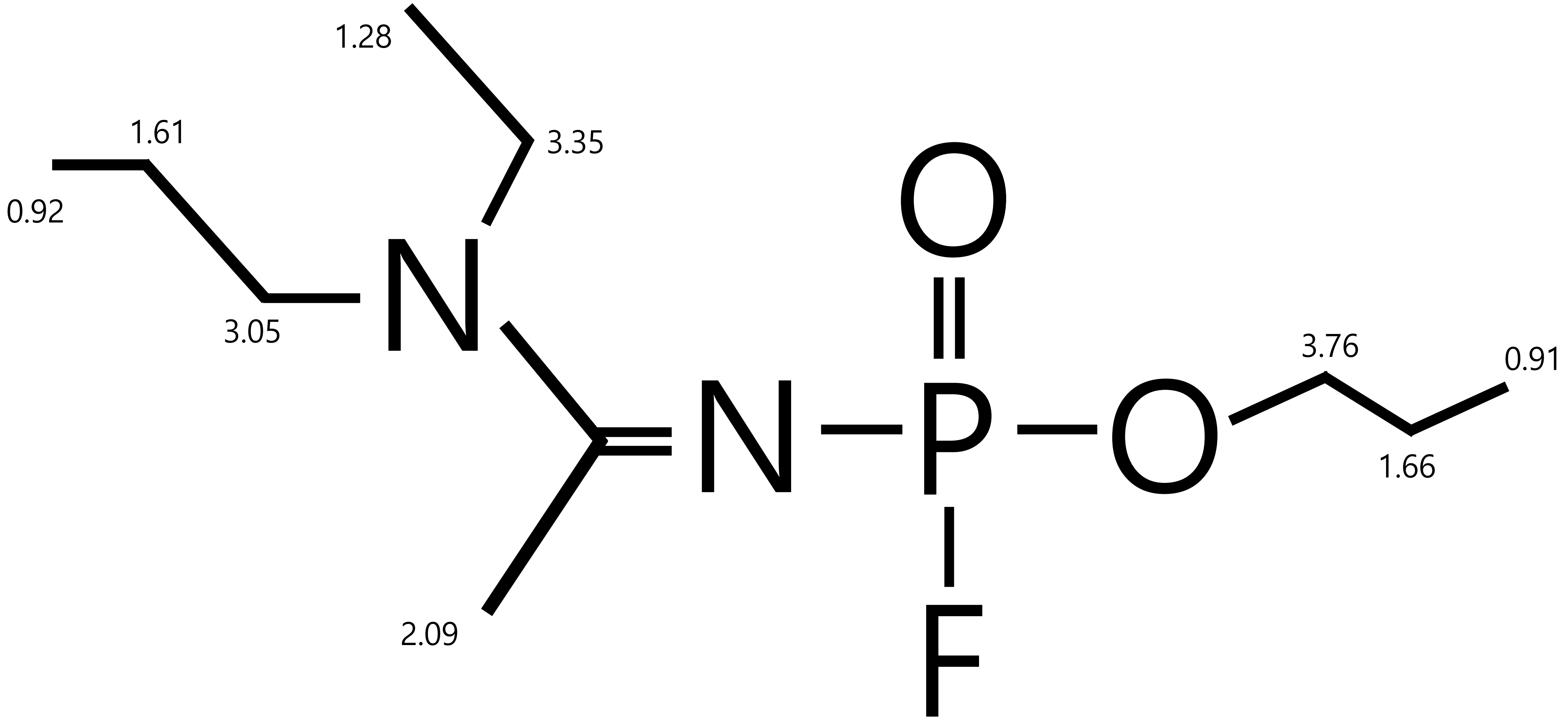

Figure S145. Structure 3132 and its ^{1}H chemical shift

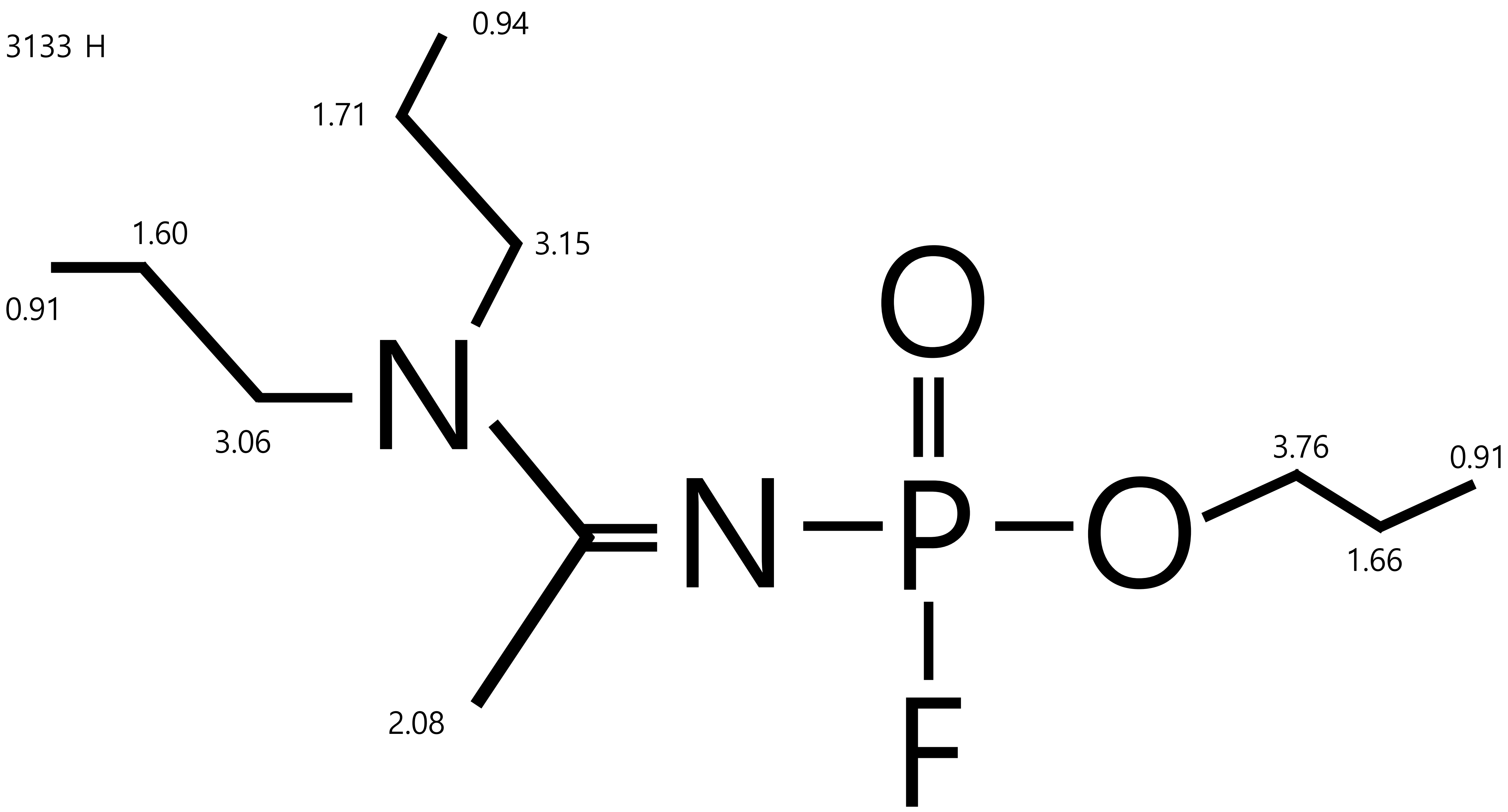

Figure S146. Structure 3133 and its ^{1}H chemical shift

3211 H

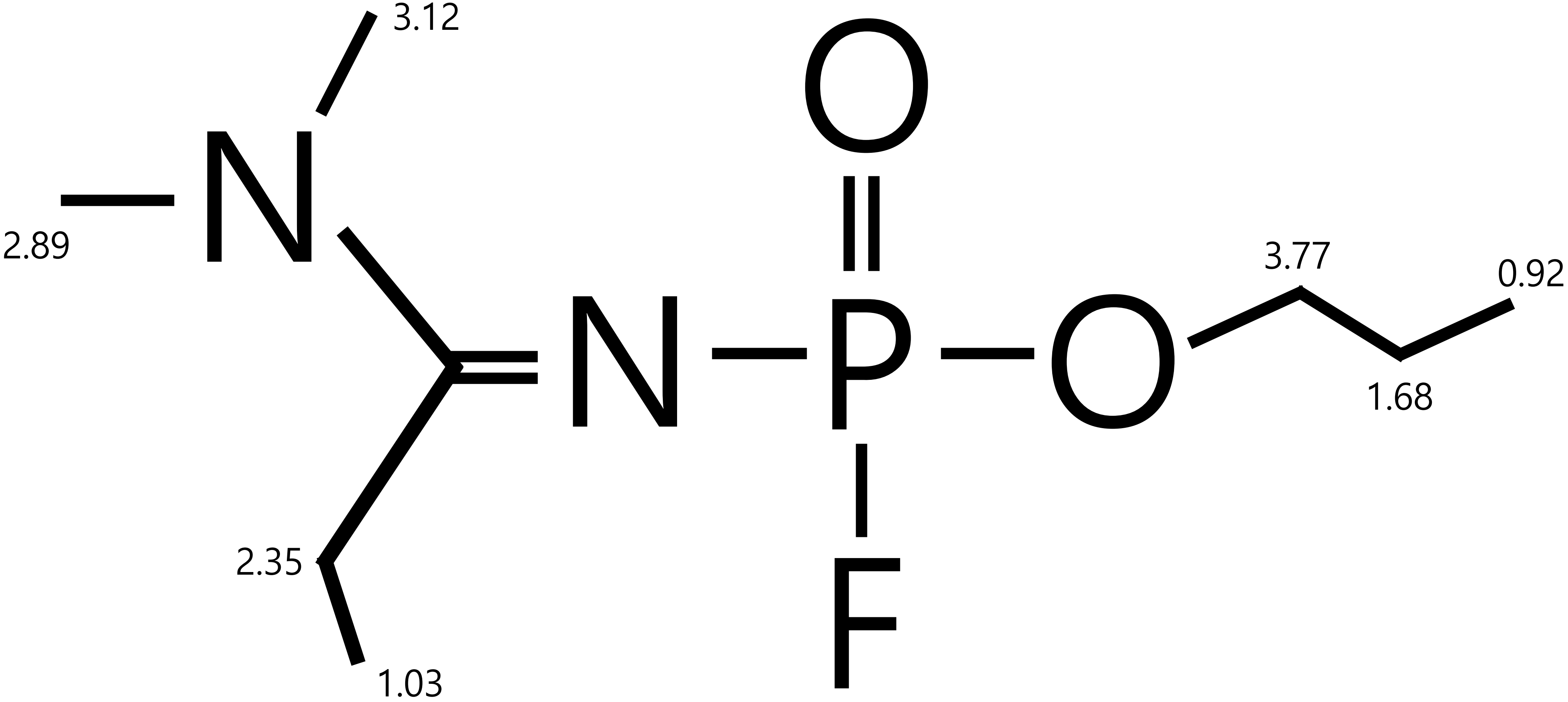

Figure S147. Structure 3211 and its ¹H chemical shift

3212 H

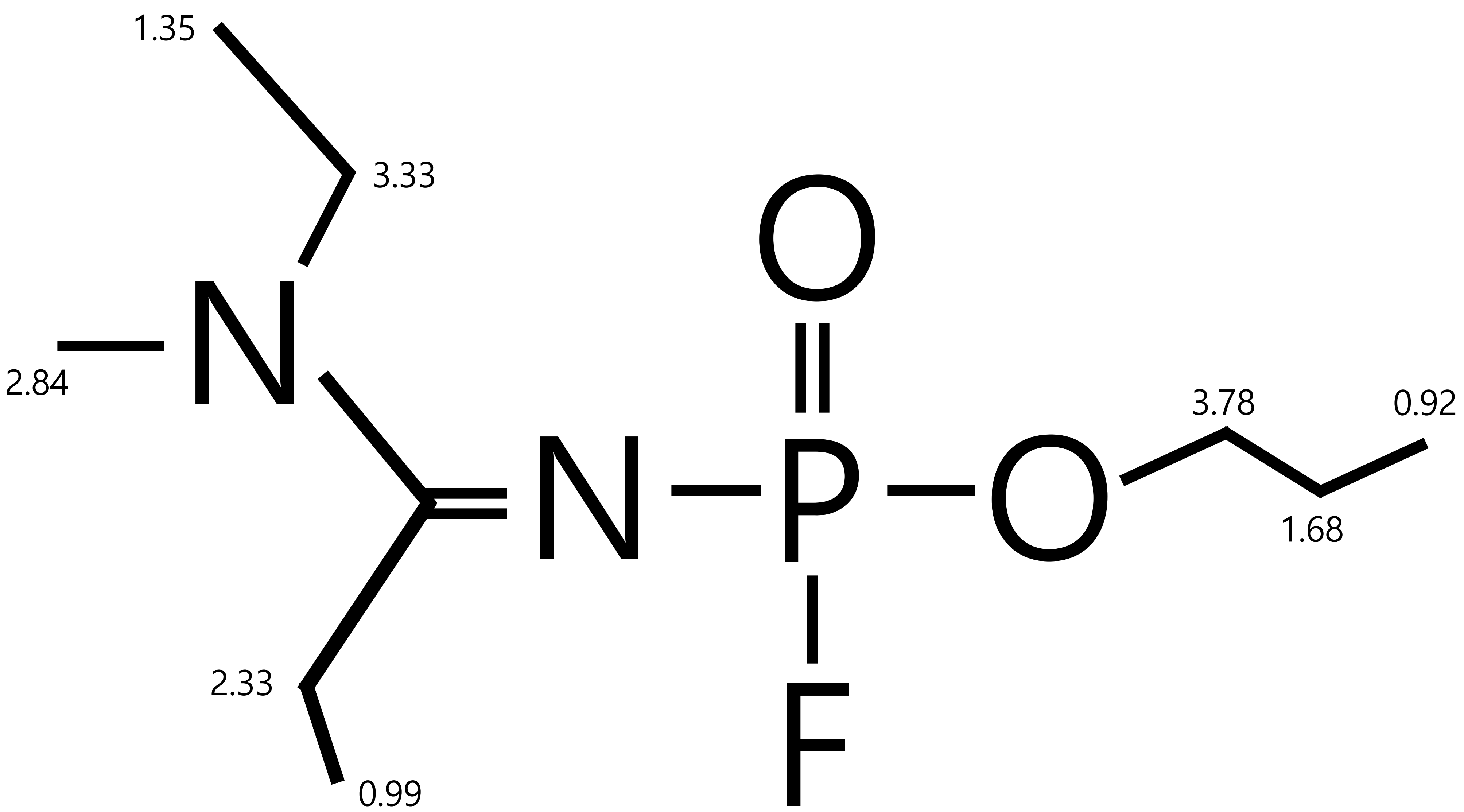

Figure S148. Structure 3212 and its ^{1}H chemical shift

3213 H

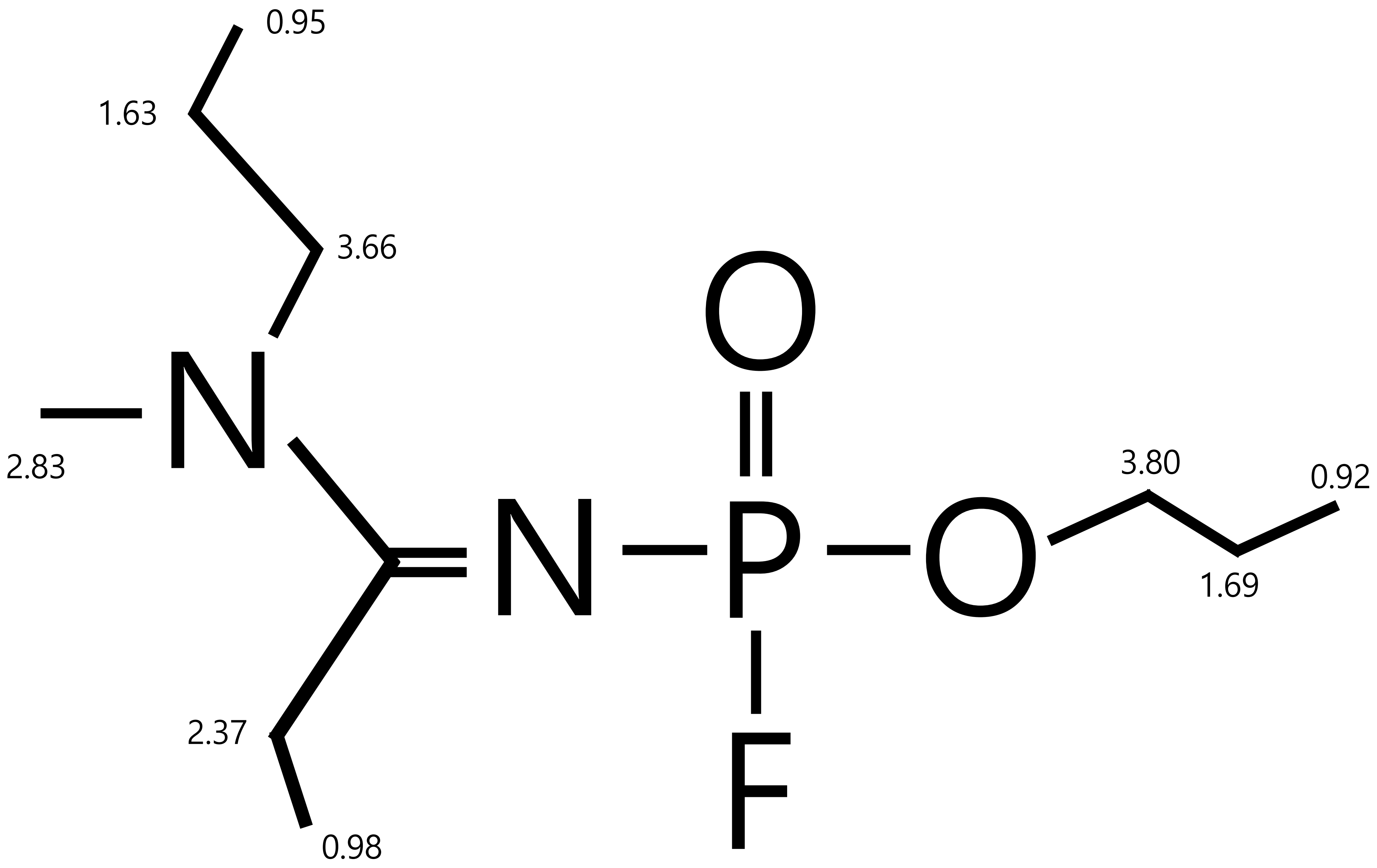

Figure S149. Structure 3213 and its ^{1}H chemical shift

3221 H

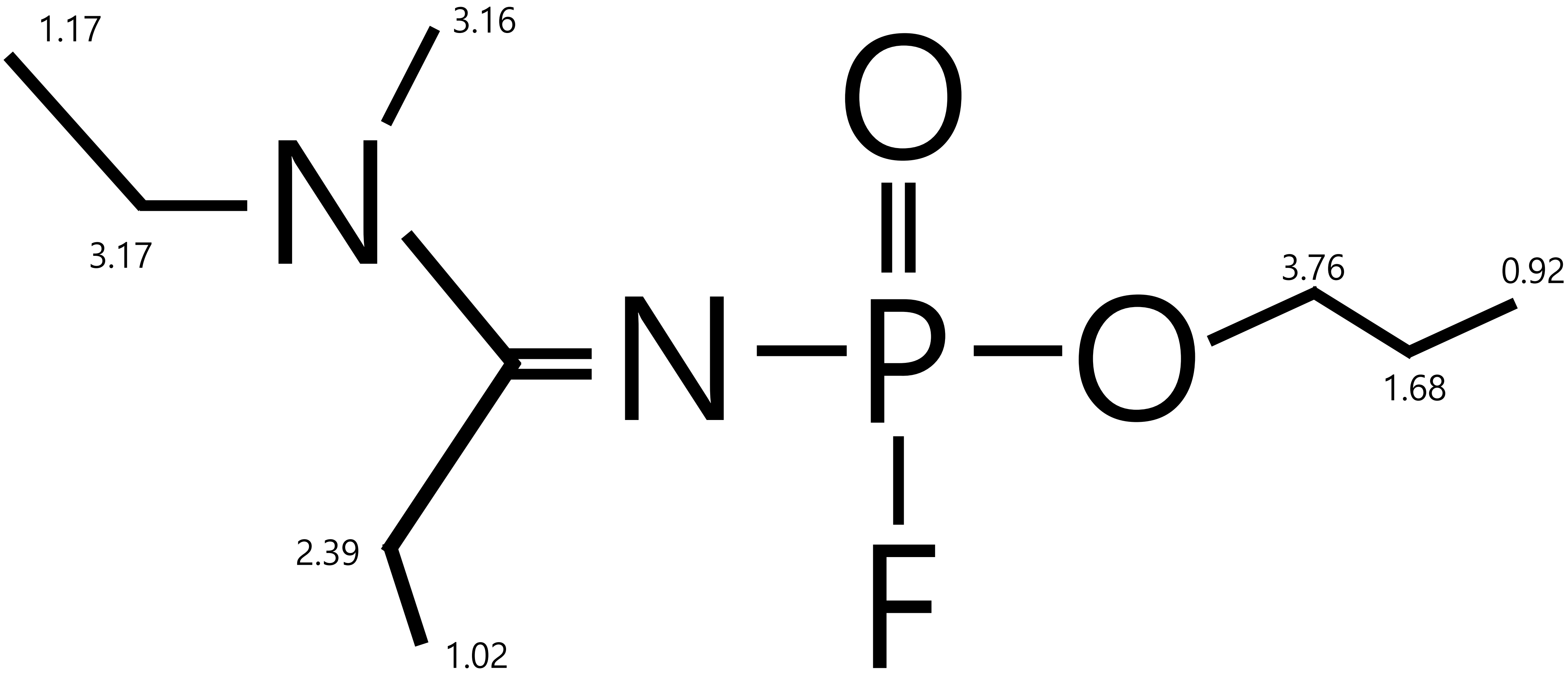

Figure S150. Structure 3221 and its ^{1}H chemical shift

3222 H

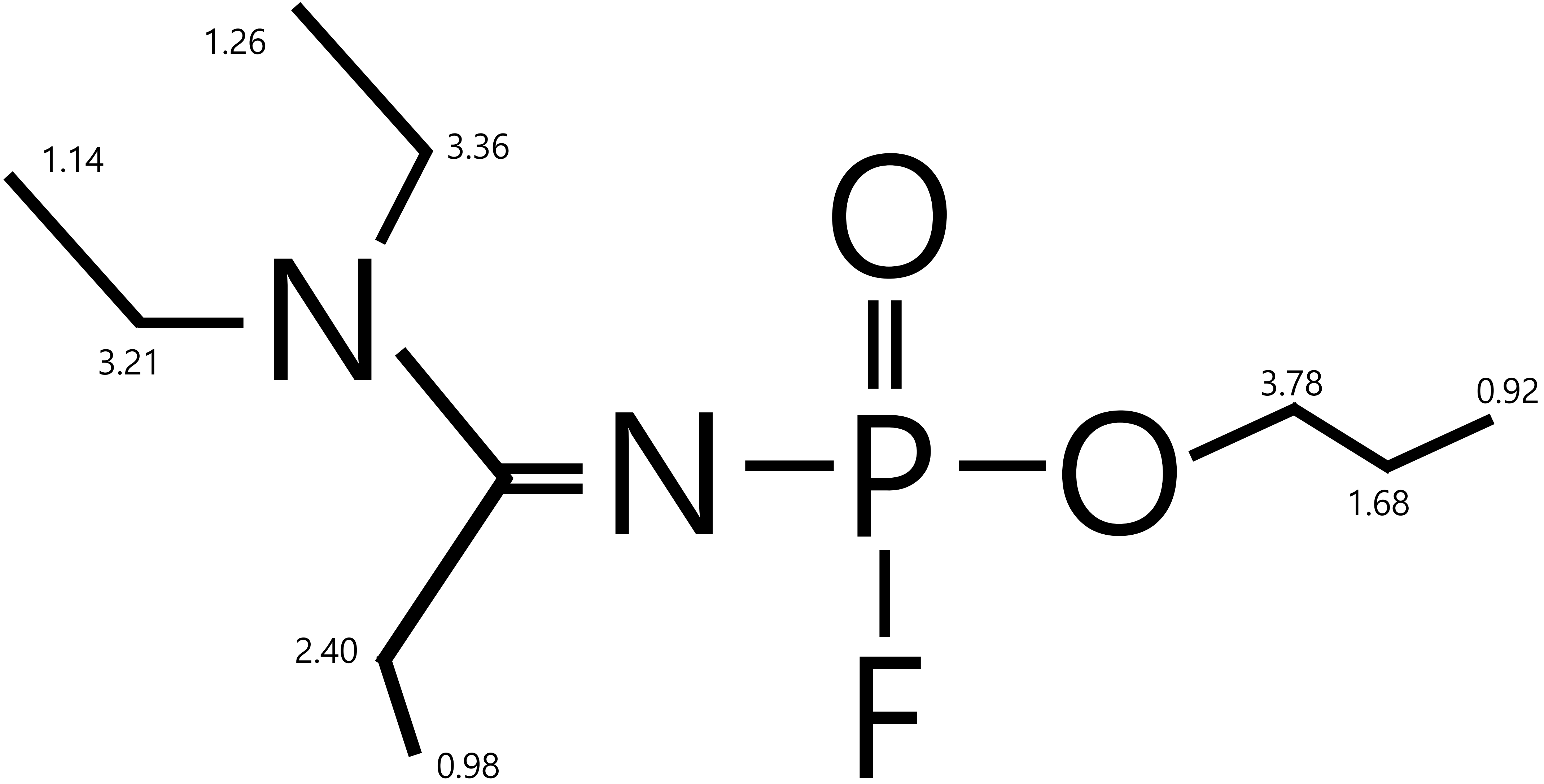

Figure S151. Structure 3222 and its ^{1}H chemical shift

3223 H

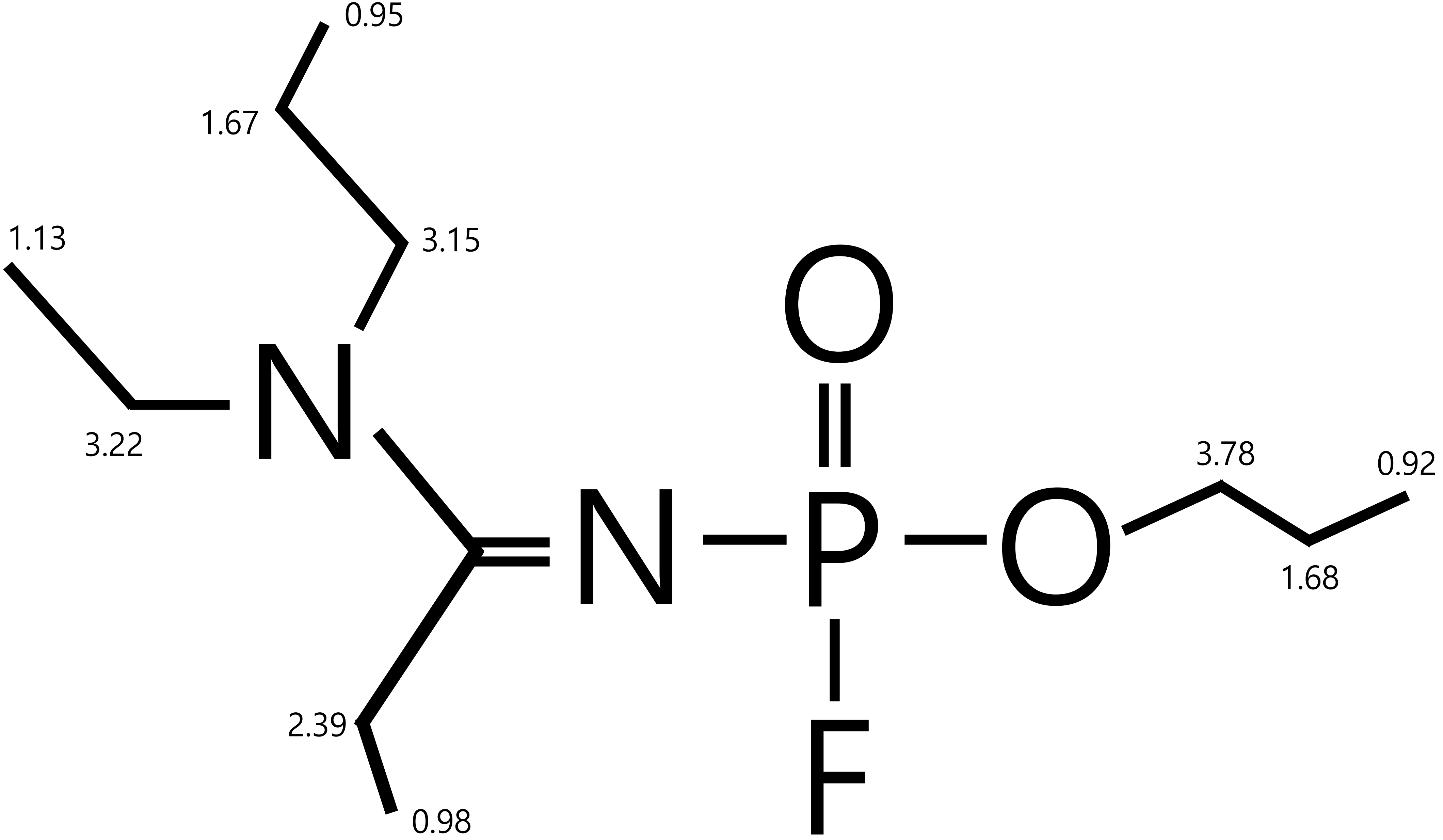

Figure S152. Structure 3223 and its ¹H chemical shift

3231 H

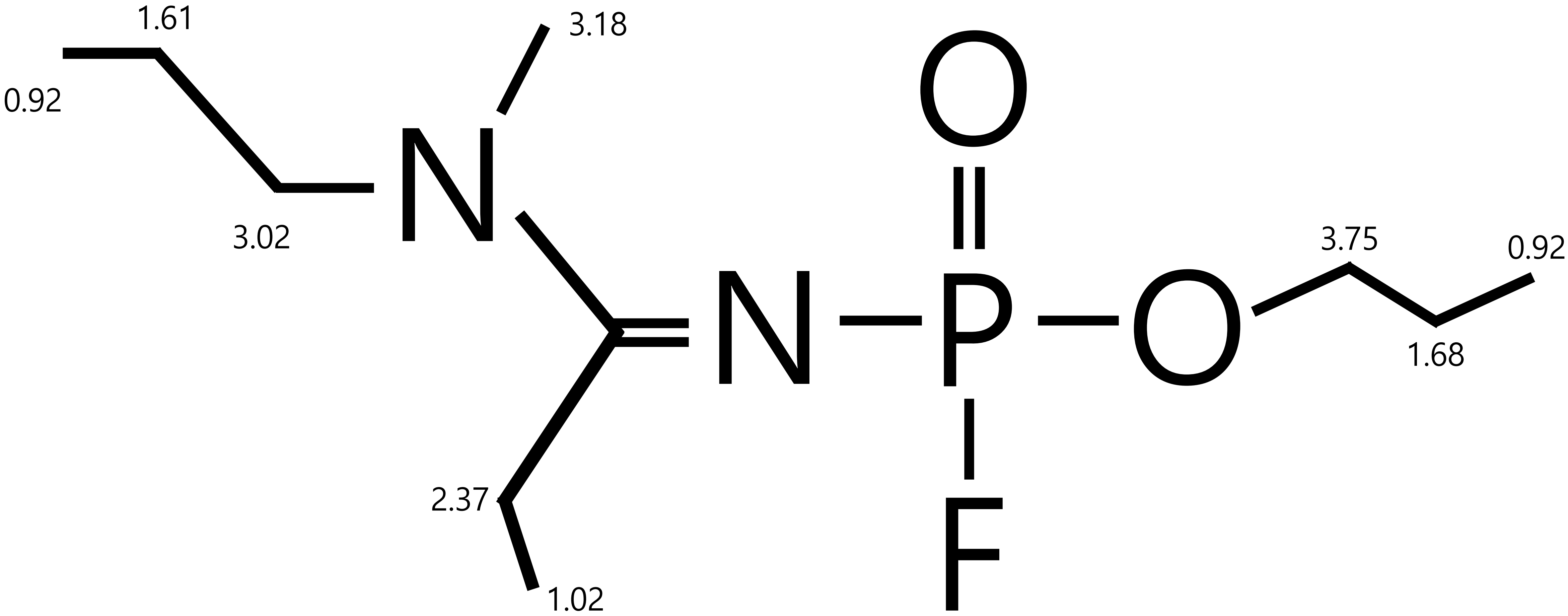

Figure S153. Structure 3231 and its ¹H chemical shift

3232 H

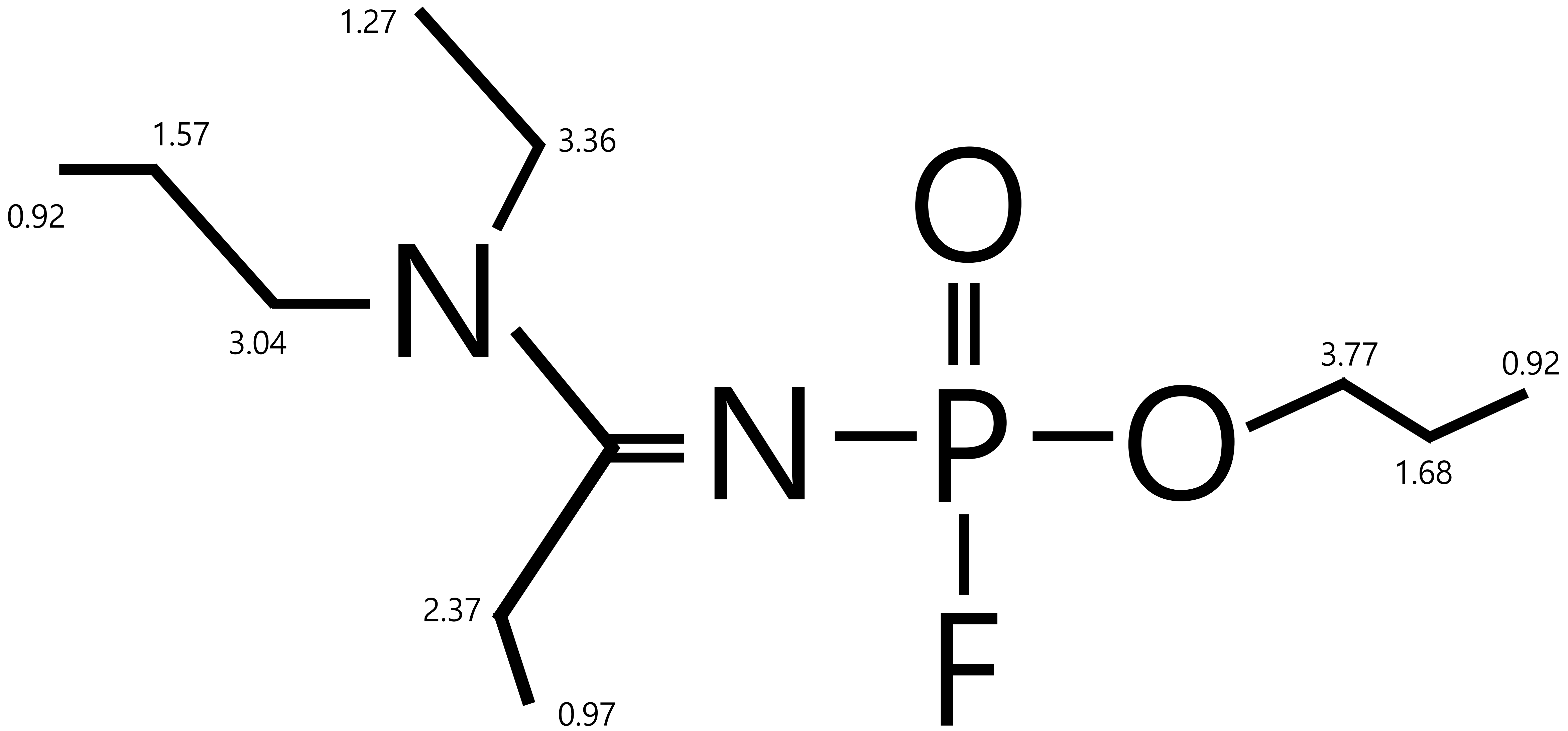

Figure S154. Structure 3232 and its ¹H chemical shift

3233 H

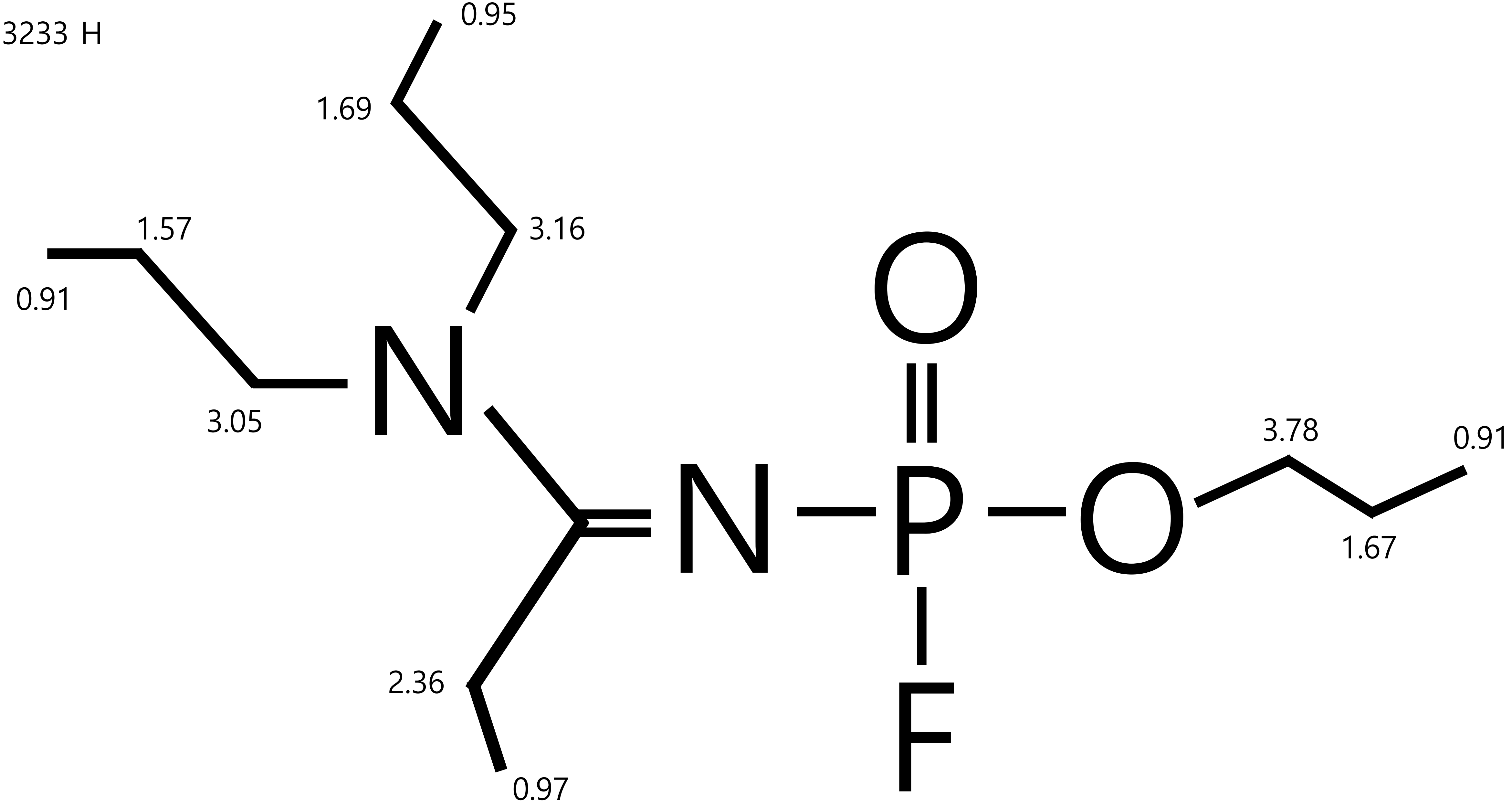

Figure S155. Structure 3233 and its ^{1}H chemical shift

3311 H

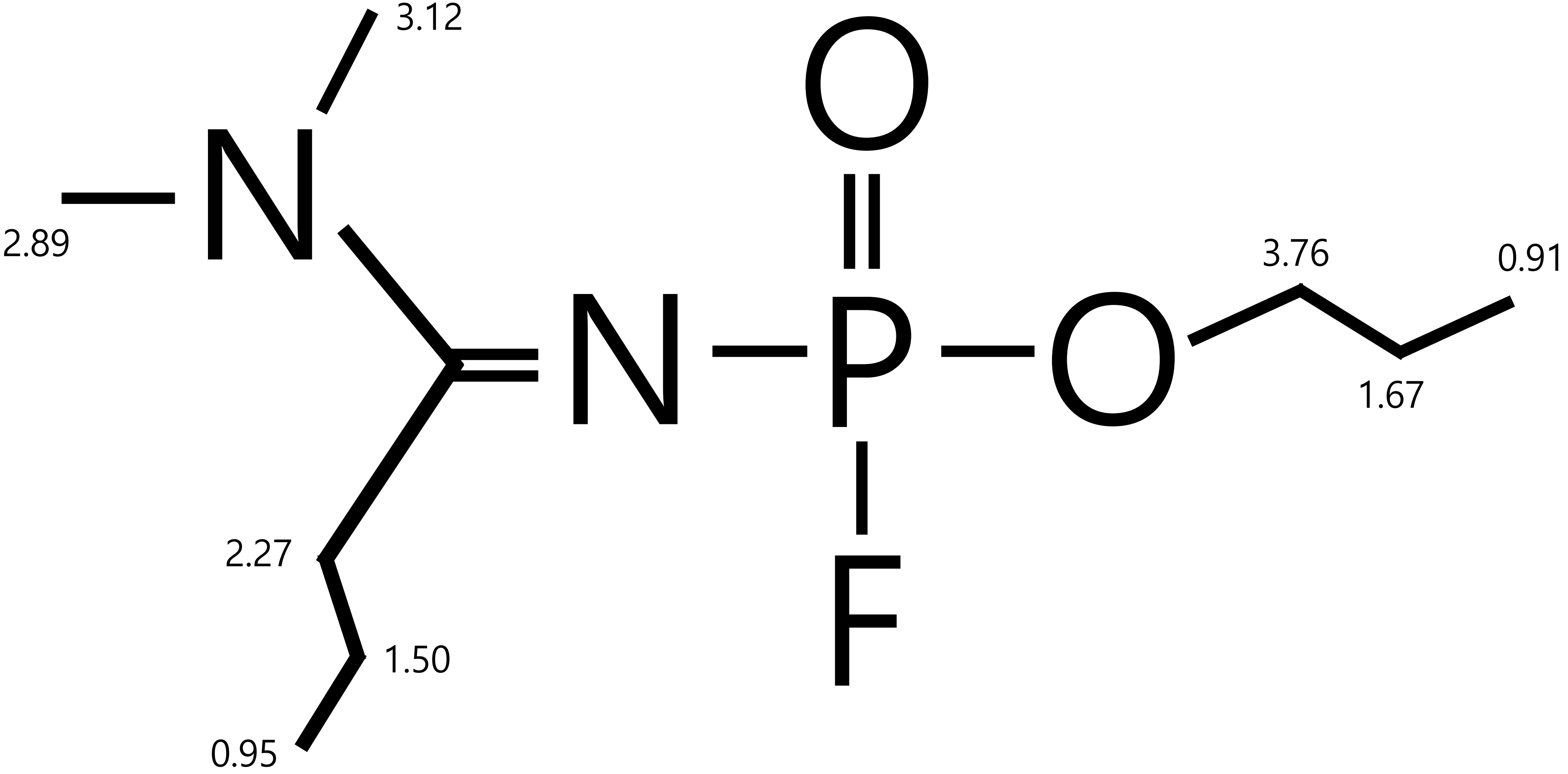

Figure S156. Structure 3311 and its ^1H chemical shift

3312 H

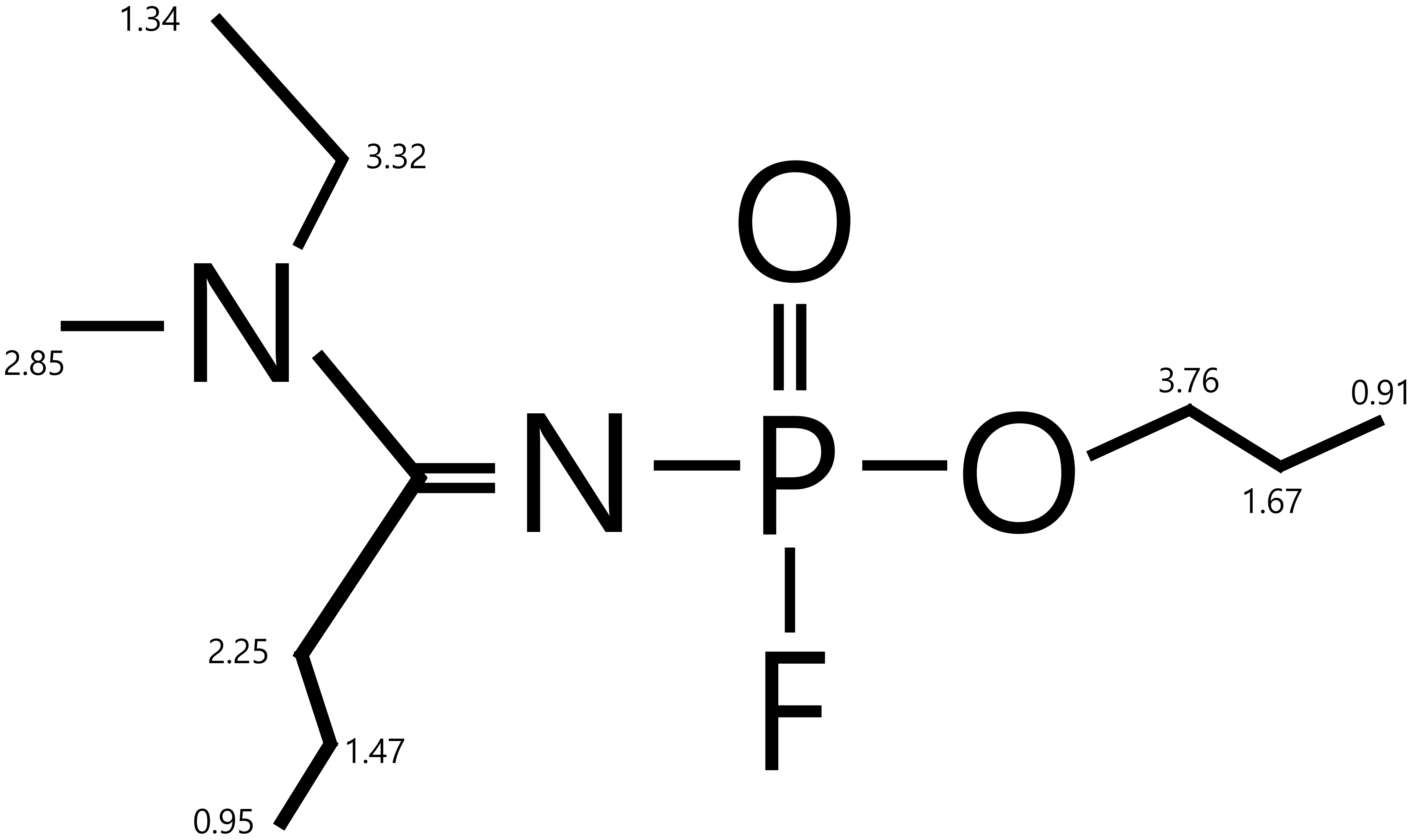

Figure S157. Structure 3312 and its ^{1}H chemical shift

3313 H

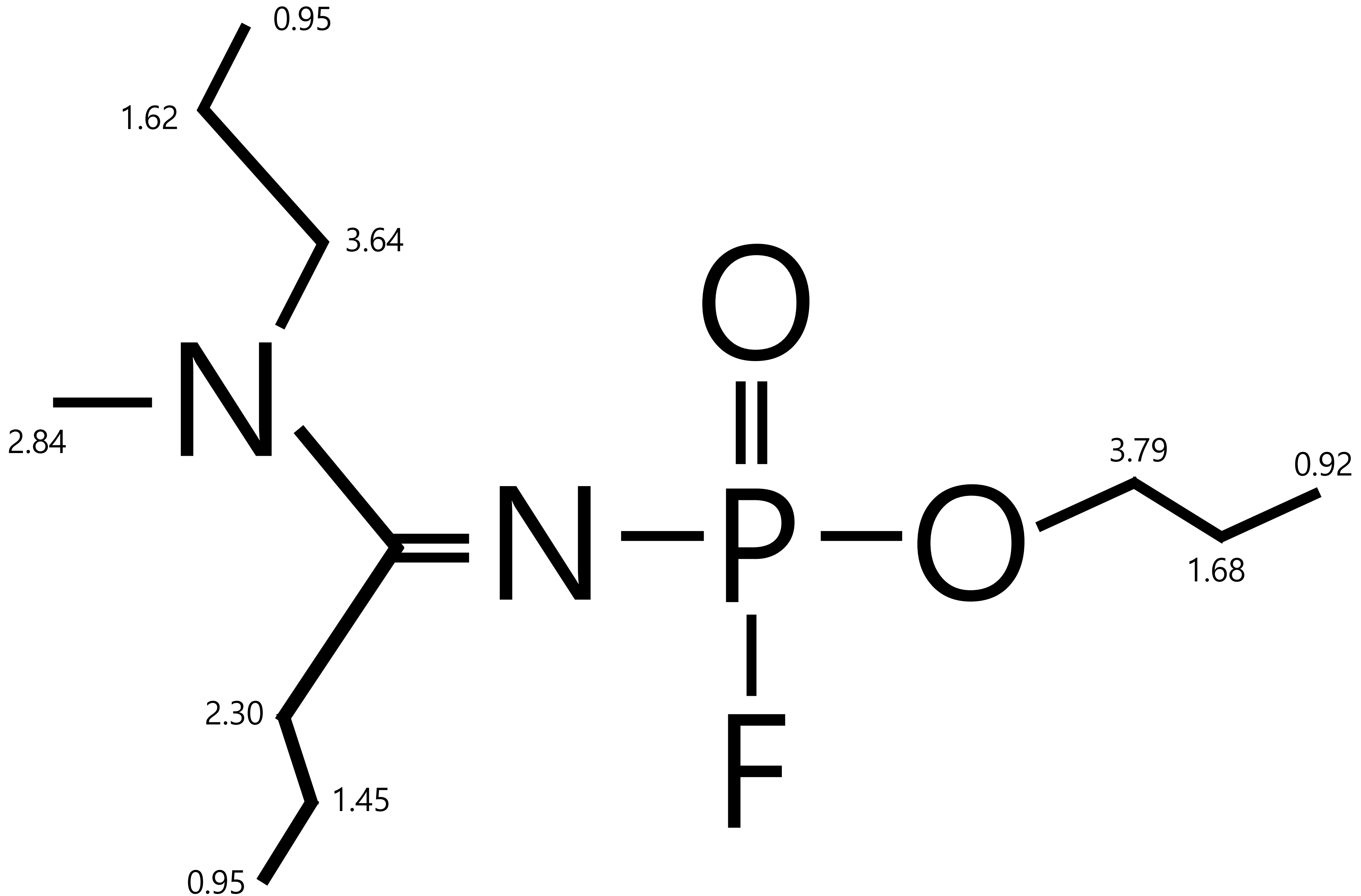

Figure S158. Structure 3313 and its ^{1}H chemical shift

3321 H

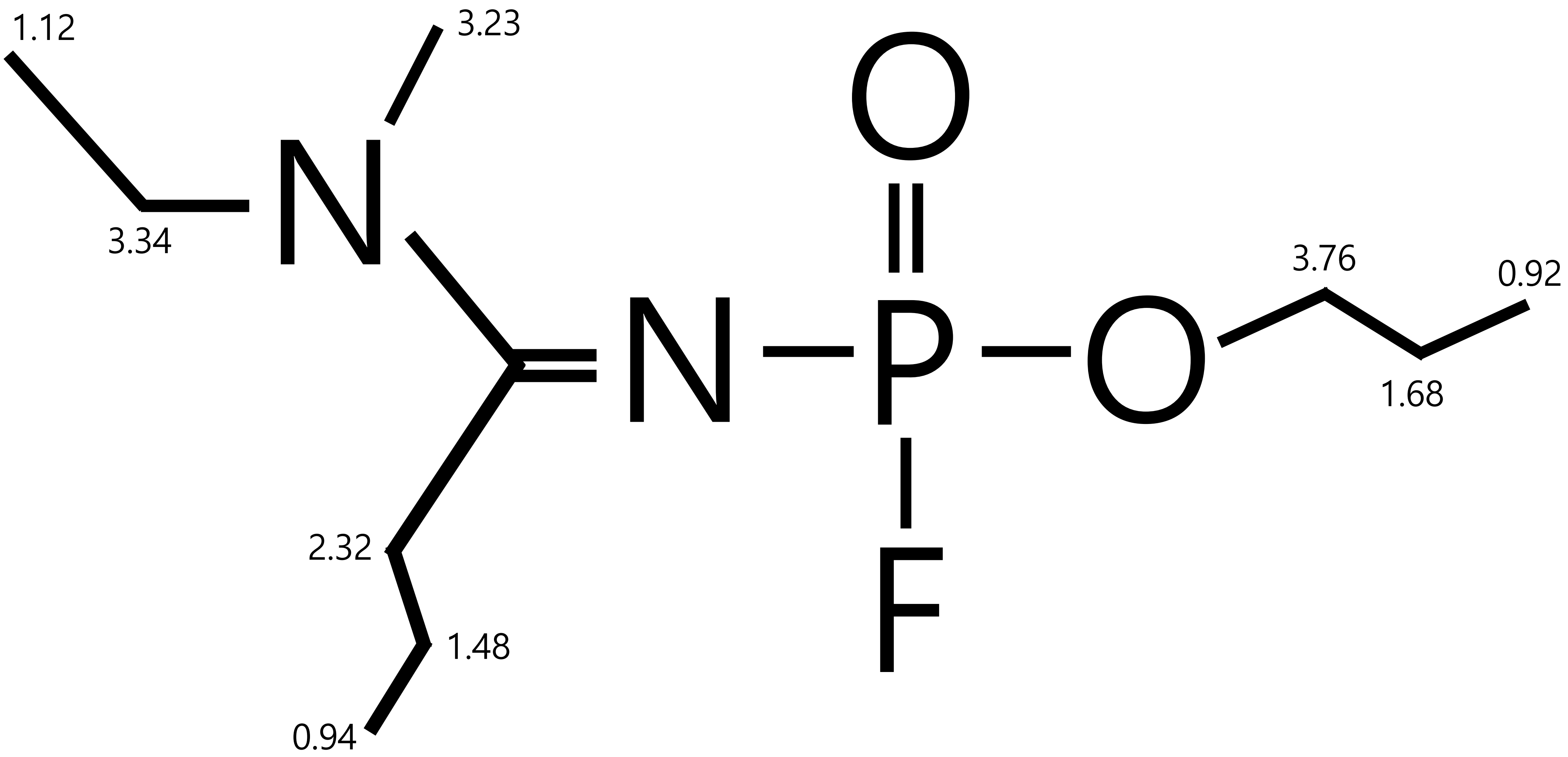

Figure S159. Structure 3321 and its ^{1}H chemical shift

3322 H

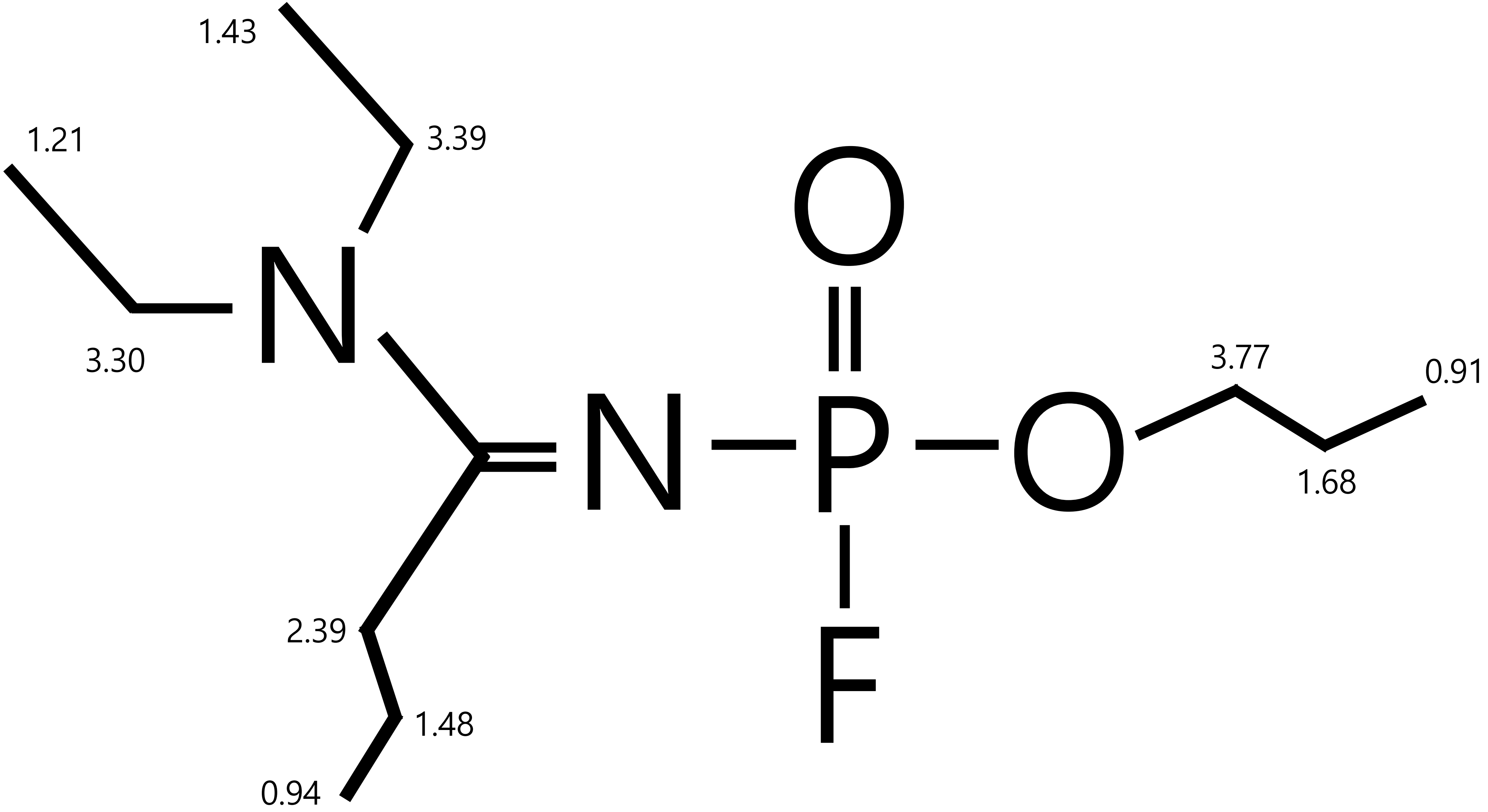

Figure S160. Structure 3322 and its ^{1}H chemical shift

3323 H

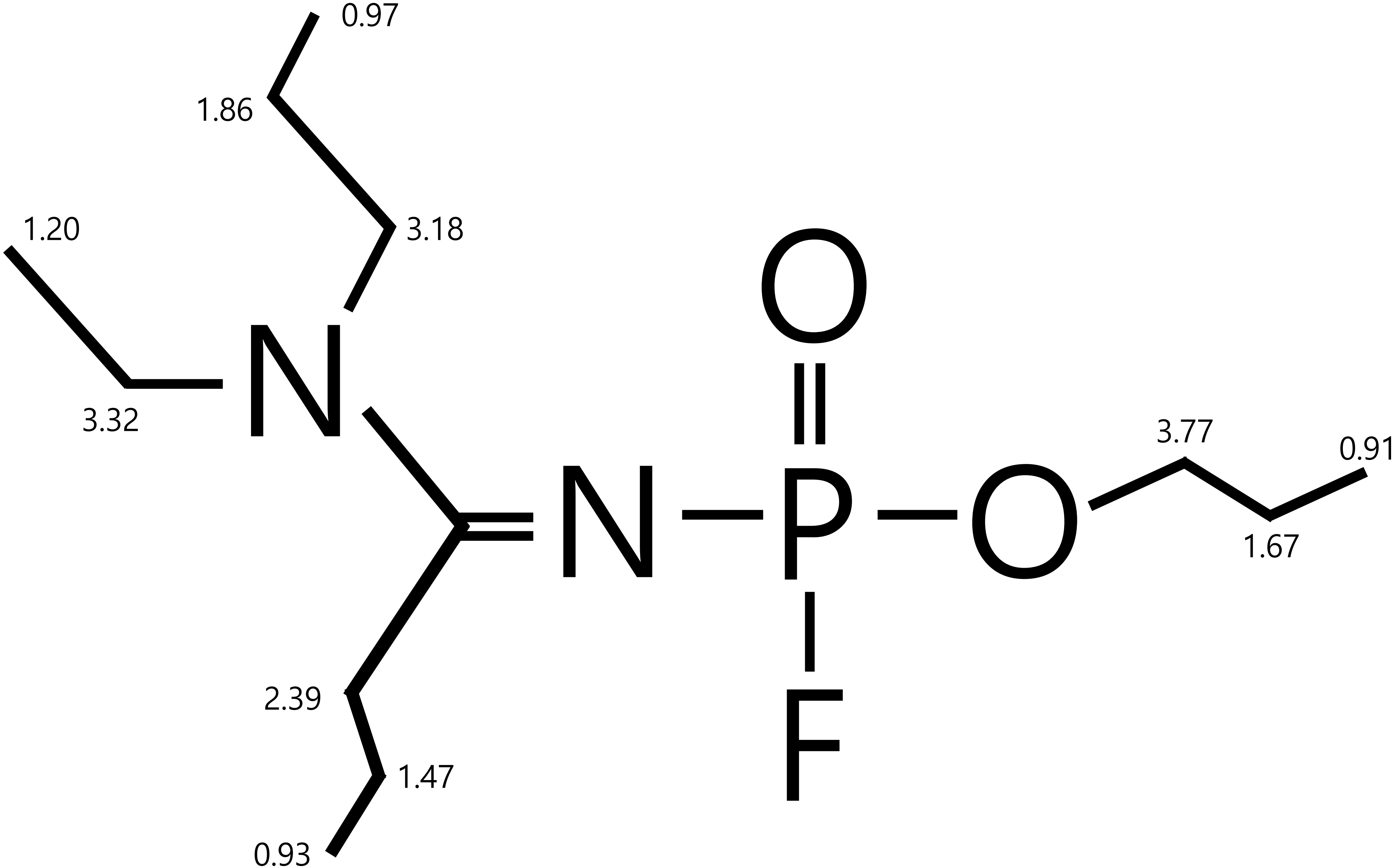

Figure S161. Structure 3323 and its ^{1}H chemical shift

3331 H

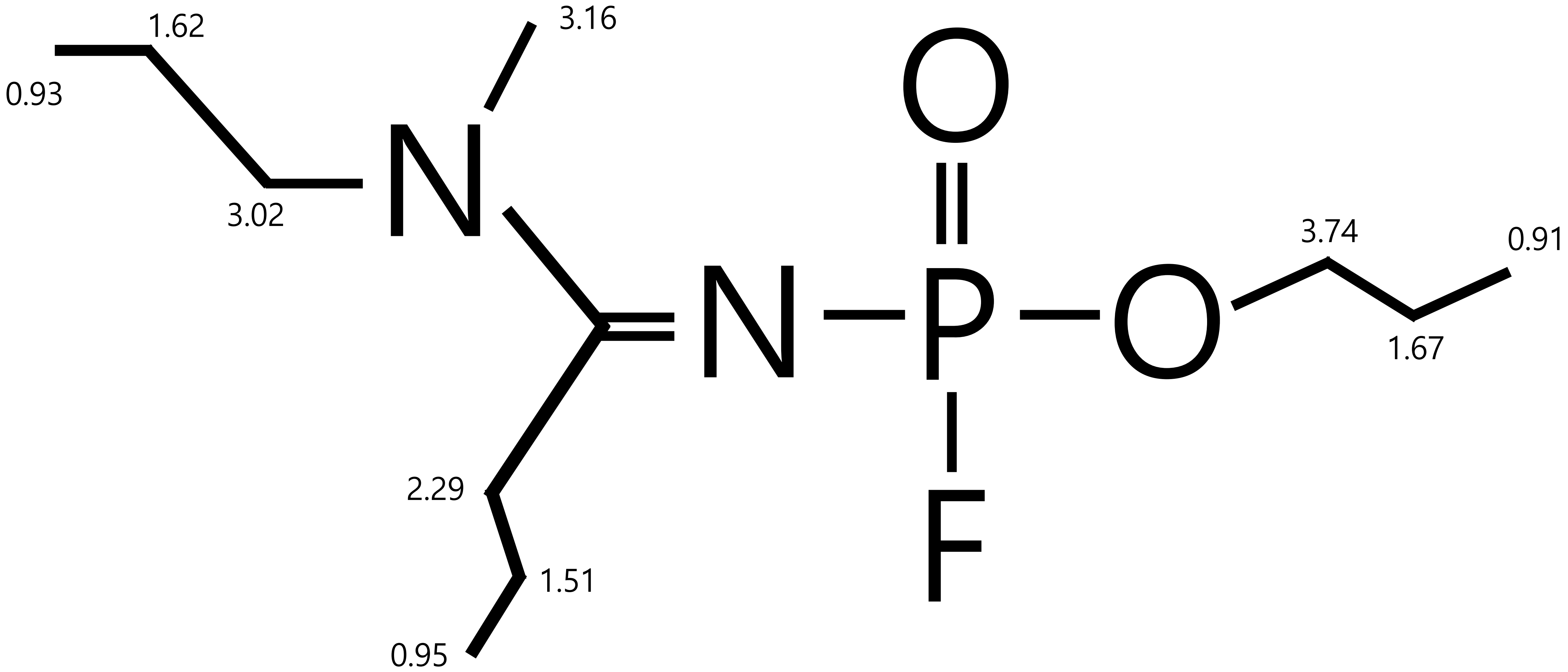

Figure S162. Structure 3331 and its ¹H chemical shift

3332 H

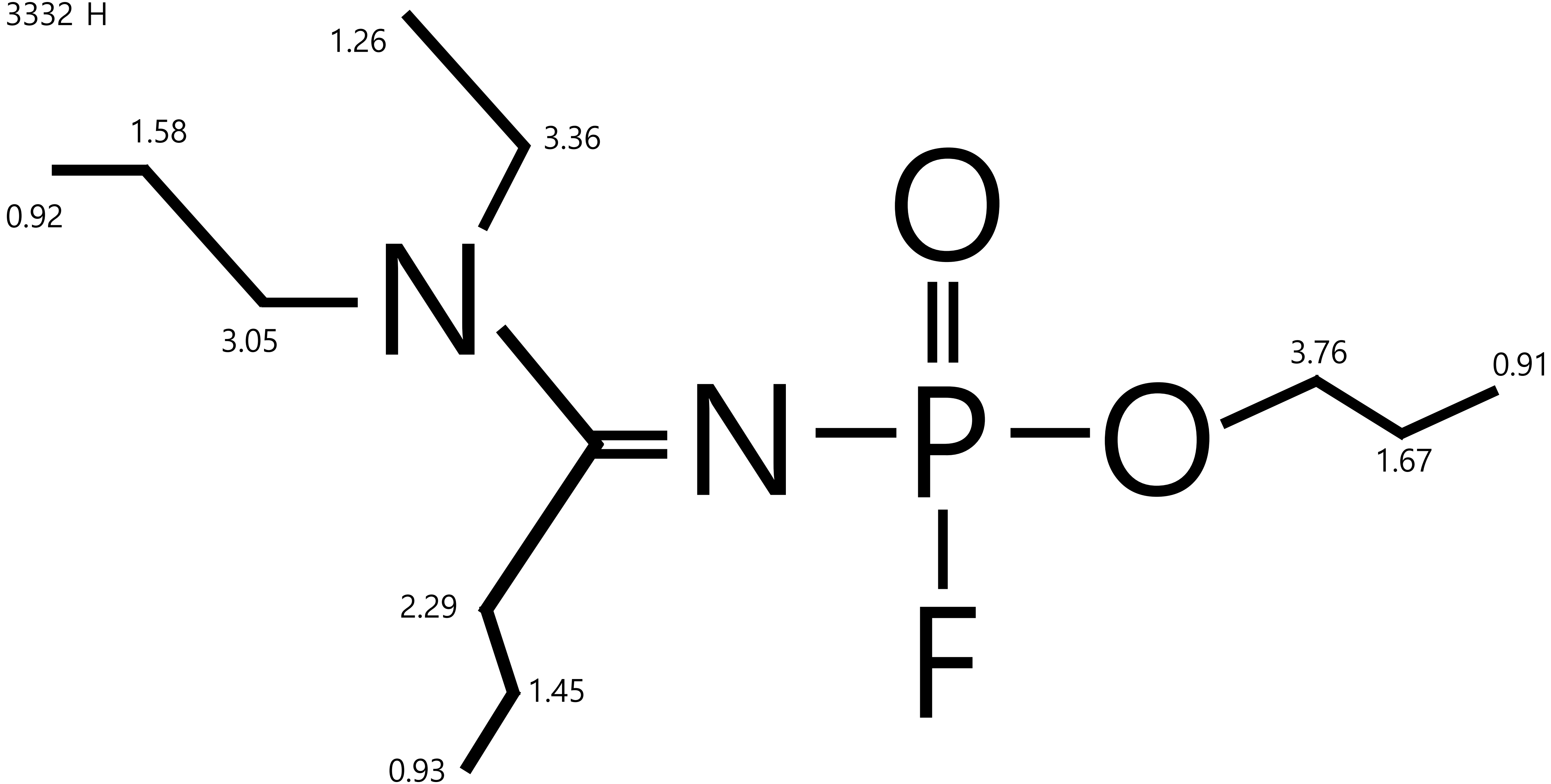

Figure S163. Structure 3332 and its ^{1}H chemical shift

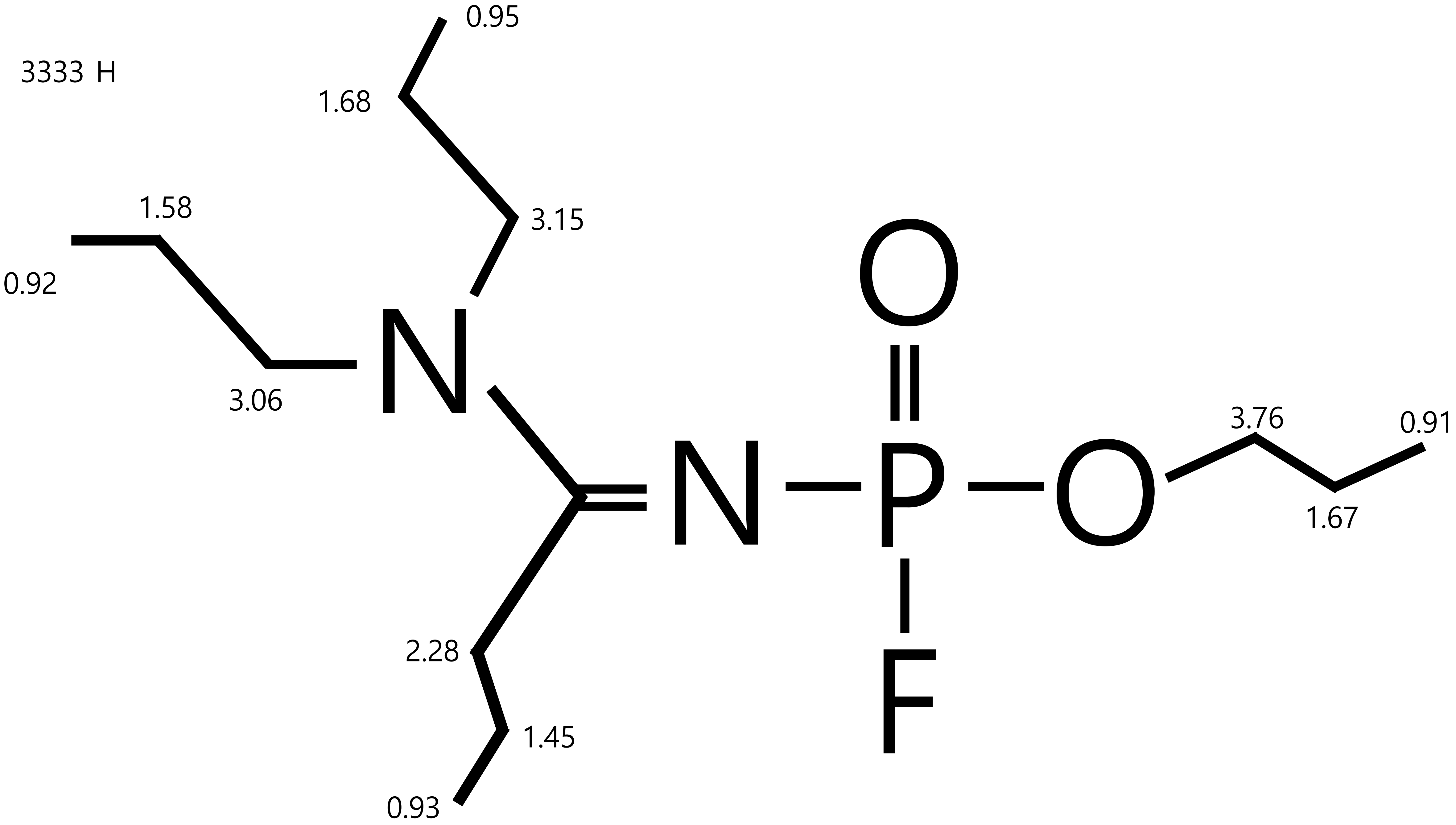

Figure S164. Structure 3333 and its ¹H chemical shift

A242 H

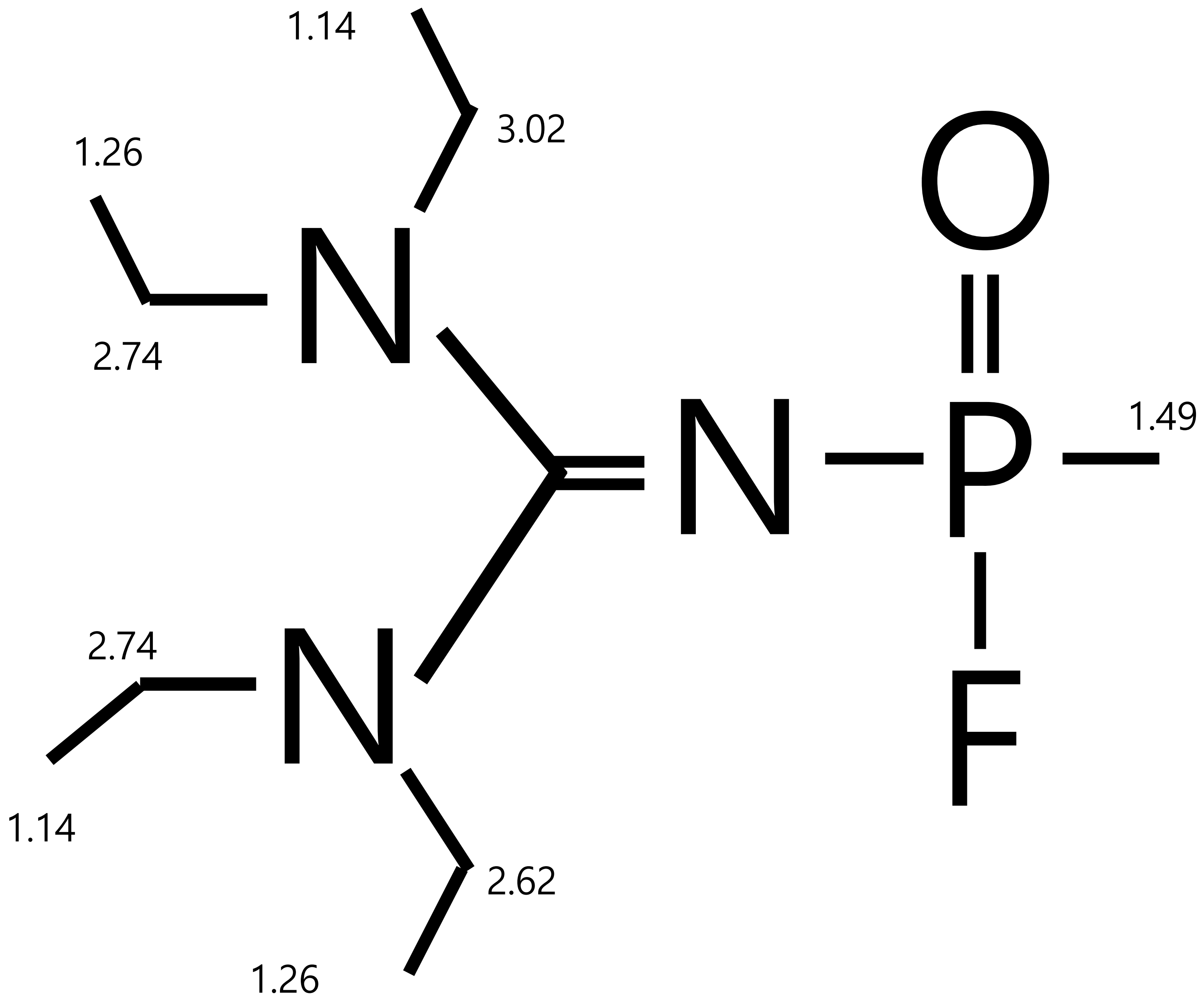

Figure S165. Structure A242 and its ^{1}H chemical shift

A262 H

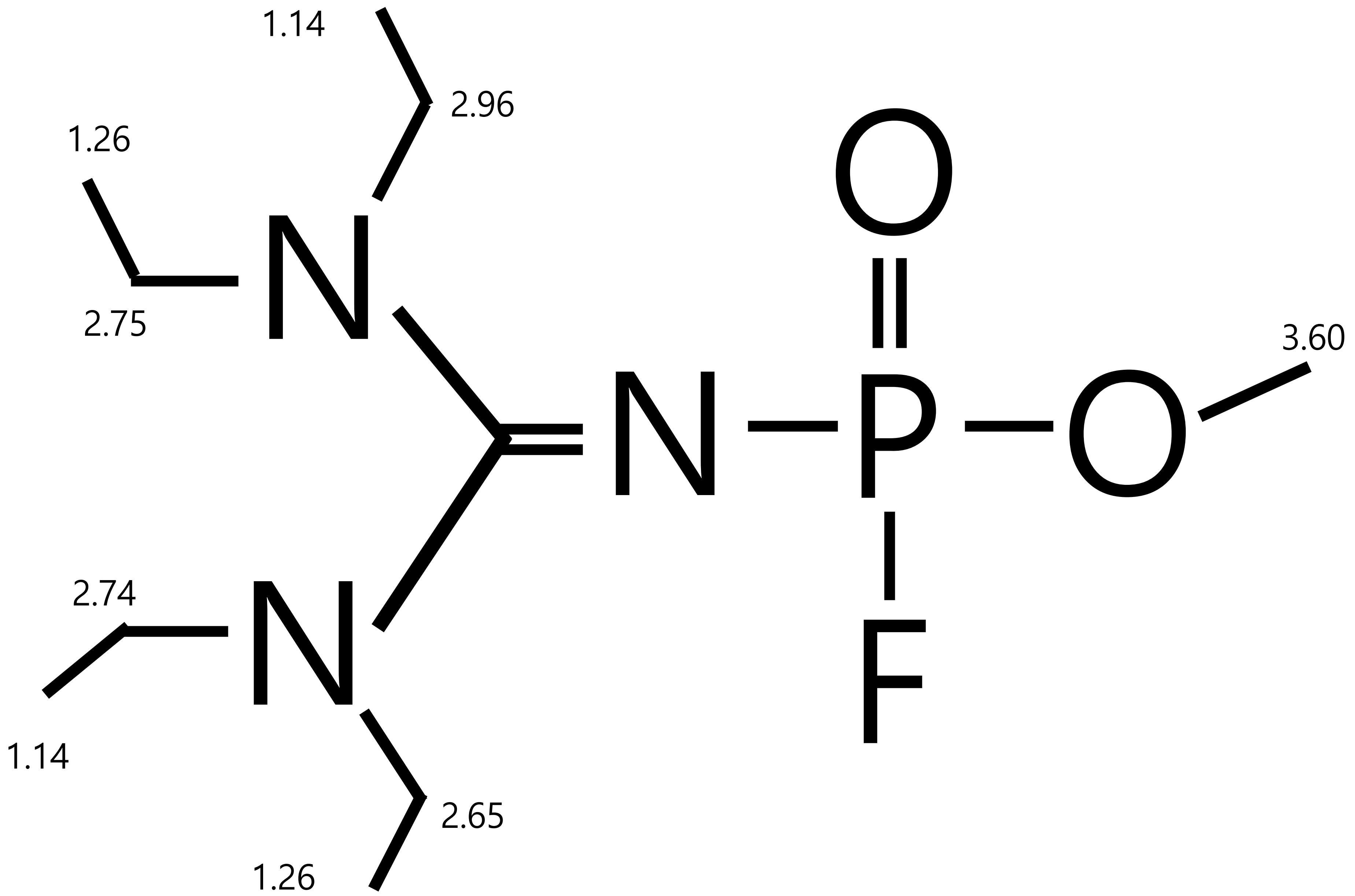

Figure S166. Structure A262 and its ¹H chemical shift
